# Atlas of diatoms (Bacillariophyta) from diverse habitats in remote regions of western Canada

**DOI:** 10.3897/phytokeys.105.23806

**Published:** 2018-07-25

**Authors:** Loren Bahls, Beverly Boynton, Barb Johnston

**Affiliations:** 1 1032 12th Avenue, Helena, Montana, 59601, USA; 2 PO Box 224, Kelly, Wyoming, 83011, USA; 3 Parks Canada, Waterton Lakes National Park, Box 200, Waterton Park, Alberta, T0K 2M0, Canada

**Keywords:** diatoms, Canada, North America, biogeography, floristics, ecology, Rocky Mountains, tundra, taiga

## Abstract

High-resolution LM images of diatoms from remote regions of western Canada are presented as a contribution to our knowledge of diatom floristics, ecology and biogeography in North America. Approximately 600 taxa are imaged in 132 plates. Genera with the most taxa are *Cymbella* (19 taxa), *Cymbopleura* (29), *Encyonema* (23), *Encyonopsis* (15), *Eunotia* (77), *Gomphonema* (42), *Navicula* (47), *Neidium* (20), *Nitzschia* (35), *Pinnularia* (50) and *Stauroneis* (34). Diatoms were collected from diverse habitats in four of North America’s major biomes: Arctic tundra, taiga, Rocky Mountains and Pacific rainforest. Many of the photographed taxa could not be identified to species and are likely new to science. Other taxa may represent new records for North America or Canada. Images of voucher specimens are keyed to individual collection sites. Detailed descriptions of the collection sites include GPS coordinates, colour photographs, vegetation, algal substrates, elevations, pH, temperature and conductivity. Samples were collected from natural substrates in fresh to brackish, flowing and standing waters. Voucher slides are deposited in the Montana Diatom Collection (Helena) and the University of Montana Herbarium (Missoula). Cleaned diatom frustules have been deposited in the Diatom Herbarium of the Academy of Natural Sciences of Philadelphia.

## Introduction

**Figure 1.**
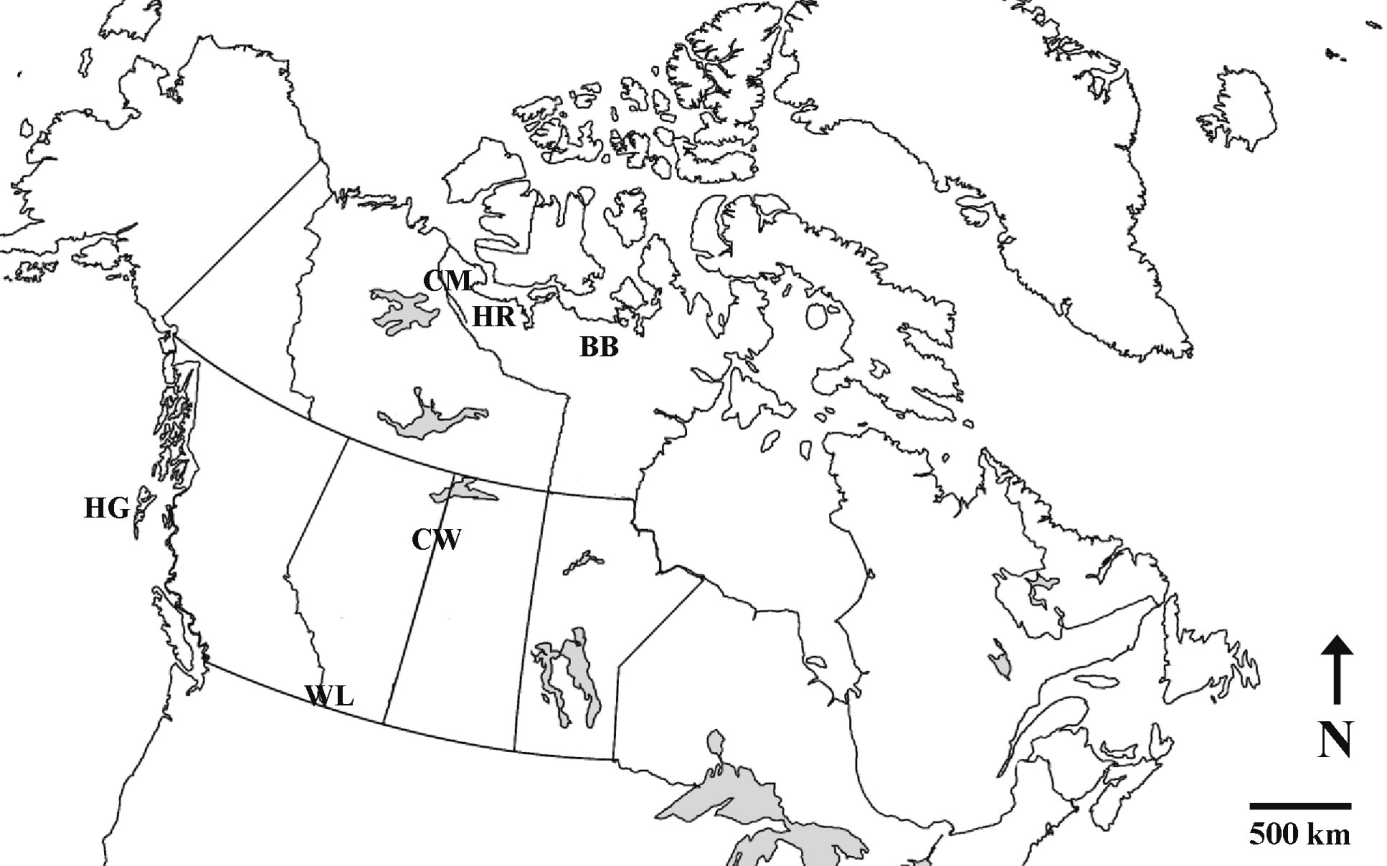
Map of Canada showing diatom collection areas. BB = Baillie and Back Rivers, CM = Coppermine River, CW = Clearwater River, HG = Haida Gwaii, HR = Hood River, WL = Waterton Lakes National Park. Source of base map: www.printablemaps.net

From 2009 to 2017, the authors collected 96 diatom samples from a variety of fresh- and brackish-, standing- and flowing-water habitats in remote regions of western Canada (Fig. 1). Collection sites were located in four of North America’s major biomes: Arctic tundra, taiga, Rocky Mountains and Pacific rainforest. Representative specimens from those collections are presented here in a series of high-resolution photographic plates as a contribution to our knowledge of diatom floristics, ecology and biogeography in North America.

This atlas is intended as a resource for the study of diatom floristics, diatom biogeography and diatom ecology. It is not intended as a taxonomic resource nor as a definitive account of taxa richness. Priority was given to imaging unknown, unusual, uncommon and visually distinctive taxa and, of the ones that were photographed, only clean, good quality images were used. If a good quality image of a taxon was not available, we did not include that taxon in the atlas. For this reason, we expect that most of the included taxa can be identified from the images alone. For practical reasons, images are presented without written descriptions.

This atlas of diatom images is comparable to Schmidt’s Atlas (A. Schmidt 1874–1959), but with three significant differences: (1) this atlas addresses only diatoms from western Canada; (2) illustrations are high-resolution (600 dpi) digital images rather than line drawings; and (3) detailed descriptions are provided for many of the collection sites, including GPS coordinates, colour photographs, terrestrial and aquatic vegetation, algal substrata, elevations, pH, water temperature and conductivity. This atlas might also be thought of as a preliminary illustrated checklist of diatoms from western Canada that are included in the Montana Diatom Collection.

## Methods

Samples were collected from rivers, streams, lakes, pools, bogs, fens, beaver ponds and wet meadows in six regions of western Canada (Fig. 1). Most of the samples are from remote locations accessible only by hiking or by kayak or canoe. Elevations at collection sites range from sea level to over 2000 m a.s.l. Many of the sampled water bodies are not named on topographic maps. In these cases, locally descriptive place names are provided by the sampler. Temperature, pH and conductivity were measured in the field with an YSI 556 Multi Probe System or a Eutech Instruments Oakton pH Tester, Model 30. Colour photographs were taken of most collection sites. The Oakton pH meter was calibrated once annually before each field season against standard solutions of pH 4.01 and pH 7.01. However, sampling trips were long and conducted under challenging conditions. The meter may have gone out of calibration and readers should be cautioned about readings other than those taken in Waterton Park.

At each site, diatoms were collected from all available near-shore substrata, including aquatic macrophytes, mosses, rocks, fine sediments and woody debris. Subsamples were combined with ambient water in a single container and preserved with iodine before transport to the laboratory, where they were treated with 30% hydrogen peroxide (H_2_O_2_) to remove organic matter. After several rinses in distilled water, cleaned diatom material was dried on cover slips and mounted permanently on glass slides using Naphrax.

Slides were examined under LM with differential interference contrast optics and images were captured using a Leica DM LB2 research microscope and a Spot Insight monochrome digital camera (Model 14.0). Slides examined for this study are deposited in the Montana Diatom Collection (MDC) in Helena and the University of Montana Herbarium in Missoula (MONTU). Vials of cleaned and dried diatom frustules have been deposited in the Diatom Herbarium of the Academy of Natural Sciences (Drexel University) in Philadelphia.

Imaged diatoms were identified by the first author to the lowest practical taxonomic unit using available identification resources. None of the species is described as new to science, but many are designated as unknowns (e.g. *Navicula* sp.) or as comparable to another taxon (cf.). Alternative identifications by reviewers (Acknowledgements) are given in brackets in the figure captions along with the reviewer’s initials, thereby giving readers other options for identification.

## Format

In keeping with the intended use of this atlas as a resource for the study of diatom floristics and biogeography, images of taxa are presented separately for each of the six regions (Fig. 1). Regions (site descriptions and diatom plates) are presented in the order in which they were sampled, first Waterton Lakes National Park (2009), followed by Haida Gwaii (2013, 2017), Clearwater River (2014), Coppermine River (2015), Baillie-Back Rivers (2016) and Hood River (2017). A master list of taxa and index to the plates is provided in a table at the end of the atlas (Appendix 2).

Taxa are presented in general phylogenetic order following the classification scheme of Round et al. (1990). Centric diatoms are presented first, then araphids, then monoraphids and finally biraphids. The order of genera in the biraphid group may vary from region to region. An effort was made to group species of the same or similar genera on the same plate, but sometimes species of dissimilar genera are presented on the same plate in order to conserve space.

When possible, multiple specimens of a taxon are presented in size-reduction series. However, this was not always possible for rare and uncommon taxa. In general, the number of specimens displayed on a plate is proportional to the relative abundance of that taxon in that region.

## Descriptions of collecting sites

The following sections describe the geographic regions that were sampled and the collecting sites in each region.

### Waterton Lakes National Park (Figs 2–14, Appendix 1: Plates 1–17)

Waterton Lakes National Park protects 505 km^2^ of the southern Canadian Rocky Mountains in south-western Alberta, ranging in elevation from 1,290 to 2,910 m a.s.l. The park forms part of the Crown of the Continent ecosystem, where several different ecological regions meet, leading to extremely high biological diversity. Here you can find species from the Great Plains, the Rocky Mountains and the Pacific Northwest. The park contains 45 different vegetation types, including grasslands, shrub lands, wetlands, lakes, spruce-fir, pine and aspen forests and alpine areas. Waterton lies within the Canadian Rockies ecoregion, which extends into western Montana as Glacier National Park and the Bob Marshall Wilderness complex.

The following description was taken from Woods et al. (2002):

“*The Canadian Rockies ecoregion is composed of high, wet mountains. Significant portions are covered by snowfields and glaciers. Glaciated terrain is common and characterized by U-shaped valleys, moraines, cirques, tarns, and outwash features. This ecoregion extends into northern Montana from Alberta and British Columbia. The ecoregion is generally higher and more snow- and ice-covered than the Northern Rockies, and portions are strongly influenced by moist maritime air masses. Melting snow and rainfall are abundant at higher elevations. Some surplus water is stored in glacial deposits, unconsolidated mountain valley fill, and permeable sedimentary rocks. However, areas underlain by crystalline rocks lack sufficient groundwater storage capacity to prevent overland runoff or to develop groundwater supplies; in these places, base flow is meager and high elevation streams generally flow only during rain and snow melt periods. The highest elevations are treeless, glaciated alpine areas. The potential natural vegetation is mostly subalpine fir, Douglas-fir, and Engelmann spruce. Soils are thin or absent on upper mountain slopes but become deeper and more developed below, especially west of the Continental Divide. Recreation, forestry, and mining are common land uses*”.

**Figure 2.**
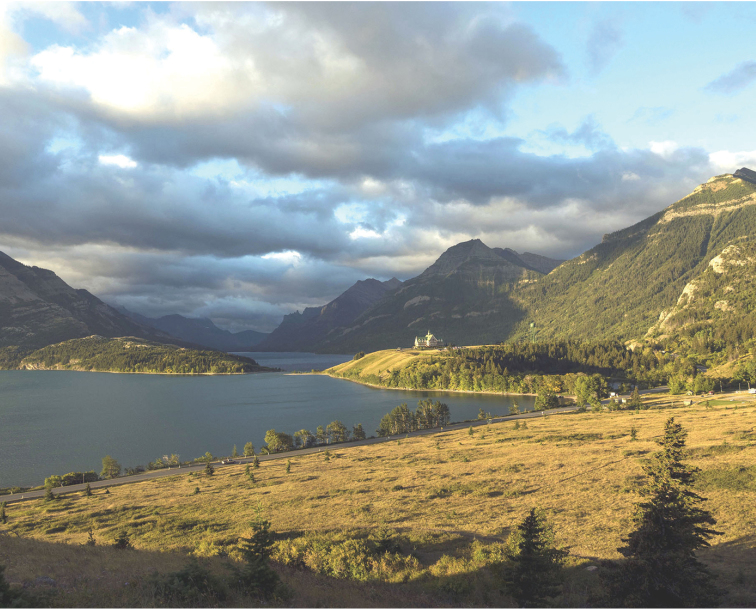
The centrepiece of Waterton Lakes National Park is Waterton Lake, which extends southwards for 11 km from the Prince of Wales Hotel in Alberta, Canada (right centre) to the Goat Haunt Ranger Station at the far end of the lake in Montana, USA (middle distance). Waterton Lake at Goat Haunt is the type locality of *Cymatopleura internationale* Bahls (2013). Photo credit: Parks Canada.

**Figures 3–8.**
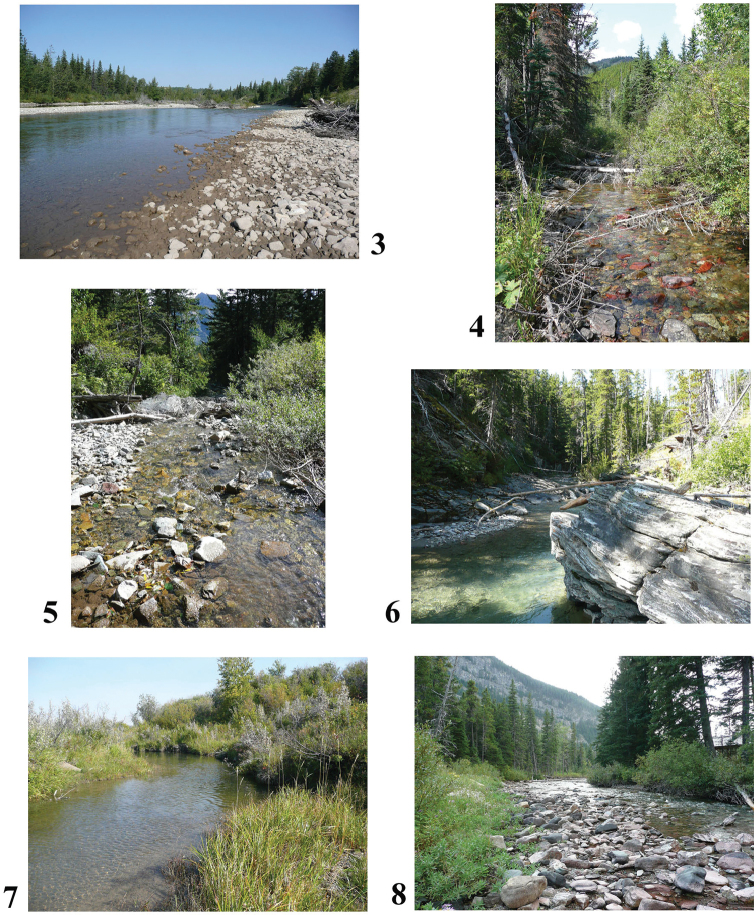
Representative flowing-water habitats sampled for diatoms in Waterton Lakes National Park **3** Belly River (4547) **4** Rowe Creek (4563) **5** Bertha Creek (4555, 4556) **6** Bauerman Creek (4565, 4566) **7** Crooked Creek (4559) **8** Cameron Creek (4546, 4548). Photos credit: Parks Canada.

**Figures 9–14.**
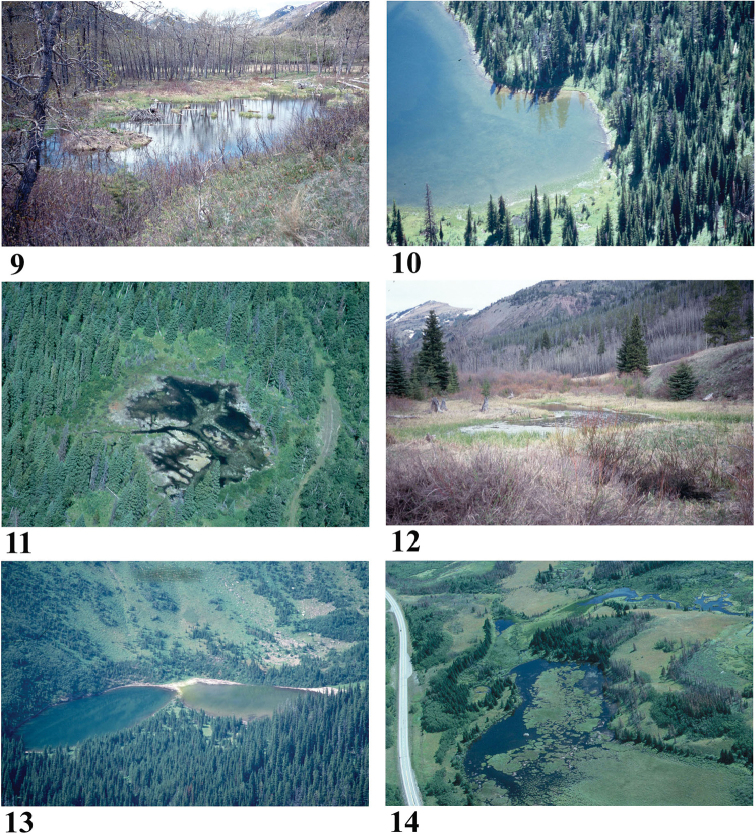
Representative standing-water habitats sampled for diatoms in Waterton Lakes National Park **9** Blakiston Beaver Pond B (4534) **10** Summit Lake (4569) **11** Lower Giant’s Mirror Pond (4541) **12** Blakiston Roadside Pond (4532) **13** Lost Lake (4568) **14** Sofa Wetland B (4542). Photos credit: Parks Canada.

In the spring and summer of 2009, 41 samples of benthic diatoms were collected from waters in Waterton Lakes National Park, the Canadian component of the Waterton-Glacier International Peace Park (Table 1, Figs 2–14). The samples were collected in the course of scheduled pond and stream monitoring and assessment projects. All samples consisted of some surface water and scraping of a submerged object. One objective of benthic diatom sampling was to determine the presence and extent in the Park of *Didymosphenia geminata* (Lyngbye) M. Schmidt (“Didymo” or “rock snot”), which at the time was reported in large numbers from many streams in adjacent Glacier National Park (Bahls 2007, Schweiger et al. 2011). Didymo was detected in 14 samples collected from Waterton Lakes National Park in 2009; all of the Waterton samples that contained Didymo were from flowing waters, including the Waterton River, Belly River and several smaller streams.

**Table 1.** Samples collected from Waterton Lakes National Park in 2009. MDC = Montana Diatom Collection; MONTU = University of Montana Herbarium; NA = data not available.

Sample Numbers		Water Body Name	Latitude (°N)	Longitude (°W)	Slide Numbers		Water Quality Variables		
MDC	Parks Canada				MDC	MONTU	T (°C)	pH	Conductivity (µS/cm)
4520		Cameron Lake at border	49.0000, -114.0578		123-59	39-88	NA	NA	NA
4531	A-13	Stable Pond	49.0683, -113.8900		123-89	40-14	13.0, 9.41		351
4532	A-10	Blakiston Roadside Pond	49.1069, -113.9811		123-90	40-15	11.2, 8.25		203
4533	A-8	Blakiston Beaver Pond A	49.0928, -113.8864		123-91	40-16	11.0, 10.14		389
4534	A-9	Blakiston Beaver Pond B	49.0961, -113.8925		123-92	40-17	10.2, 8.71		530
4535	A-1	Linnet Lake	49.0614, -113.9047		123-93	40-18	10.8, 9.50		242
4536	A-3	Maskinonge Picnic Area Ponds	49.1114, -113.8397		123-94	40-19	11.4, 8.84		560
4537	A-5A	Waterton River Pond A	49.1319, -113.8300		123-95	40-20	11.5, 7.05		234
4538	A-5B	Waterton River Pond B	49.1308, -113.8314		123-96	40-21	10.7, 7.13		364
4539	A-2	Lonesome Lake	49.0736, -113.8931		123-97	40-22	19.6, 8.81		320
4540	A-14A	Sofa Wetland A	49.0656, -113.7450		123-98	40-23	13.7, 9.06		306
4541	A-16	Lower Giant’s Mirror Pond	49.0522, -113.6861		123-99	40-24	12.8, 9.23		279
4542	A-14B	Sofa Wetland B	49.0672, -113.7625		123-100	40-25	13.0, 9.08		238
4543	A-6	Indian Springs Pond	49.1297, -113.8731		124-1	40-26	11.3, 11.12		302
4544	A-7	Buffalo Springs Pond	49.1253, -113.8531		124-2	40-27	10.2, 8.08		344
4545		Waterton River	49.1089, -113.8503		124-3	40-28	NA	NA	NA
4546	WLN-09-01	Cameron Creek	49.0453, -113.9133		124-4	40-29	9.6, 7.94		171
4547	WLN-09-02	Belly River	49.0475, -113.6889		124-5	40-30	13.3, 8.00		183
4548	WLN-09-05	Cameron Creek	49.0786, -113.9669		124-6	40-31	10.1, 8.08		153
4549	WLN-09-07	Lost Horse Creek	49.1211, -113.9983		124-7	40-32	12.1, 7.95		241
4550	WLN-09-08	Coppermine Creek	49.1047, -113.9603		124-8	40-33	13.4, 7.88		247
4551	WLN-09-09	Hell Roaring Creek	49.0219, -113.8989		124-9	40-34	9.3, 8.36		169
4552	WLN-09-10	Boundary Creek	48.9961, -113.9047		124-10	40-35	9.3, 8.17		141
4553	WLN-09-11	Blakiston Creek	49.0739, -113.8689		124-11	40-36	11.9, 8.82		222
4554	WLN-09-12	Belly River tributary	49.0300, -113.6792		124-12	40-37	18.2, 8.62		395
4555	WLN-09-15	Bertha Creek	49.0344, -113.9253		124-13	40-38	7.7, 9.02		108
4556	WLN-09-16	Bertha Creek	49.0325, -113.9125		124-14	40-39	8.9, 8.90		130
4557	WLN-09-13	Crooked Creek	49.0647, -113.7564		124-15	40-40	11.2, 8.61		322
4558	WLN-09-14	Blakiston Creek	49.1058, -113.9814		124-16	40-41	6.7, 8.33		214
4559	WLN-09-17	Crooked Creek	49.1167, -113.8294		124-17	40-42	15.2, 8.50		392
4560	WLN-09-18	Sofa Creek	49.0775, -113.8386		124-18	40-43	6.0, 9.28		289
4561	A-11	Akamina Pools	49.0314, -114.0428		124-19	40-44	14.3, 8.25		92
4562	A-17	Cameron Lake Pools	49.0200, -114.0469		124-20	40-45	10.6, 8.17		449
4563	WLN-09-03	Rowe Creek	49.0575, -114.0122		124-21	40-46	9.5, 7.68		153
4564	WLN-09-04	Lineham Creek	49.0647, -114.0022		124-22	40-47	10.6, 8.19		165
4565	WLN-09-06	Bauerman Creek	49.1311, -114.0308		124-23	40-48	12.4, 8.01		192
4566	WLN-09-19	Bauerman Creek	49.1389, -114.0417		124-24	40-49	8.0, 8.77		201
4567	WLN-09-20	Blakiston Creek	49.1133, -114.0711		124-25	40-50	5.3, 9.55		205
4568		Lost Lake	49.1472, -114.1461		124-26	40-51	15.7, 8.58		74
4569		Summit Lake	49.0078, -114.0258		124-27	40-52	18.3, 8.45		8
4570		Sofa Mountain Ponds	49.0333, -113.7536		124-28	40-53	15.9, 7.84		306

### Haida Gwaii Archipelago (Queen Charlotte Islands) (Figs 15–28, Appendix 1: Plates 18–37)

The Haida Gwaii Archipelago lies about 500 km northwest of Vancouver Island and is separated from the British Columbia mainland by the 70 to 100 km-wide Hecate Strait (Fig. 1). The Archipelago consists of a 300 km-long, north-south trending group of islands in the shape of a “V”. Along the western branch of the “V” is a mountain range with summits over 1,100 m elevation. Higher elevations on the archipelago support mountain hemlock and alpine tundra vegetation; lower elevations are dominated by coastal red cedar, pine, western hemlock and Sitka spruce (Banner et al. 1989).

The coastline along the eastern edge of Haida Gwaii has fluctuated dramatically since 12,000 years BP (Josenhans et al. 1995, 1997). At that time, sea level was about 150 m lower than it is today. By 9,000 years BP, the sea level had risen sharply to 15 m higher than today and remained at that level until about 5,000 years BP, falling to current levels by about 2,000 years BP. These fluctuations are the result of interplay between isostatic, eustatic and tectonic forces in the area. As a result of these fluctuations, Holocene archaeological sites (9,000–5,000 years BP) are stranded in the forest well above their original coastal locations. Diatom remains in sediments of coastal fresh-water ponds include evidence of past salt-water intrusions (Pienitz et al. 2003).

Six samples were collected from freshwater habitats in July 2013 and another three samples were collected from fresh and brackish waters in May 2017 (Table 2). The three samples collected in 2017 were originally numbered 1, 2 and 3 but are re-numbered 7, 8 and 9 here to avoid confusion with the 2013 samples. Sites sampled in 2013 were accessed from the coast by kayak; sites sampled in 2017 were accessed by inland routes. The following descriptions of the sample sites are taken from the field notes of Beverly Boynton.

**Table 2.** Samples collected from Haida Gwaii Archipelago in 2013 and 2017. BB = Beverly Boynton; MDC = Montana Diatom Collection; MONTU = University of Montana Herbarium.

Sample Number		Habitat Type	Latitude (°N)	Longitude (°W)	Slide Numbers	
MDC	BB				MDC	MONTU
5062	1	stream pool	52.2903, -131.2133		127-55	42-61
5063	2	stream pool	52.3522, -131.4108		127-56	42-62
5064	3	small lake	52.3422, -131.4361		127-57	42-63
5065	4	large lake	52.5519, -131.6403		127-58	42-64
5066	5	stream pool	52.5792, -131.7164		127-59	42-65
5067	6	stream pool	52.7075, -131.7172		127-60	42-66
6888	7	bog	53.9272, -132.1070		136-27	49-62
6889	8	bog	52.9108, -131.6145		136-28	49-63
6890	9	river mouth	53.4090, -132.5304		136-29	49-64

**Fresh-water samples, July 2013**

All samples included squeezed vegetation, a scraped rock or stick, surface water and a few ml of iodine added. Samples were taken well above any tidal influence. Sites would all have infrequent human visitation (maybe less than yearly, or even never), especially since I (BB) walked upstream further than needed if people were getting drinking water.

**Figures 15–21.**
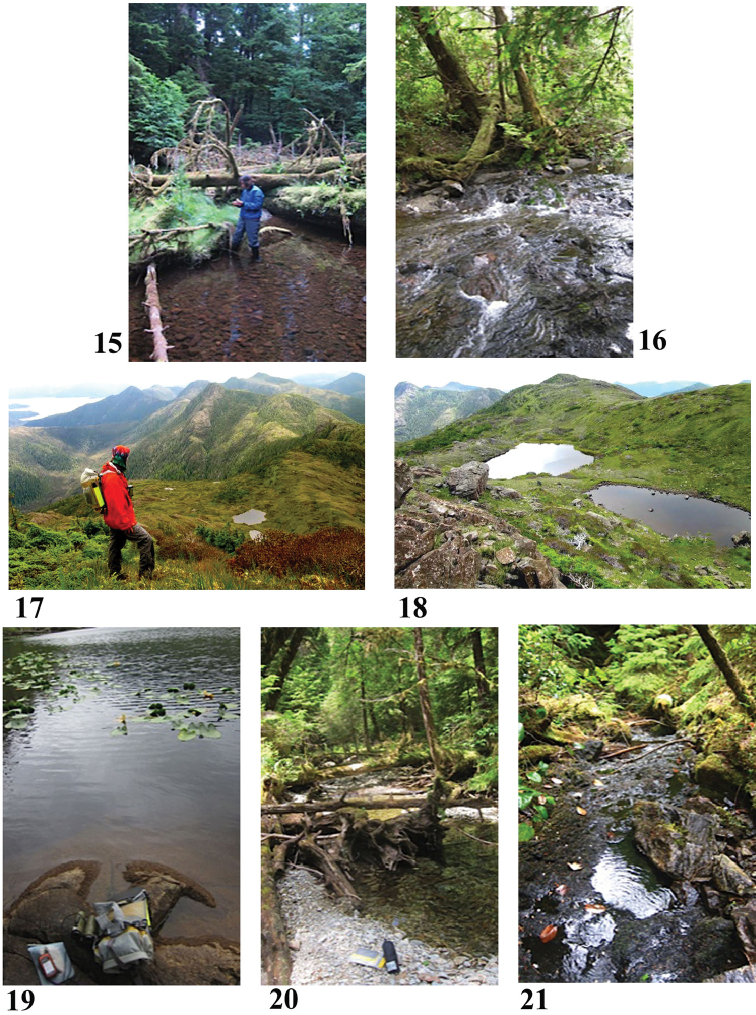
Haida Gwaii collection sites in 2013 **15** Site 1 (5062) **16** Site 2 (5063) **17, 18** Site 3 (5064) **19** Site 4 (5065) **20** Site 5 (5066) **21** Site 6 (5067). Photo credits: Beverly Boynton, D. Moore (**15**), Hope Sneller Moore (**17**).

Sample #1, Harriet Harbour stream pool (Fig. 15) July 11, 2013

52°17.422'N, 131°12.799'W Elevation near sea level

Flowing stream, sample from a pool. Mature western hemlock forest with some western red cedar and red alder, plus salal, grasses, mosses. The abandoned Jedway mine is to the west and north, but this stream seems to be outside their operations. (Jedway was an iron-copper mine, last operating in 1969.) Rock scrapings, plus vegetation squeeze, surface water.

Sample #2, Island Bay stream pool (Fig. 16) July 13, 2013

52°21.130'N, 131°24.643'W Elevation 20 m

Clear water, pools interspersed with small fast-flowing rocky cascades. Western hemlock, western red cedar, salal, mosses, ferns. Vegetation squeeze was of short black and green mosses, scraping was of a submerged stick with filamentous green vegetation (algae?) and surface slime.

Sample #3, lake on Mt. Yatza (front cover, Figs 17, 18) July 14, 2013

52°20.533'N, 131°26.172'W Elevation 550 m

Vegetation squeeze consisted of roots of submerged sedge and black moss; scraping from a submerged rock and surface water. Many sundews on shore, along with grasses, sedges.

Sample #4, lake on Juan Perez Sound/De La Beche Inlet (Fig. 19) July 20, 2013

52°33.121'N, 131°38.422'W Elevation 10 m

Site is 170 m from sea by GPS straight line, beyond the outlet choked with deadfall and yellow pond lilies (no ducks, but other birds seen on the lake, many dragonflies). Clear water, rocky (granite?), moss and sediment on bottom, some submerged grass-like plants. Forested around lake with western hemlock, western red cedar, no Sitka spruce or shore pines. Vegetation squeeze of dirty moss, rock scrape, surface water. Probably very few have been here, with good reason as even though it was a short distance, it was a vicious bushwhack.

Sample #5, stream pool on west end of Kostan Inlet (Fig. 20) July 20, 2013

52°34.752'N, 131°42.999'W Elevation 11 m

A pool just below a riffle. Open ground with grasses, in forest of western hemlock, Sitka spruce, western red cedar, red alder. Rocky shore and stream bottom. Hard to squeeze water from the brown moss on some rocks, no submerged plants or other vegetation. Squeezed what I could, added some water from near bottom, scraped rock with brown moss and slime, surface water.

Sample #6, stream pool east of Lyell Point (Fig. 21) July 22, 2013

52°42.456'N, 131°43.033'W Elevation 116 m

Stream scant but brisk flow into a small pool. Clear water, brown moss and rocks. Forest of red alder, western hemlock, western red cedar, salal, mosses, ferns. Squeezed brown moss (which was longer and easier to squeeze than previous samples), scraped a rock, surface water. Probably no one has been to this particular place.

**Fresh- and brackish-water samples, May 2017**

All samples included squeezed vegetation, a scraped rock or stick, surface water and a few ml of iodine added. According to locals, May was more rainy and cooler than usual.

**Figures 22–28.**
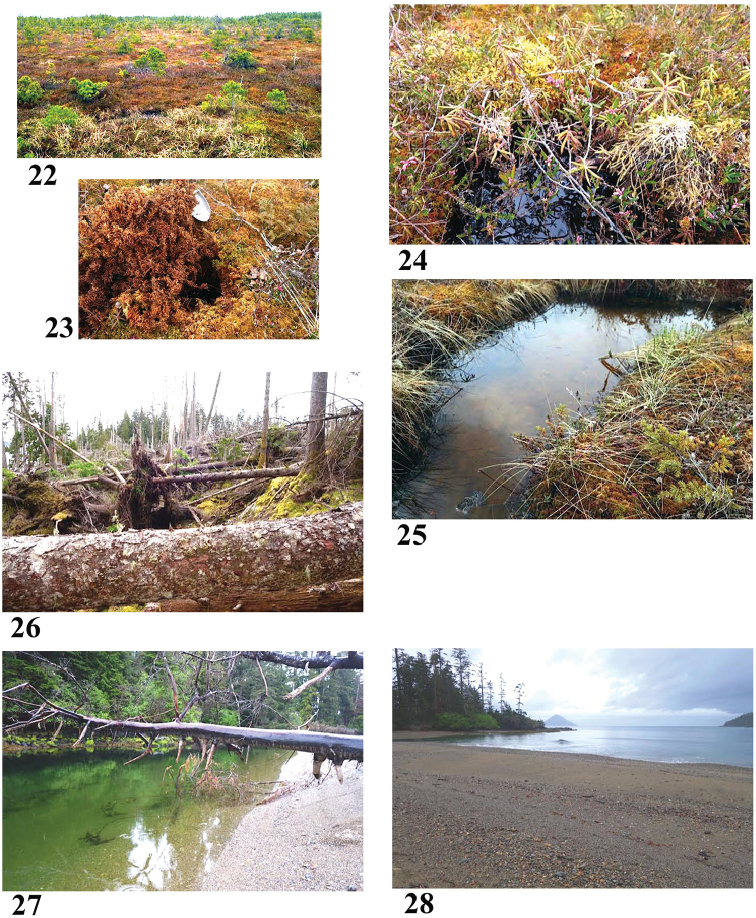
Haida Gwaii collection sites in 2017 **22–25** Site 7 (6888) **26** Site 8 (6889) **27** Site 9 (6890) **28** Rennell Sound from sample site 9. Photos credit: Beverly Boynton.

Sample #7, roadside bog off Route BC-16 W (Figs 22–25) May 9, 2017

53°55.629'N, 132°06.419'W Elevation 48 m pH 4.36, T 10.6 °C

Large bog area, mostly dry, with scattered shore pine, tiny western red cedar with yellowish needles, scattered small common juniper and black crowberry, grasses, bog rosemary, Labrador tea, bog cranberry, *Sphagnum* and other mosses, lichen. Deep layer of peat (dug 0.3 m down with more peat below). Sample from a small area of standing water ~2 m square. Sunny, except where grassy edges of water block the sun. This bog is in the Queen Charlotte Lowlands, on northeast Graham Island, an area quite unlike the topography of the rest of Haida Gwaii, which has a central forested plateau area and mountain ranges. This lowland area is part of the Hecate Depression and includes the Argonaut Plain.

Sample #8, East Limestone Island forest bog (Fig. 26) May 14, 2017

52°54.645'N, 131°36.868'W Elevation 33 m pH 7.6, T 10.8 °C

East Limestone Island is a small limestone island off the east coast of Haida Gwaii. The sample is from a bog with standing water, at the base of a huge Sitka spruce uprooted during a blow down in 2010. The site seems too far from ocean to get sea spray or surge tides. Mosses, grasses, ferns. Sunny when sun in the east, then shady. Thimbleberry, a few other forbs not yet budding.

Sample #9, mouth of river entering Rennell Sound (Fig. 27) May 20, 2017

53°24.543'N, 132°31.821'W Elevation 0 m pH 7.40, T 12.2 °C

Brackish (?) sample. River mouth is directly facing Rennell Sound, tide was rising (still had 1–2 hours to go), wind was blowing into the Sound from Pacific Ocean. Rainy and foggy as often is the case on west coast Haida Gwaii. There was a faint current in middle of the river, slack/eddy on edges where sample was taken (about 15 m from the Sound itself). Sand and gravel bottom and on surrounding land, with a submerged dead western red cedar at sample site providing the scrape; a few clumps of green algae floating around provided the vegetation squeeze.

### Clearwater River Corridor (Figs 29–41, Appendix 1: Plates 38–55)

The following account is taken from the field notes of Beverly Boynton:

Sampling was done in June 2014 while on a 20-day canoe trip from the headwaters of the Clearwater River in Saskatchewan (Forrest Lake) to its confluence with the Athabasca River in Alberta (Fig. 1), estimated to be about 480 river kilometres. Moser et al. (2004) reported on the ecology of diatoms in lakes of Wood Buffalo National Park, which is located about 200 km northwest of the Clearwater/Athabasca River confluence.

The first six samples are from the boreal forest of the Canadian Shield. This area is largely an open-canopy jack pine, black spruce and white spruce forest, with extensive areas of lichens. There are also numerous shrubs, including willows, alders, birch and some forbs. The soil is shallow, i.e. often just an inch (2.5 cm) of forest detritus, on top of deep sand. The area has almost no rocks of any size that we saw (except in rapids) until we were almost off the Shield. The Shield itself is Precambrian granitic rock.

The last three samples are from the Western Canada Sedimentary Basin (depositions from inland seas advancing and retreating). This area has numerous outcrops and gorges of limestone and dolomite. The soil is much deeper, organic soil with clay, supporting a mixed forest of closed-canopy paper birch, white spruce, balsam poplar and a great many shrubs and forbs.

Both areas are topographically fairly flat (especially on the Shield), with an enormous number of shallow gouges from the Laurentide Ice Sheet, which are filled with water. One does not need to walk far to encounter a bog, fen, marsh, swamp, pond, lake or creek. Drainage is generally poor because of the flatness. The hydrology is still quite young and sorting itself out and still influenced by post-glacial rebound.

No samples came from the Clearwater River itself or from areas that seemed to be receiving flow from the River. Because of the low number of people on most sections of the river, along with most people’s general distaste for spending time in bogs, fens, marshes and swamps, the specific sampling sites are probably rarely if ever visited and did not seem to be disturbed. Mosquitoes and blackflies were moderately bad in general on this trip, but were not worse at the sampling sites and posed little problem for the Slime Crew (my husband assisted with some collecting). As always, seeking out these microhabitats added immensely to the interest of the trip; botany and geology were highlights.

All samples consisted of some surface water, a vegetation squeeze and scraping of a submerged object. There were no rocks to scrape and the sticks (when found) had only a minimal slimy feel. If sediment was present on the surface, it was sampled.

Coordinates are WGS84, latitude-longitude in degrees and decimal minutes, elevation in metres. Unfortunately, my camera drowned after the first week; pictures are then from my Itouch; however, the last nine days were quite overcast and I barely was able to keep my Itouch recharged from my Solio. All samples are from the watershed of the Clearwater River.

**Figures 29–35.**
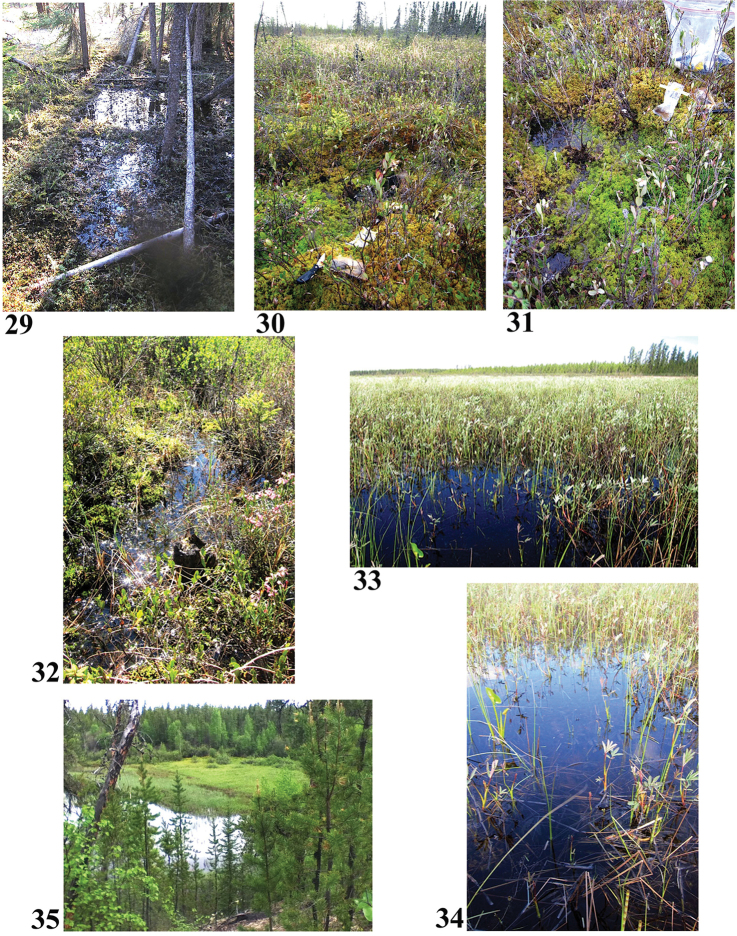
Collection sites along the Clearwater River corridor **29** Site 1 (6273) **30, 31** Site 2 (6274) **32** Site 3 (6275) **33, 34** Site 4 (6276) **35** Site 5 (6277). Photos credit: B. Boynton.

Sample #1, bog near Naomi Lake (Fig. 29) June 8, 2014

**Table 3.** Samples collected from the Clearwater River corridor, June 2014. BB = Beverly Boynton; MDC = Montana Diatom Collection; MONTU = University of Montana Herbarium.

Sample Number		Habitat Type	Latitude (°N)	Longitude (°W)	Slide Numbers	
MDC	BB				MDC	MONTU
6273	1	bog	57.6397, -109.0350		131-34	46-40
6274	2	*Sphagnum* bog	57.5892, -108.8581		131-35	46-41
6275	3	*Sphagnum* bog	57.1761, -108.6394		131-36	46-42
6276	4	fen	57.0922, -108.3267		131-37	46-43
6277	5	wet grassy meadow	57.0050, -108.4447		131-38	46-44
6278	6	swamp	56.9292, -108.6589		131-39	46-45
6279	7	small stream	56.7722, -109.2839		131-40	46-46
6280	8	wet meadow	56.6978, -109.9767		131-41	46-47
6281	9	shady pool	56.6539, -110.9553		131-42	46-48

57°38.383'N, 109°02.102'W Elevation 498 m pH 6.2, T not taken

Looks like I forgot the iodine on this; I added some on July 1. Area of small bogs within a larger area of semi-open jack pine, reindeer moss and Labrador tea. In the bog: mountain cranberry, Labrador tea, lichens, mosses.

Sample #2, bog near outlet of Dell Lake (Figs 30, 31) June 9, 2014

57°35.342'N, 108°51.487'W Elevation 480 m pH 4.6, T not taken

Clear, sunny, no obvious flow. *Sphagnum* blanket with a lot of leatherleaf, some larch, small balsam fir, Labrador tea, cottongrass, cloudberry.

Sample #3, bog between Lloyd Lake and First Gorge (Fig. 32) June 13, 2014

57°10.563'N, 108°38.360'W Elevation 470 m pH 4.96, T not taken

*Sphagnum* moss, sunny, clear, no obvious flow. A few small jack pine and black spruce, a lot of bog birch, sedges, bog rosemary and bog laurel. Elk scat in the water.

Sample #4, fen adjacent to a lake (Figs 33, 34) June 14, 2014

57°05.537'N, 108°19.593'W Elevation 453 m no pH or T taken

Looks like I forgot the iodine on this; I added some on July 1. Extensive area of standing water adjacent to a lake. Moss, *Sphagnum*, bog rosemary, bog birch, leather leaf, jack pine, river birch, a few sedges, Labrador tea. Sediment on surface.

**Figures 36–41.**
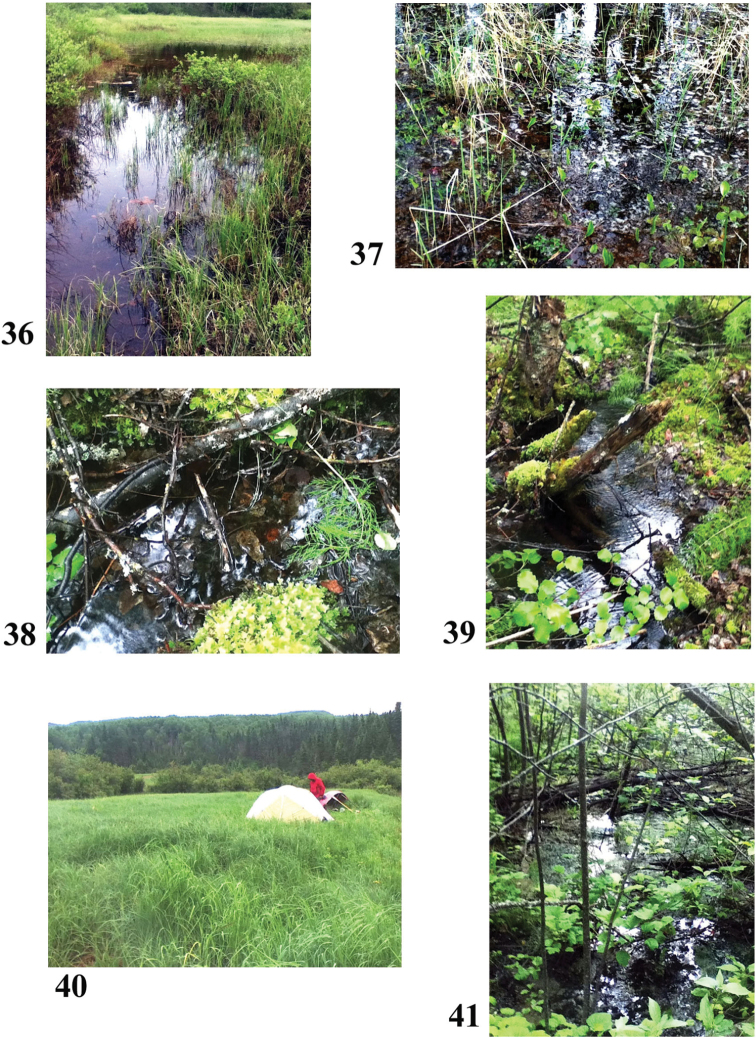
Collection sites along the Clearwater River corridor **36** Site 5 (6277) **37** Site 6 (6278) **38, 39** Site 7 (6279) **40** Site 8 (6280) **41** Site 9 (6281). Photos credit: Beverly Boynton.

Sample #5, wet grassy meadow near Granite Gorge (Figs 35, 36) June 16, 2014

57°00.298'N, 108°26.680'W Elevation 439 m pH 6.89, T 16.2 °C

The Clearwater runs through Granite Gorge with big rapids, then a fairly large arm doubles back to end at edge of sample site meadow. Samples are at an elevation such that it probably does not get flooded from the river (this was a high water year while we were there). The grass is thick with wet muck and some standing water. My sample is near base of a steep-sloped bench of jack pines and paper birch, with a few small balsam poplars, plus lichens, mosses, cranberries, kinnikinnick etc. One picture shows a small flow of water coming off the bench to sample site. Sample site has marsh cinquefoils, bog birch, grasses, willows, dwarf raspberry. Sample taken from a small area of open water with no obvious flow, sunny, clear. Some specks of sediment floating on top, plus 10 × 10 cm blobs of red-brown stuff floating. Some (natural?) oil seeps here. Bottom is muck and debris. [The Alberta tar sands are about 100 km northwest of here.]

Sample #6, swamp above Olsen Rapid (Fig. 37) June 17, 2014

56°55.749'N, 108°39.526'W Elevation 437 m pH 5.22, T 13.9 °C Swamp with mature jack pine, alive in water. A few grasses. Water clear, no obvious flow, surface has clusters of bubbles (amphibian eggs?). Bottom mucky with organic detritus. Swamp is within a mature jack pine forest with the usual lichens, cranberries, dwarf blueberries, many wild lily of the valley and bunchberries. Some fireweed, mosses, Saskatoon (service berry).

Sample #7, small stream (Figs 38, 39) June 20, 2014

56°46.338'N, 109°17.041'W Elevation 371 m pH 7.7, T 21.5 °C

Small semi-shaded, slowly-flowing stream, clear water, draining towards Clearwater. Birch, willow and alder forest, with mosses, currant, strawberry, horsetails, *Mertensia*. Bottom was leafy detritus, muck. Sample was from a still pool. Still on the Shield, pink granitic rock with frequent outcrops.

Sample #8, wet meadow above bend in Clearwater (Fig. 40) June 22, 2014

56°41.860'N, 109°58.600'W Elevation 304 m pH 7.52, T 18.7 °C

Depression in meadow with standing water, no visible flow, clear, sunny. Willows, cattails, grasses, plumed false Solomon seal, patches of mosses in water along with detritus on bottom. This was the only such extensive wet meadow we saw on the trip and it was in the Western Sedimentation Basin, with the river now in a wide valley with wooded ridges on either side of the valley. No outcrops or boulders and the sandy benches were quite infrequent. Shrubs and trees were bigger and more diverse, with soil much deeper (black, a lot of organic material before reaching clay-sand). Grass was 1–1.5 m tall, thick, meadow rue. Many pools of water in the entire area.

Sample #9, pool near Greentree Provincial Campground (Fig. 41) June 24, 2014

56°39.233'N, 110°57.320'W Elevation 256 m pH 7.52, T 12.7 °C

In woods (birch, balsam poplar, alder, balsam fir), standing water, no obvious flow, shady. Immediate area around this was pretty flat. Dwarf raspberry, currants, grasses, horsetails. Brown and green algae blobs on surface, bottom has muck and detritus.

### Coppermine River Corridor (Figs 42–57, Appendix 1: Plates 56–76)

The following account is taken from the field notes of Beverly Boynton:

Sampling was done while on a 28-day canoe trip on the Coppermine River (Fig. 1), from Point Lake near the headwaters, to its mouth in Coronation Gulf of the Arctic Ocean, about 450 river kilometres. All samples were from fresh water, none closer to the Arctic Ocean than about 10 km; no samples were taken from the Coppermine River itself. Only sample #1 was from Northwest Territories; the rest are from Nunavut, Canada.

The trip began in the upper Coppermine, which is a system of lakes on Arctic tundra, above the treeline. At Redrock Lake, the Coppermine leaves the tundra to enter white spruce forest with willow and birch shrubs. Treeline and its transition zone follow the protected river valley up to the Coppermine Mountains, though tundra predominates in places beyond the river valley. At Big Bend, the trees thin and become shorter. Past the Coppermine Mountains, the terrain is predominantly tundra vegetation of grasses and sedges, lichens, willows and smaller birch shrubs.

The entire trip was on the Canadian Shield, though this Precambrian granitic rock was not always visible due to postglacial till and sediment deposition. A huge glacial lake, Lake Coppermine, formed during deglaciation when a lobe of the ice sheet blocked the Coppermine’s outlet to the sea. This lake extended from Fairy River to Rocky Defile and has left behind lake sediments of marl. Further downriver, the Coppermine cuts through sandstone, limestone and dolomite, forming gorges. The river then goes through the Muskox Intrusion, which was formed during the Proterozoic from mantle plumes. This is one of the globe’s largest basalt flood plains and consists of rock types such as gabbro, which is the rock type from Escape Rapids to Coronation Gulf (Dredge 2001).

Place names were non-existent, so descriptive terms are used here.

**Figures 42–49.**
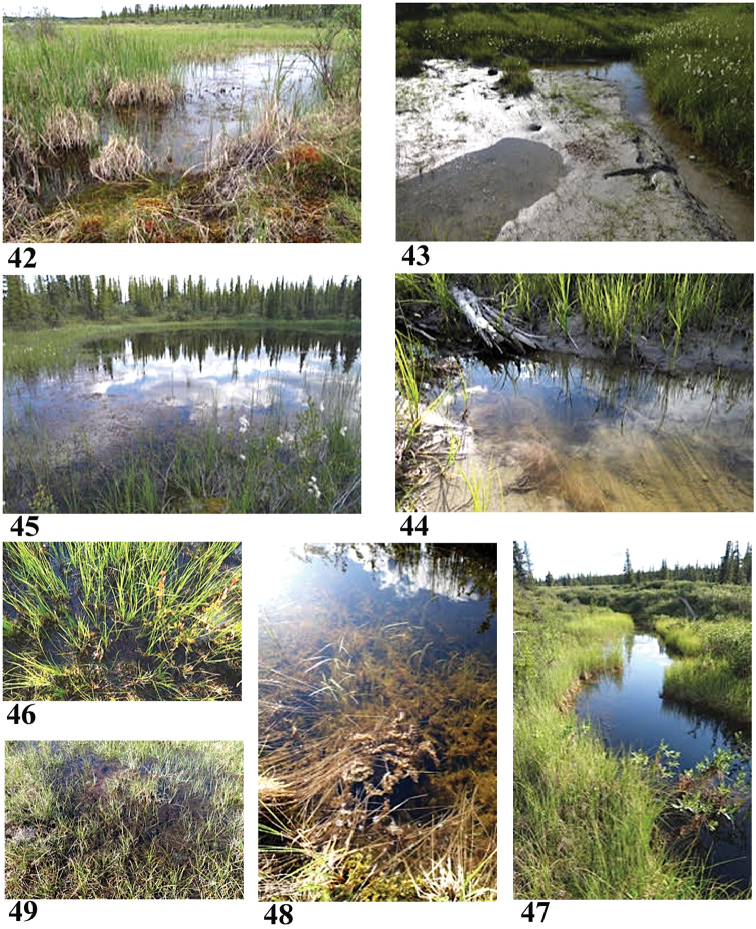
Collection sites along the Coppermine River corridor **42** Site 1 (6824) **43, 44** Site 2 (6825) **45, 46** Site 3 (6826) **47, 48** Site 4 (6827) **49** Site 5 (6828). Photos credit: Beverly Boynton.

Sample #1, grassy meadow at Wolf Camp (Fig. 42) July 20, 2015

**Table 4.** Samples collected from the Coppermine River corridor, July/August 2015. BB = Beverly Boynton; MDC = Montana Diatom Collection; MONTU = University of Montana Herbarium.

Sample Numbers		Habitat Type	Latitude (°N)	Longitude (°W)	Slide Numbers	
MDC	BB				MDC	MONTU
6824	1	lake	65.8268, -114.3896		135-62	48-97
6825	2	stream	66.3652, -114.4949		135-63	48-98
6826	3	lake	66.7441, -115.3878		135-64	48-99
6827	4	stream	66.8800, -116.3331		135-65	48-100
6828	5	pool	67.2461, -116.3628		135-66	49-1
6829	6	lake	67.2528, -116.3602		135-67	49-2
6830	7	lake	67.1936, -115.7955		135-68	49-3
6831	8	lake	67.3350, -115.7965		135-69	49-4
6832	9	lake	67.6181, -115.4367		135-70	49-5
6833	10	lake	67.7657, -115.3817		135-71	49-6

65.8268°N, 114.3896°W Elevation 350 m pH 8.18, T 12 °C

Standing water, about 10 m × 5 m, in a large, sunny grassy meadow with a few willows. About 100 m from river. No visible surface inlet/outlet. Beyond the meadow, there is a small ridge with granitic bedrock outcrops. Water is clear, with bottom of *Sphagnum* moss. Surrounded by red and green moss, grasses, no forbs. Sample: surface water, *Sphagnum* squeeze, scraped slimy stick.

Sample #2, Coppermine tributary at marl bluff (Figs 43, 44) July 24, 2015

66.3652°N, 114.4949°W Elevation 281 m pH 8.38, T 19.2 °C

Small, sluggish tributary of Coppermine River, about 105 m above the confluence. Sunny area with grey clay on bottom and on stream bank (strong reaction to HCl). Some cotton grass, grasses, no forbs; further away are white spruce, birch shrubs, buffalo berry, marl bluffs. Water is clear, with a small fish. Sample: surface water, superficial bottom sediment, squeezed grass roots, scraped branch and twig.

Sample #3, small lake at Orchid Camp (Figs 45, 46) July 26, 2015

66.7441°N, 115.3878°W Elevation 282 m pH 8.6, T 22.4 °C

Multiple little lakes about 113 m from Coppermine. Sunny, maybe 1 m deep, *Sphagnum* moss, surrounded by cotton grass, mosses, birch shrubs, white spruce. In a low area, no obvious surface inlet/outlet. Twinflowers, squirrel egg yellow orchid and yellow lady’s slipper orchid nearby.

Sample #4, small stream at Bear Skull Camp (Figs 47, 48) July 28, 2015

66.8800°N, 116.3331°W Elevation 288 m pH 7.5, T 17.4 °C

Small meandering stream of still water in large, sunny, hummocky meadow between two long sandy eskers. Maybe 0.6 m deep, *Sphagnum* and aquatic grasses on bottom. Sample: surface water, grass root squeeze, scraped a branch.

Sample #5, tundra pool (Fig. 49) July 30, 2015

67.2461°N, 116.3628°W Elevation 489 m pH 8.1, T 17.2 °C

Sunny area of hummocks with pools of standing water, surrounded by sedges, grasses, no forbs, rocky. Maybe 0.3 m deep, muddy bottom with decaying vegetation. Clear water, no surface inlet/outlet noted. Split a rock, good reaction from HCl; green specks on split surface may have been copper ore. About 7 km from Coppermine River.

**Figures 50–57.**
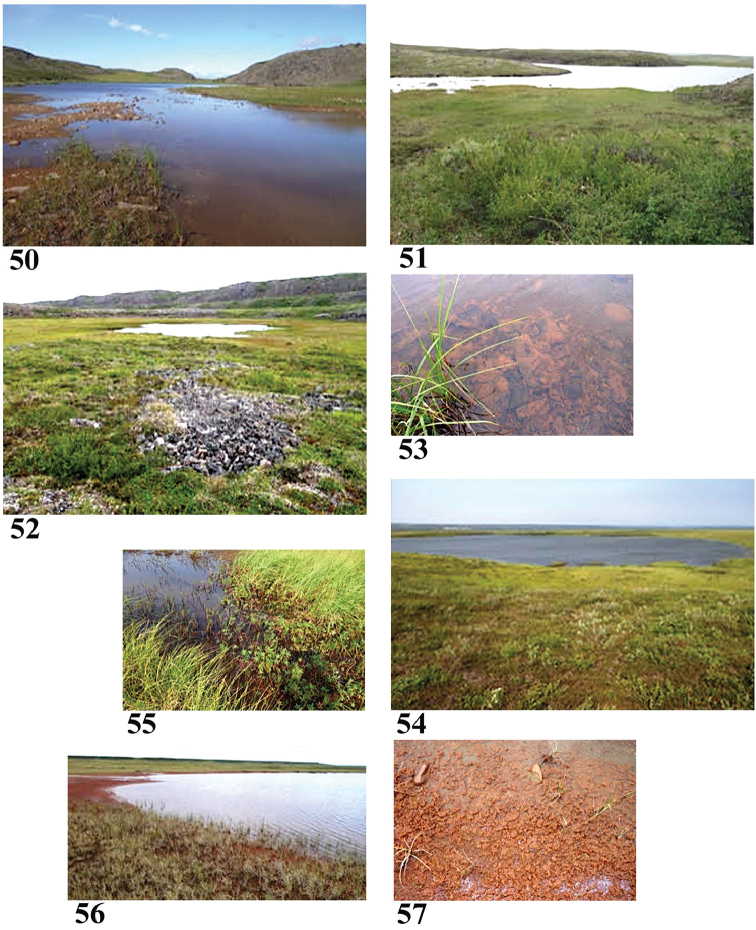
Collection sites along the Coppermine River corridor **50** Site 6 (6829) **51** Site 7 (6830) **52, 53** Site 8 (6831) **54, 55** Site 9 (6832) **56, 57** Site 10 (6833). Note mats of iron-oxidising bacteria in Figs **50, 53, 56, 57**. Photos credit: Beverly Boynton.

Sample #6, Red Sand Lake (Fig. 50) July 30, 2015

67.2528°N, 116.3602°W Elevation 472 m pH 8.0, T 20.5 °C

Lake 6.9 km from Coppermine. Clear water with surface algae, sunny, about 15 cm deep at edge. Surrounded by grasses, sedges, no forbs. Sample: surface water, poor squeeze of hard-to-pull grasses, scraped a slimy rock. Bottom with reddish-brown colour [iron-oxidizing bacteria?].

Sample #7, September Mountains Lake (Fig. 51) August 1, 2015

67.1936°N, 115.7955°W Elevation 440 m pH 8.3, T 17.4 °C

Lake with clear water, sunny. *Sphagnum* moss and mud on bottom, surrounded by aquatic grasses. About 3.7 km from Coppermine. Sample: surface water, grass squeeze, no sticks or accessible stones to scrape.

Sample #8, Coppermine Mountains Lake (Figs 52, 53) August 3, 2015

67.3350°N, 115.7965°W Elevation 435 m pH 8.2, T 14.8 °C

Small lake with clear water, surrounded by aquatic grasses, sedges. Bottom with reddish-brown algae-like growth [iron-oxidising bacteria?]. About 4 km from Coppermine. Sample: surface water, squeezed grass roots, scraped rocks from bottom.

Sample #9, Escape Rapids Lake (Figs 54, 55) August 6, 2015

67.6181°N, 115.4367°W Elevation 145 m pH 6.76, T 18 °C

Moderately large lake in tundra in wet, hummocky area. No definite inlet, outlet. This is now in the area of post-glacial marine sedimentation from sea level changes; fresh water sample may be influenced by marine sediments. Clear water, sunny, aquatic plants, grasses, sedges, willows, marsh cinquefoils. Mud and algae on bottom. Sample is from a quiet backwater on a very windy day. Sample: surface water, plant squeeze, no rocks or sticks to scrape.

Sample #10, Bloody Falls Tundra Lake (Figs 56, 57) August 8, 2015

67.7657°N, 115.3817°W Elevation 72 m pH 8.17, T 11.3 °C One of many small lakes in area of tundra and hummocks. Red sand, rocks, and algae [iron-oxidising bacteria?] on bottom. Clear water, sunny, some insects in water. Grasses, sedges, willows surround the lake. Sample: surface water, grass squeeze, scraped non-slimy rock.

### Baillie & Back River Corridors (Figs 58–72, Appendix 1: Plates 77–108)

The following account is taken from the field notes of Beverly Boynton:

Sampling was done while on a 26-day canoe trip that went from a lake on the Baillie River (a main tributary of the Back River), to the Baillie’s confluence with the Back River, then down the Back River, passing through Pelly Lake and ending on Mission Island in Upper Garry Lake, about 445 river km from our starting point (Fig. 1). Elevation at our put-in on the Baillie was 284 m, about 168 m near the confluence of the Baillie and Back Rivers (a 120 m drop over about 185 km), then the Back essentially becomes flatwater, with an elevation at our take out on Mission Island of 148 m (a drop of only 20 m over the final 260 km). All samples are fresh water from Nunavut.

The sampling area is subarctic tundra on the Canadian Shield, with continuous permafrost and a thin active layer of reportedly acidic soil. There are some areas of granitic outcrops, but most of the river corridor is covered with extensive deposits from de-glaciation of the Laurentide Ice Sheet, including sandy eskers, large sand flats and areas of mostly sorted till and sediments. In addition, there are extensive areas of peat, with sphagnum moss and other mosses. Lichen species were fairly ubiquitous. The area is well above the treeline, except for one small area on the Baillie that has a relic stand of white spruce. The shrubs include dwarf birch, small willow species, red alder and various Ericaceae species. Other plants included grasses, sedges and the expected flora for subarctic bogs, fens and uplands.

Annual precipitation is low, making this area a polar desert, but during summer, snow melt and thawing of the active layer results in waterlogged soil and a network of lakes, streams, rivers and wetlands. Throughout the collection area were numerous ponds, pools of standing water, wet peatlands and wet to moist areas of hummocks and patterned ground, in addition to dry uplands.

From local reports, May and June were rainy months, at least in Yellowknife, about 480 km to the west of our put-in. The Back clearly had high waters, as most dry riverbeds noted on maps were covered with water. The Back drains a huge area and, being a lowlands river on permafrost, the accumulated water is slow to discharge into the Arctic Ocean. We had a number of high wind days that kept us from paddling and a few periods of rain.

Place names are my own descriptive terms, sometimes adding nearby names from Canadian maps.

**Figures 58–63.**
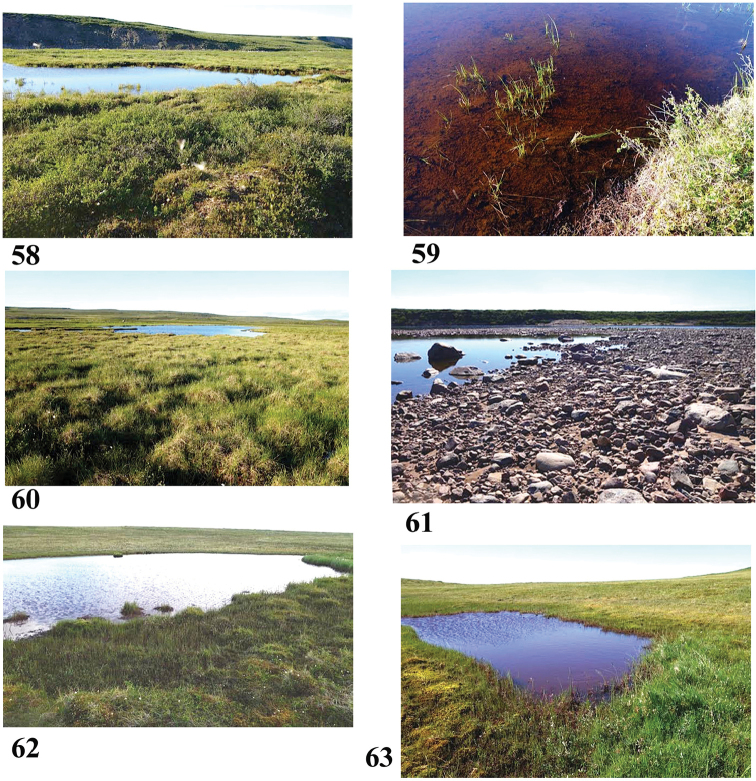
Collection sites along the Baillie and Back River corridors **58, 59** Site 1 (6856) **60** Site 2 (6857) **61** Site 3 (6858) **62** Site 4 (6859) **63** Site 5 (6860). Note microbial mat of red iron-oxidising bacteria in **59** Photos credit: Beverly Boynton.

Sample #1, pond in wetland near the Baillie River (Figs 58, 59) July 4, 2016

**Table 5.** Samples collected from the Baillie and Back River corridors, July 4–July 26, 2016. BB = Beverly Boynton; MDC = Montana Diatom Collection; MONTU = University of Montana Herbarium.

Sample Numbers		Habitat Type	Latitude (°N)	Longitude (°W)	Slide Numbers	
MDC	BB				MDC	MONTU
6856	1	pool in wetland	64.8837, -105.7789		135-94	49-29
6857	2	pond	64.8853, -105.0805		135-95	49-30
6858	3	river backwater	64.9621, -104.5984		135-96	49-31
6859	4	pond	65.9621, -103.5971		135-97	49-32
6860	5	pond	65.3920, -103.3882		135-98	49-33
6861	6	pool	65.6039, -102.6807		135-99	49-34
6862	7	river backwater	65.9111, -101.8610		135-100	49-35
6863	8	wetland	65.9405, -101.4412		136-1	49-36
6864	9	pond	65.8964, -101.0479		136-2	49-37
6865	10	wetland	65.8989, -101.0356		136-3	49-38
6866	11	small stream	65.9063, -100.7711		136-4	49-39

64°53.020'N, 105°46.733'W Elevation 239 m pH 7.9, T 22 °C

A small, shallow, sunny tundra pool, about 10 m by 5 m, in a large wetland with plenty of mosquitoes, situated on a terrace above the Baillie River. No surface inlet or outlet seen. Clear water, red-brown algae and sediments [iron-oxidising bacteria] on bottom. Surrounding vegetation includes dwarf birch, willow, Labrador tea, grass, sedges. Further away from river and pond is a ridge with granitic outcrops. Sample: surface water, grass and algae squeeze, scraped stick.

Sample #2, pond near Merganser Camp off the Baillie River (Fig. 60) July 6, 2016

64°53.118'N, 105°04.829'W Elevation 214 m pH 7.15, T 18.6 °C

Tundra pond in a large, flat, sunny area of moist hummocks with no standing water between them. No surface inlet or outlet seen. Clear water, brown algae and sediments on bottom. Surrounding vegetation includes dwarf birch, willow, Labrador tea, cloudberry, mountain avens, pink and yellow louseworts, bog rosemary, grass, and sedges including cotton grass. Further away from the river and pond is a ridge with granitic outcrops. Sample: surface water, squeezed vegetation and scrape.

Sample #3, backwater on the Baillie (Fig. 61) July 8, 2016

64°57.726'N, 104°35.904'W Elevation 198 m pH 7.1, T 19.4 °C

Still backwater in sunny area on the Baillie River, in an area of flooding. Clear water, river bottom of sand and fine silt, submerged aquatic plants. Sand and boulders on shoreline, but no plants. Sample: surface water, rooted submerged plant squeeze, scraped rock.

Sample #4, pond near Mud Beach Camp off the Back (Fig. 62) July 11, 2016

65°57.726'N, 103°35.828'W Elevation unknown pH 6.4, T 23.9 °C

Tundra pond in sunny area. Surrounding vegetation includes willow, Labrador tea, yellow lousewort, bog rosemary, aquatic grasses, sedges, mosses.

Sample #5, pond near Hill Camp off the Back (Fig. 63) July 12, 2016

65°23.521'N, 103°23.291'W Elevation 189 m pH 6.2, T 22.7 °C

Tundra pond in sunny area, with short grasses, forbs and shrubs further from the pond. Clear water, bottom with red-coloured sediments [probably colonies of iron-oxidising bacteria]. Vegetation surrounding the pond includes dwarf birch, willow, Labrador tea, yellow lousewort, bog rosemary, cloudberry, mosses. Sample taken on windy day from lee end.

**Figures 64–68.**
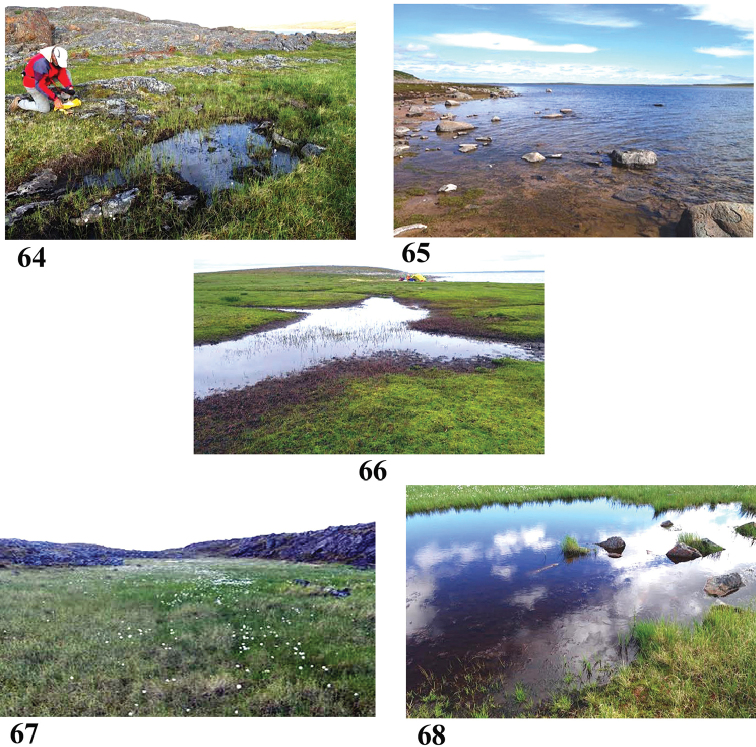
Collection sites along the Baillie and Back River corridors **64** Site 6 (6861) **65** Site 7 (6862) **66** Site 8 (6863) **67, 68** Site 9 (6864). Photos credit: Beverly Boynton.

Sample #6, pool near Inuksuk Camp off the Back (Fig. 64) July 14, 2016

65°36.231'N, 102°40.844'W Elevation 205 m pH 6.44, T 18.7 °C

Small pool of standing water in area of moist hummocks, atop a bedrock granite ridge. Clear water, grasses and mosses in water and on bottom (intermittent standing water). Surrounding vegetation includes dwarf birch, Labrador tea, alpine azalea, cloudberry, sedges, cottongrass, mosses, lichens. Sample: surface water, squeezed aquatic grasses.

Sample #7, eddy on the Back River (Fig. 65) July 19, 2016

65°54.663'N, 101°51.659'W Elevation 156 m pH 7.28, T 13.6 °C

Sunny area in a backwater of the Back River. Clear water, fine silt bottom. Aquatic plants in water. Sample: surface water, squeeze submerged roots/stems, difficult scrape of submerged rock.

Sample #8, wetland near Pelly Monument Camp at Pelly Lake (Fig. 66) July 20, 2016

65°56.431'N, 101°26.474'W Elevation 158 m pH 7.17, T 16.2 °C

Sunny area of patterned ground, filled with clear standing water. Vegetation includes aquatic plants, grasses, mosses, a few forbs, but no shrubs. Much goose scat, feathers, many molting geese ran off upon our arrival. Sample: surface water, squeezed submerged roots.

Sample #9, pond on tundra near Cabin Camp (Figs 67, 68) July 22, 2016

**Figures 69–72.**
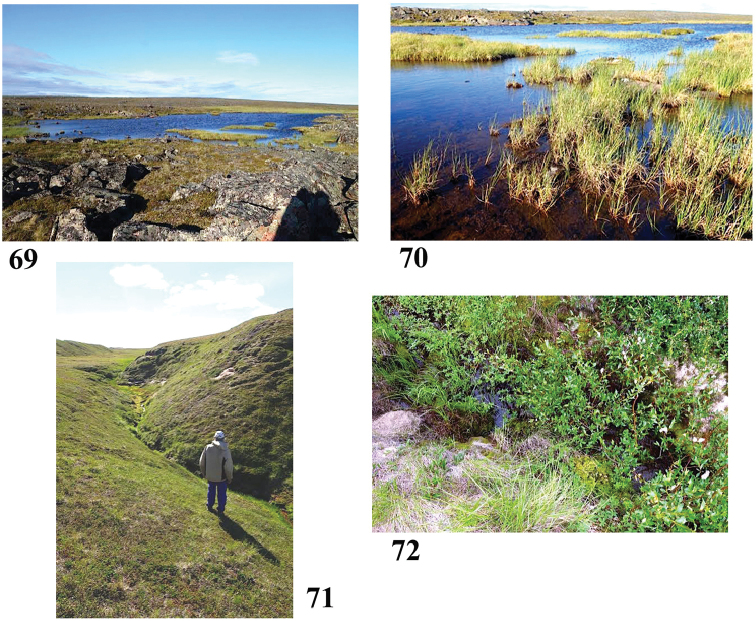
Collection sites along the Baillie and Back River corridors **69, 70** Site 10 (6865) **71, 72** Site 11 (6866). Photos credit: Beverly Boynton.

65°53.784'N, 101°02.873'W Elevation 166 m pH 6.80, T, 15.7 °C

Long tundra pond in a swath of moist hummocks with cottongrass, situated between two granite ridges. Water possibly has channels that connect with Pelly Lake. Sunny, sediments and algae on bottom. Vegetation includes a few dwarf birch, Labrador tea, cottongrass, grass, moss. Many mosquitoes, a pair of red-necked phalaropes and snow buntings flew by. Sample: surface water, plant squeeze, rock scrape.

Sample #10, Pelly Cove wetland (Figs 69, 70) July 24, 2016

65°53.931'N, 101°02.134'W Elevation 179 m pH 6.9, T 11.6 °C

Sunny area of clear water, bottom with sediments and *Sphagnum* moss, algae, surrounded by granite rocks. Vegetation includes Labrador tea, mountain cranberry, bog rosemary, grasses and mosses.

Sample #11, Mission Island rivulet (Figs 71, 72) July 26, 2016

65°54.380'N, 100°46.266'W Elevation 166 m pH 6.6, T 13.5 °C

Sample from pool of clear still water, partly shaded by willows, in small creek flowing down a broad swale between two tundra ridges. Bottom with algae, sediments, mosses. Surrounding vegetation includes willow, *Potentilla*, grass, moss. Sample: surface water, grass squeeze, scraped stick.

### Hood River Corridor (Figs 73–97, Appendix 1: Plates 109–132)

The following account is taken from the field notes of Beverly Boynton:

Sampling was done while on a 27-day, 300-km canoe trip in Nunavut, from the headwaters of the Hood River to the north end of the peninsula dividing the Hood River mouth in Arctic Sound from Baillie Bay (both are in Bathurst Inlet of Coronation Gulf of the Arctic Ocean). Elevation at our put-in is 414 m and the mouth of the Hood is at sea level.

The Hood River is on the Central Continental Arctic portion of North America, on the Precambrian Canadian Shield. It lies between the Contwoyto Plateau to the south (a 450 m high plateau of gently rolling drift) and the Tree River uplands to the north and west (a lower, dissected granite plateau of smooth rock-knob hills with deep valleys) and flows through isolated, rugged tundra. For most of its length, the Hood is less than 150 km south of Coronation Gulf of the Arctic Ocean as it runs west to east. There are many areas of Precambrian granitic outcrops, but much of the surface along the river corridor is covered with extensive deposits from de-glaciation of the Laurentide Ice Sheet, with areas of sorted and unsorted till and sediment, including sandy eskers, sand, mud and clay flats.

The lower half of the Hood has some metamorphic rocks of quartzite and slate, with clay tills. The Wilberforce Hills to the east are the dissected edge of the Contwoyto Plateau. About 50 km from its mouth, the Hood turns abruptly to the north in its run to the coast. The river then lies in a broad flood plain with evidence of previous salt-water incursions from when sea levels were higher.

Bathurst Inlet is a physiographic division of the Shield, with a major NNE to SSW fault forming the boundary between the uplands and the Coronation Gulf Lowlands. It is a complex submerged valley, a 200 km-long extension of the Coronation Gulf lowlands penetrating the Shield, with west-dipping diabase and basalt sills, often overlying basalt. The Queen Maud Lowlands lie to the east of the Inlet.

The river is all above the Arctic Circle, well above the treeline, with continuous permafrost and a thin active layer of soil. Lichen species were ubiquitous as were Ericaceae spp., dwarf birch, willow, alder, sedges and grasses. Mosses seemed less extensive than seen on other barren grounds trips; we identified 50 species of arctic wildflowers.

Unfortunately, strong headwinds prevented us from paddling to the actual estuary of the Hood in Arctic Sound and, even more disappointing, a hike to the tip of the peninsula ended on tall undercut bluffs that prevented a descent to the ocean at the northernmost point. For this reason, the final Hood River sample was about 8 km upstream from Arctic Sound and the first Bathurst Inlet sample (on east side of the peninsula) was perhaps half a mile to the south of the headlands of the peninsula.

The Hood River was very low this season, presumably due to low winter snow, an early snow and ice meltoff and lack of rain. Compared to a personal account by friends who paddled the river in 2013 and found it to be low water, this year the river was much lower.

There is considerable research being done on the arctic freshwater system in the face of climate change. Significant changes include rising surface air temperatures, warming permafrost and shrub encroachment on the northern tundra. Storage and cycling of fresh water on land has changed along with precipitation, river discharge, lake abundance and size and soil moisture.

In total, 16 samples of benthic diatoms were collected from water bodies along the Hood River corridor. (There is no sample #10.) The following field notes describe the sampling sites. Datum is WGS84, coordinates are latitude-longitude in degrees and decimal minutes; elevation is in metres. Place names are my own (BB) descriptive terms, sometimes adding nearby names from Canadian maps.

**Figures 73–78.**
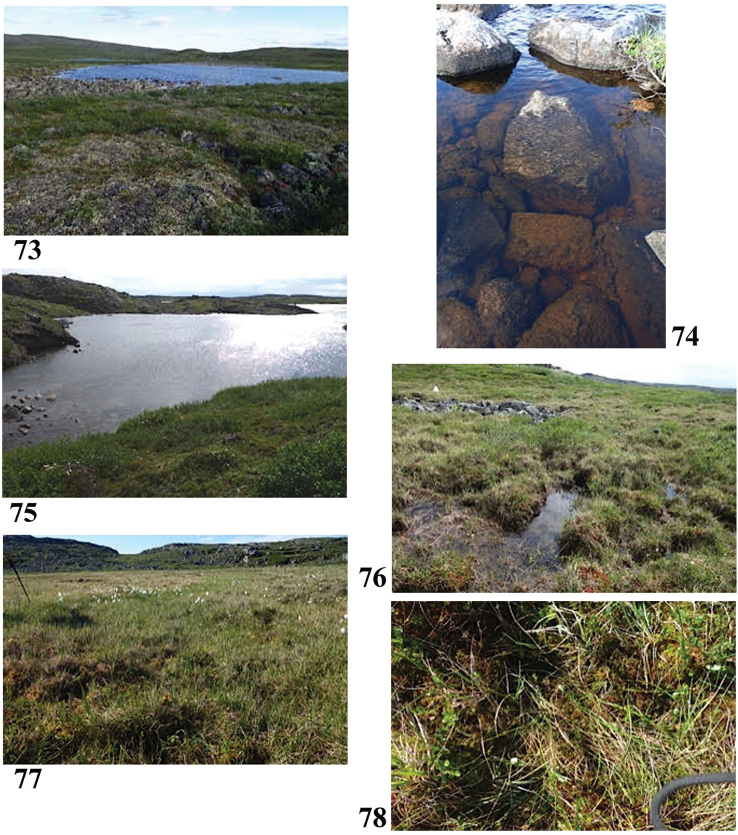
Collection sites along the Hood River corridor **73, 74** Site 1 (6898) **75** Site 2 (6899) **76** Site 3 (6900) **77, 78** Site 4 (6901). Photos credit: Beverly Boynton.

Sample #1, lake near headwaters of the Hood River (Figs 73, 74) July 2, 2017

**Table 6.** Samples collected from the Hood River corridor in 2017. BB = Beverly Boynton; MDC = Montana Diatom Collection; MONTU = University of Montana Herbarium.

Sample Numbers		Habitat Type	Latitude (°N)	Longitude (°W)	Slide Numbers	
MDC	BB				MDC	MONTU
6898	1	lake	66.5752, -112.8793		136-37	49-72
6899	2	lake	66.6155, -112.4682		136-38	49-73
6900	3	stream	66.6155, -112.4682		136-39	49-74
6901	4	hummocks	66.6527, -111.8972		136-40	49-75
6902	5	lake	66.6361, -111.8739		136-41	49-76
6903	6	meadow	66.6328, -111.4347		136-42	49-77
6904	7	river	66.8362, -110.3873		136-43	49-78
6905	8	lake	67.0637, -108.6731		136-44	49-79
6906	9	river	67.1155, -108.8199		136-45	49-80
6907	11	meadow	67.2046, -108.8091		136-46	49-81
6908	12	lake	67.1985, -108.8485		136-47	49-82
6909	13	lake	67.3837, -108.8626		136-48	49-83
6910	14	pool	67.3885, -108.8655		136-50	49-84
6911	15	sandy beach	67.4166, -108.8595		136-51	49-85
6912	16	mud flat	67.4125, -108.8571		136-52	49-86
6913	17	river	67.3745, -108.8864		136-53	49-87

66°34.513'N, 112°52.756'W Elevation 426 m pH 8.18, T 18.2 °C

Lake in dry uplands with no defined inlet/outlet, but surrounded by various low ridges with granite bedrock. Scrape was on rock with leafy black algae; water was clear but with glops of gold/brown floating on surface. Usual dwarf birch, Ericaceae, forbs mosses, lichens.

Sample #2, lake near Windy Point Camp, headwaters of the Hood River (Fig. 75) July 4, 2017

66°36.929'N, 112°28.089'W Elevation 415 m pH 5.72, T 15.9 °C

Lake in dry uplands with granite outcrops and boulders, in a valley between two ridges. Dwarf birch, sedges, bog rosemary, Labrador tea, cottongrass, mountain cranberry, lichens and mosses. Brown sediments and algae on bottom.

Sample #3, flowing stream (Fig. 76) Elevation unknown July 4, 2017

66°36.929'N, 112°28.089'W (coordinates approximate) pH 5.95, T 11.1 °C

Briskly flowing stream with bed of small granite boulders, sunny with some shade from banks. Sample is from a flat area of stream that is maybe an area of springs (the stream flows down a pretty good gradient for the area). Sample is from a quiet side pool. Green mosses, some sedges, tall willows.

Sample #4, moist hummocks (Figs 77, 78) July 8, 2017

66°39.164'N, 111°53.829'W Elevation 380 m pH 5.7, T 12.2 °C

Red moss, *Vaccinium*, scattered dwarf birch and Labrador tea. The tundra in general seems very dry this season. Sample was just an ooze in the hummocks.

**Figures 79–84.**
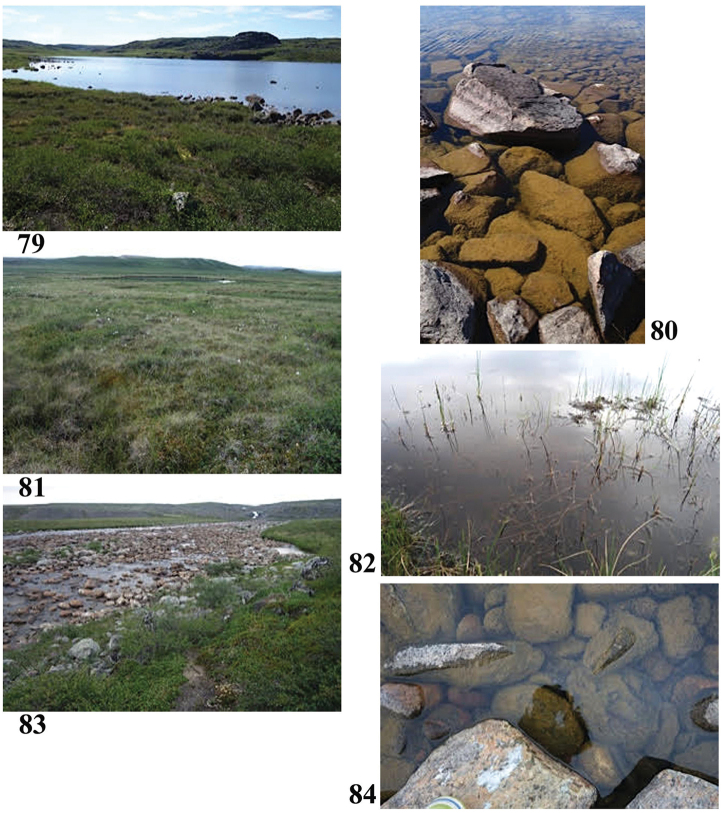
Collection sites along the Hood River corridor **79, 80** Site 5 (6902) **81, 82** Site 6 (6903) **83, 84** Site 7 (6904). Photos credit: Beverly Boynton.

Sample #5, large lake with short outlet to Hood (Figs 79, 80) July 8, 2017

66°38.165'N, 111°52.434'W Elevation 376 m pH 6.73, T 20.6 °C

Sample #6, wet meadow near Kapolak Camp (Figs 81, 82) July 11, 2017

66°37.970'N, 111°26.082'W Elevation 372 m pH 5.9, T 13.1 °C

Sedges, mosses, bottom with sediments and brown moss, sunny. In area of dwarf birch, Labrador tea.

Sample #7, Wright River (Figs 83, 84) July 15, 2017

66°50.173'N, 110°23.237'W Elevation 285 m pH 7.4, T 16.9 °C

Quiet pool on edge Wright River, a major tributary to the Hood River. Water clear, sunny, brown algae on rocks, no vegetation in water, the usual dwarf birch and tundra vegetation.

**Figures 85–90.**
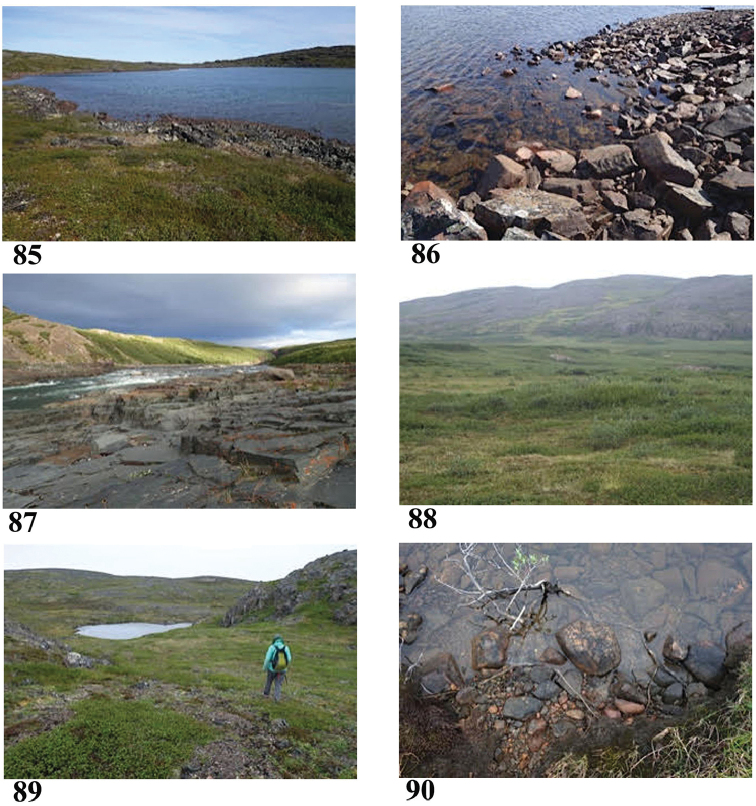
Collection sites along the Hood River corridor **85, 86** Site 8 (6905) **87** Site 9 (6906) **88** Site 11 (6907) **89, 90** Site 12 (6908). Photo credits: Beverly Boynton, Raymond White (Fig. **88**).

Sample #8, Wilberforce Hills, lake (Figs 85, 86) July 20, 2017

67°03.819'N, 108°40.383'W Elevation 273 m pH 7.24, T 11.8 °C

Large sunny lake with inlet from relatively high hills to the east and short outlet into the Hood River. Granite on shore, dwarf birch, willow, mosses, lichens, sedges, Ericaceae.

Sample #9, Hood River below Wilberforce Falls (Fig. 87) July 22, 2017

67°06.931'N, 108°49.194'W Elevation 35 m pH 6.80, T 15.7 °C

Tiny eddy with sand and gravel bottom, no plants or algae visible.

Sample #10, James River moist meadow (Fig. 88) July 23, 2017

67°12.274'N, 108°48.547'W Elevation 33 m pH 6.28, T 8.4 °C

Sample from standing water in a moist meadow to south of James River, a main tributary of the Hood River. Sedges, clear, sunny.

Sample #11, James River lake (Figs 89, 90) July 23, 2017

67°11.910'N, 108°50.912'W Elevation 236 m pH 7.26, T 13.0 °C

Sample from a moderately large lake that drains into the James River, though drainage was dry. No definite inlet noted, but is in basin of granite ridges. Sunny area, but had started to rain.

**Figures 91–97.**
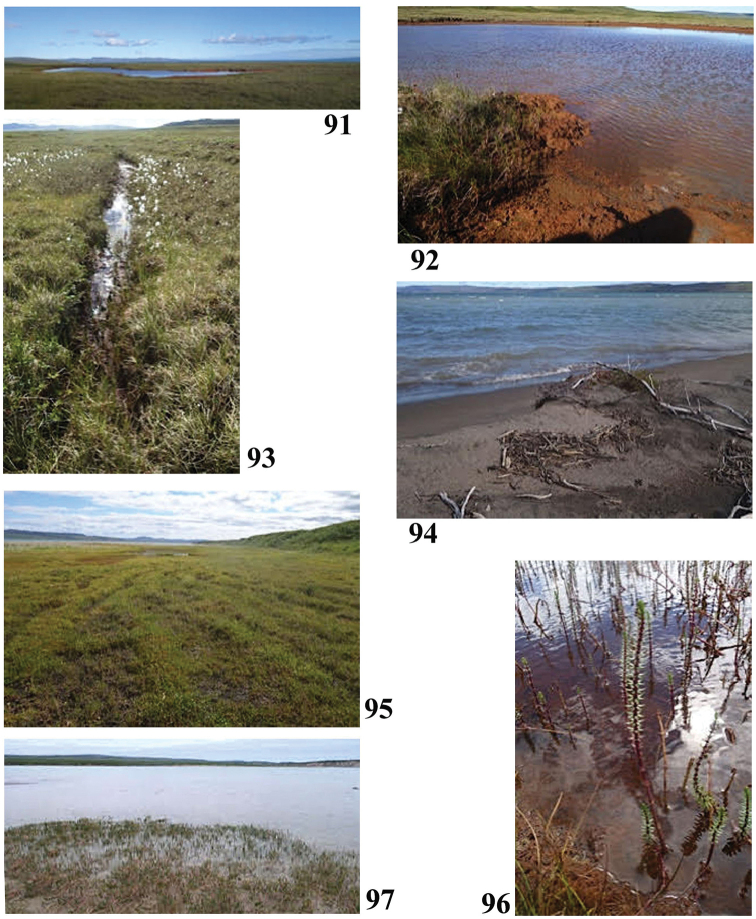
Collection sites along the Hood River corridor **91, 92** Site 13 (6909) **93** Site 14 (6910) **94** Site 15 (6911) **95, 96** Site 16 (6912) **97** Site 17 (6913). Photos credit: Beverly Boynton.

Sample #12, Red Sediment Lake (Figs 91, 92) July 27, 2017

67°23.024'N, 108°51.758'W (coordinates approximate) pH 8.5, T 13.7 °C

Sample from area of tundra on the peninsula that divides the final 8 km of the Hood River and its estuary from Baillie Bay. Dwarf birch, Ericaeae, sedges, cottongrass, heather, cranberry, willow. Includes a benthic sample.

Sample #13, ice wedge (Fig. 93) July 27, 2017

67°23.307'N, 108°51.931'W Elevation 23 m pH 5.9, T 16.1 °C

Moderately large ice wedge with standing water, on tundra of the peninsula that divides the final 8 km of the Hood River and its estuary from Baillie Bay. Dwarf birch, Ericaceae.

Sample #14, Arctic Sound of Bathurst Inlet (Fig. 94) July 27, 2017

67°24.994'N, 108°51.572'W Elevation 0 m pH 6.6, T 13.5 °C

Sample from sandy beach, with moderately strong north winds causing small surf. Area is somewhat south and east from the head of the peninsula that divides the final 8 km of the Hood River and its estuary from Baillie Bay. Tried to get surface water. No plants, sunny.

Sample #15, mare’s tail flooded area, Baillie Bay (Figs 95, 96) July 27, 2017

67°24.752'N, 108°51.423'W Elevation 0 m pH 8.5, T 18.0 °C

Mud flat flooded with water, with mare’s tails, sample from west side of Baillie Bay.

Sample #16, Hood River at last camp (Fig. 97) July 28, 2017

67°22.472'N, 108°53.183'W Elevation 5 m pH 7.14, T 9.6 °C

Sandy shore of Hood River, about 8 km upstream from its mouth in Arctic Sound.

## Figures and Tables

**Plate 1.**
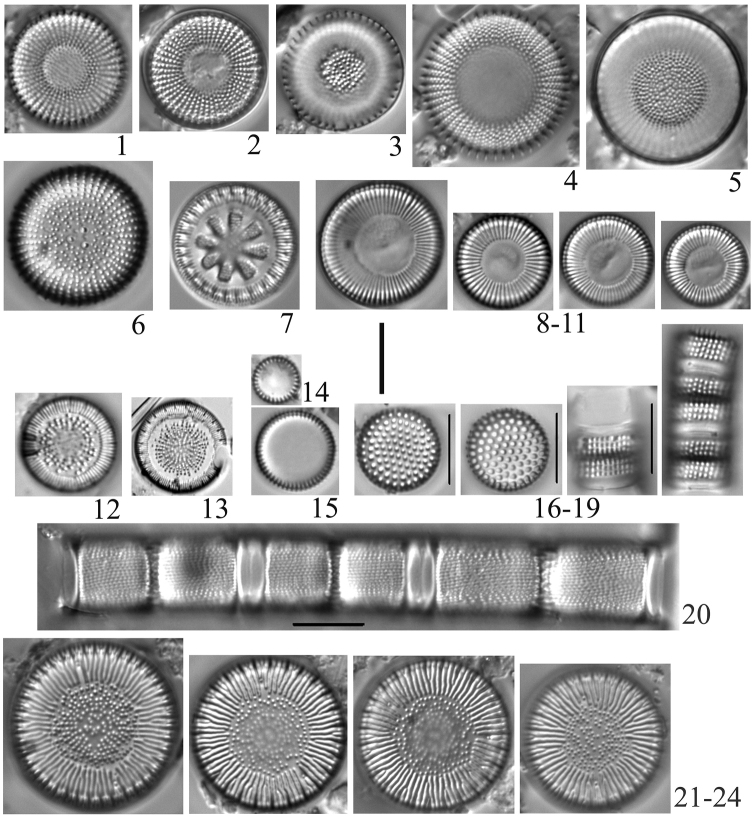
Waterton. **1–5** *Stephanodiscus alpinus* (4520, 4546). **2–3** and **4–5** are of the same valves **6** *Orthoseira roeseana* (4531) **7** *Lindavia antiqua* (4520) **8–11** *Cyclotella distinguenda* (4533) **12, 13** *Lindavia praetermissa* (4544) **14, 15** *Aulacoseira alpigena* (4569) **16–19** *Aulacoseira nivalis* (4569) **20** *Aulacoseira italica* (4536) **21–24** *Lindavia affinis* (4520). Scale bars: 10 µm.

**Plate 2.**
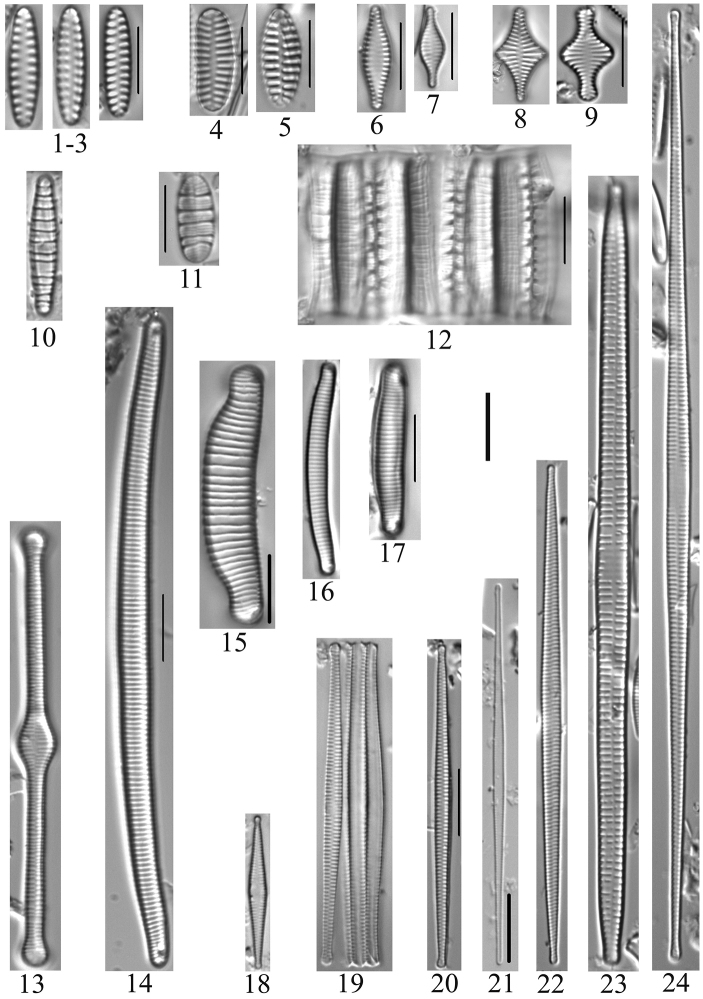
Waterton. **1–3** *Staurosirella lapponica* (4520, 4532, 4542) **4** *Staurosira* sp. (4545) **5** *Staurosirella* sp. [cf. *S. pinnata*] (4568) **6** Pseudostaurosira brevistriata var. inflata (4533) **7** *Staurosira oldenburgioides* (4562) **8** *Staurosirella* sp. [cf. *S. leptostauron*] (4562) **9** *Staurosira construens* (4542) **10** *Diatoma moniliformis* (4547) **11, 12** *Meridion lineare* (4532) **13** *Tabellaria flocculosa* (4520) **14** *Eunotia juettnerae* [*E. bilunaris* PH] (4538) **15** *Eunotia arcus* (4542) **16** *Eunotia botuliformis* (4520) **17** *Eunotia rhomboidea* (4569) **18** *Fragilaria* sp. (4520) **19** *Fragilaria crotonensis* (4532) **20, 22** *Fragilaria tenera* (4532) **21** *Fragilaria nanana* (4520) **23** *Ulnaria ulna* (4546) **24** *Synedra* sp. [cf. S. acus var. delicatissima] (4520). Scale bars: 10 µm.

**Plate 3.**
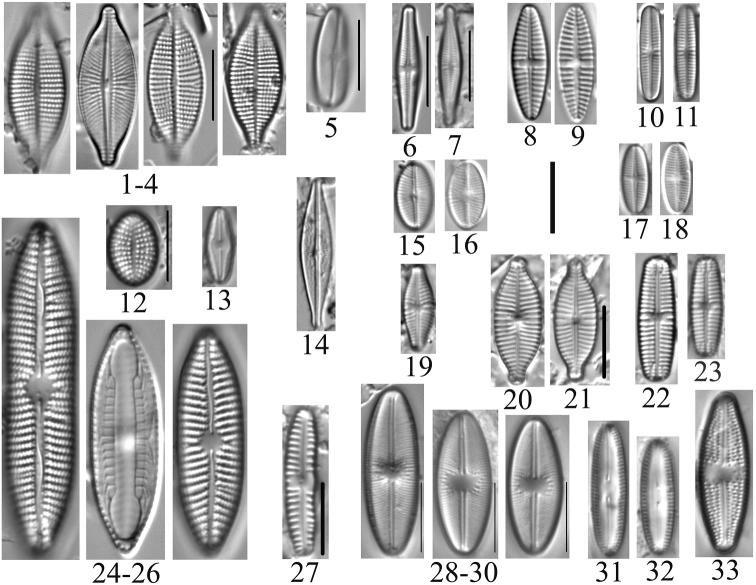
Waterton. **1–4** Karayevia clevei var. bottnica (4545) **5** *Eucocconeis alpestris* (4533) **6, 7** *Achnanthidium* sp. (4546, 4547) **8, 9** *Platessa conspicua* (4539) **10, 11** *Rossithidium pusillum* (4520) **12** *Cocconeis pseudothumensis* (4545) **13** *Diadesmis perpusilla* (4520) **14** *Brachysira microcephala* (4520) **15, 16** *Psammothidium* sp. [cf. *P. daonense* PH] (4569) **17, 18** *Psammothidium curtissimum* [*P. saccula* PH] (4520) **19** *Geissleria* (?) sp. (4520) **20, 21** *Geissleria similis* (4520, 4570) **22, 23** *Geissleria paludosa* (4520, 4542) **24–26** *Mastogloia grevillei* (4533) **27** *Reimeria* sp. [*R. sinuata* PH] (4520) **28–30** *Cavinula davisiae* (4562) **31, 32** *Diatomella balfouriana* (4562) **33** *Luticola mutica* (4561). Scale bars: 10 µm.

**Plate 4.**
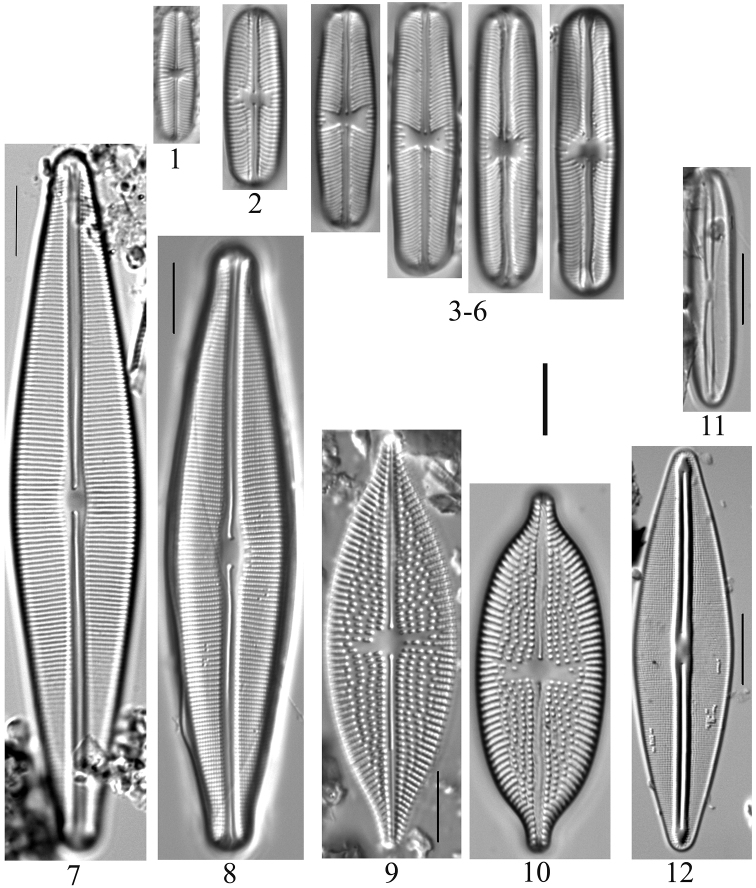
Waterton. **1** *Sellaphora pupula* (4570) **2** *Sellaphora laevissima* (4568) **3–6** *Sellaphora parapupula* (4520, 4531, 4542) **7** *Craticula sardiniana* (4543) **8** *Craticula johnstoniae* (4561) **9** *Aneumastus rostratus* (4547) **10** *Aneumastus tusculus* (4540) **11** *Frustulia amosseana* (4543) **12** *Frustulia saxonica* (4569). Scale bars: 10 µm.

**Plate 5.**
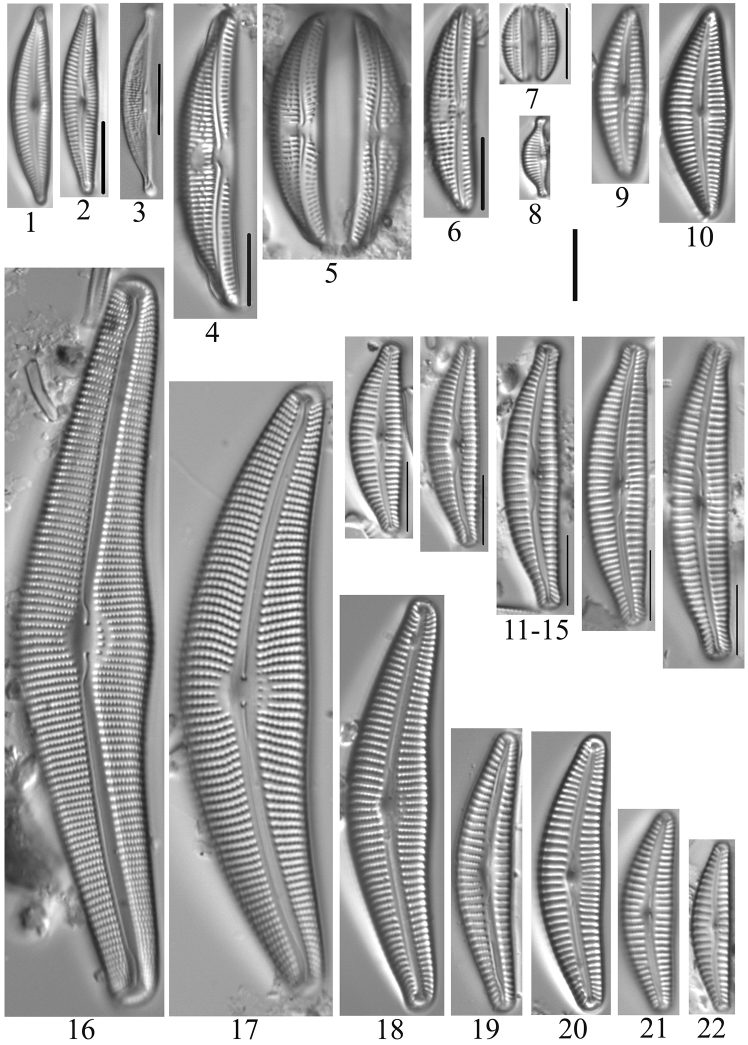
Waterton. **1, 2** *Delicata delicatula* (4534) **3** *Halamphora coraensis* (4543) **4, 5** *Amphora copulata* (4542, 4562) **6** *Amphora* sp. (4543) **7** *Amphora pediculus* (4562) **8** *Amphora thumensis* (4545) **9** *Cymbella neoleptoceros* (4542) **10** *Cymbella stigmaphora* (4534) **11–15** *Cymbella excisiformis* (4546). Valves in this population have two stigmata **16** Cymbella neocistula var. islandica (4520) **17** *Cymbella proxima* (4520) **18** *Cymbella neocistula* (4534) **19** *Cymbella alpestris* (4541) **20, 21** *Cymbella hantzschiana* (4534) **22** *Cymbella cosleyi* (4520). Scale bars: 10 µm.

**Plate 6.**
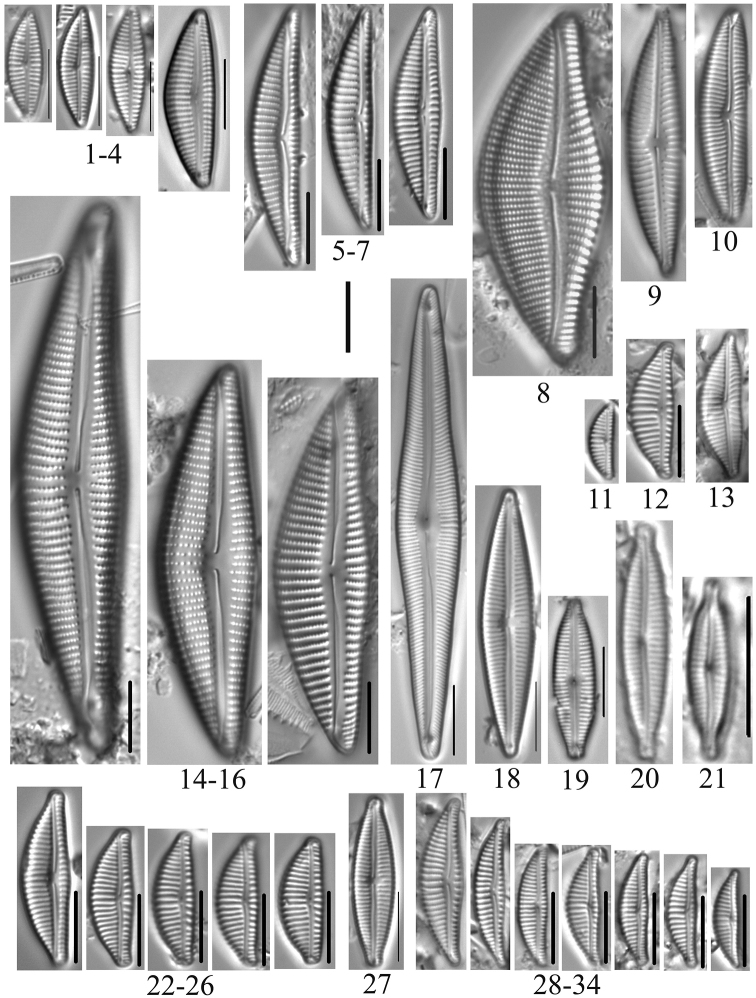
Waterton. **1–4** *Encyonema* sp. (4543, 4569) **5–7** *Encyonema procerum* [*E. silesiacum* sensu lato PH] (4542) **8** *Encyonema temperei* [cf. *E. temperei* PH] (4542) **9** *Encyonema hebridicum* (4569) **10** *Encyonema norvegicum* (4542) **11** *Encyonema minutum* (4520) **12** *Encyonema ventricosum* (4568) **13** *Encyonema hamsherae* (4570) **14–16** *Encyonema hintzii* (4539, 4542) **17** *Encyonopsis montana* (4545) **18** *Encyonopsis* sp. (4569) **19** *Encyonopsis* sp. (4569) **20** *Encyonopsis subminuta* (4520) **21** *Encyonopsis alpina* [*E. microcephala* PH] (4547) **22–26** *Encyonema ventricosum* (4520, 4568) **27** *Kurtkrammeria aequalis* (4569) **28–34** *Encyonema fogedii* (4520). Scale bars: 10 µm.

**Plate 7.**
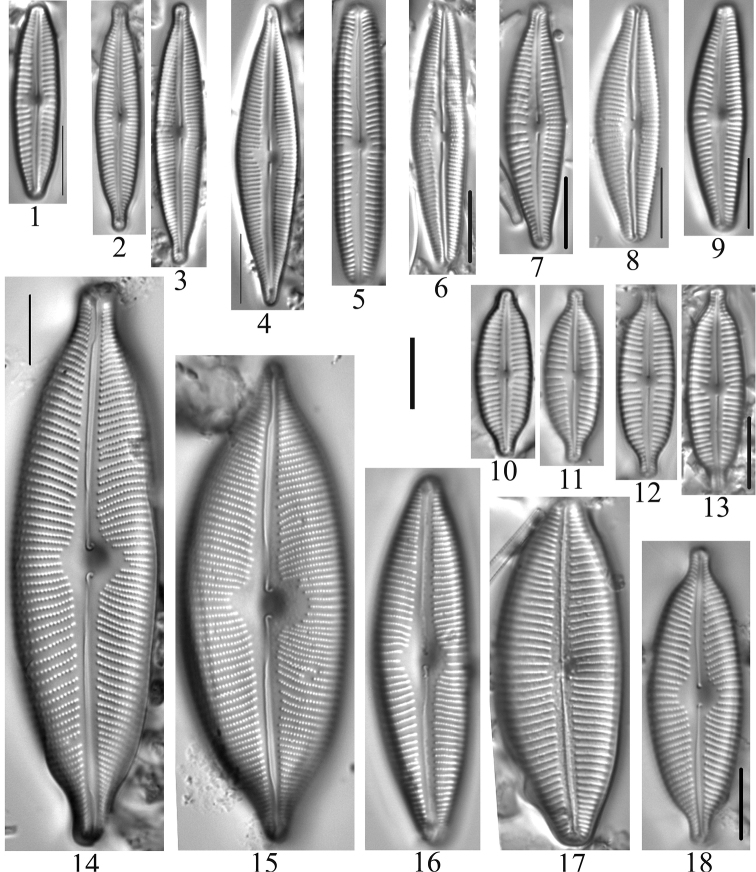
Waterton. **1** *Cymbopleura hybrida* (4533, 4545) **2, 3** *C. angustata* [C. angustata var. tenuis PH] (4533, 4544) **4** *C. lapponica* (4533, 4547) **5** *C. oblongata* (4533, 4546, 4547) **6** *C. incerta* [C. incerta var. grunowii PH] (4547) **7** *C. rupicola* [cf. *C. rupicola* PH] (4547) **8** *C. heilprinensis* [cf. *C. heilprinensis* PH] (4534) **9** *C. subaequalis* [cf. *C. florentina* PH] (4533, 4534, 4547) **10, 11** *C. amphicephala* [*C. similiformis* PH] (4520) **12, 13** *C. similiformis* (4533, 4547) **14** *C. subcuspidata* (4534, 4542) **15** *C. apiculata* (4540) **16** *C. rainierensis* [= *Cymbella gondwana*?] (4569) **17** *C. lata* (4545) **18** *C. anglica* (4520). Scale bars: 10 µm.

**Plate 8.**
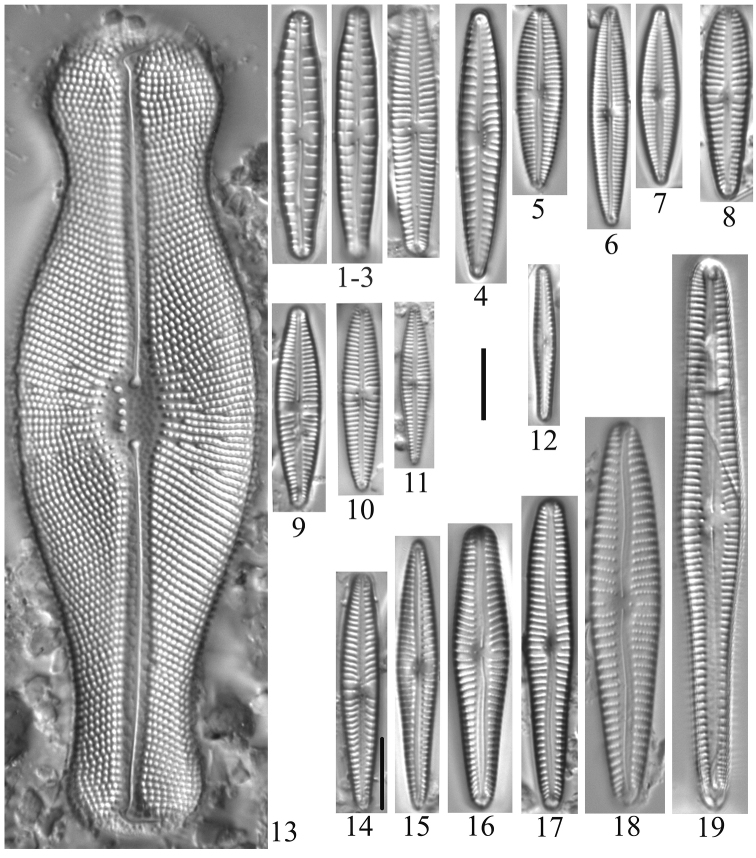
Waterton. **1–3** *Gomphonema sarcophagus* [Fig. **3** *Gomphonema* sp. PH] (4532, 4536) **4, 5** *Gomphonema multipunctatum* (4536) **6, 7** *Gomphonema hebridense* (4531, 4539) **8** *Gomphonema minutum* (4520) **9** *Gomphonema exilissimum* (4532) **10** *Gomphonema* sp. (4539) **11** *Gomphonema auritum* (4570) **12** *Gomphonema pygmaeum* (4520) **13** *Didymosphenia geminata* (4547) **14** *Gomphonema longilineare* [*G. cymbelliclinum* PH] (4547) **15** *Gomphonema acidoclinatum* (4543) **16, 17** *Gomphonema subclavatum* (4536, 4538, 4540) **18** *Gomphonema affine* (4531) **19** *Gomphonema* sp. (4536). Scale bars: 10 µm.

**Plate 9.**
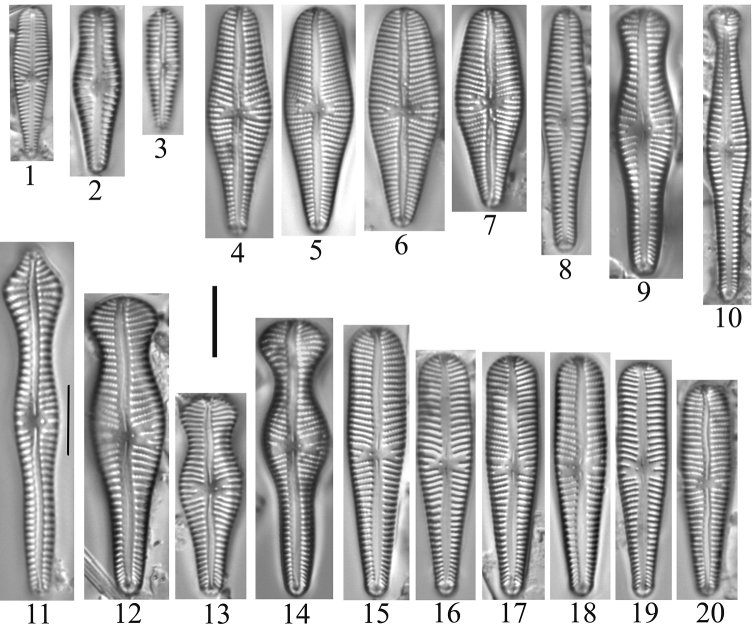
Waterton. **1** *Gomphosinica geitleri* (4520) **2** *Gomphonema* sp. [cf. *G. capitatum*] (4543) **3** *Gomphonema angusticephalum* (4542) **4–7** *Gomphonema pala* (4539) **8** *Gomphonema* sp. (4520) [cf. *Gomphonema angusticlavatum* or cf. *G. subclavatum* (KJ)] **9** *Gomphonema capitatum* (4532) **10** *Gomphonema subtile* (4542) **11** *Gomphonema brebissonii* sensu lato (4543) **12, 13** *Gomphonema truncatum* sensu lato (4545) **14** *Gomphonema anglicum* (4542) **15–20** *Gomphonema* sp. [*G. sublaticollum* (KJ)] (4562, 4570). Scale bar: 10 µm.

**Plate 10.**
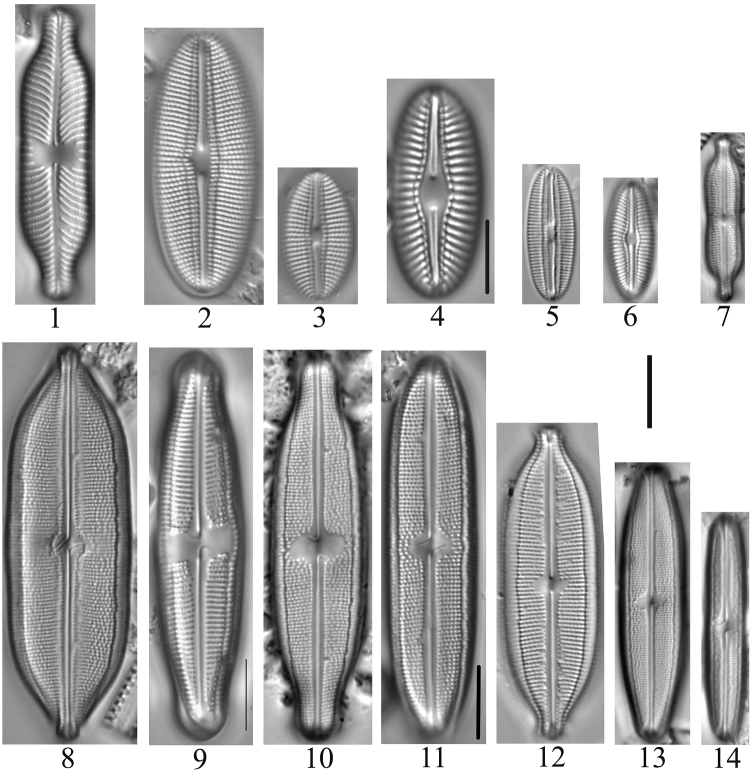
Waterton. **1** *Placoneis abiskoensis* (4542, 4545) **2, 3** *Diploneis parma* (4520) **4** *Diploneis pseudovalis* (4543) **5** *Diploneis oculata* (4533) **6** *Diploneis oblongella* (4533) **7** *Neidiomorpha binodiformis* (4533) **8** *Neidium apiculatum* (4542) **9** *Neidium fogedii* (4543) **10** Neidium affine var. longiceps [*Neidium* sp. PH] (4570) **11** *Neidium* sp. (4543) **12** *Neidium dubium* (4520, 4542) **13** *Neidium affine* [*Neidium potapovae* PH] (4569) **14** *Neidium bisulcatum* (4532). Scale bars: 10 µm.

**Plate 11.**
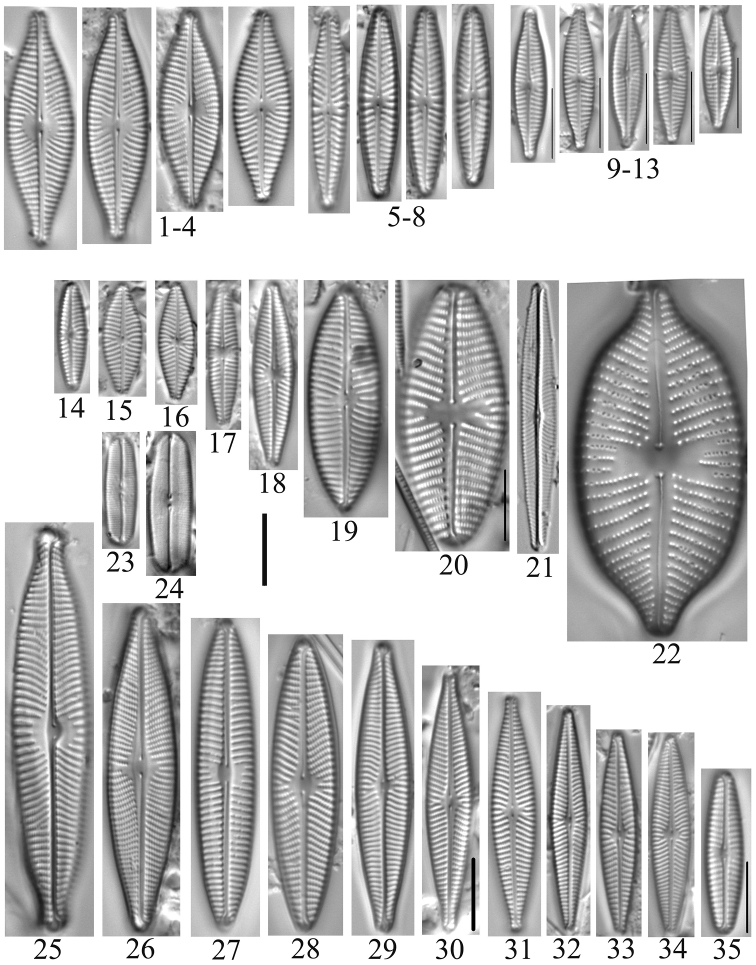
Waterton. **1–4** *Navicula caroliniae* (4532, 4539, 4570) **5–8** *N. seibigiana* (4533) **9–13** *N. trilatera* (4533) **14** *N. arctotenelloides* (4520) **15** *N. antonii* (4570) **16** *N. reichardtiana* (4570) **17** *N. veneta* [cf. *N. veneta* PH] (4570) **18** *N. lundii* (4562) **19** *N. upsaliensis* (4545) **20** *N. reinhardtii* (4545) **21** *N. leptostriata* (4569) **22** N. (Placoneis) amphibola (4540) **23** *N. lenzii* (4545) **24** *N. subhamulata* (4520) **25** *N. viridulacalcis* (4568) **26** *N. trivialis* (4533) **27** *N. tripunctata* (4562) **28** *N. weberi* (4520) **29** *N. schweigeri* (4546, 4568) **30, 31** *N. wildii* (4534, 4545, 4547, 4568) **32** *N. cryptotenella* (4520) **33** *N. notha* [*Navicula* sp. PH] (4533, 4540, 4541 4542) **34** *N.* sp. (4520) **35** *N. libonensis* (4545). Scale bars: 10 µm.

**Plate 12.**
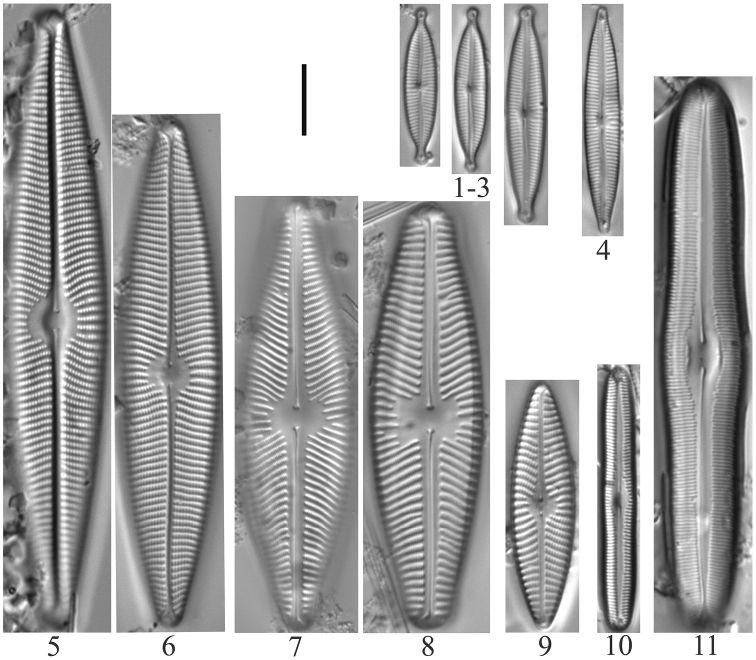
Waterton. **1–3** *Encyonopsis czarneckii* [*E. alpina* or *E. subminuta* PH] (4533) **4** *Encyonopsis cesatii* (4533) **5, 6** *Navicula vulpina* (4520) **7, 8** *Navicula aurora* (4520) **9** *Navicula weberi* (4520) **10** *Caloneis tenuis* (4520) **11** *Caloneis* sp. (4532). Scale bar: 10 µm.

**Plate 13.**
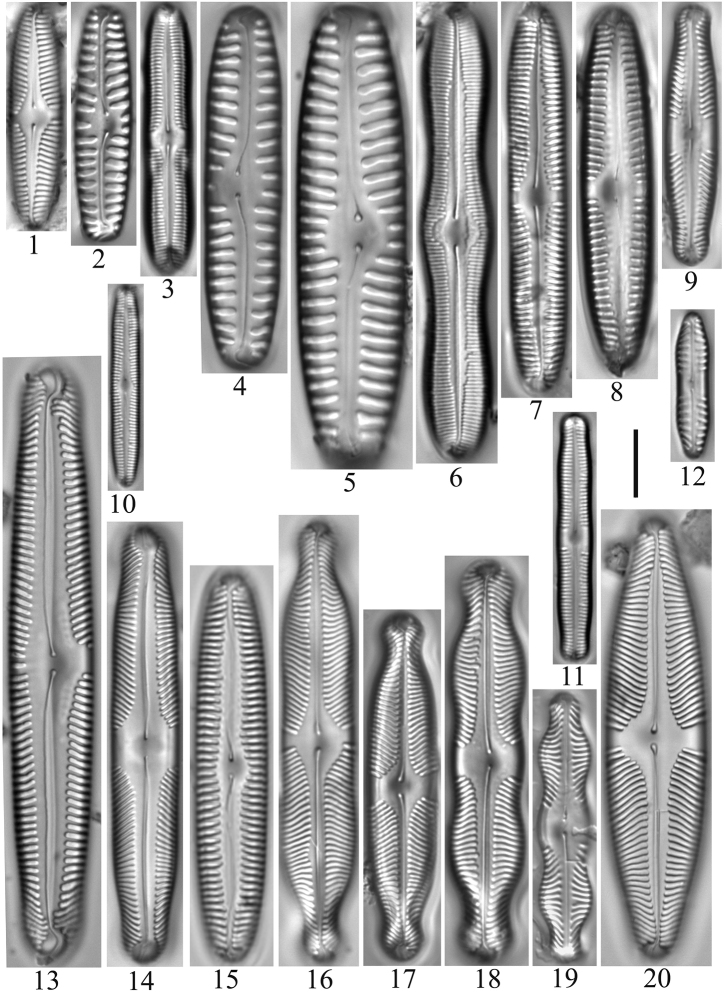
Waterton. **1** *Pinnularia brebissonii* (4570) **2** *Pinnularia borealis* (4561) **3** *Caloneis* sp. [*C. bacillum* sensu lato PH] (4531) **4** Pinnularia borealis var. scalaris (4561) **5** *Pinnularia rabenhorstii* (4561) **6** *Caloneis schumanniana* (4543, 4547) **7** *Pinnularia crucifera* (4543) **8** *Pinnularia isostauron* (4561) **9** *Pinnularia lunata* (4531) **10** *Caloneis tenuis* (4520) **11** *Caloneis undulata* [*Caloneis* sp. PH] (4520, 4540, 4543) **12** *Pinnularia obscura* (4568) **13** *Pinnularia sudetica* (4569) **14** *Pinnularia microstauron* (4537, 4543) **15** *Pinnularia viridis* [P. complexa var. minor PH] (4531) **16** *Pinnularia biceps* (4569, 4570) **17** *Pinnularia anglica* [*P. biceps* PH] (4534) **18** *Pinnularia septentrionalis* (4568) **19** *Pinnularia turbulenta* (4534) **20** *Pinnularia pseudosuchlandtii* (4569). Scale bar: 10 µm.

**Plate 14.**
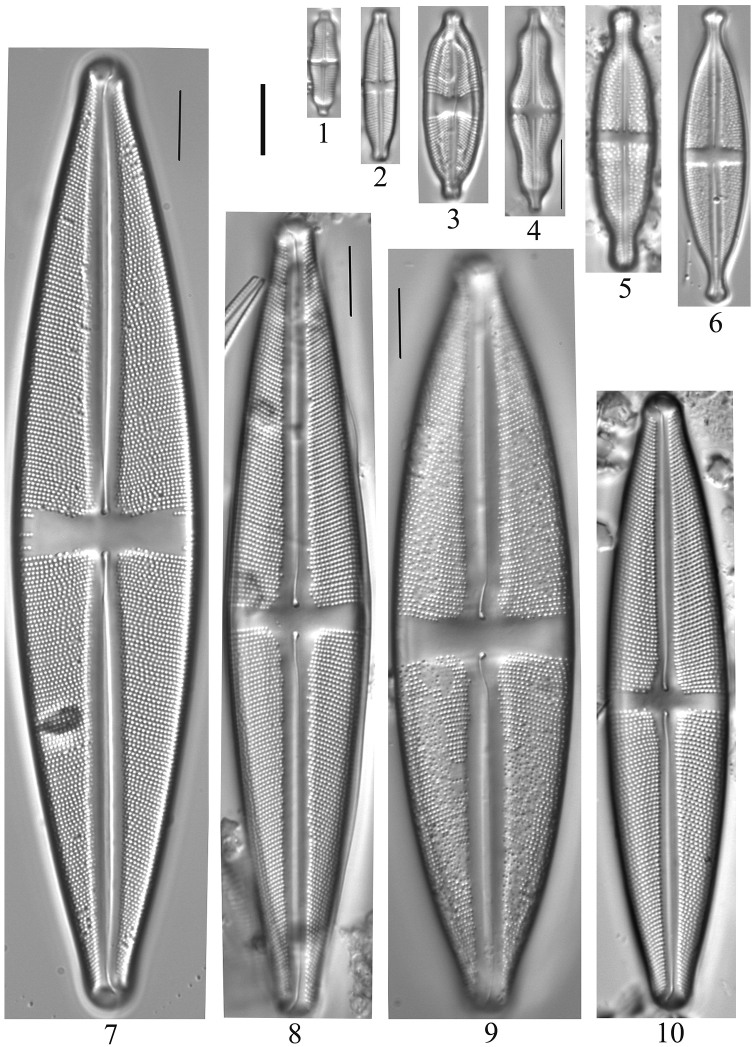
Waterton. **1** *Stauroneis separanda* (4532) **2** *Stauroneis kriegeri* (4562) **3** *Stauroneis* sp. (4535) **4** *Stauroneis smithii* (4562) **5** *Stauroneis lauenburgiana* (4532) **6** *Stauroneis kootenai* (4561) **7** *Stauroneis heinii* (4541, 4561) **8** *Stauroneis phoenicenteron* (4532, 4539) **9** *Stauroneis circumborealis* (4561) **10** *Stauroneis gracilis* (4532, 4539). Scale bars: 10 µm.

**Plate 15.**
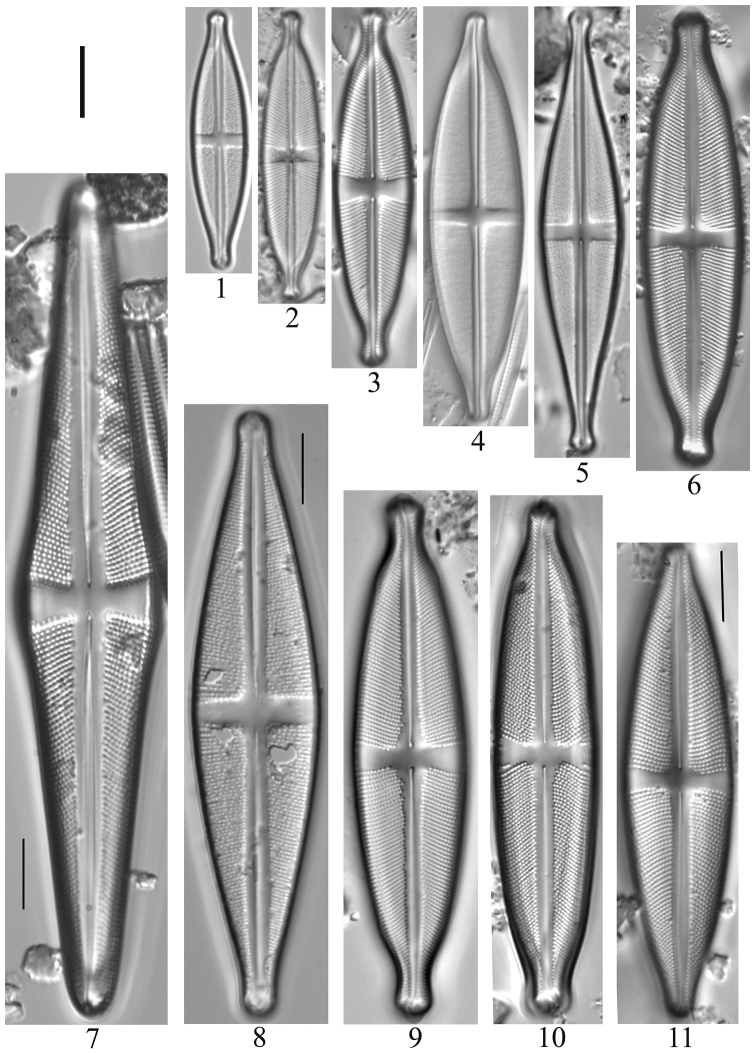
Waterton. **1** *Stauroneis* sp. [cf. *S. silvahassiaca*] (4543) **2** *Stauroneis reichardtii* (4561, 4570) **3** *Stauroneis vandevijveri* (4533, 4534, 4539, 4570) **4** *Stauroneis siberica* (4520) **5** *Stauroneis pikuni* (4532) **6** *Stauroneis jarensis* (4532) **7** *Stauroneis acuta* (4539) **8** *Stauroneis akamina* (4561) **9** *Stauroneis amphicephala* (4542) **10** *Stauroneis pax* (4530, 4547) **11** *Stauroneis conspicua* (4520, 4533, 4534). Scale bars: 10 µm.

**Plate 16.**
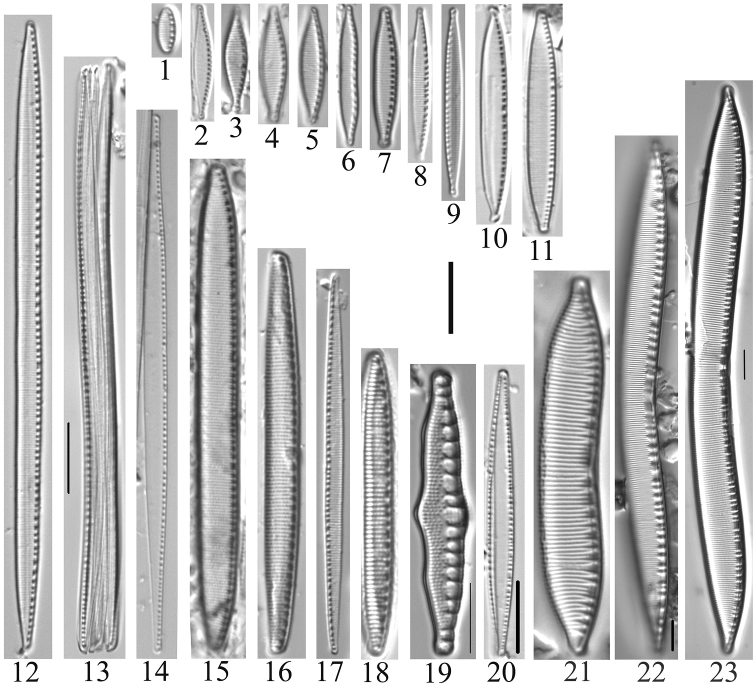
Waterton. **1** *Nitzschia inconspicua* (4520) **2** *Nitzschia lacuum* (4542) **3, 4** *Nitzschia bacillum* (4520, 4533) **5** Nitzschia fonticola var. pelagica [*Nitzschia bacillum* PH] (4520) **6** *Nitzschia acidoclinata* (4540) **7** *Nitzschia alpina* (4568) **8, 9** *Nitzschia perminuta* (4541, 4542) **10** *Nitzschia* sp. [*Nitzschia palea* sensu lato PH] (4533) **11** *Nitzschia palea* (4520) **12** *Nitzschia diversa* or *N. gessneri* (4536, 4542) **13** *Nitzschia vermicularis* (4562) **14** *Nitzschia gracilis* (4520) **15** Nitzschia regula var. robusta (4547) **16** *Nitzschia frauenfeldii* (4541) **17** *Nitzschia radicula* (4539) **18** *Nitzschia amphibia* (4541) **19** *Nitzschia sinuata* (4540) **20** *Nitzschia pura* (4520) **21** *Hantzschia abundans* (4531) **22, 23** *Hantzschia elongata* (4531, 4540, 4541). Scale bars: 10 µm. Note different scale for images in Figs 22 and 23.

**Plate 17.**
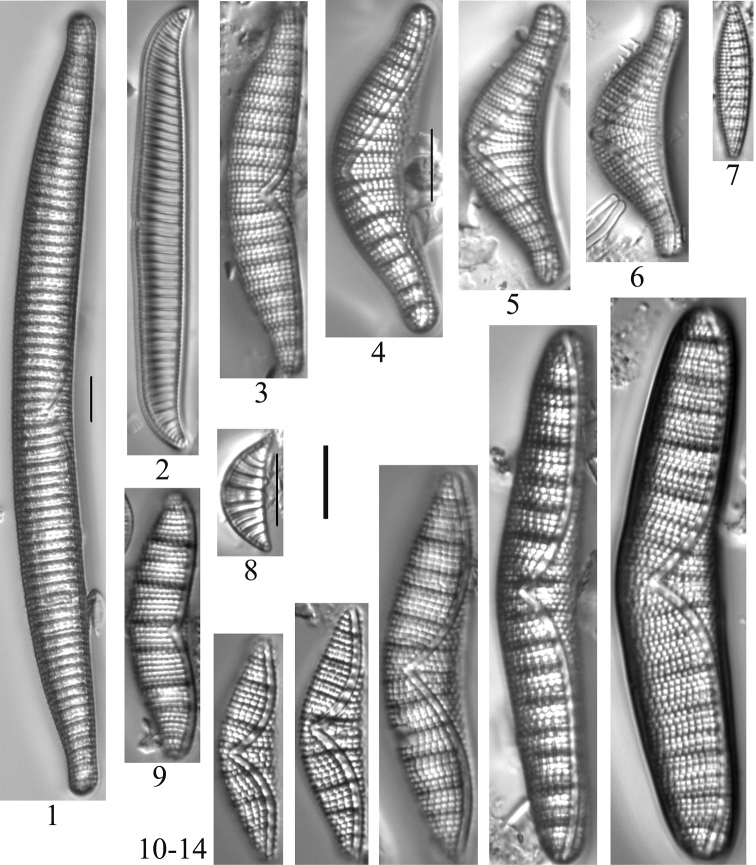
Waterton. **1** Epithemia turgida var. granulata (4541) **2** *Rhopalodia gibba* (4533) **3** *Epithemia adnata* (4532) **4–6** *Epithemia smithii* (4520, 4535) **7** *Denticula kuetzingii* (4544) **8** *Rhopalodia operculata* (4570) **9** *Epithemia* sp. (4543) **10–14** *Epithemia argus* (4541, 4543). Scale bars: 10 µm.

**Plate 18.**
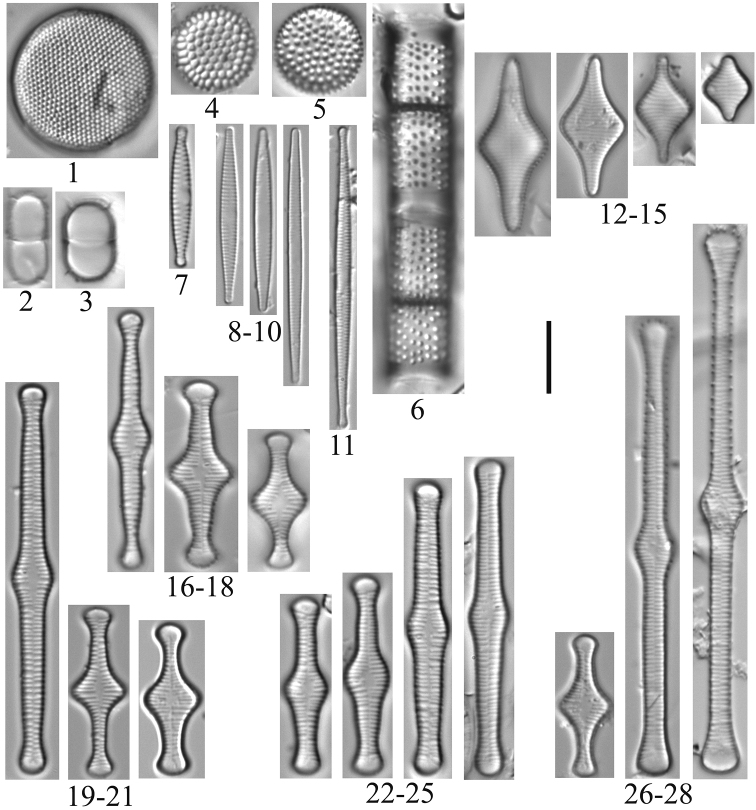
Haida Gwaii. **1** *Thalassiosira* sp.? (6890) **2, 3** *Melosira nummuloides* (6890) **4** *Coscinodiscus* sp. (6890) **5** *Aulacoseira* sp. (5064) **6** *Aulacoseira crassipunctata* (5065) **7** *Fragilaria vaucheriae* (5067) **8–10** Fragilaria capucina var. rumpens (5063, 5066, 5067) **11** *Fragilaria capucina* (5066) **12–15** *Fragilariforma polygonata* (?) (5064) **16–28** *Tabellaria flocculosa* **16–18** 5066 **19–21** 5063 **22–25** 6890 **26–28** 5064. Scale bar: 10 µm.

**Plate 19.**
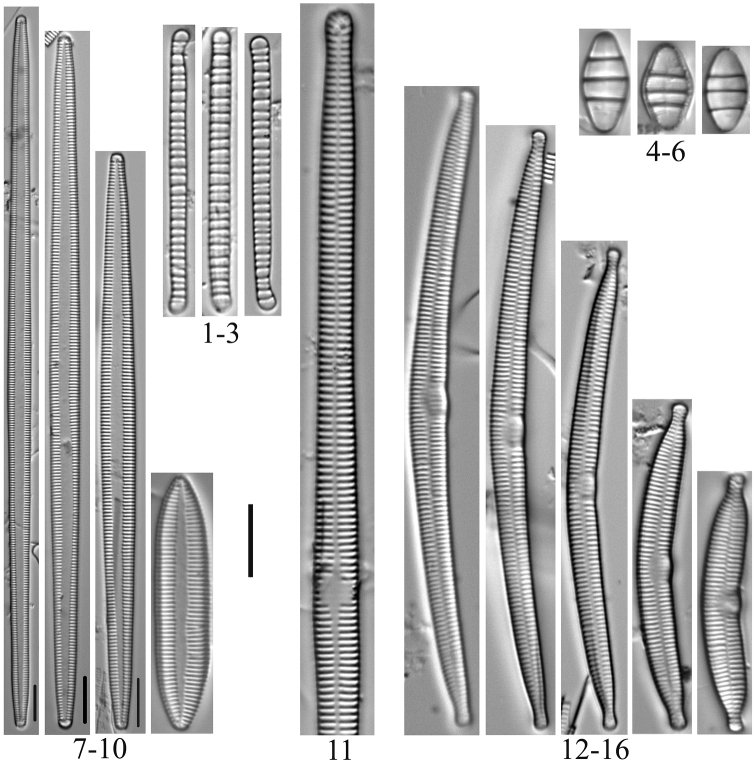
Haida Gwaii. **1–3** *Diatoma tenuis* (5062) **4–6** *Odontidium mesodon* (5066, 6890) **7–10** *Tabularia fasciculata* [Fig. **10** *Tryblionella* sp.? PH] (6890) **11** *Ulnaria* sp. (6890) **12–16** *Hannaea arcus* (6890). Scale bars: 10 µm; scale bar on plate applies to images without scale bars; note different scales of images **7–10**.

**Plate 20.**
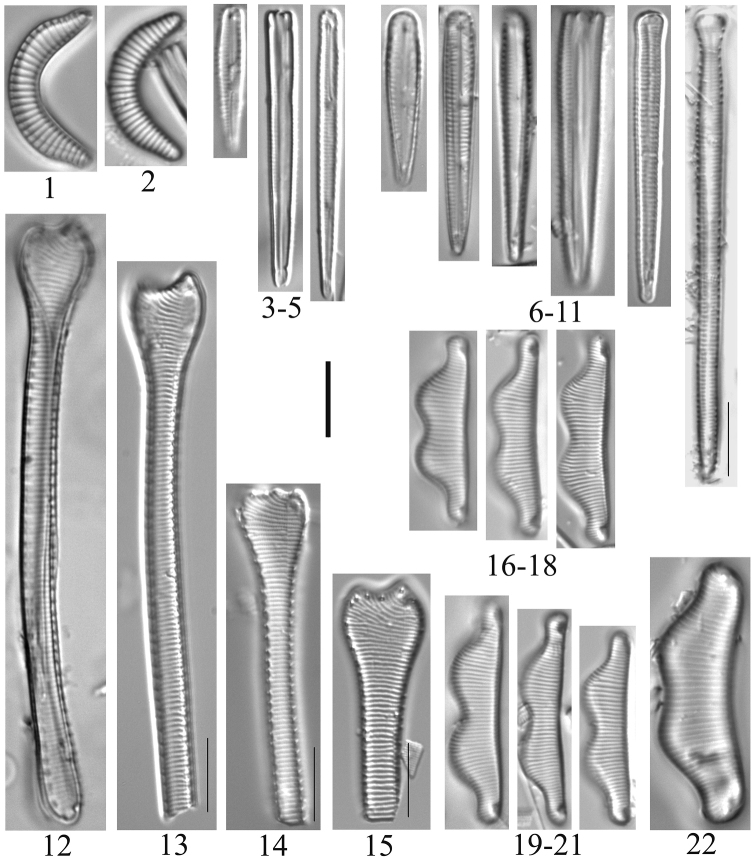
Haida Gwaii. **1, 2** *Semiorbis rotundus* (5064) **3–11** *Peronia fibula* **3–5** 5064 **6–11** 5065 **12–15** *Actinella punctata* (5064) **16–21** *Eunotia bidentula* (5064, 5066) **22** *Eunotia islandica* (5064). Scale bars: 10 µm.

**Plate 21.**
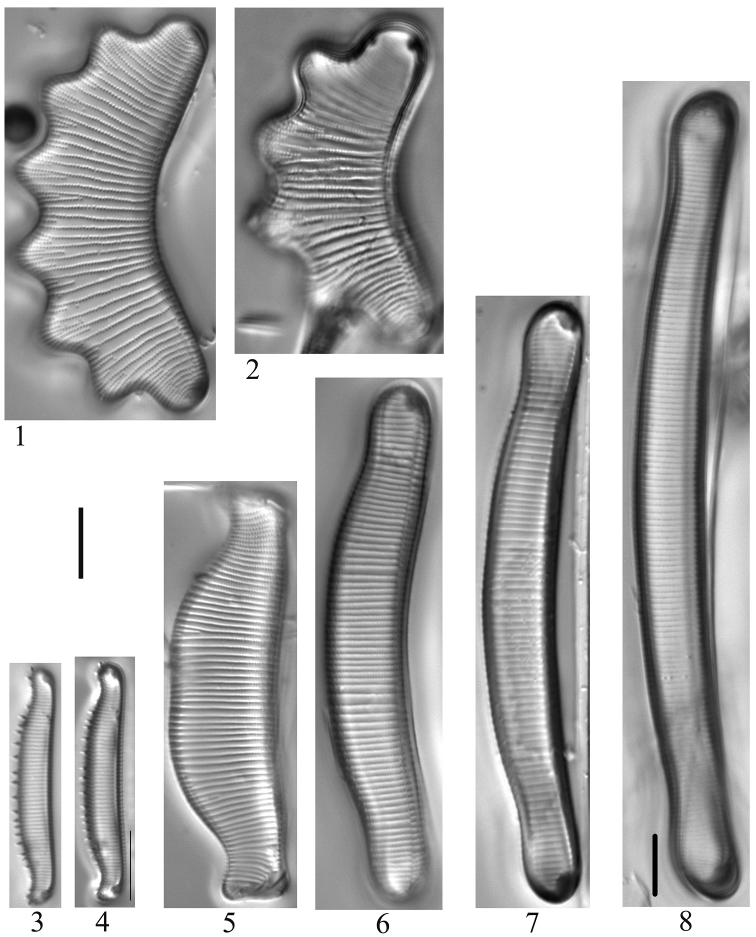
Haida Gwaii. **1** *Eunotia diadema* (5064, 5065, 5067) **2** *E. tetraodon* (5065) **3, 4** *E. neoborealis* [*E. fennica* PH] (5063) **5** *E. superbidens* (5064) **6–8** *E. metamonodon* (5063, 5065, 5067). Scale bars: 10 µm. Note different scales of 7 and 8; scale bar on plate applies to images without scale bars.

**Plate 22.**
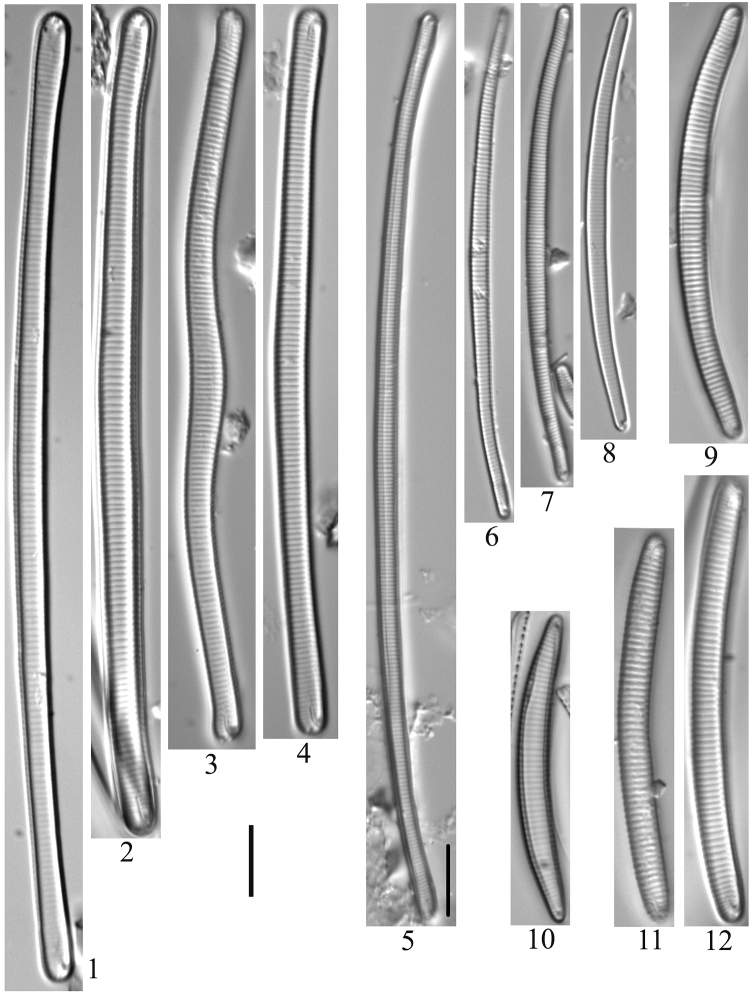
Haida Gwaii. **1–4** *Eunotia flexuosa* (5063, 5066, 5067) **5–8** *E. naegelii* (5063, 5065, 5066, 6890) **9** *E. bilunaris* (5064, 5067) **10** *Eunotia* sp. [cf. *E. mertensiae*] (5062) **11, 12** *Eunotia* sp. [cf. *E. julma*] [cf, *Eunotia furyae* PH] (5063). Scale bar: 10 µm.

**Plate 23.**
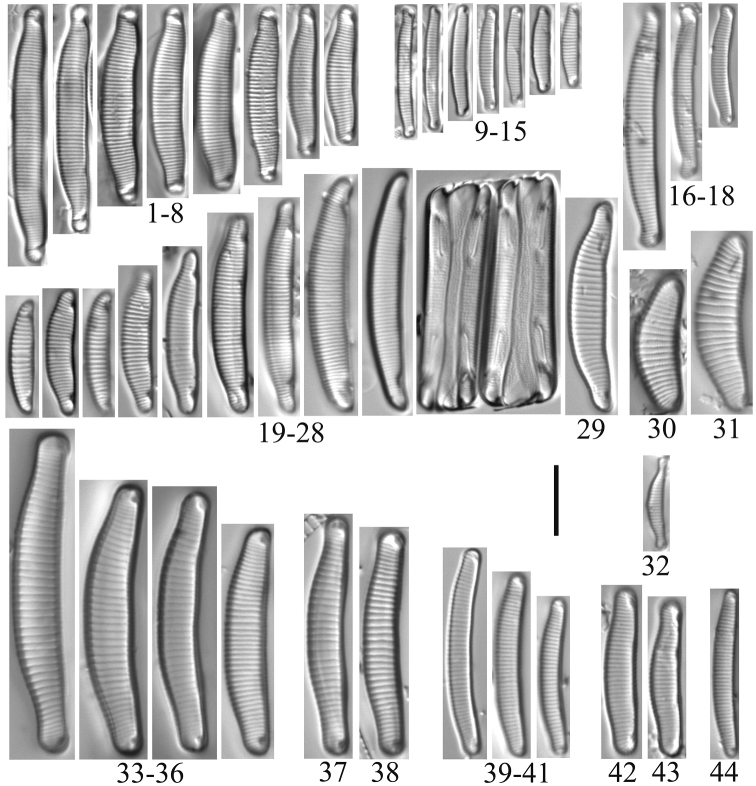
Haida Gwaii. **1–8** *Eunotia lewisii* (5064, 6888) **9–15** *E. paludosa* (5064, 6888) **16–18** *E. superpaludosa* (5064) **19–28** *E. incisa* (5063, 5064, 5066, 5067) **29** *Eunotia* sp. [cf. *E. incisa*] (5064) **30, 31** *Eunotia* sp. [cf. *E. boreotenuis*] (5064) **32** *E. arculus* (5064) **33–36** *E. minor* (5063) **37, 38** *Eunotia* sp. [cf. *E. minor*] (5062) **39–41** *E. botuliformis* (5066) **42, 43** *Eunotia* sp. [cf. *E. arcofallax*] [cf. *E. rhomboidea* PH] (5064) **44** *E. subarcuatoides* (5063). Scale bar: 10 µm.

**Plate 24.**
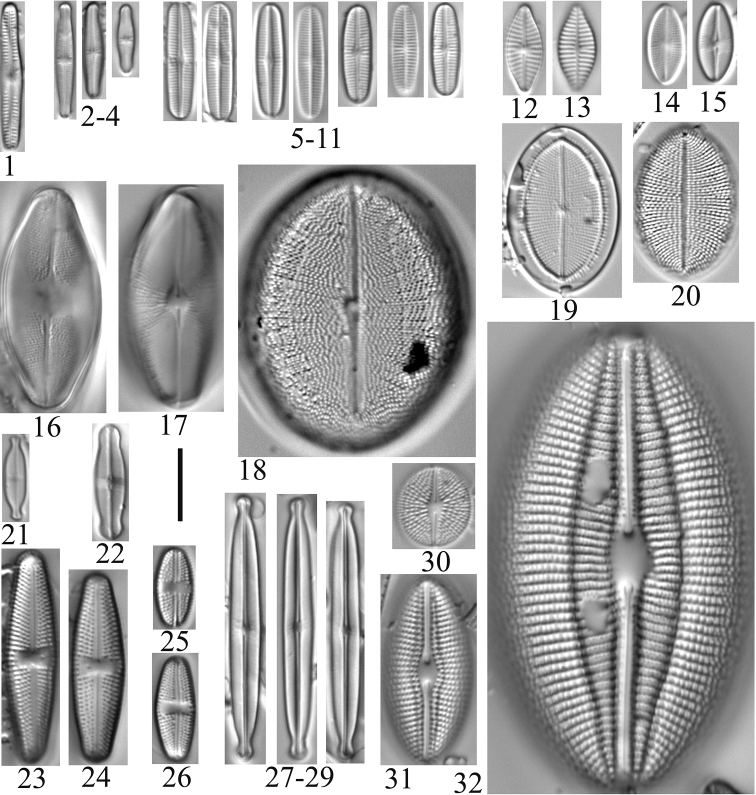
Haida Gwaii. **1** *Achnanthidium kriegeri* (5066) **2–4** *Achnanthidium minutissimum* (5063, 5066, 6890) **5–11** *Rossithidium pusillum* (5063, 5065, 5067) **12, 13** *Planothidium delicatulum* (6890) **14, 15** *Psammothidium nivale* (6890) **16, 17** *Eucocconeis flexella* (5065, 5066) **18** *Cocconeis* sp. (5062) **19, 20** *Cocconeis placentula* (5062) **21** *Nupela tenuicephala* (5064) **22** Unknown genus (5063) **23, 24** *Luticola* sp. (6889) **25, 26** *Luticola mutica* (6890) **27–29** *Kobayasiella parasubtilissima* (5064, 6888) **30** *Cavinula pseudoscutiformis* (5065) **31** *Diploneis elliptica* (5062, 5065) **32** *Diploneis finnica* (5065). Scale bar: 10 µm.

**Plate 25.**
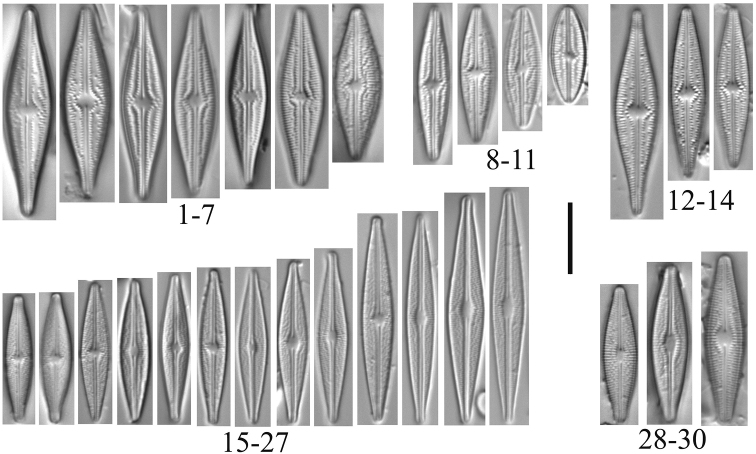
Haida Gwaii. **1–7** *Brachysira* sp. [cf. *B. neoacuta* PH] (5065, 5067) **8–11** *B. brebissonii* (5063, 5064, 5065) **12–14** *B.* sp. [cf. *B. neoacuta* PH] (5065) **15–27** *B. procera* [PH] or *B. ocalanensis* (5063, 5065, 5066, 5067) **28–30** *B.* sp. (5064, 5065). Scale bar: 10 µm.

**Plate 26.**
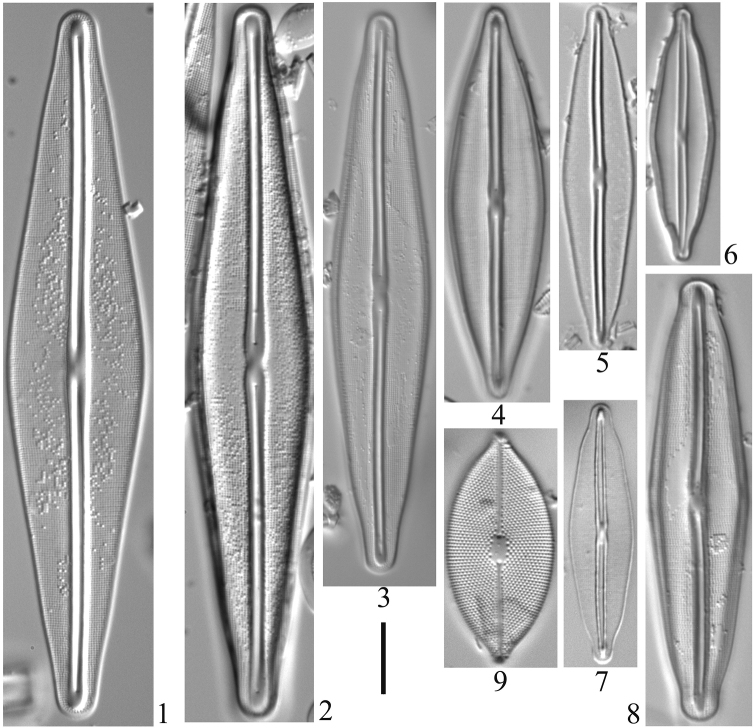
Haida Gwaii. **1** *Frustulia saxonica* (5064, 6888) **2–7** *Frustulia crassinervia* (5063, 5064, 5065, 5066, 5067, 6888) **8** *Frustulia quadrisinuata* (5063) **9** *Decussata placenta* (5067). Scale bar: 10 µm.

**Plate 27.**
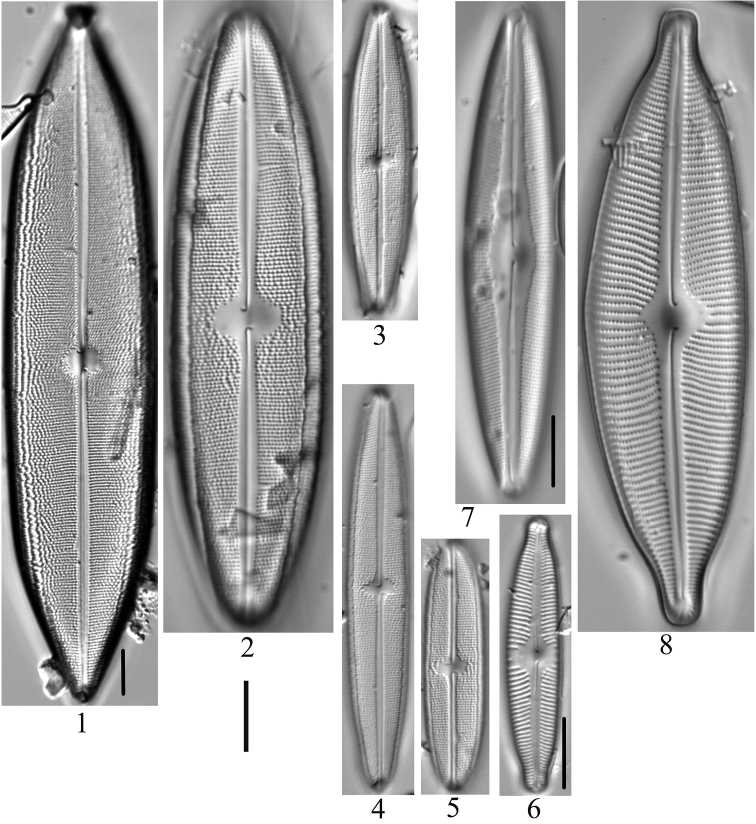
Haida Gwaii. **1** *Neidium amphigomphus* (5064) **2** *Neidium fossum* (6890) **3, 4** *Neidium affine* (5064) **5** *Neidium* sp. [*N. occidentale* PH] (5064) **6** *Cymbopleura fluminea* [*C. hybrida* or *C. lineare* PH] (5063) **7** *Cymbopleura* sp. (5063) **8** *Cymbopleura subcuspidata* (5065). Scale bars: 10 µm.

**Plate 28.**
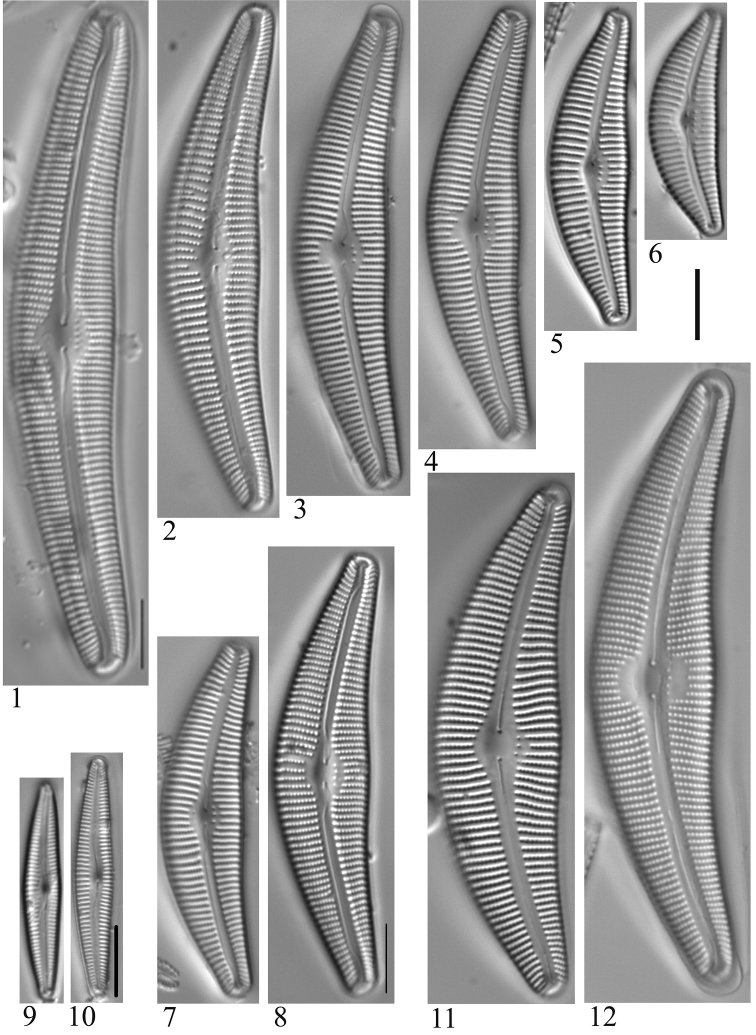
Haida Gwaii. **1–8** *Cymbella neocistula* (5062, 5063, 5066, 6890) **9, 10** *Delicata alpestris* (5063) **11, 12** *Cymbella proxima* (5062, 5063). Scale bars: 10 µm.

**Plate 29.**
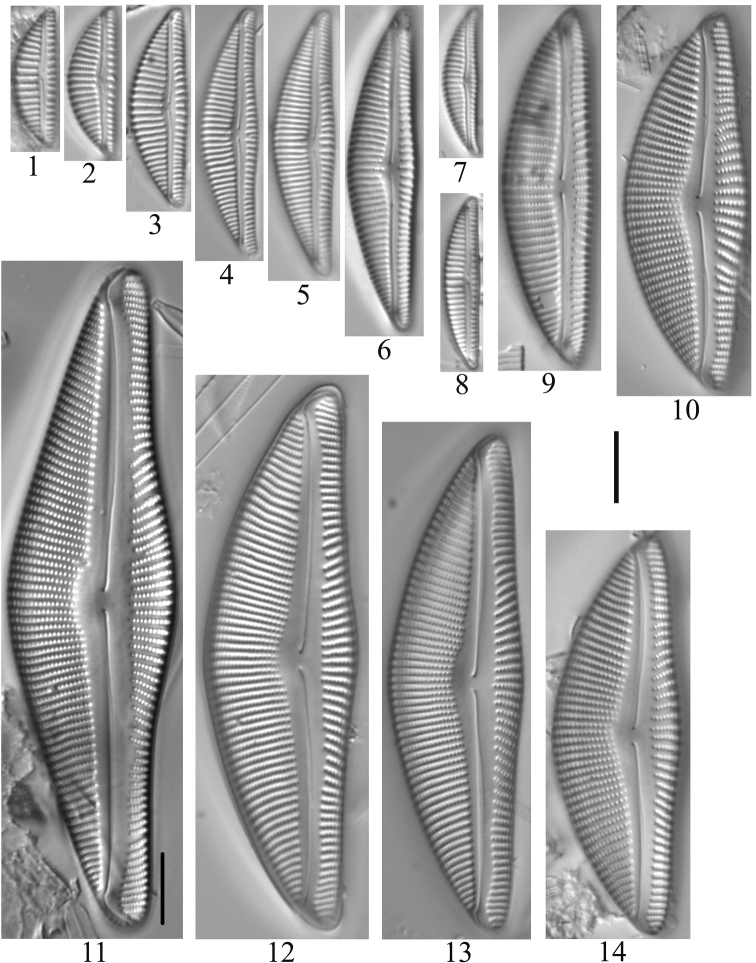
Haida Gwaii. **1–6** *Encyonema silesiacum* (5062, 5065, 5066) **7** *E. fogedii* [*E. silesiacum* var. PH] (5066) **8** *E. minutiforme* [*E. silesiacum* var. PH] (5067) **9** *E. vulgare* (5067) **10–14** *E. latum* (5065). Scale bars: 10 µm.

**Plate 30.**
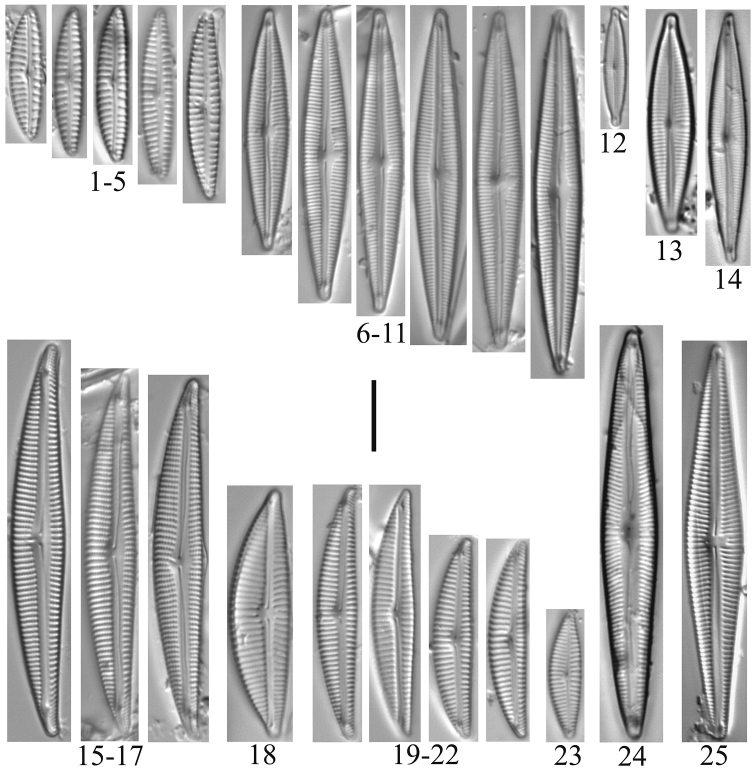
Haida Gwaii. **1–5** *Encyonema perpusillum* (5064) **6–11, 13** *Encyonopsis cesatii* (5062, 5065) **12** *Encyonopsis subminuta* [*E. microcephala* var. PH] (5065) **14** *Encyonopsis stafsholtii* (5063) **15–17** *Encyonema pergracile* (5065) **18** *Encyonema hebridicum* (5064) **19–22** *Encyonema neogracile* (5063, 5067) **23** *Encyonema* sp. (5064) **24** *Kurtkrammeria treinishii* (5065) **25** *Kurtkrammeria lacusglacialis* (5064). Scale bar: 10 µm.

**Plate 31.**
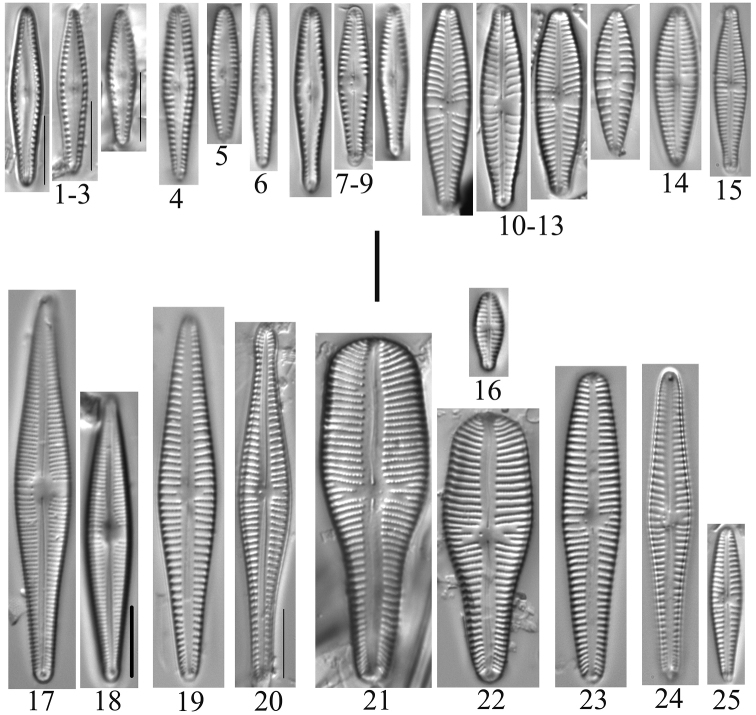
Haida Gwaii. **1–3** *Gomphonema louisiananum* (5065) **4** *G.* sp. [cf. *G. pumilum*] [cf. *G. obstipum* PH] (5065) **5** *G. kobayasii* (5062) **6** *G.* sp. [cf. *G. minusculum*] (5062) **7–9** *G.* sp. (5063, 5067) **10–13** *G. micropus* (5062, 5067) **14** *G.* sp. [cf. *G. citera*] (6890) **15** *G. exilissimum* (5067) **16** *G.* sp. (6890) **17, 18** *G.* sp. (5063) **19** *G. gracile* (5067) **20** *G. procerum* (5065) **21, 22** *G. laticollum* [*G. italicum* sensu stricto PH] (5065) **23** *G. clavatum* (5067) **24, 25** *G. duplipunctatum* (5063, 5066). Scale bars: 10 µm.

**Plate 32.**
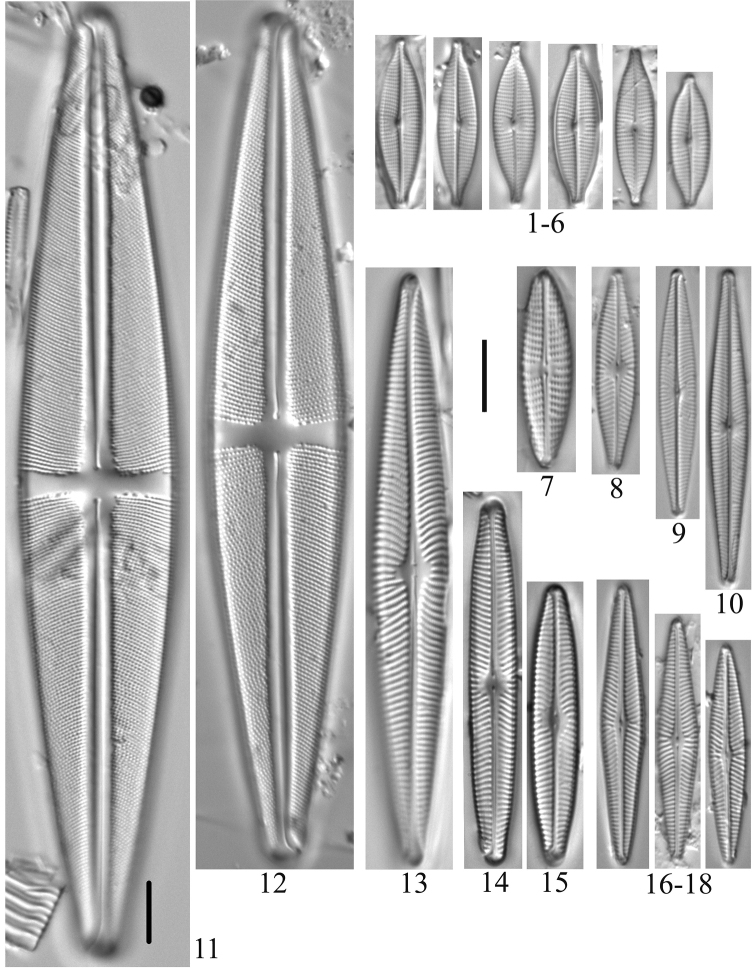
Haida Gwaii. **1–6** *Navicula gregaria* (6888, 6889, 6890) **7** *Navicula eidrigiana* (6890) **8** *Navicula exilis* (6889) **9, 10** *Navicula leptostriata* (5064) **11, 12** *Stauroneis heinii* (5065, 6889) **13** *Navicula radiosa* (5065) **14, 15** *Navicula angusta* (5063) **16–18** *Navicula cryptotenella* (5063, 5065). Scale bars: 10 µm.

**Plate 33.**
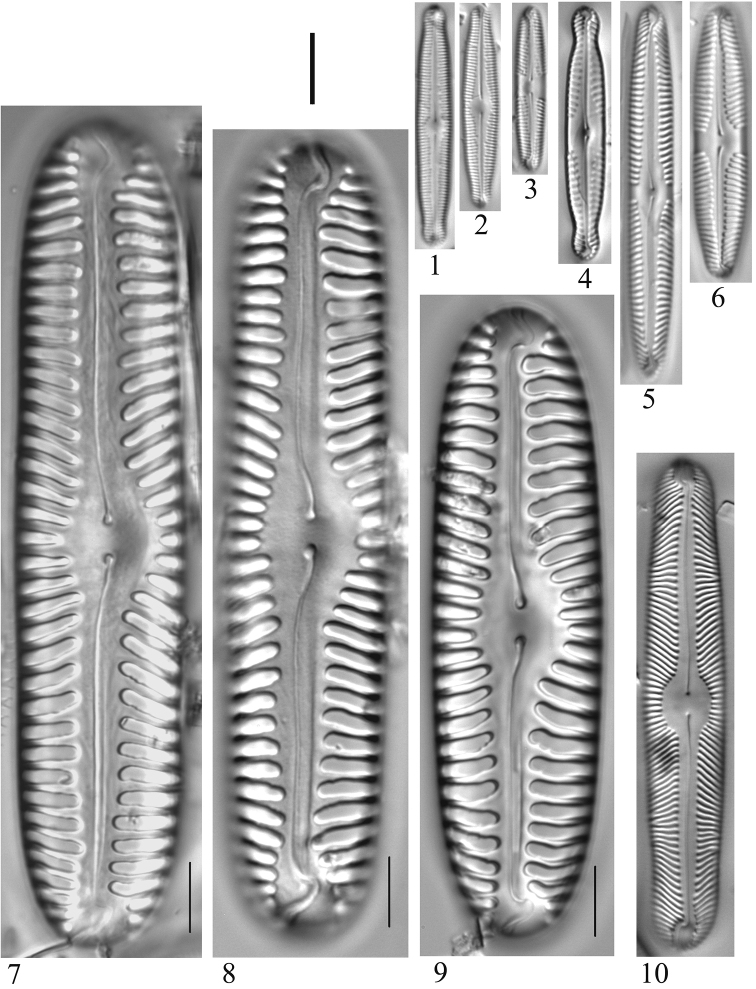
Haida Gwaii. **1, 2** *Caloneis tenuis* (5065, 5067) **3** cf. *Caloneis bacillum* (6890) **4** *Pinnularia subcapitata* (6890) **5** Pinnularia microstauron var. angusta [*P. submicrostauron* or P. microstauron var. rostrata PH] (5064) **6** *Pinnularia microstauron* (5064) **7–9** *Pinnularia lata* (5064) **10** *Pinnularia decrescens* [P. decrescens var. rhombarea PH] (5067). Scale bars: 10 µm.

**Plate 34.**
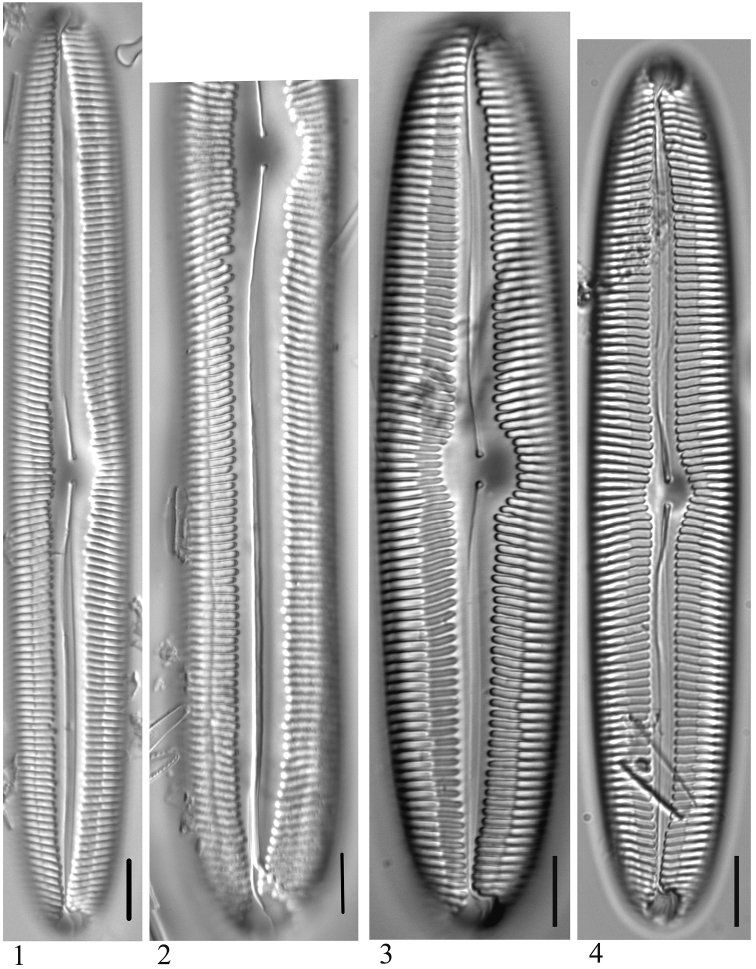
Haida Gwaii. **1, 2** *Pinnularia transversa* [Fig. **1** *P. viridis* morphotype PH] (5064) **3** *P. mesogongyla* [*P. gigas* PH] (5065) **4** *P. viridiformis* [*P. viridis* PH] (5064). Scale bars: 10 µm.

**Plate 35.**
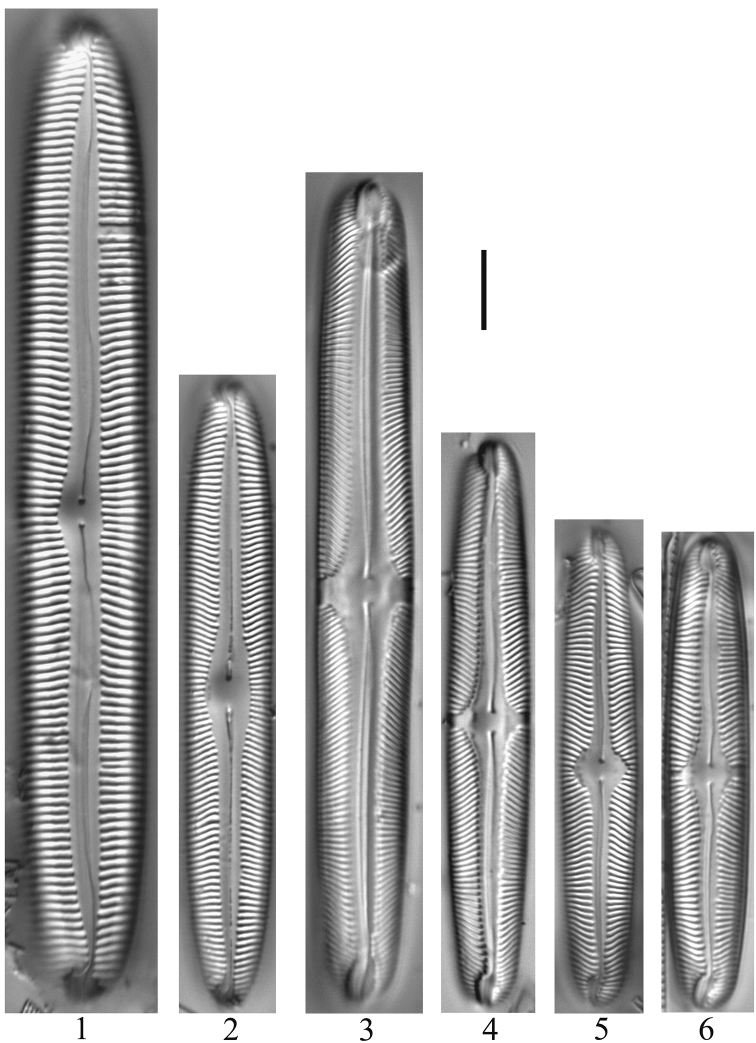
Haida Gwaii. **1** *Pinnularia neomajor* (5065) **2** *P. rupestris* (5064) **3–6** *P. stomatophora* (5062, 5065, 5067). Scale bar: 10 µm.

**Plate 36.**
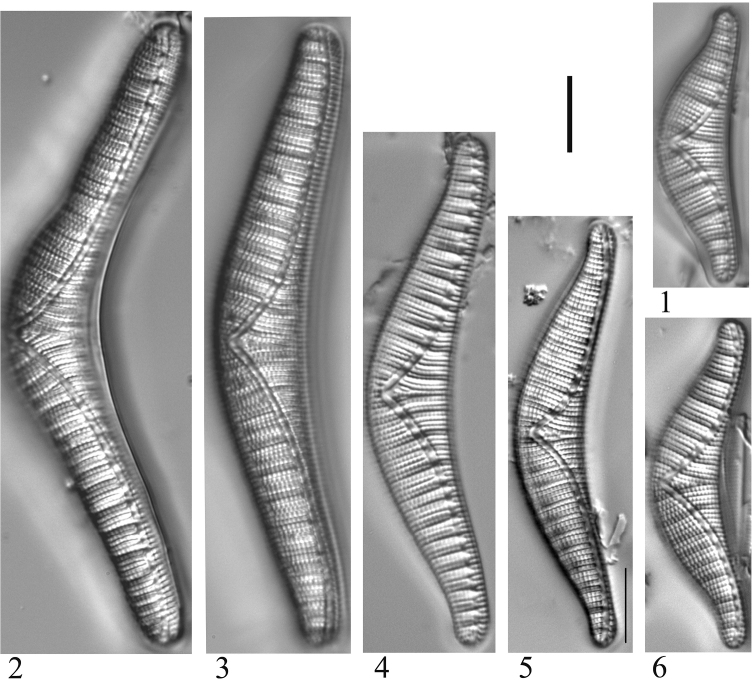
Haida Gwaii. **1–6** *Epithemia smithii* (5065). Scale bars: 10 µm.

**Plate 37.**
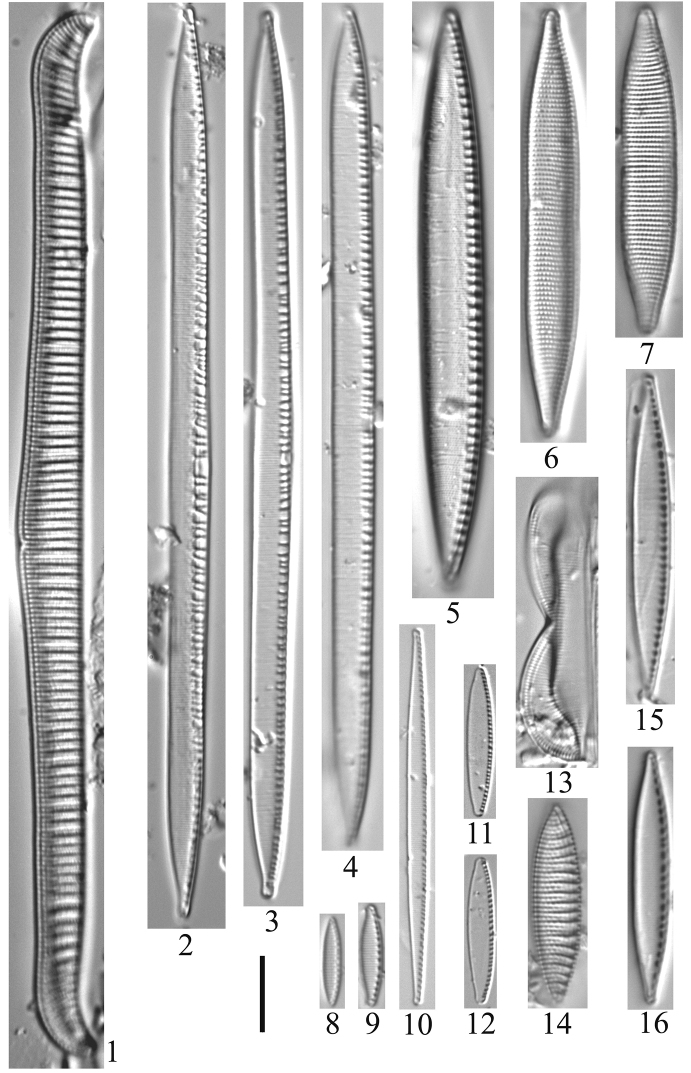
Haida Gwaii. **1** *Rhopalodia gibba* (5065) **2–4** *Nitzschia linearis* (6889) **5** *Nitzschia recta* (6890) **6, 7** *Nitzschia angustata* (5065) **8** *Nitzschia liebetruthii* (6890) **9** *Nitzschia* sp. [cf. *N. microcephala*] [cf. *N. fonticola* PH] (5064) **10** *Nitzschia gracilis* (5064) **11, 12** *Nitzschia pusilla* (6889) **13** *Entomoneis paludosa* (6890) **14** *Denticula kuetzingii* (5065) **15, 16** *Nitzschia pseudofonticola* (5062, 5065). Scale bar: 10 µm.

**Plate 38.**
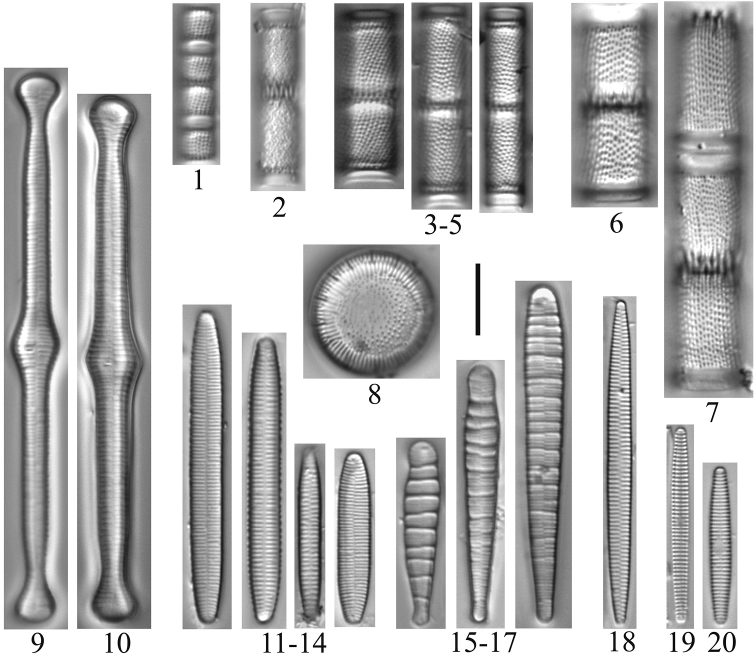
Clearwater. **1** *Aulacoseira alpigena* (6273) **2** *Aulacoseira subarctica* (?) (6276) **3–5** *Aulacoseira ambigua* (6275, 6276) **6, 7** *Aulacoseira italica* (6277) **8** *Lindavia praetermissa* (6277) **9, 10** *Tabellaria flocculosa* (6279) **11–14** *Fragilariforma nitzschioides* (6281) **15–17** *Meridion circulare* (6279, 6281) **18** *Fragilaria* sp. [*Ulnaria* sp. PH] (6281) **19, 20** *Synedra famelica* (?) (6280). Scale bar: 10 µm.

**Plate 39.**
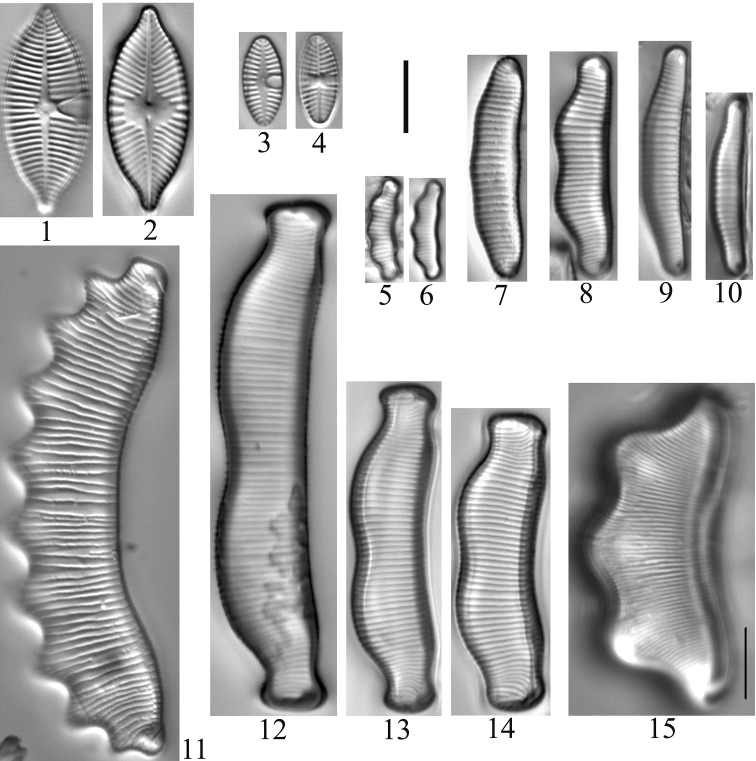
Clearwater. **1, 2** *Planothidium apiculatum* (6277) **3, 4** *Planothidium* sp. (6276) **5, 6** *Eunotia paratridentula* (6279) **7** *Eunotia* sp. (6276) **8** *Eunotia circumborealis* (6279) **9, 10** *Eunotia minor* (6277, 6279) **11** *Eunotia serra* (6276) **12–14** *Eunotia bidens* (6276, 6279) **15** *Eunotia triodon* (6276). Scale bar: 10 µm.

**Plate 40.**
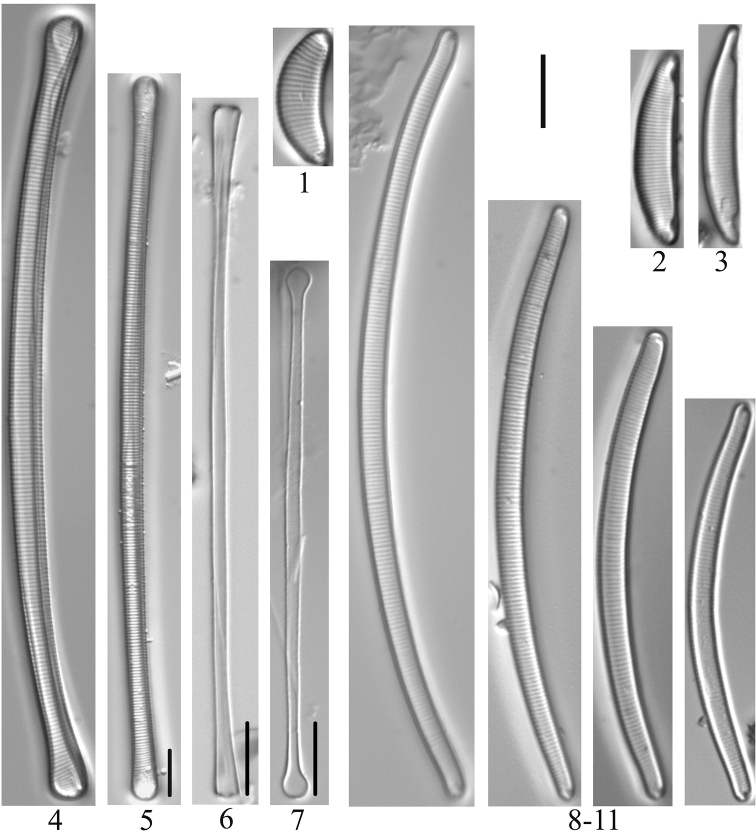
Clearwater. **1** cf. *Eunotia faba* [PH] (6279) **2, 3** *E. incisa* (6275, 6277) **4** *E. flexuosa* (6276) **5** *E. eurycephala* [*E. flexuosa* PH] (6276) **6** *E.* sp. (6277) **7** *E.* sp. (6276) **8–11** *E. ambivalens* (6276, 6278). Scale bars: 10 µm.

**Plate 41.**
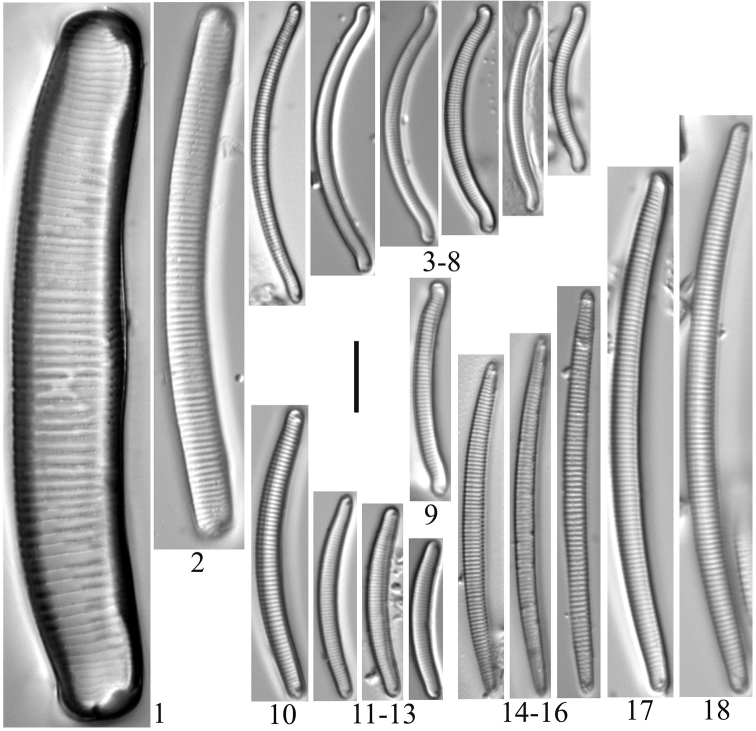
Clearwater. **1** *Eunotia* sp. [cf. *E. dorofeyukae* PH] (6279) **2** *E. julma* (6276) **3–8** *E. nymanniana* [*E. elegans* PH] (6274, 6276, 6279) **9** *E.* sp. [cf. *E. nymanniana*] (6277) **10** *E. monnieri* [*E.* sp. PH] (6279) **11–13** *E. mucophila* (6276) **14–16** *E.* sp. [*E. monnieri* PH] (6276, 6277) **17, 18** *E. ambivalens* [*E. bilunaris* PH] (6279). Scale bar: 10 µm.

**Plate 42.**
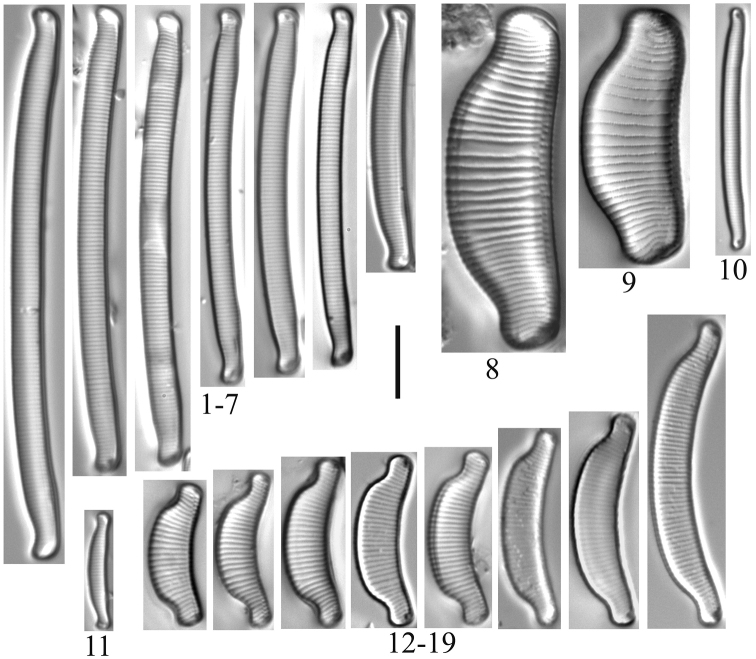
Clearwater. **1–7** *Eunotia superpaludosa* (6274, 6276) **8, 9** *E. praerupta* (6279) **10** *E. groenlandica* [*E. pseudogroenlandica* PH] (6274) **11** *E. fallax* [*E. paludosa* PH] (6279) **12–19** *E. ursamaioris* (6278, 6279). Scale bar: 10 µm.

**Plate 43.**
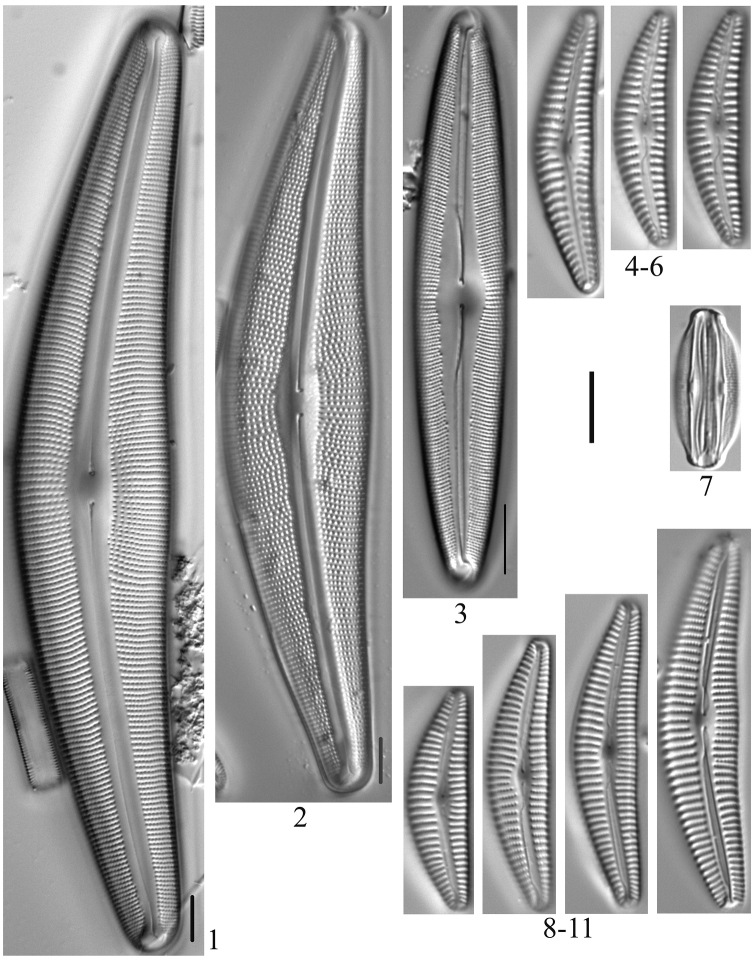
Clearwater. **1, 2** *Cymbella aspera* (6277) **3** *Cymbella* (*Cymbopleura*?) *naviculacea* [syn. *Encyonopsis grunowii*] (6277) **4–6** *Cymbella* sp. (6280) **7** *Halamphora coraensis* [*H. obscura* PH] (6280) **8–11** *Cymbella hantzschiana* (6280). Scale bars: 10 µm (specimens in images 1 and 2 are at a different scale than the others).

**Plate 44.**
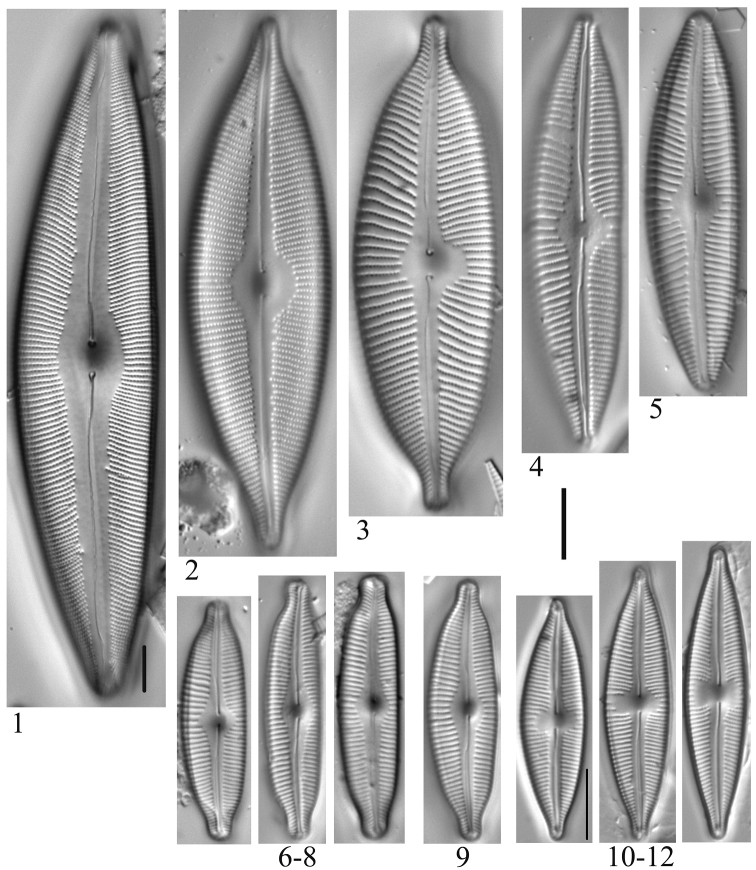
Clearwater. **1** *Cymbopleura crassipunctata* (6277) **2** *C. apiculata* (6277) **3** *C. subcuspidata* (6277) **4, 5** *C. rainierensis* [= *Cymbella gondwana*?] (6277) **6–8** *C. fluminea* (6277) **9** *C. naviculiformis* (6277) **10–12** *C. stauroneiformis* (6277, 6279). Scale bars: 10 µm (specimen in image 1 is at a different scale than the others).

**Plate 45.**
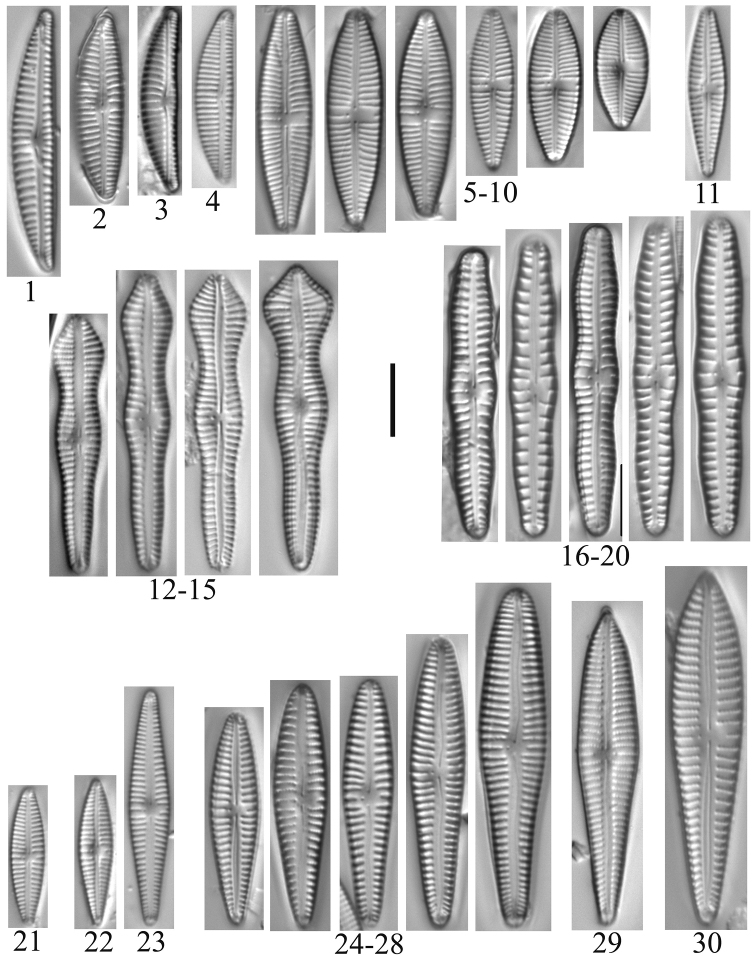
Clearwater. **1** *Encyonema neogracile* (6277) **2** *Encyonema hebridicum* (6273) **3** *Encyonema fogedii* (6279) **4** *Encyonema minutum* (6277) **5–10** *Gomphonema micropus* (6281) **11** *Gomphonema duplipunctatum* (6277) **12–15** *Gomphonema brebissonii* (6276, 6277) **16–20** *Gomphonema distans* (6279) **21** *Gomphonema parvulum* (6277) **22, 23** *Gomphonema auritum* (6276, 6277) **24–28** *Gomphonema subclavatum* (6280) **29** *Gomphonema insigniforme* (6276) **30** *Gomphonema* sp. [cf. *G. bukycanyonum* PH] (6277). Scale bars: 10 µm.

**Plate 46.**
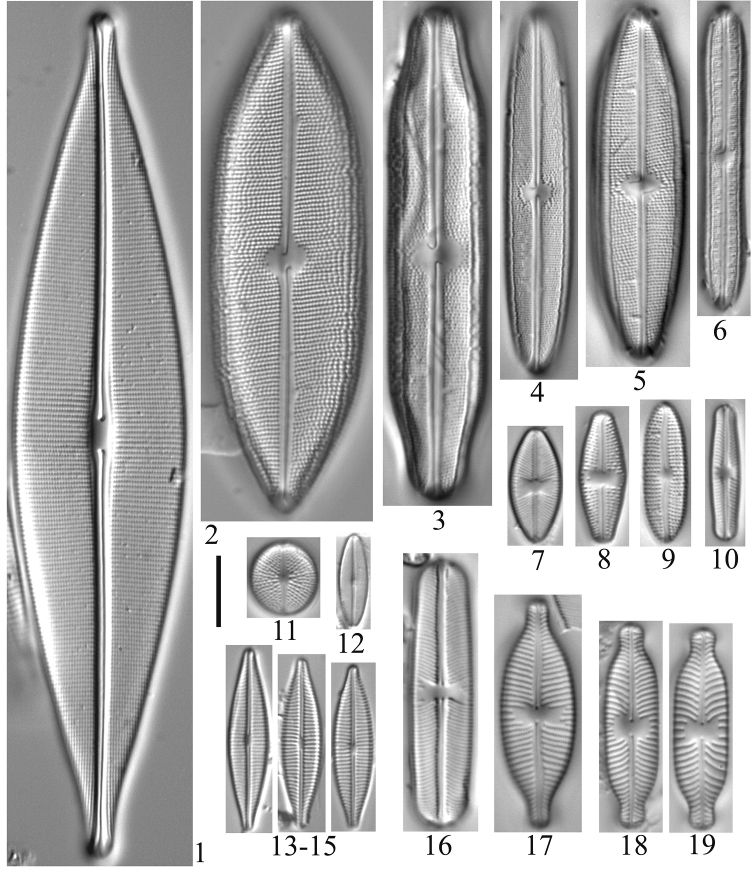
Clearwater. **1** *Craticula cuspidata* (6277) **2** *Neidium amphigomphus* (6276) **3** *Neidium* sp. (6277) **4** *Neidium* sp. (6276) **5** *Neidium ampliatum* [cf. *N. affine* PH] (6277) **6** *Neidium bisulcatum* (6279) **7** *Sellaphora* sp. (6279) **8** *Luticola mutica* (6281) **9** *Boreozonacola olympica* (6277) **10** *Sellaphora* (?) sp. (6281) **11** *Cavinula pseudoscutiformis* (6278) **12** *Adlafia minuscula*? (6277) **13–15** *Craticula buderi* (6280) **16** *Sellaphora rectangularis* (6277) **17** *Placoneis explanata* (6277) **18, 19** *Placoneis elginensis* (6277). Scale bar: 10 µm.

**Plate 47.**
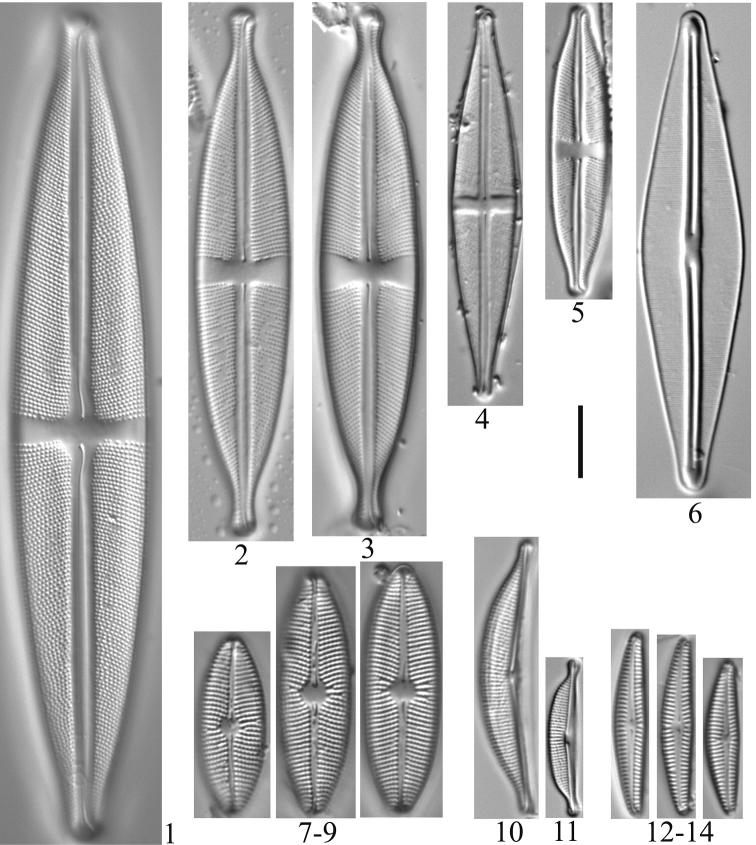
Clearwater. **1** *Stauroneis gracilis* (6276) **2, 3** *Stauroneis amphicephala* (6277) **4** *Stauroneis neohyalina* (6277) **5** *Stauroneis acidoclinata* (6279) **6** *Frustulia saxonica* (6276) **7–9** *Mastogloia elliptica* (6280) **10** *Halamphora* sp., **11** *Halamphora borealis* (6280) **12–14** *Navicymbula pusilla* [*Encyonema* sp.? PH] (6280). Scale bar: 10 µm.

**Plate 48.**
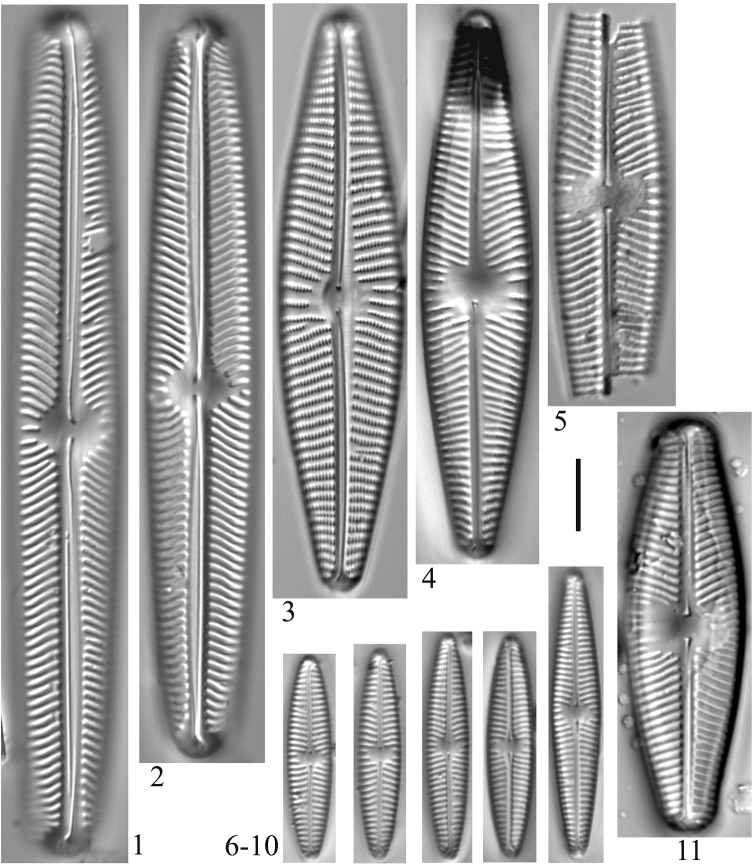
Clearwater. **1, 2** *Navicula oblonga* (6280) **3** *N. peregrina* (6280) **4, 5** *N. kefvingensis* (6280) **6–10** *N. libonensis* (6280) **11** *N. aurora* (6277). Scale bar: 10 µm.

**Plate 49.**
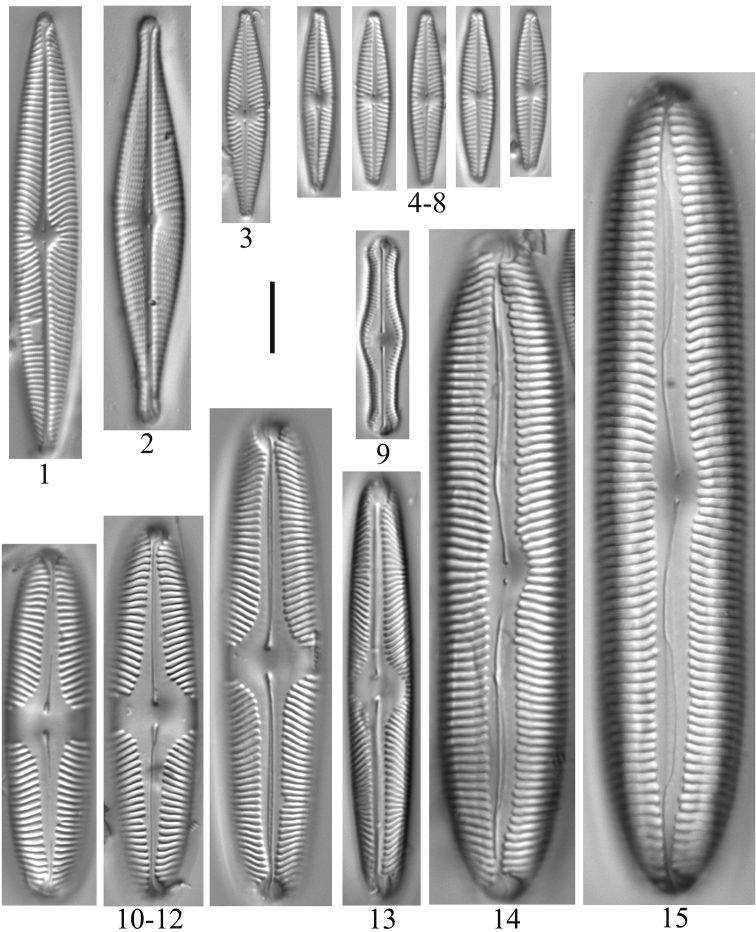
Clearwater. **1** *Navicula radiosa* (6277) **2** *Navicula rhynchocephala* (6277) **3** *Navicula cryptocephala* [cf. *N. cryptocephala* PH] (6277) **4–8** *Navicula trilatera* (6280) **9** *Pinnularia ignobilis* [cf. *Chamaepinnularia krookii* PH] (6280) **10–12** *Pinnularia brebissonii* (6280) **13** *Pinnularia lenticula* [cf. *P. lenticula* PH] (6278) **14, 15** *Pinnularia viridiformis* (6280). Scale bar: 10 µm.

**Plate 50.**
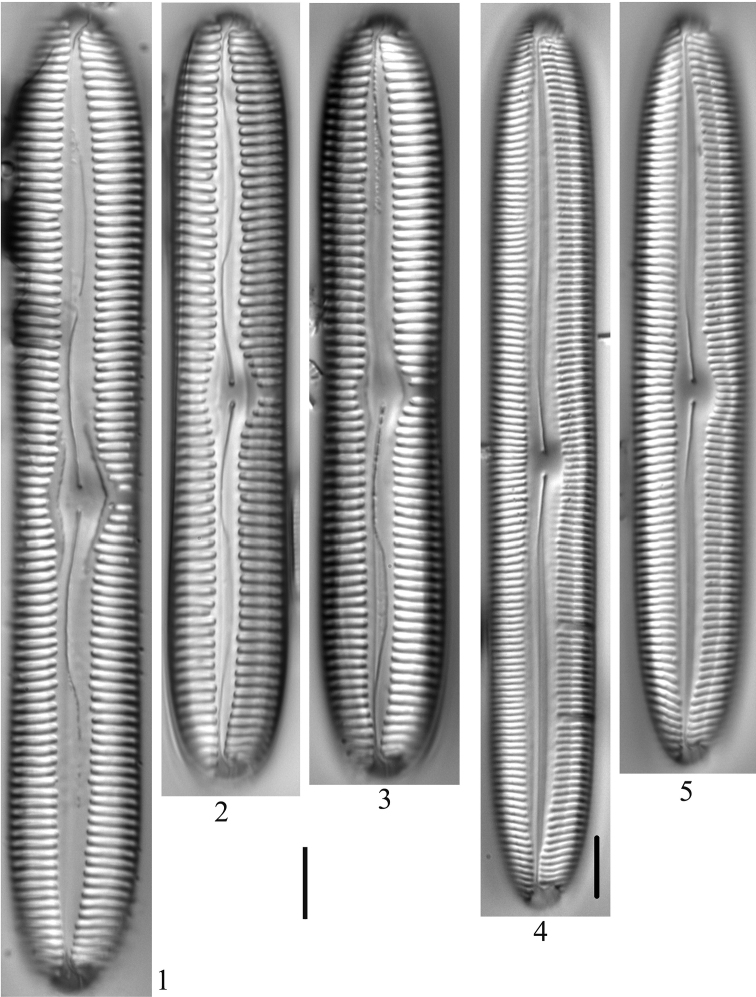
Clearwater. **1–3** *Pinnularia* sp. [cf. *P. genkalii*] (6279) **4, 5** *P. neomajor* (6276, 6277). Scale bars: 10 µm (specimen in image 4 is at a different scale than the others).

**Plate 51.**
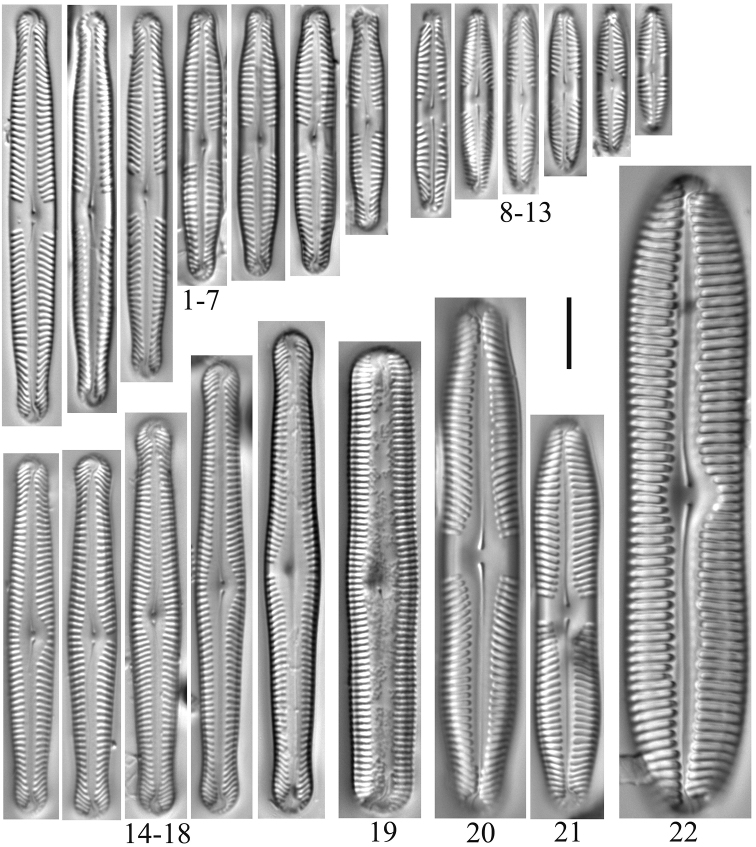
Clearwater. **1–7** Pinnularia subcapitata var. elongata (6275, 6279) **8–13** *P. obscura* (6279) **14–18** *P. gibbiformis* (6276) **19** *P. acrosphaeria* (6277) **20, 21** *P.* sp. [*P. biceps* sensu lato PH] (6276, 6278) **22** *P.* sp. [*P. viridiformis* sensu lato PH] (6279). Scale bar: 10 µm.

**Plate 52.**
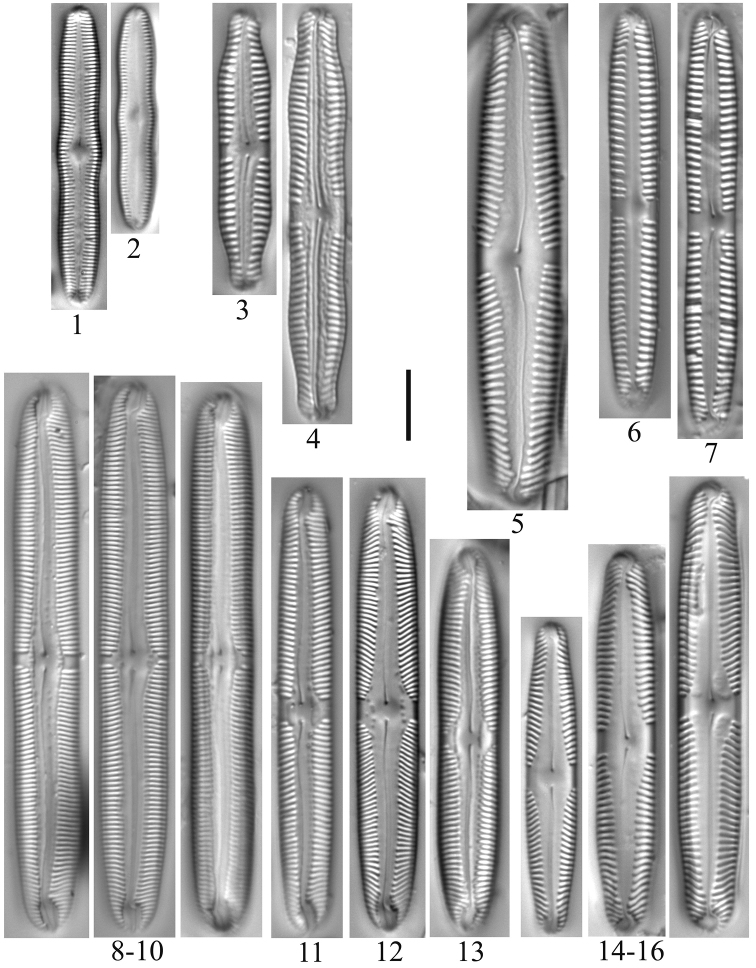
Clearwater. **1, 2** *Pinnularia* sp. [cf. *P. subpulchra*] [cf. *Caloneis arctica* PH] (6277) **3, 4** *P. nodosa* (6279) **5** *P.* sp. [cf. *P. sudetica* or *persudetica* PH] (6279) **6, 7** *P. ivaloensis* (6279) **8–10** *P. spitsbergensis* (6276, 6279) **11** *P.* sp. [cf. *P. spitsbergensis*] (6276) **12, 13** *P. stomatophora* (6277) **14–16** *P.* sp. [cf. P. graciloides var. triundulata] [cf. *P. subgibba* PH] (6277). Scale bar: 10 µm.

**Plate 53.**
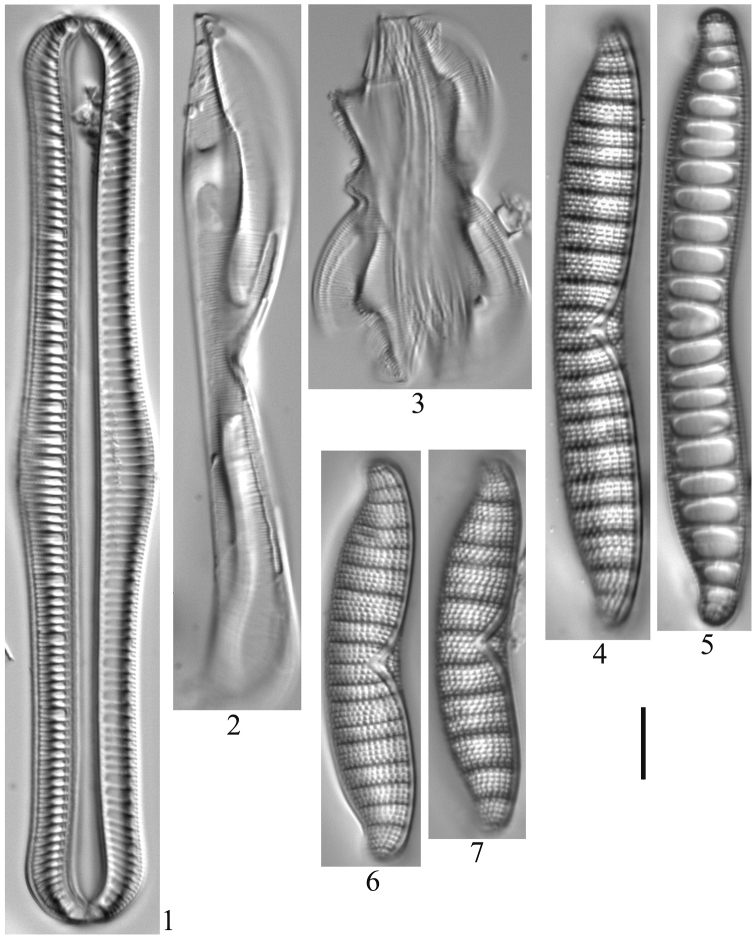
Clearwater. **1** *Rhopalodia gibba* (6277) **2, 3** *Entomoneis paludosa* (6280) **4–7** *Epithemia argus* (6277). Images **4** and **5** are the same specimen at high and low focus. Scale bar: 10 µm.

**Plate 54.**
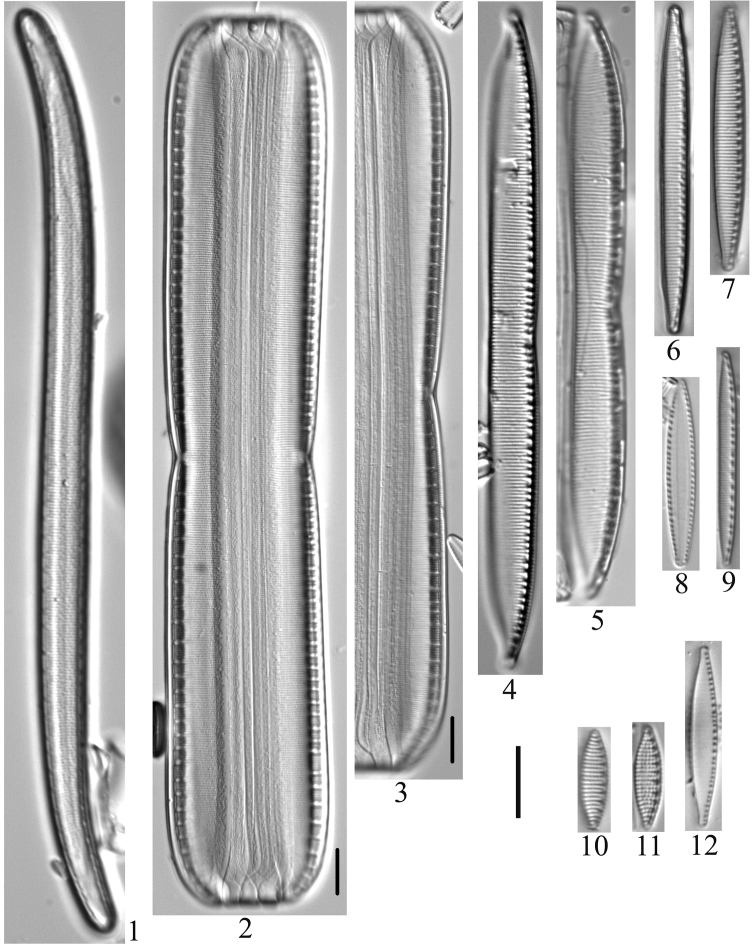
Clearwater. **1** *Stenopterobia curvula* (6276) **2, 3** *Nitzschia kittlii* (6280) **4** *Nitzschia linearis* (6280) **5** *Nitzschia commutata* (6280) **6, 7** *Nitzschia radicula* [*N. fossilis* PH] (6280) **8** *Nitzschia perspicua* [cf. *N. bergii* PH] (6280) **9** *Nitzschia perminuta* (6277) **10, 11** *Nitzschia amphibia* (6280) **12** *Nitzschia palea* (6277). Scale bars: 10 µm (images 2 and 3 are at different scales than the others).

**Plate 55.**
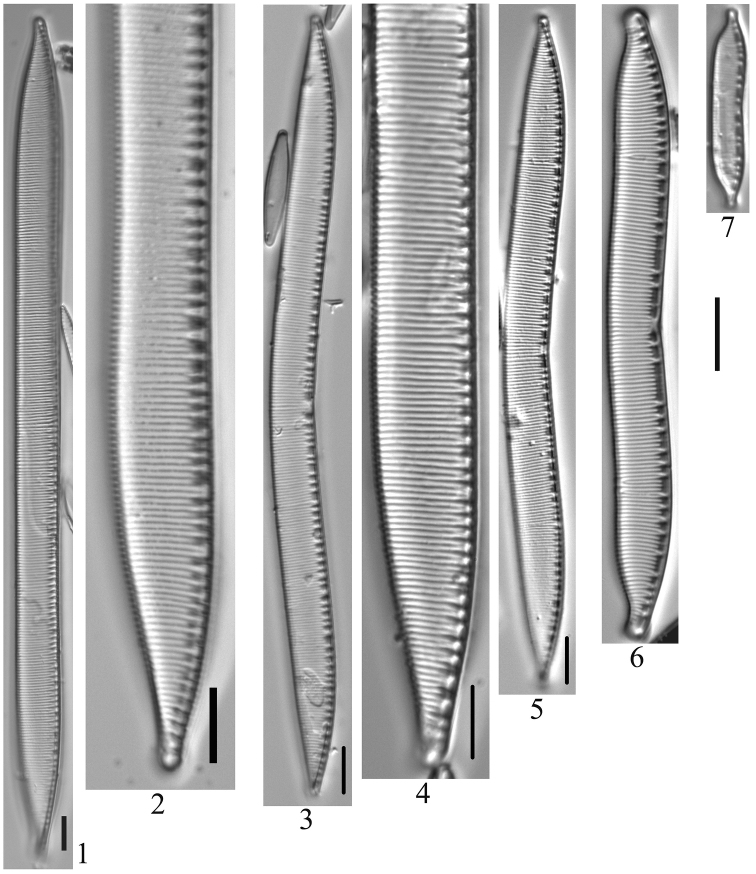
Clearwater. **1, 2** *Hantzschia elongata* (6280) **3–5** *H. vivacior* (6280) **6** *H.* sp. (6279) **7** *H. amphioxys* [*H. amphioxys* sensu lato PH] (6278). Scale bars: 10 µm (images **1, 3** and **5** are at different scales than the others).

**Plate 56.**
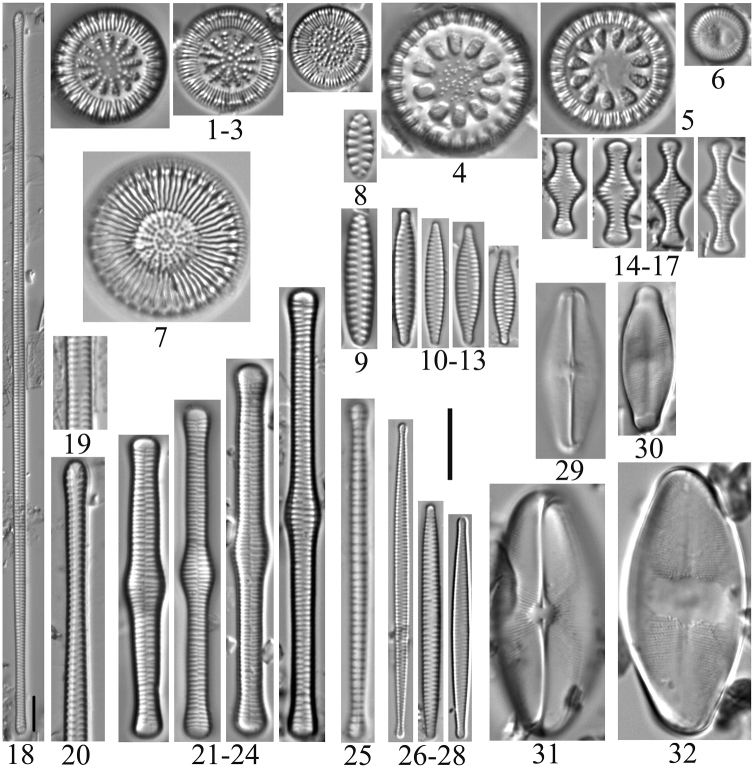
Coppermine. **1–3** *Lindavia radiosa* [*L. antiqua* PH] (6830, 6831) **4, 5** *Lindavia antiqua* (6830, 6831) **6** *Lindavia michiganiana* (6832) **7** *Lindavia intermedia* (6830) **8, 9** *Staurosirella* sp. [cf. *S. pinnata*] (6832) **10–13** *Fragilaria* spp. [cf. *F. vaucheriae*] [*F. capucina* PH] (6832, 6833) **14–17** *Tabellaria flocculosa* (6830, 6831, 6832) **18–20** *Ulnaria* sp. (6832) (all three images are of the same specimen; image **18** is at a different scale) **21–24** *Tabellaria fenestrata* [T. flocculosa var. linearis PH] (6828, 6830, 6831, 6832) **25** *Diatoma tenuis* (6832) **26–28** *Fragilaria* spp. [cf. *F. capucina*] (6832, 6833) **29, 30** *Eucocconeis laevis* (6826, 6830, 6833) **31, 32** *Eucocconeis flexella* (6827, 6831, 6833). Scale bars: 10 µm.

**Plate 57.**
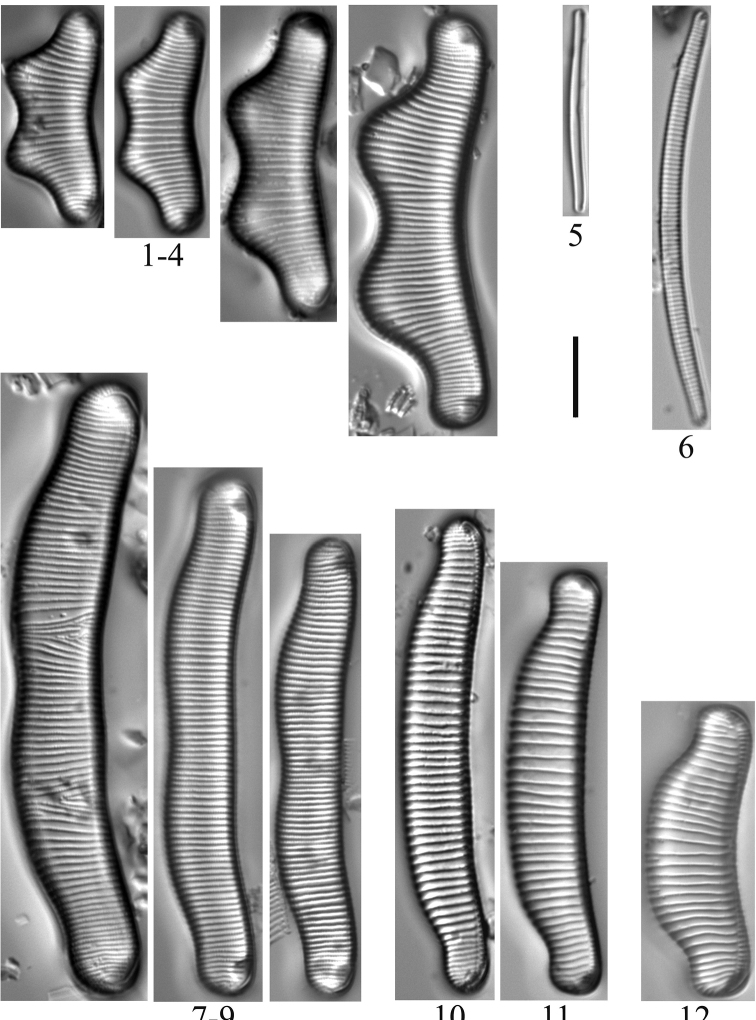
Coppermine. **1–4** *Eunotia islandica* (6828) **5** *E.* sp. (6833) **6** *E. mucophila* (6833) **7–9** *E. altimontana* (6828, 6830, 6831) **10, 11** *E. arcus* (6833) **12** *E. ursamaioris* [*E. praerupta* PH] (6824). Scale bar: 10 µm.

**Plate 58.**
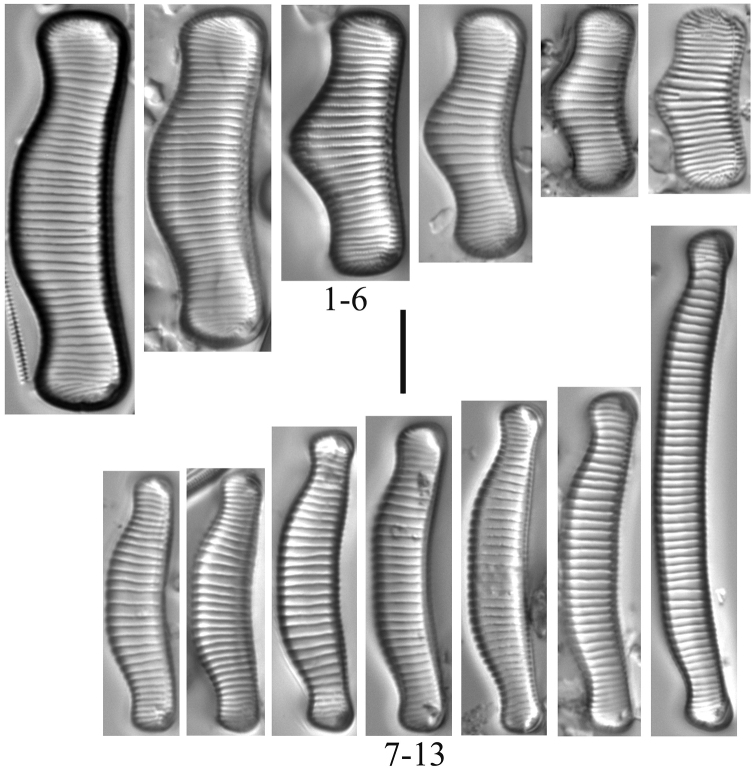
Coppermine. **1–6** *Eunotia excelsa* (6826, 6828) **7–13** *E. arcubus* [*E. arcus* PH (small forms only)] (6831, 6833). Scale bar: 10 µm.

**Plate 59.**
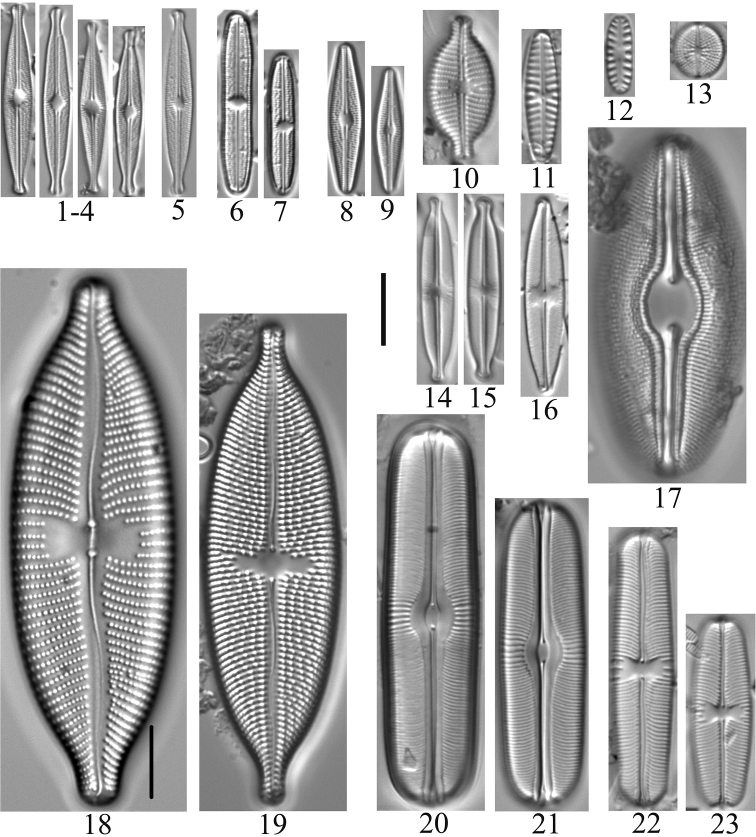
Coppermine. **1–4** *Brachysira* sp. [cf. *B. microcephala*] (6830) **5** *Brachysira* sp. [cf. *B. microcephala* (6827) **6, 7** *Brachysira zellensis* (6830) **8, 9** *Brachysira* sp. [*B. microcephala* form 1 PH] (6830) **10** *Placoneis* (?) sp. (6832) **11** *Hippodonta hungarica* (6832) **12** *Hygroptera balfouriana* (6827) **13** *Cavinula pseudoscutiformis* (6832) **14, 15** *Kobayasiella micropunctata* [cf. *K. subtilissima* PH] (6827, 6830, 6831) **16** *Kobayasiella jaagii* (6830) **17** *Diploneis krammeri* (6833) **18** *Placoneis amphibola* (6828) **19** *Aneumastus tusculus* (6830, 6831) **20, 21** *Sellaphora alastos* (6832) **22, 23** *Sellaphora* sp. [*S. rectangularis* PH] (6832). Scale bar: 10 µm.

**Plate 60.**
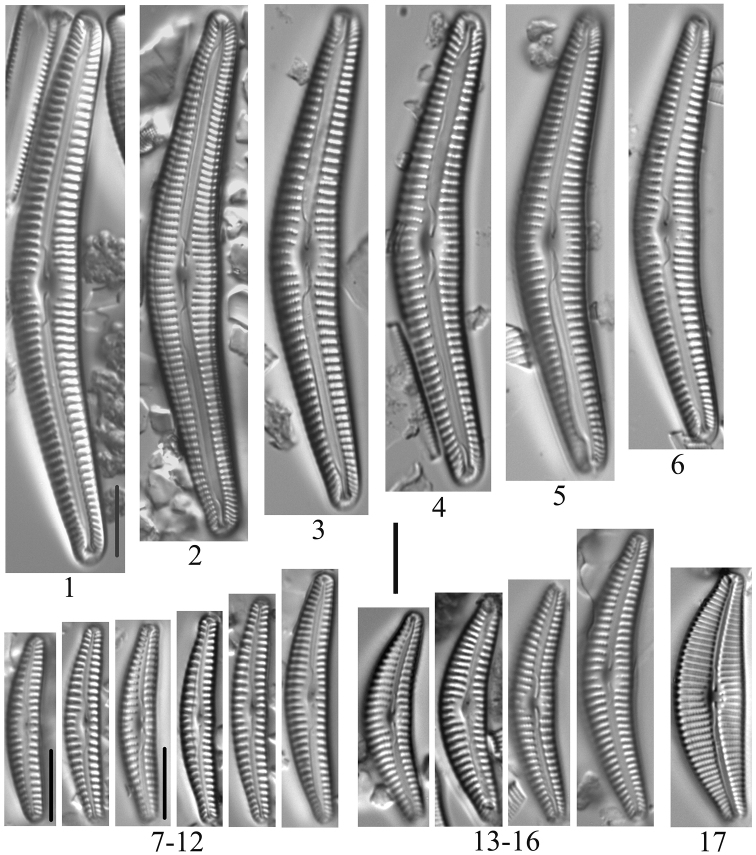
Coppermine. **1–6** *Cymbella* sp. [cf. *C. cleve-eulerae*] [*C. neocistula* var. PH] (6830, 6831, 6833) **7–12** *C. botellus* (6826, 6829, 6830) **13–16** *C. cleve-eulerae* (6826, 6828, 6830, 6831, 6833) **17** *C. subturgidula* (6833). Scale bars: 10 µm.

**Plate 61.**
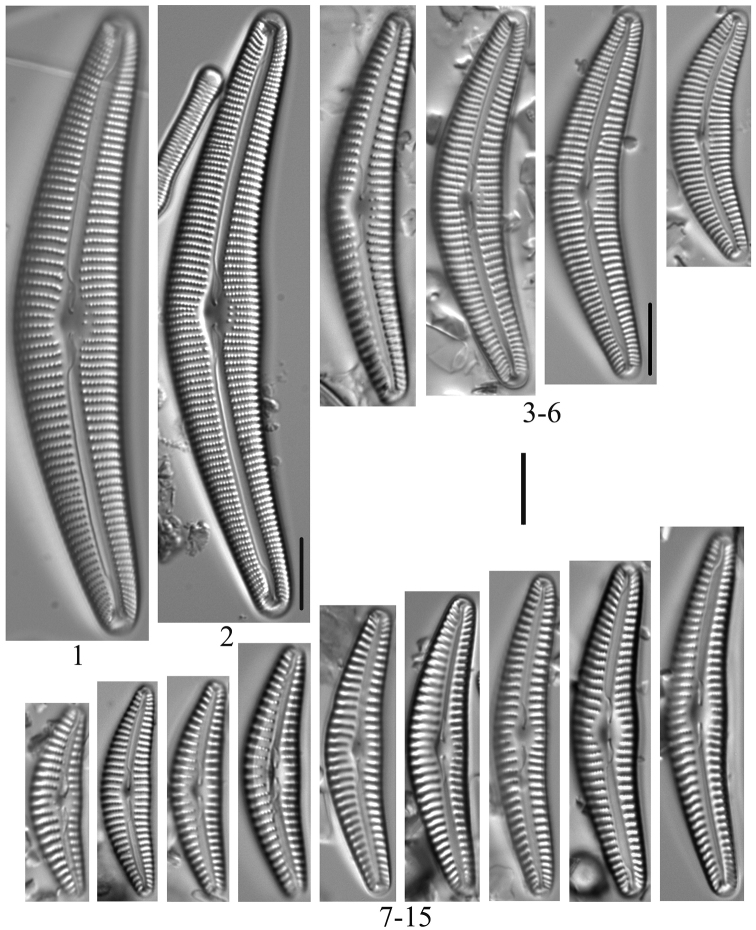
Coppermine. **1, 2** *Cymbella neocistula* (6824, 6831) **3–6** *C. krammeri* (6826, 6827, 6829, 6830, 6833) **7–15** *C. cleve-eulerae* (6826, 6828, 6830, 6831, 6833). Scale bars: 10 µm.

**Plate 62.**
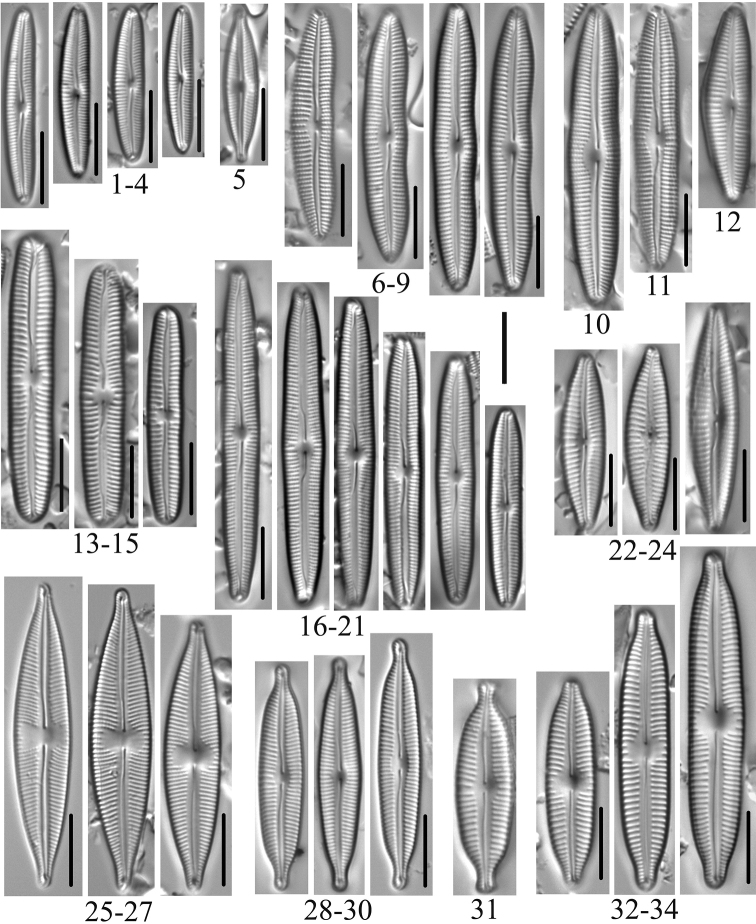
Coppermine. **1–4** *Cymbopleura tundraphila* (6830, 6833) **5** *C. geofriedii* [cf. *C. geofriedii* PH] (6830) **6–9** C. incerta var. spitsbergensis (6830, 6831, 6833) **10–12** *C. incerta* (6826, 6829, 6831) **13–15** *C. oblongata* (6828, 6830, 6833) **16–21** C. incertiformis var. linearis (6830, 6833) **22–24** *C. rupicola* (6830) **25–27** *C. stauroneiformis* (6828, 6830, 6833) **28–30** *C. angustata* (6829, 6830, 6831, 6833) **31** *C. amphicephala* (6833) **32–34** *C. hybrida* (6833). Scale bars: 10 µm.

**Plate 63.**
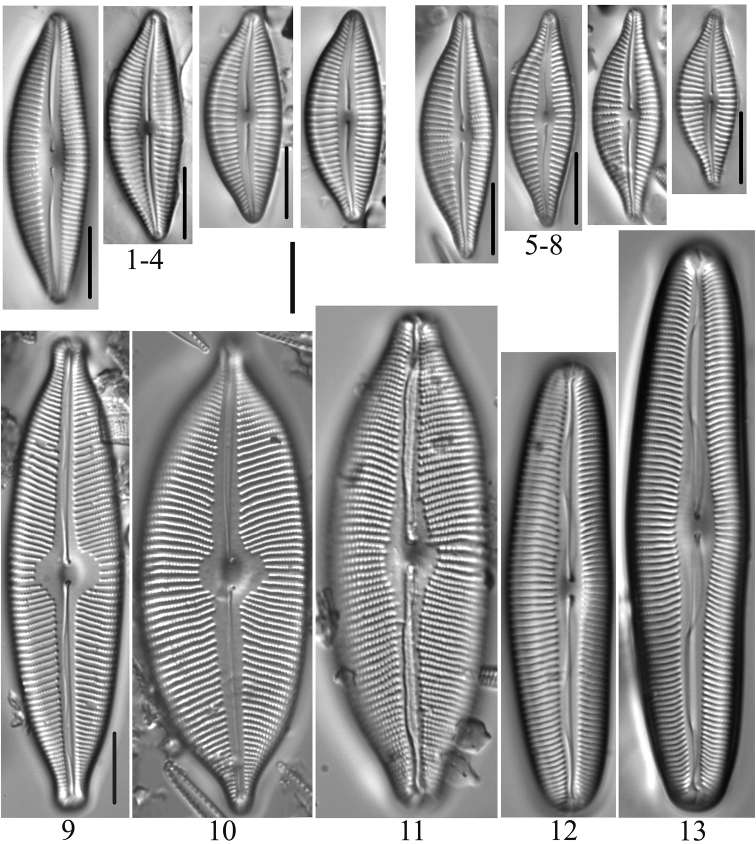
Coppermine. **1–4** *Cymbopleura heilprinensis* (6828, 6829, 6831, 6833) **5–8** *Cymbella designata* [syn. *Cymbopleura citriformis*] (6829, 6830, 6831) **9** *Cymbopleura tynnii* (6828, 6831) **10** *Cymbopleura apiculata* (6832) **11** *Cymbopleura lata* (6830) **12, 13** *Cymbopleura austriaca* (6824). Scale bars: 10 µm.

**Plate 64.**
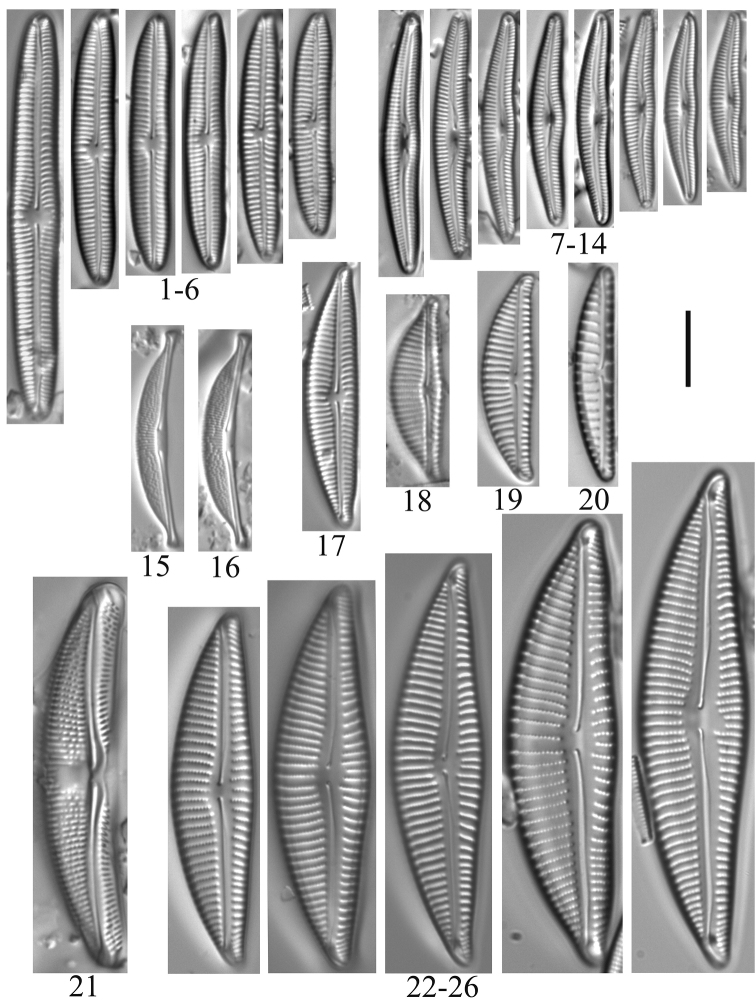
Coppermine. **1–6** *Encyonema norvegicum* (6829, 6830, 6833) **7–14** *Delicata canadensis* (6829, 6830, 6833) **15, 16** *Halamphora coraensis* (6829, 6831) **17** *Encyonema hebridicum* (6829) **18** *Encyonema ventricosum* (6832) **19** *Encyonema silesiacum* (6831) **20** *Encyonema paucistriatum* (6833) **21** *Amphora lange-bertalotii* (6826) **22–26** *Encyonema hintzii* or *Encyonema vulgare* (6827, 6830, 6831, 6832, 6833). Scale bar: 10 µm.

**Plate 65.**
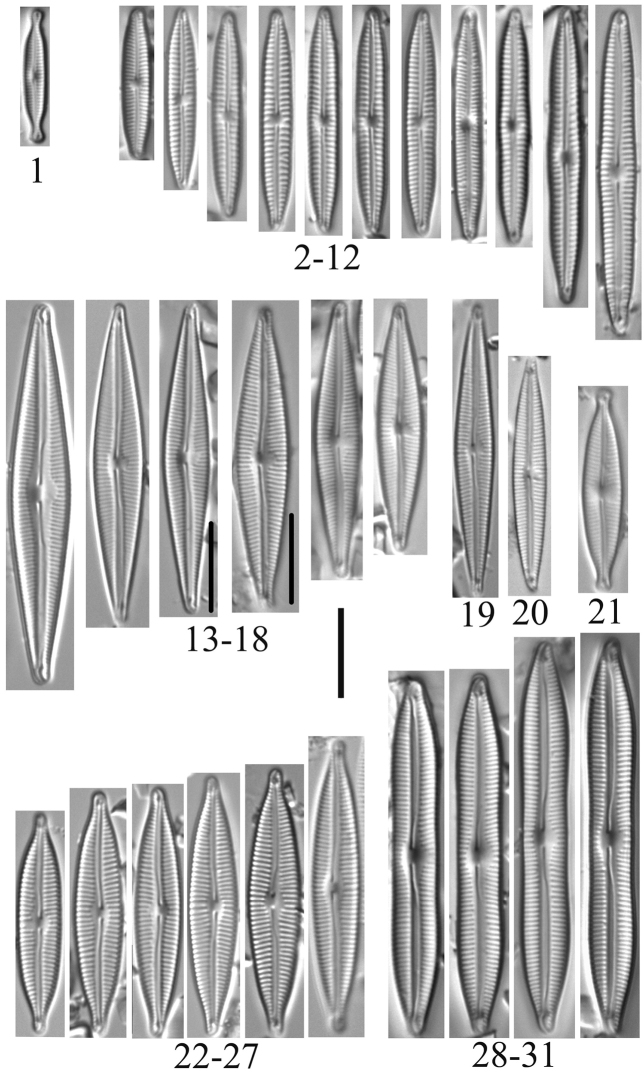
Coppermine. **1** *Encyonopsis* sp. [cf. *E. angusta*] (6830) **2–12** *E. inuitorum* (6830, 6831, 6833) **13–18** *E. stafsholtii* (6827, 6829, 6830, 6831, 6833) **19, 20** *E.* sp. [cf. *E. neerlandica*] (6831, 6833) **21** *E.* sp. [cf. *E. descripta*] (6833) **22–27** *E. cesatiformis* [*E. cesatii* sensu stricto PH] (6826, 6830, 6831, 6833) **28–31** *E. lacuscaerulei* (6829, 6830). Scale bars: 10 µm.

**Plate 66.**
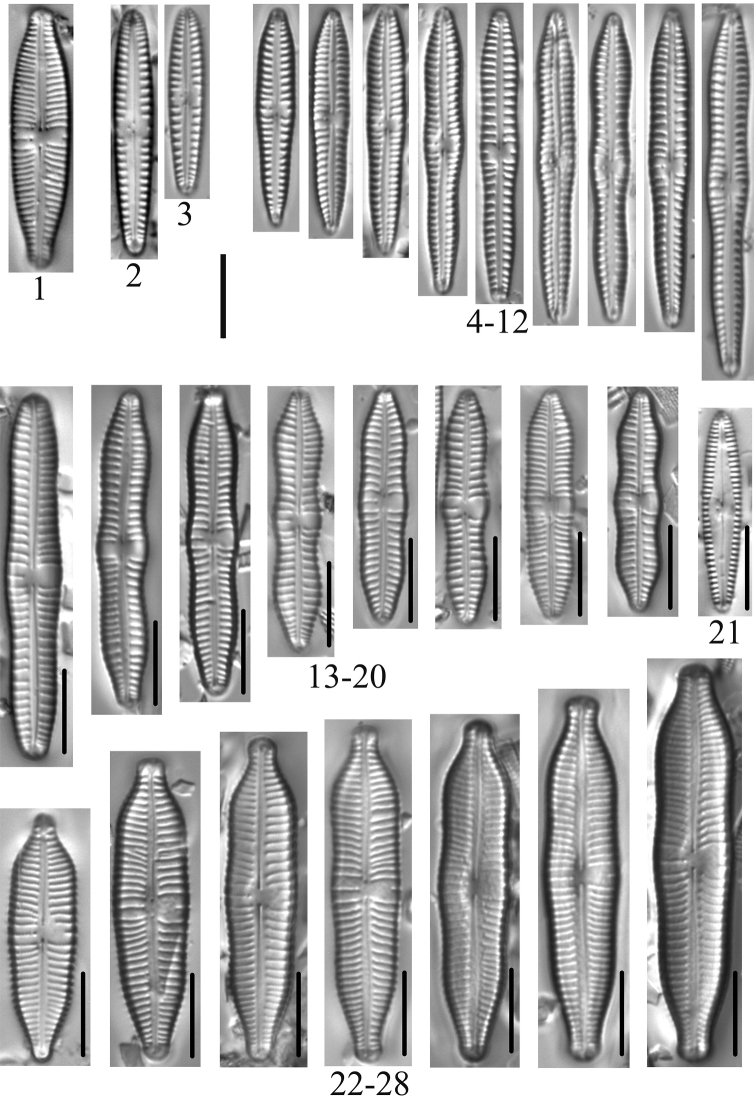
Coppermine. **1** *Gomphonema micropus* (6828) **2, 3** *G. lateripunctatum* (6826, 6833) **4–12** *G. lagerheimii* (6830, 6831, 6833) **13–20** *G. distans* (6828, 6830) **21** *G. caperatum* (6828) **22–28** *G. nathorstii* [G. angustatum var. undulatum sensu Foged PH] (6828). Scale bars: 10 µm.

**Plate 67.**
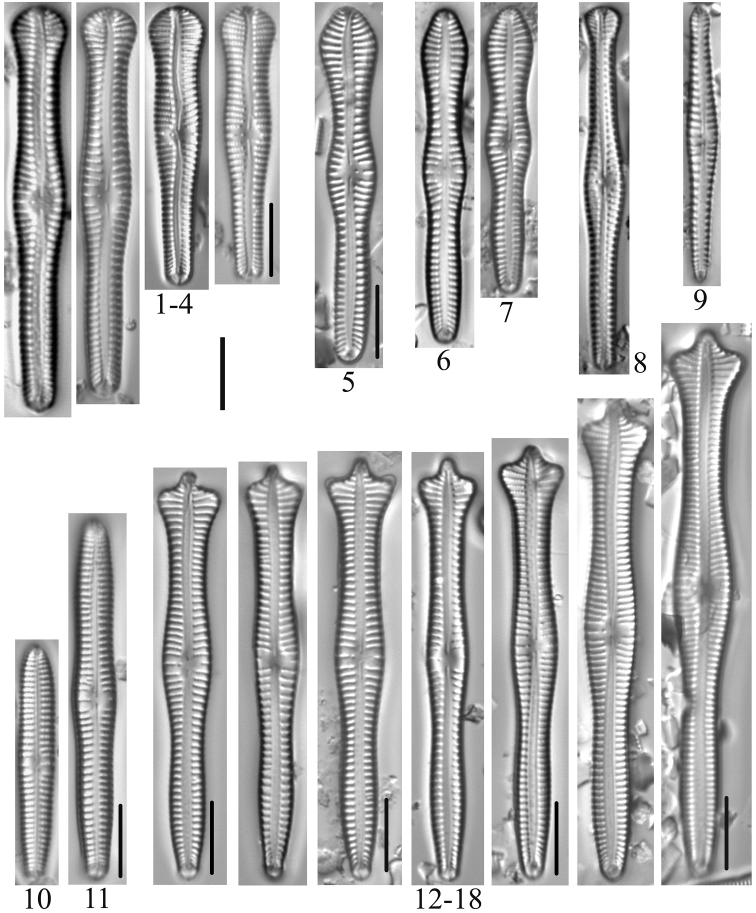
Coppermine. **1–4** *Gomphonema* sp. [cf. *G. capitatum*] (6833) **5** *G. brebissonii* (6830) **6, 7** *G.* sp. [cf. *G. interpositum*] (6832, 6833) **8** *G. subtile* (6833) **9** G. subtile var. sagitta (6833) **10, 11** *G.* sp. (6830, 6831) **12–18** *G. coronatumaceum* (6830, 6831). Scale bars: 10 µm.

**Plate 68.**
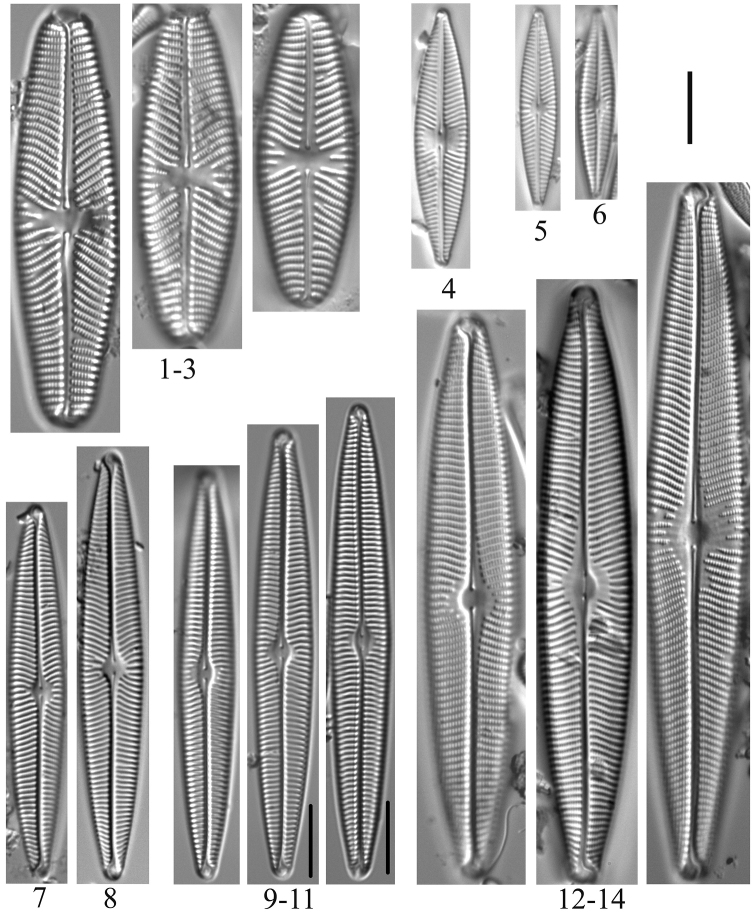
Coppermine. **1–3** *Navicula reinhardtii* (6832) **4** *N. sieminskiae* [cf. *N. sieminskiae* PH] (6833) **5, 6** *N. notha* (6830, 6833) **7, 8** *N. radiosa* (6832, 6833) **9–11** N. tripunctata var. arctica (6830, 6831, 6832) **12–14** *N. vulpina* (6831, 6833). Scale bars: 10 µm.

**Plate 69.**
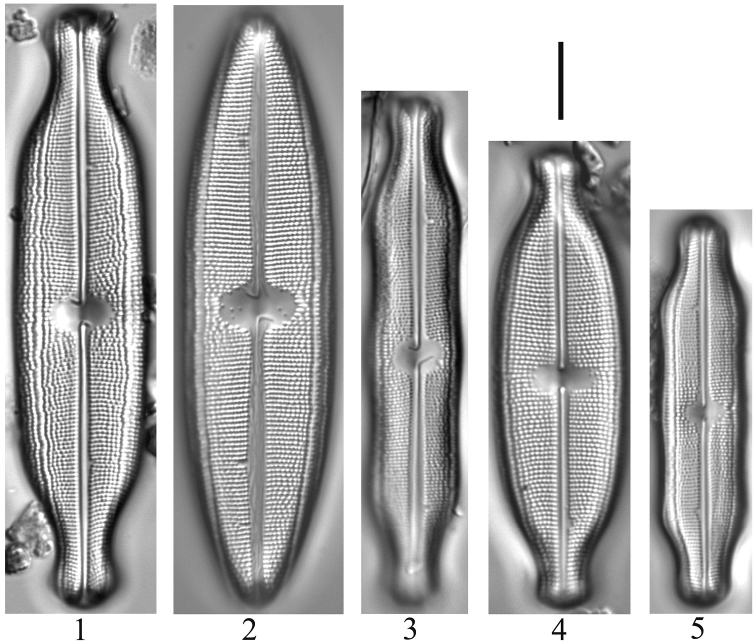
Coppermine. **1** *Neidium productum* (6828, 6833) **2** *N. fossum* (6828, 6831) **3, 5** N. affine var. undulatum (6832) **4** *N. temperei* (6831, 6833). Scale bar: 10 µm.

**Plate 70.**
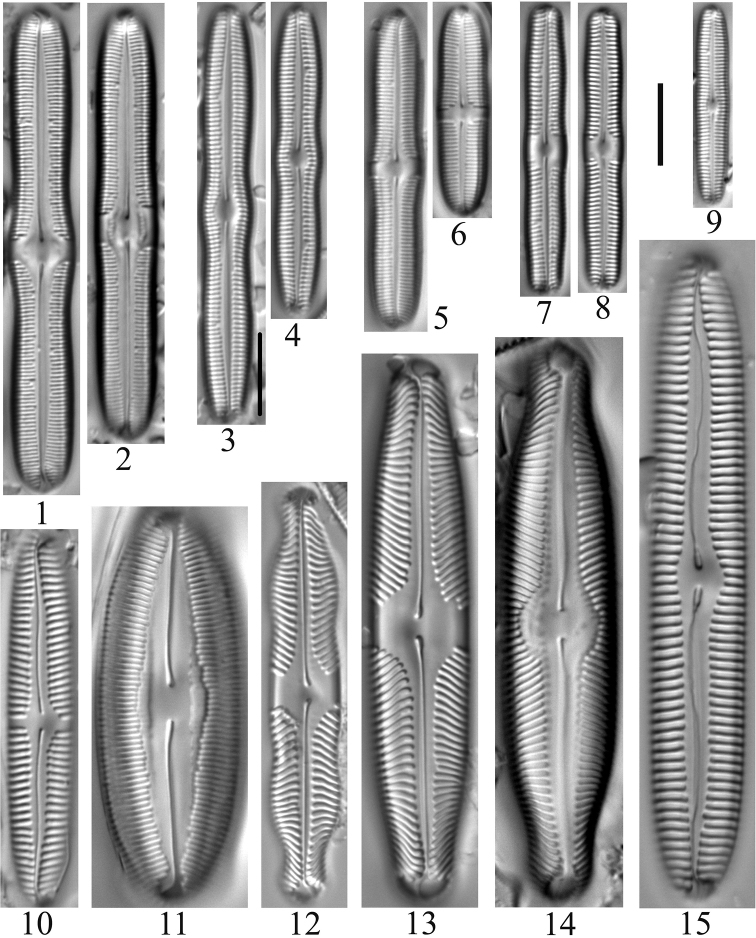
Coppermine. **1–2** *Caloneis* sp. [cf. *C. falcifera*] [*C. falcifera* PH] (6828, 6831) **3, 4** *Caloneis fusus* (6828) **5, 6** *Caloneis* sp. [cf. *C. fasciata*] (6831, 6832) **7, 8** *Caloneis* (*Pinnularia*?) sp. [*Chamaepinnularia* sp.? PH] (6828) **9** *Caloneis tenuis* (6830) **10** *Pinnularia* sp. [cf. *P. pseudogibba*] (6828) **11** *Caloneis obtusa* [*C. obtusa* sensu lato PH] (6824) **12** *Pinnularia grunowii* (6833) **13** *Pinnularia pseudosuchlandtii* [*P. brebissonii* PH] (6826, 6831, 6833) **14** *Pinnularia decrescens* (6832) **15** *Pinnularia* sp. (6828). Scale bars: 10 µm.

**Plate 71.**
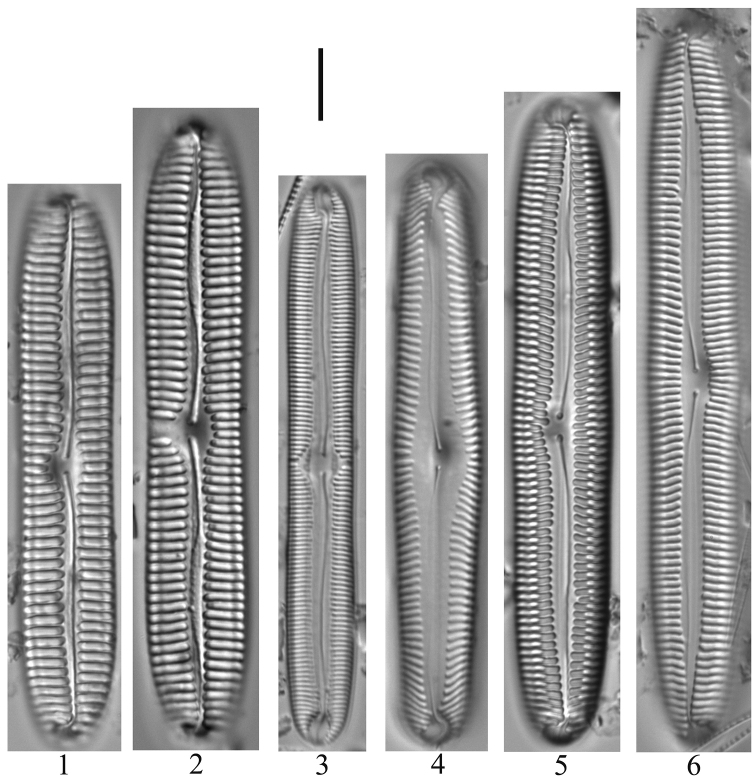
Coppermine. **1, 2** *Pinnularia genkalii* (6833) **3** *P. spitsbergensis* (6833) **4** *P.* sp. [cf. *P. lokana*] (6832) **5** *P. viridiformis* (6832) **6** *P. rupestris* [cf. *P. rupestris* PH] (6832). Scale bar: 10 µm.

**Plate 72.**
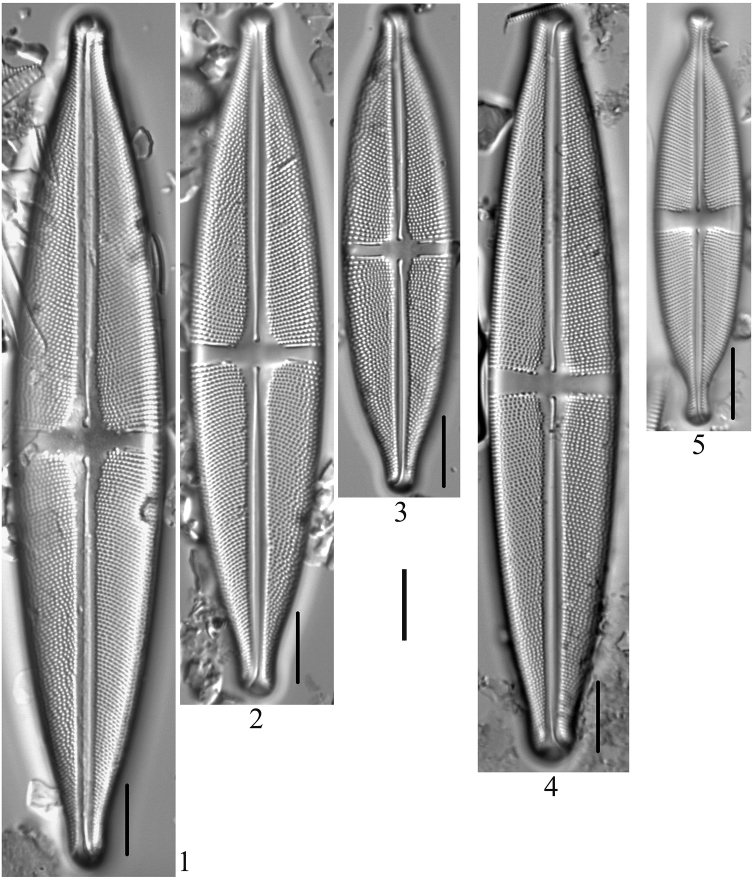
Coppermine. **1–3** *Stauroneis kuelbsii* or *S. superkuelbsii* [*S. gracilis* sensu lato PH] (6828, 6830, 6833) **4** *S. gracilis* (6832) **5** *S. reichardtii* [*S. anceps* PH] (6833). Scale bars: 10 µm.

**Plate 73.**
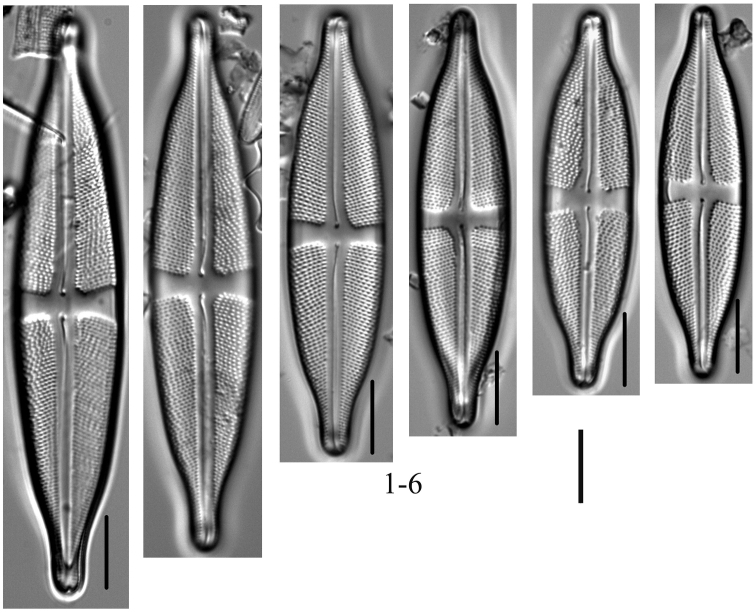
Coppermine. **1–6** *Stauroneis hyperborea* (6831, 6833). Scale bars: 10 µm.

**Plate 74.**
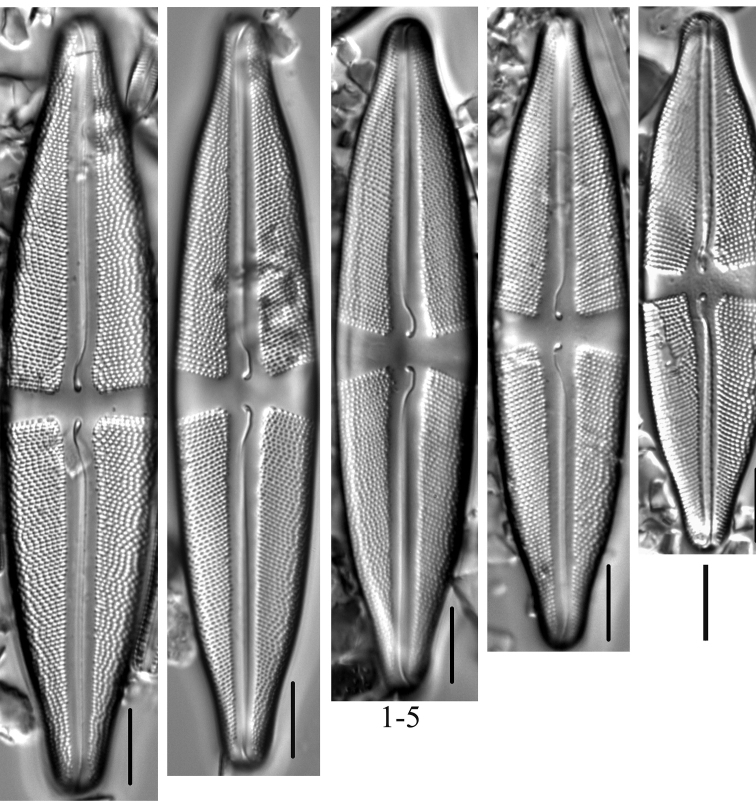
Coppermine. **1–5** *Stauroneis superhyperborea* [syn. S. obtusa var. lapponica Hustedt PH] (6828). Scale bars: 10 µm.

**Plate 75.**
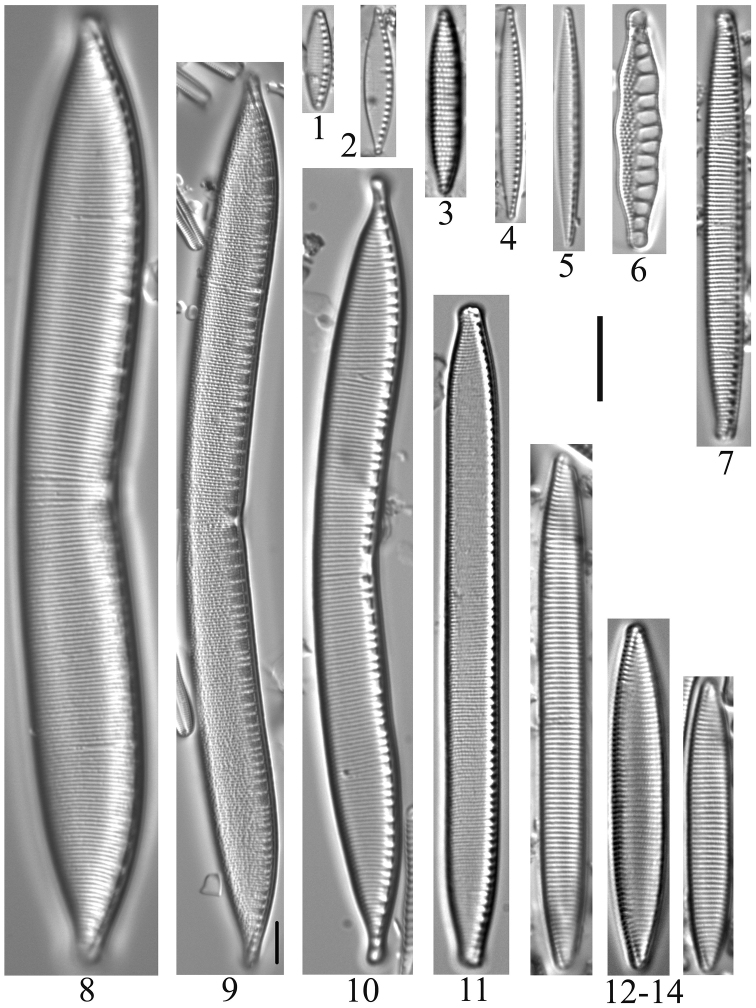
Coppermine. **1** *Nitzschia frustulum* [*N. alpina* PH] (6832) **2** *Nitzschia* sp. [cf. *N. lanceolata*] (6830) **3** *Nitzschia amphibia* (6832) **4** *Nitzschia perminuta* (6831) **5** *Nitzschia radicula* (6832) **6** *Nitzschia sinuata* (6828) **7** *Nitzschia fossilis* [cf. *N. fossilis* PH] (6831) **8** *Hantzschia hyperborea* (6828) **9** *Hantzschia elongata* (6828) (note different scale) **10** *Hantzschia vivacior* (6828) **11** Nitzschia regula var. robusta (6831) **12–14** *Nitzschia angustata* (6831, 6833). Scale bars: 10 µm.

**Plate 76.**
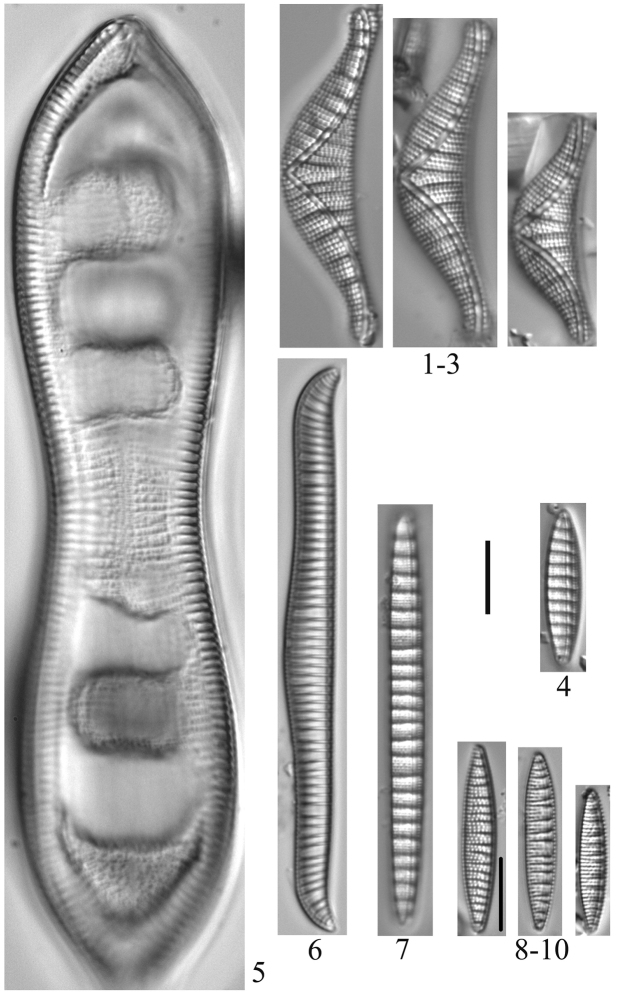
Coppermine. **1–3** *Epithemia smithii* (6826, 6827) **4** *Denticula tenuis* (6830) **5** *Cymatopleura solea* (6832) **6** *Rhopalodia gibba* (6832) **7** *Denticula* sp. [cf. *D. valida*] (6831) **8–10** *Denticula kuetzingii* (6830, 6833). Scale bars: 10 µm.

**Plate 77.**
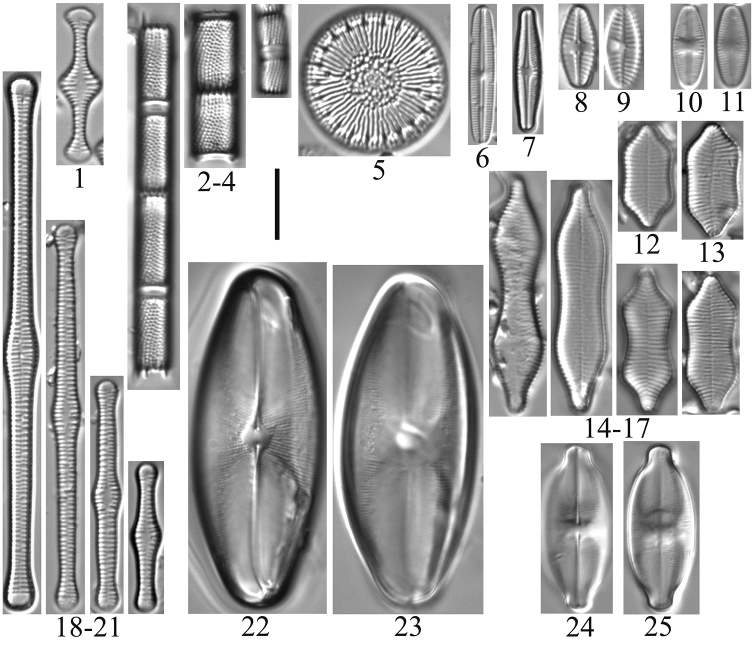
Baillie-Back. **1** *Tabellaria flocculosa* (6864) **2–4** *Aulacoseira subarctica* (6858, 6864) **5** *Lindavia intermedia* (6858) **6** *Rossithidium petersenii* (6859) **7** *Achnanthidium* sp. (6858) **8, 9** *Planothidium frequentissimum* (6856) **10, 11** *Psammothidium marginulatum* (6864) **12–17** *Fragilariforma constricta* (6864) **18–21** *Tabellaria fenestrata* [*T. flocculosa* planktonic form sensu Koppen PH] (6857, 6858, 6859) **22, 23** *Eucocconeis flexella* (6857) **24, 25** *Eucocconeis depressa* (6857). Scale bar: 10 µm.

**Plate 78.**
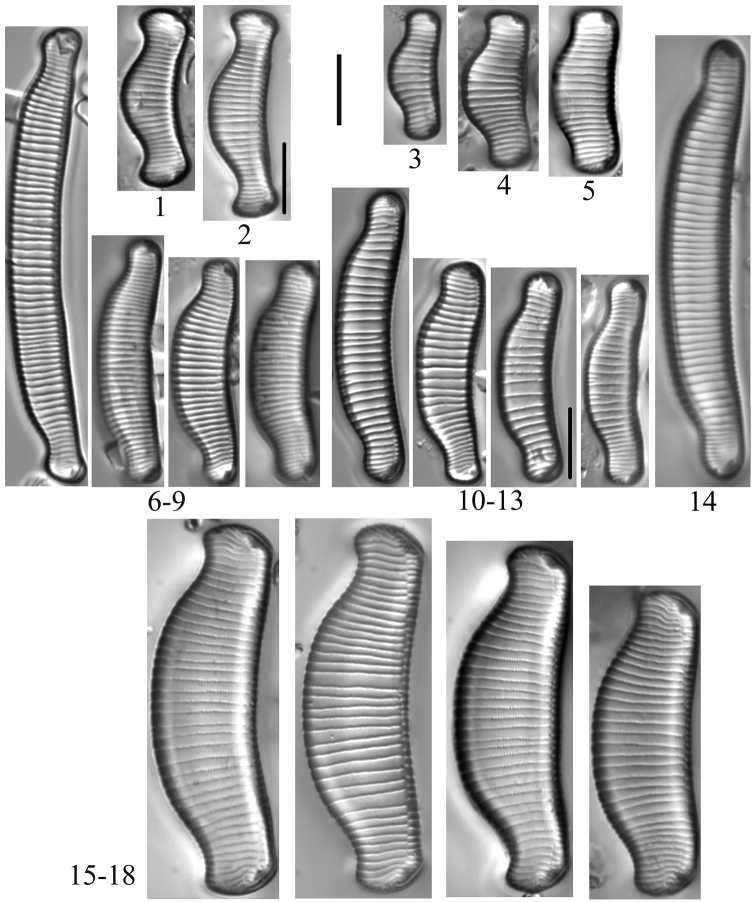
Baillie-Back. **1, 2** *Eunotia curtagrunowii* morphotype I [E. praerupta var. laticeps = *E. laticeps* PH] (6861) **3–5** *E. curtagrunowii* morphotype II (6856, 6857) **6–9** *E. arcus* (6858, 6863) **10–13** *E. arcubus* (6856, 6857, 6858) **14** *E.* sp. [cf. *E. soleirolii*] (6857) **15–18** *E. praerupta* or *E. mayamae* (6866). Scale bars: 10 µm.

**Plate 79.**
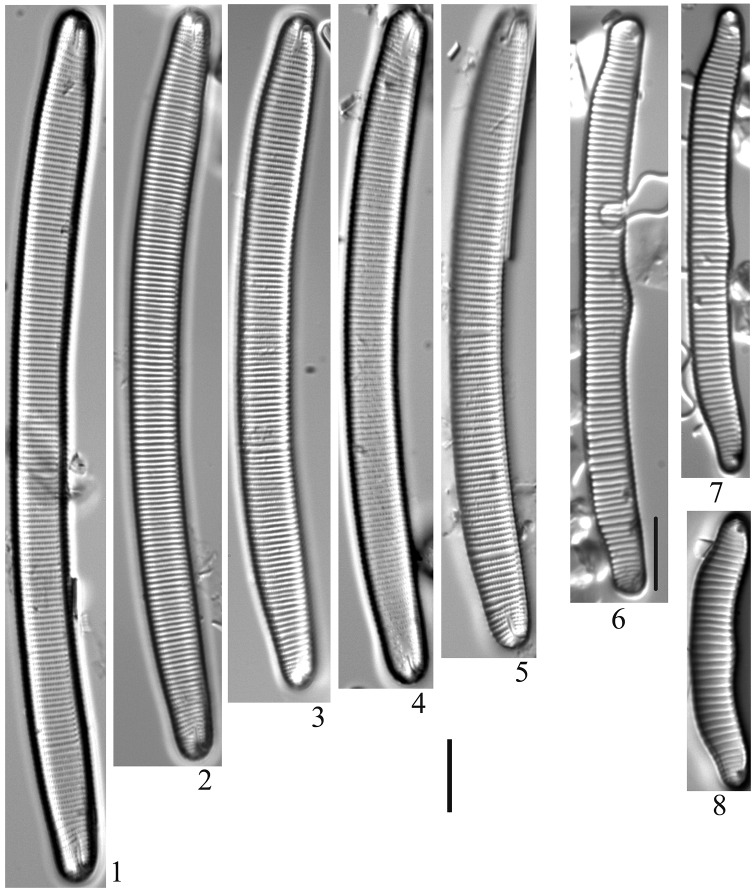
Baillie-Back. **1–5** *Eunotia pseudopectinalis* (6856, 6857, 6859, 6865) **6–8** *E. pectinalis* (6862). Scale bars: 10 µm.

**Plate 80.**
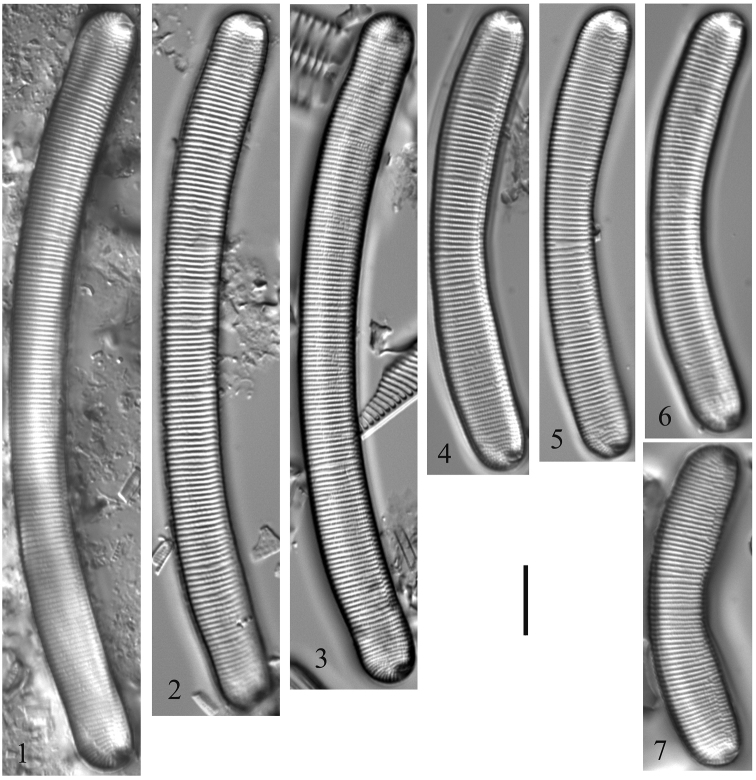
Baillie-Back. **1–7** *Eunotia paralleladubia* (6857, 6861, 6864, 6865). Scale bar: 10 µm.

**Plate 81.**
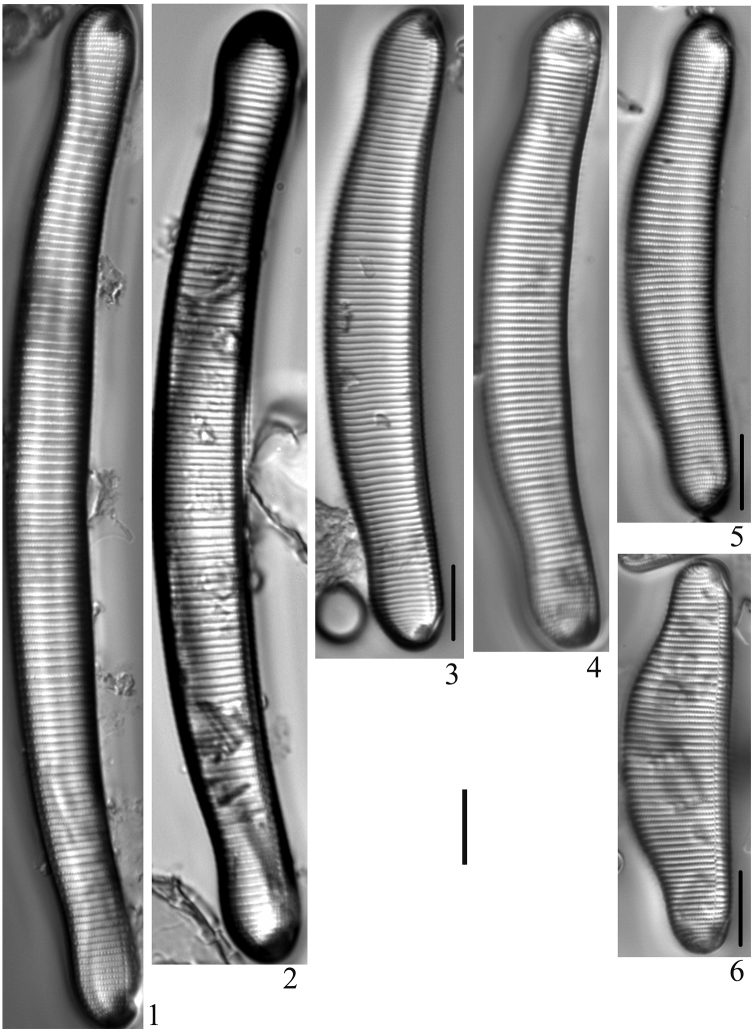
Baillie-Back. **1–5 (6?).** *Eunotia metamonodon* (6856, 6857, 6860, 6862, 6865). Scale bars: 10 µm.

**Plate 82.**
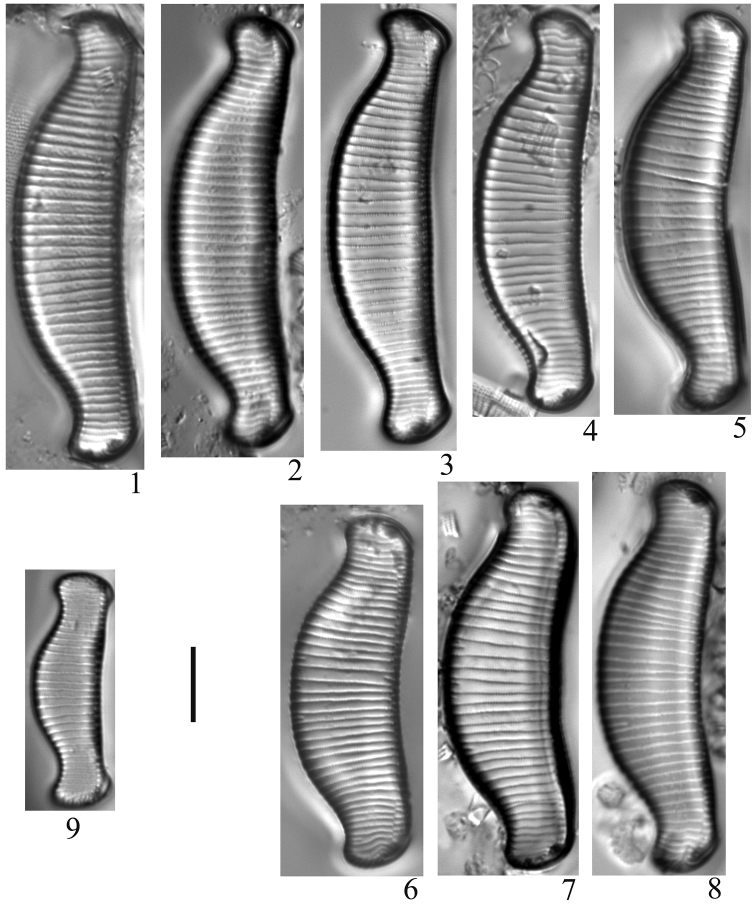
Baillie-Back. **1–8** *Eunotia mayamae* (6856) **9** *E. curtagrunowii* morphotype I [E. praerupta var. laticeps = *E. laticeps* PH] (6866). Scale bar: 10 µm.

**Plate 83.**
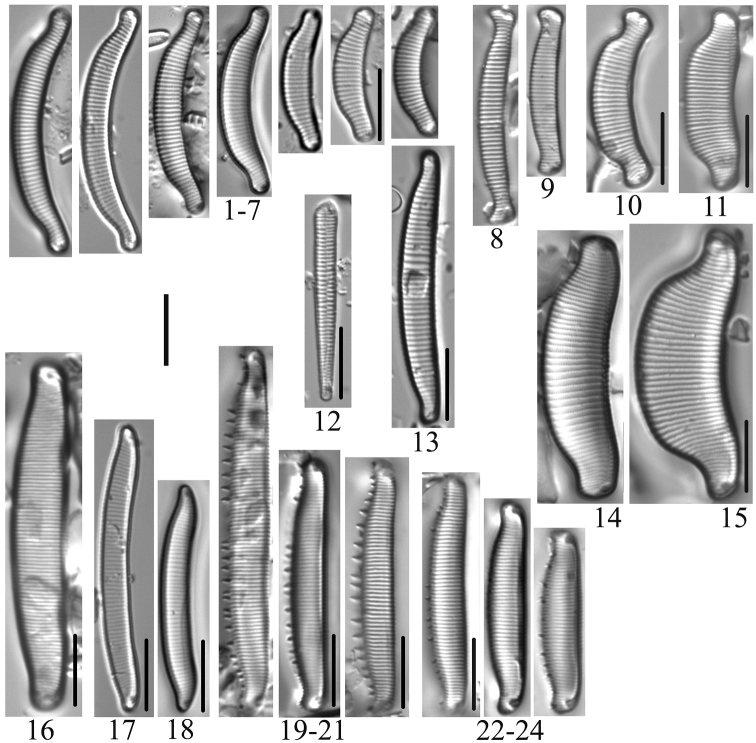
Baillie-Back. **1–7** *Eunotia elegans* [*E. nymanniana* sensu lato PH] (6856, 6857, 6860) **8, 9** *Eunotia neocompacta* (6859, 6864) **10** *Eunotia septentrionalis* (6858) **11** *Eunotia ursamaioris* [*E. ursamaioris* sensu lato PH] (6862) **12** *Peronia fibula* (6865) **13** *Eunotia minor* (6862, 6865) **14** *Eunotia braendlei* (6862) **15** *Eunotia* sp. [cf. *E. ursamaioris*] (6862) **16** *Eunotia lapponica* (6862) **17, 18** *Eunotia silesioscandica* (6858, 6862) **19–21** *Eunotia neoborealis* (6864, 6865) **22–24** *Eunotia denticulata* (6857). Scale bars: 10 µm.

**Plate 84.**
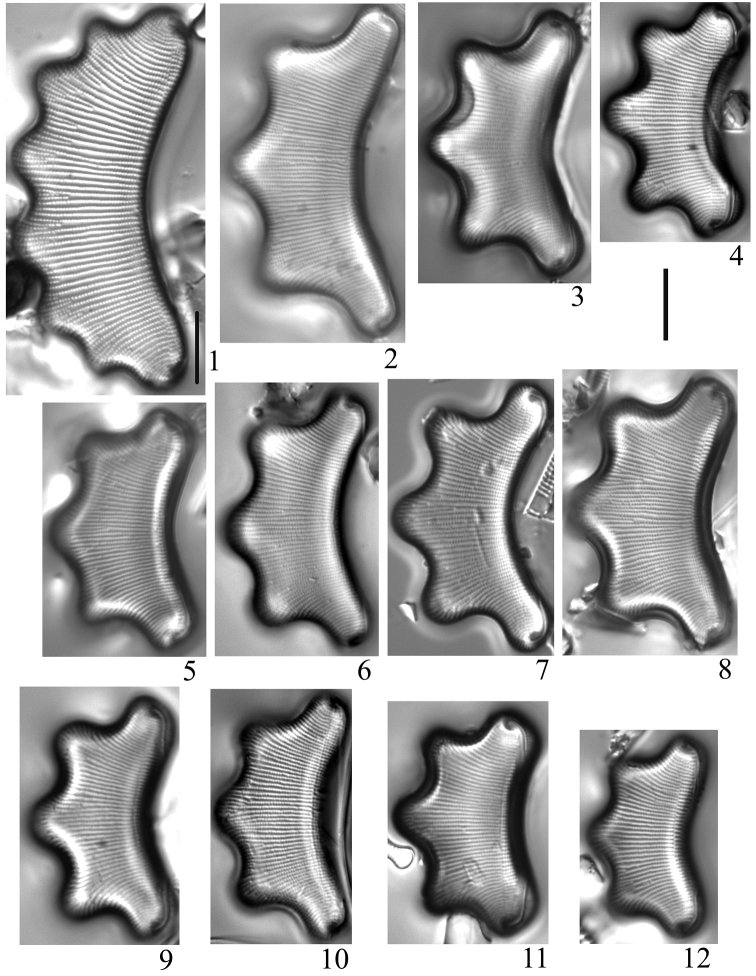
Baillie-Back. **1** *Eunotia diadema* (6862) **2–12** *E. semicircularis* [E. triodon var. semicircularis PH] (6857, 6859, 6865). Scale bars: 10 µm.

**Plate 85.**
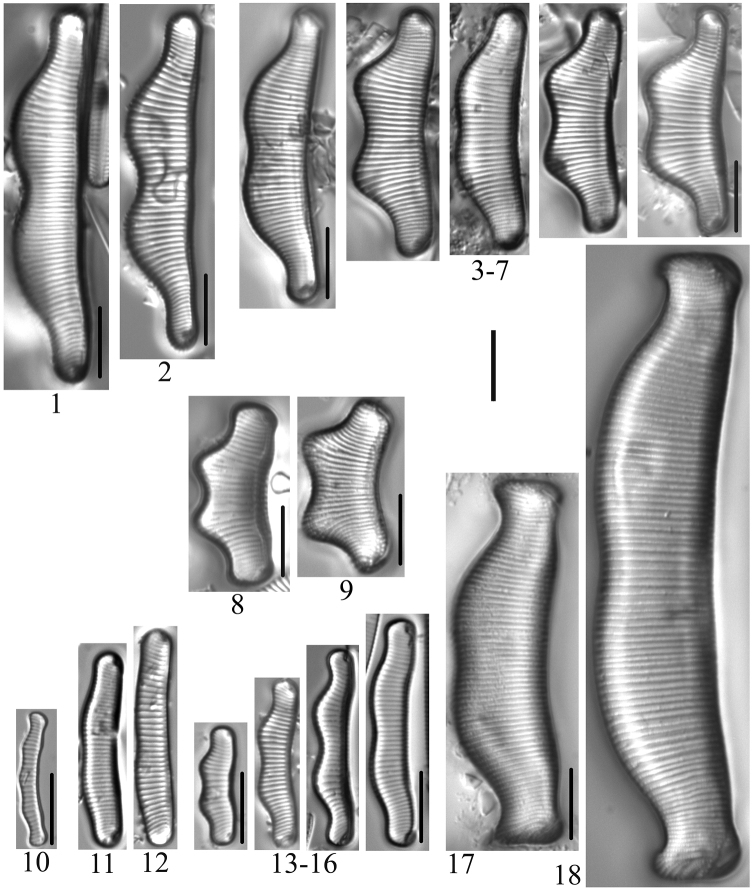
Baillie-Back. **1, 2** *Eunotia diodon* (6865) **3–7** *E. islandica* (6861, 6864) **8, 9** *E. suecica* [Fig. **9** *E. bigibboidea* PH](6861) **10** *E. perminuta* (6861) **11, 12** *E.* sp. (6859, 6865) **13–16** *E. circumborealis* (6856, 6859, 6860, 6865) **17, 18** *E. superbidens* (6861, 6866). Scale bars: 10 µm.

**Plate 86.**
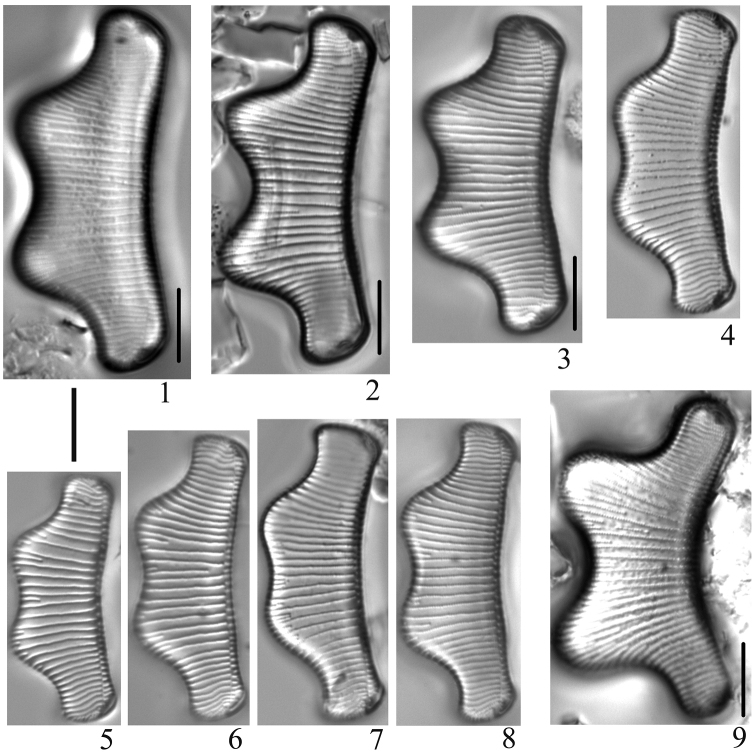
Baillie-Back. **1–8** *Eunotia sarek* (6856, 6863, 6866) **9** *E. pseudopapilio* (6861). Scale bars: 10 µm.

**Plate 87.**
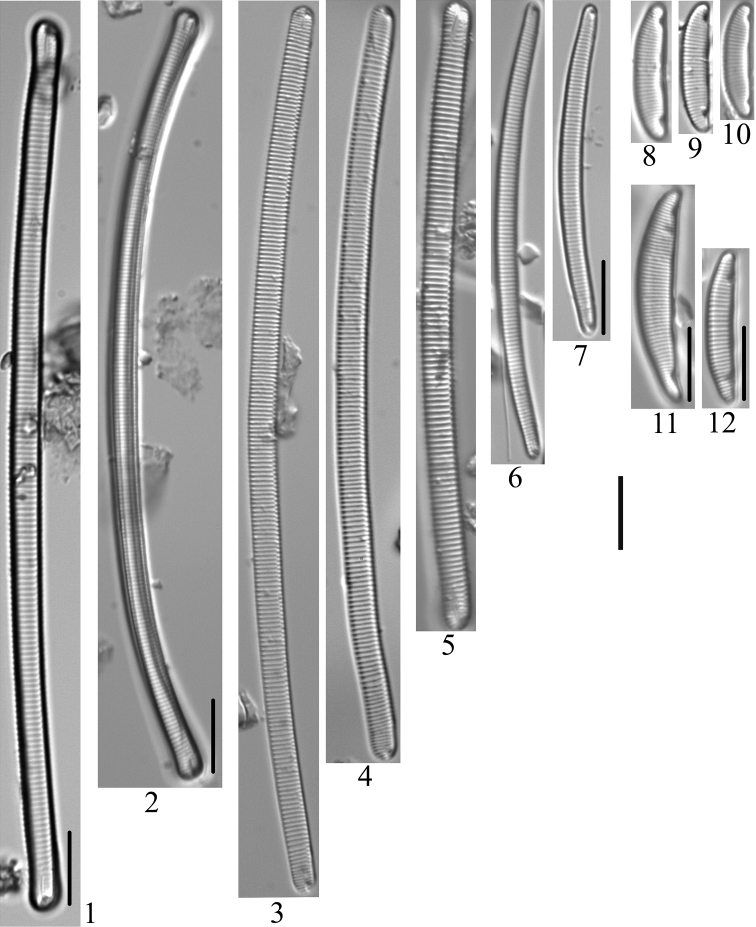
Baillie-Back. **1** *Eunotia pseudoflexuosa* (6860) **2** *E. eurycephala* (6860) **3, 4** *E. ambivalens* (6866) **5** *E. latitaenia* (6859) **6, 7** *E. juettnerae* [*E. bilunaris* PH] (6860, 6864) **8–10** *E. boreoalpina* (6859) **11, 12** *E. incisa* (6862). Scale bars: 10 µm.

**Plate 88.**
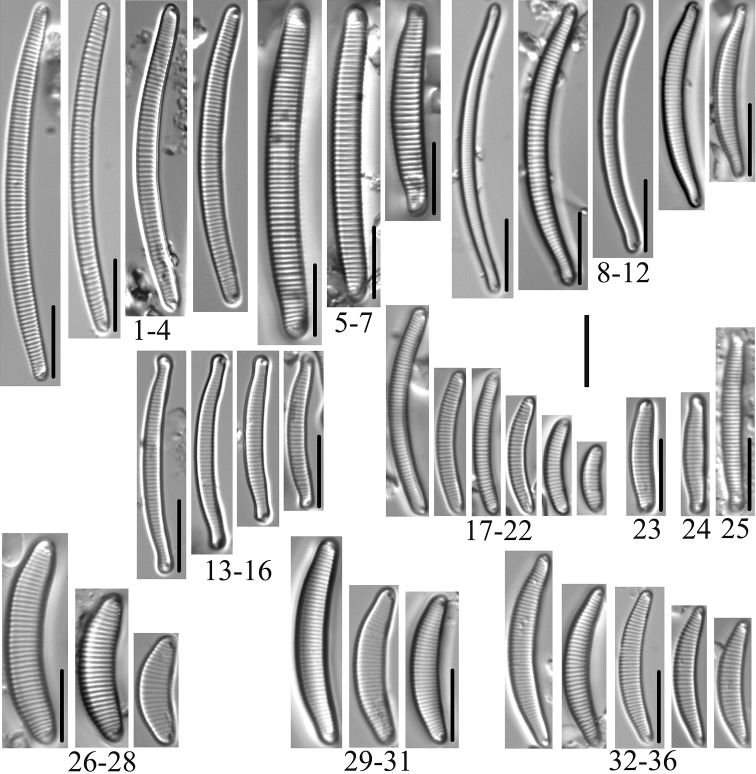
Baillie-Back. **1–4** *Eunotia bilunaris* (6856, 6860, 6863) **5–7** *E. valida* (6856, 6862) **8–12** *E. naegelii* (6860, 6861, 6862) **13–16** *E. pseudogroenlandica* (6860, 6862) **17–22** *E. botuliformis* (6857, 6864) **23** *E. rhomboidea* [*E. paludosa* PH] (6860) **24, 25** *E. paludosa* (6861, 6864) **26–28** *E. scandiorussica* (6856, 6860) **29–31** *E. boreotenuis* [*E. subarcuatoides* PH] (6865) **32–36** *E. subarcuatoides* (6856, 6857, 6860, 6864). Scale bars: 10 µm.

**Plate 89.**
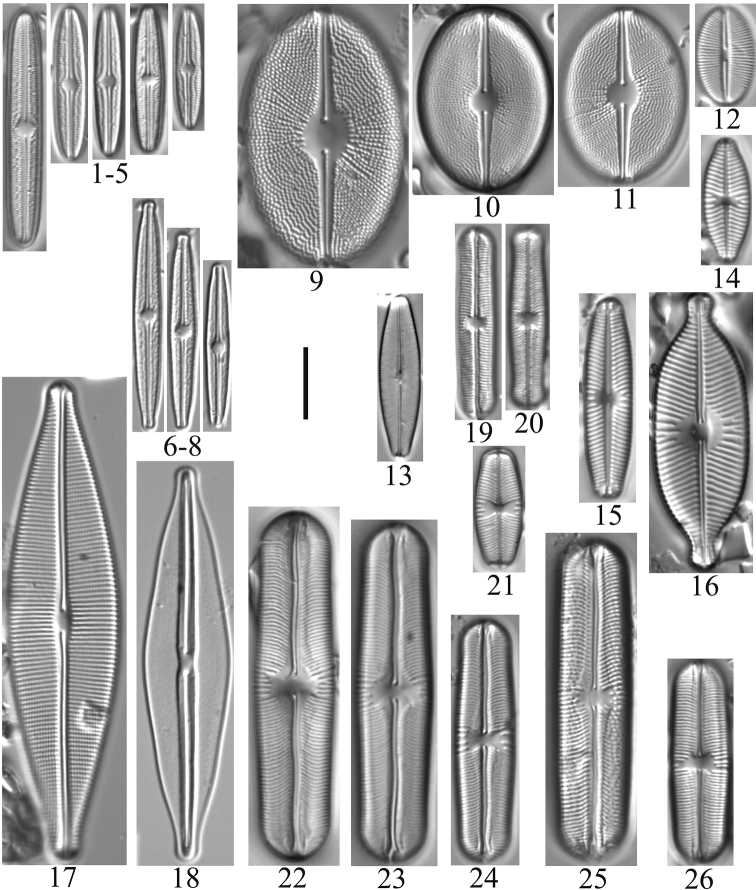
Baillie-Back. **1–5** *Brachysira* sp. (6863, 6864, 6865) **6–8** *Brachysira* sp. (6862, 6865) **9–11** *Cavinula scutiformis* (6862, 6863) **12** *Cavinula jaernefeltii* (6863) **13** *Fallacia* (?) sp. [*Sellaphora* (?) sp. PH] (6862) **14** *Geissleria schoenfeldii* (6862) **15** *Geissleria* sp. [cf. *G. moseri*] (6862) **16** *Geissleria tectissima* (6862) **17** *Craticula* sp. (6862) **18** *Frustulia crassinervia* (6857) **19, 20** *Sellaphora* sp. (6862) **21** *Sellaphora* sp. (6862) **22, 23** *Sellaphora parapupula* (6856, 6860) **24** *Sellaphora* sp. [*S. rectangularis* PH] (6856) **25** *Sellaphora* sp. (6858) **26** *Sellaphora* sp. (6862). Scale bar: 10 µm.

**Plate 90.**
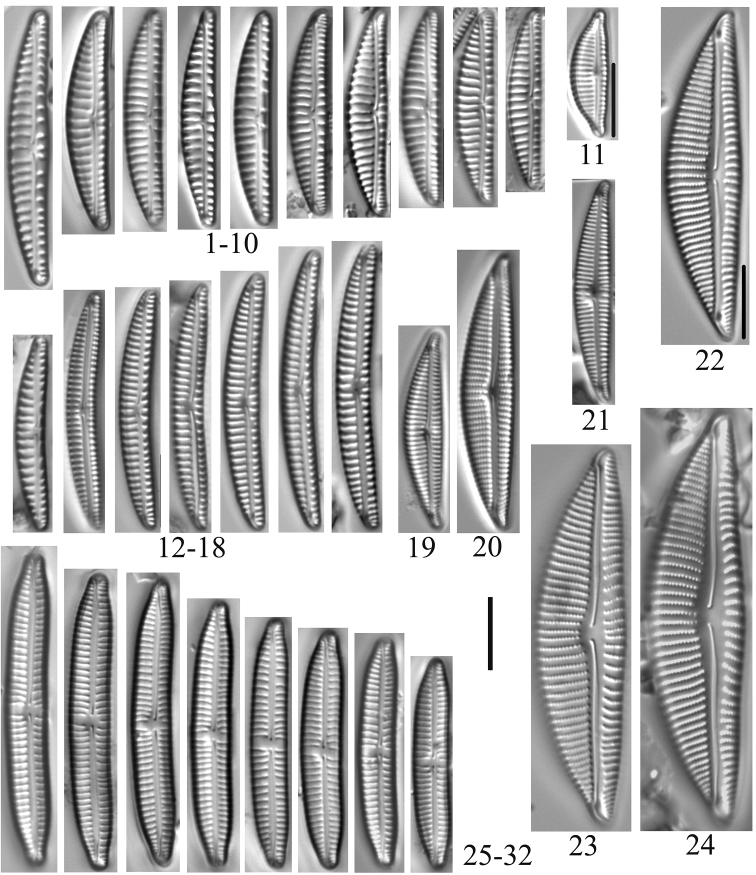
Baillie-Back. **1–10** *Encyonema paucistriatum* (6856, 6857) **11** *E. ventricosum* (6858) **12–18** *E. lunatum* [E. lunatum var. alaskensis PH] (6857, 6860, 6863) **19, 20** *E. willeyorum* (6858) **21** *E. neogracile* (6864) **22–24** *E. hintzii* or *E. vulgare* [*E. vulgare* PH] (6858, 6862, 6866) **25–32** *E. sibericum* (6857, 6860, 6865). Scale bars: 10 µm.

**Plate 91.**
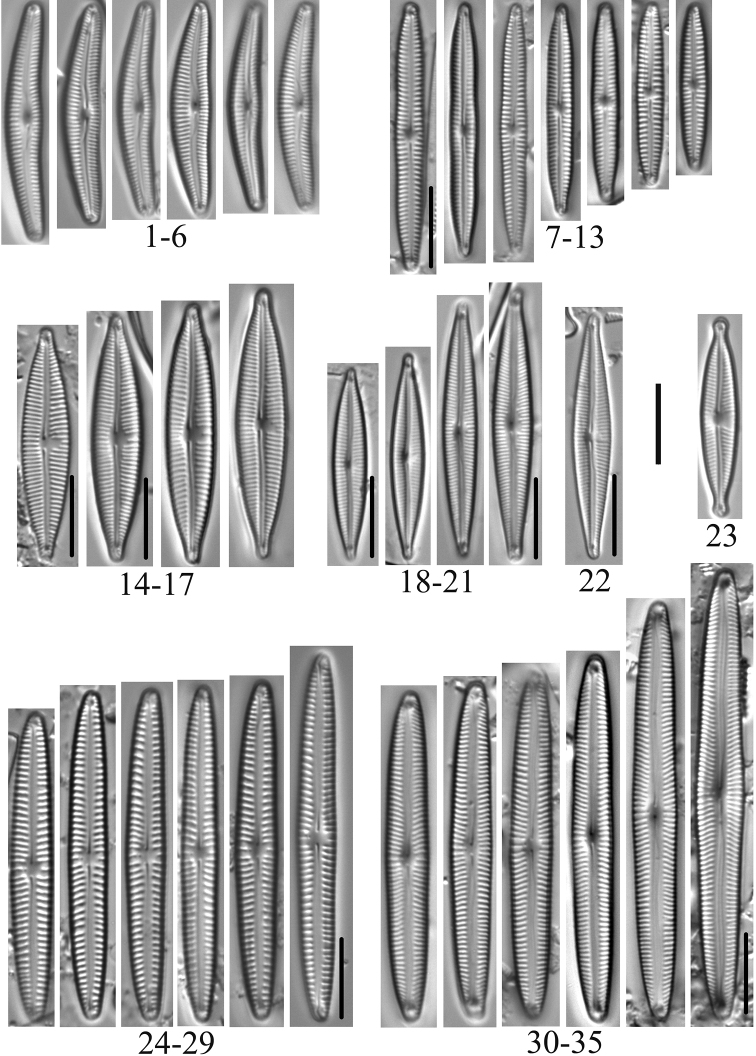
Baillie-Back. **1–6** *Delicata canadensis* (6857, 6858) **7–13** *Encyonopsis inuitorum* (6856, 6857) **14–17** *Encyonopsis cesatiformis* [*E. cesatii* PH] (6856, 6857, 6865, 6866) **18–21** *Encyonopsis cesatii* (6857) **22** *Encyonopsis stafsholtii* (6856) **23** *Encyonopsis* sp. [cf. *E. czarneckii*] (6858) **24–29** *Kurtkrammeria pseudoamphioxys* (6856) **30–35** *Kurtkrammeria neoamphioxys* (6856, 6857). Scale bars: 10 µm.

**Plate 92.**
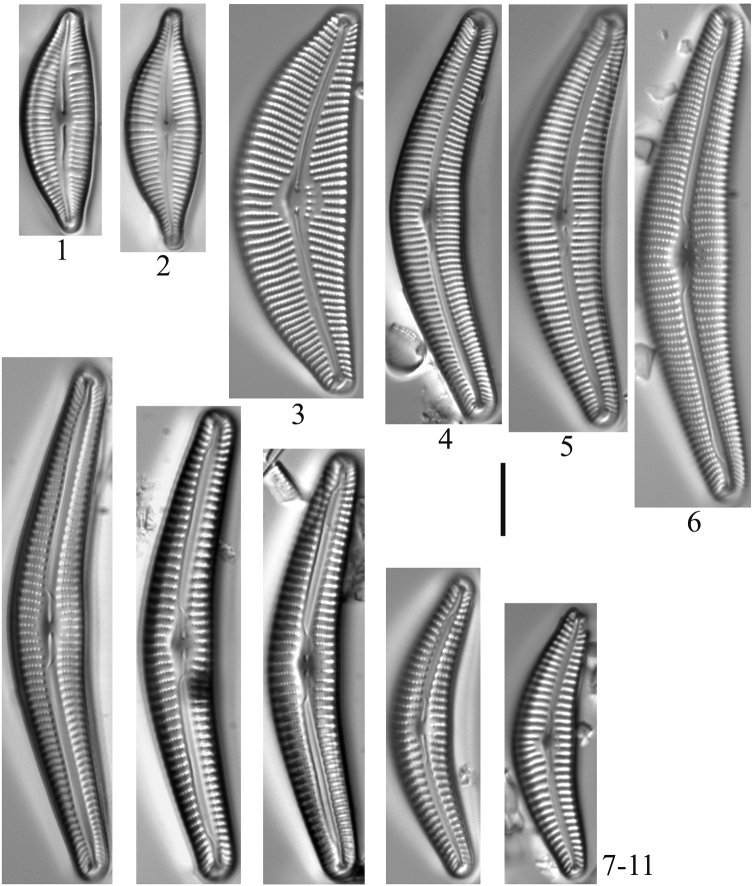
Baillie-Back. **1** *Cymbopleura heilprinensis* [Cymbella (Cymbopleura) designata = *Cymbopleura citriformis* PH] (6857) **2** Cymbella (Cymbopleura) designata = *Cymbopleura citriformis* (6857) **3** *Cymbella proxima* (6858) **4** *Cymbella krammeri* or C. neocistula var. lunata [? PH] (6857) **5, 6** *Cymbella neocistula* (6857, 6858) **7–11** *Cymbella cleve-eulerae* [*C. neocistula* var. PH] (6857, 6862). Scale bar: 10 µm.

**Plate 93.**
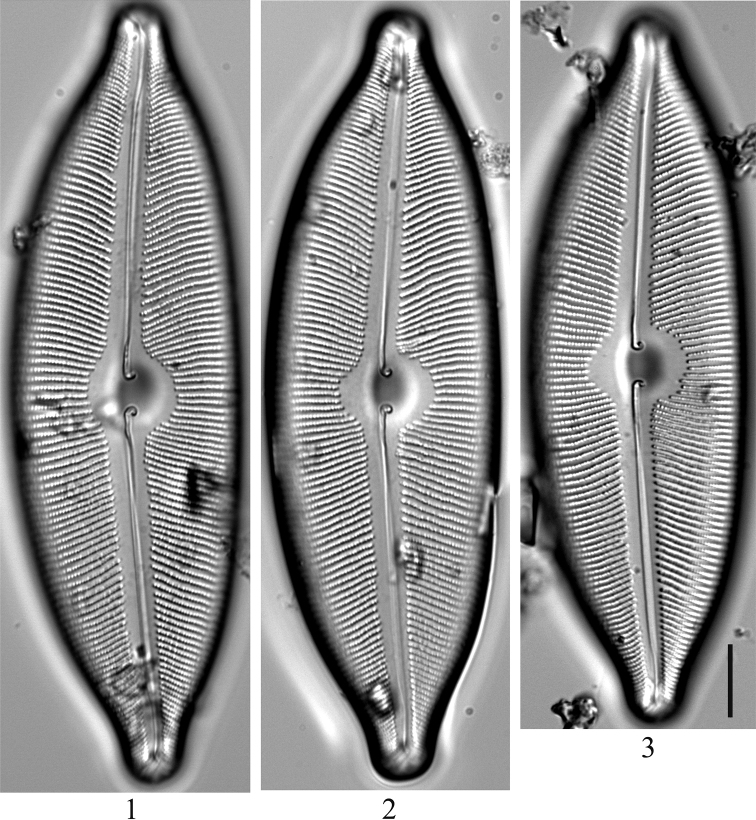
Baillie-Back. **1–3** *Cymbopleura neoheteropleura* (6860). Scale bar: 10 µm.

**Plate 94.**
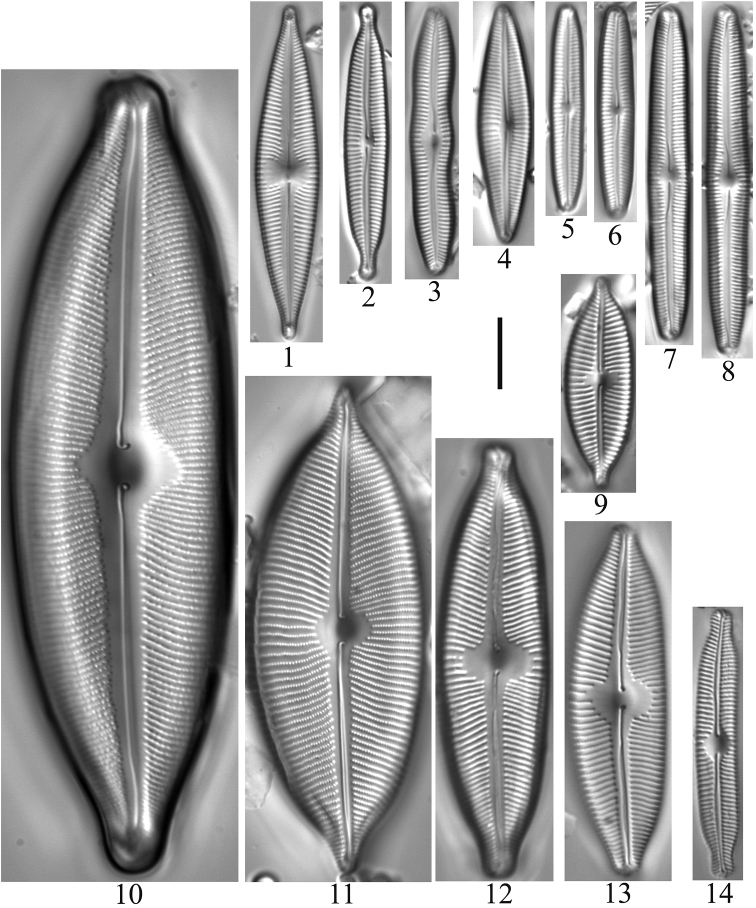
Baillie-Back. **1** *Cymbopleura stauroneiformis* (6857) **2** *C. angustata* (6857) **3** C. incerta var. spitsbergensis (6857) **4** *C.* sp. (6862) **5–8** C. incertiformis var. linearis (6856) **9** *C. anglica* (6862) **10** *C. neoheteropleura* (6857) **11** *C. apiculata* (6858) **12, 13** *C. tynnii* (6856, 6857, 6859, 6863) **14** *C. fluminea* (6858, 6866). Scale bar: 10 µm.

**Plate 95.**
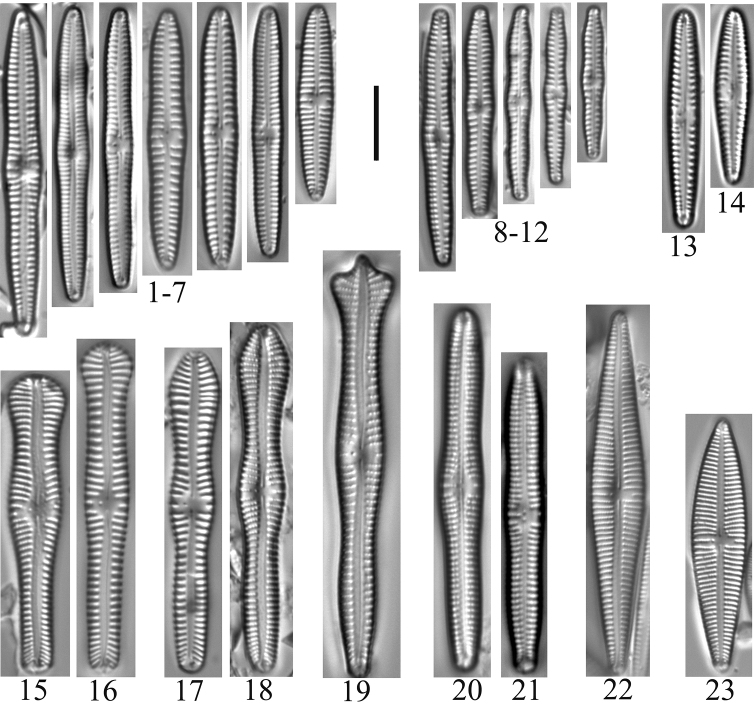
Baillie-Back. **1–7** *Gomphonema lagerheimii* sensu lato (6856, 6857, 6860, 6862) **8–12** *G. astridae* (6856, 6860) **13, 14** *G. lateripunctatum* (6858) **15, 16** *G.* sp. [cf. *G. capitatum*] (6857, 6858) **17, 18** *G. brebissonii* (6856, 6863) **19** *G. coronatumaceum* (6857) **20, 21** *G.* sp. (6857) **22** *G. gracile* (6856) **23** *G.* sp. (6862). Scale bar: 10 µm.

**Plate 96.**
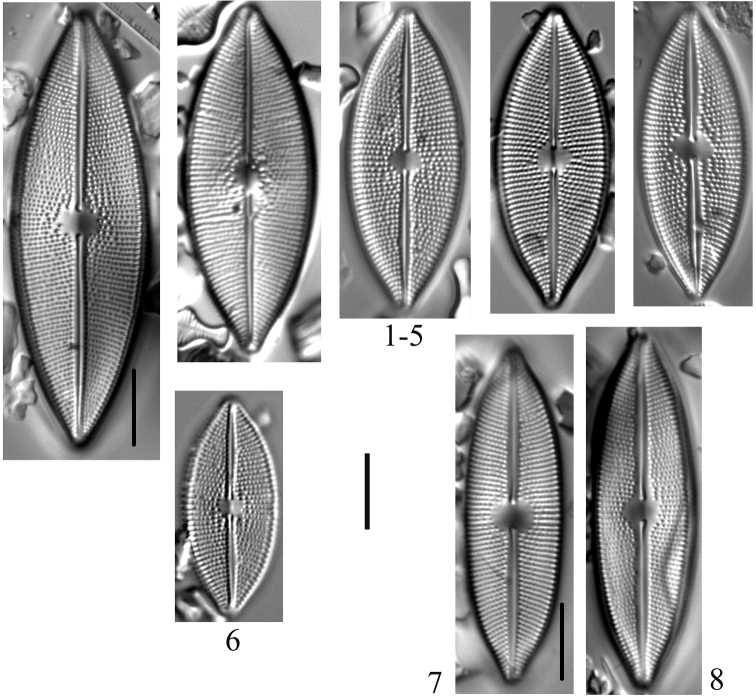
Baillie-Back. **1–6** *Lacustriella lacustris* (6862) **7, 8** *Lacustriella* sp. (6862). Scale bars: 10 µm.

**Plate 97.**
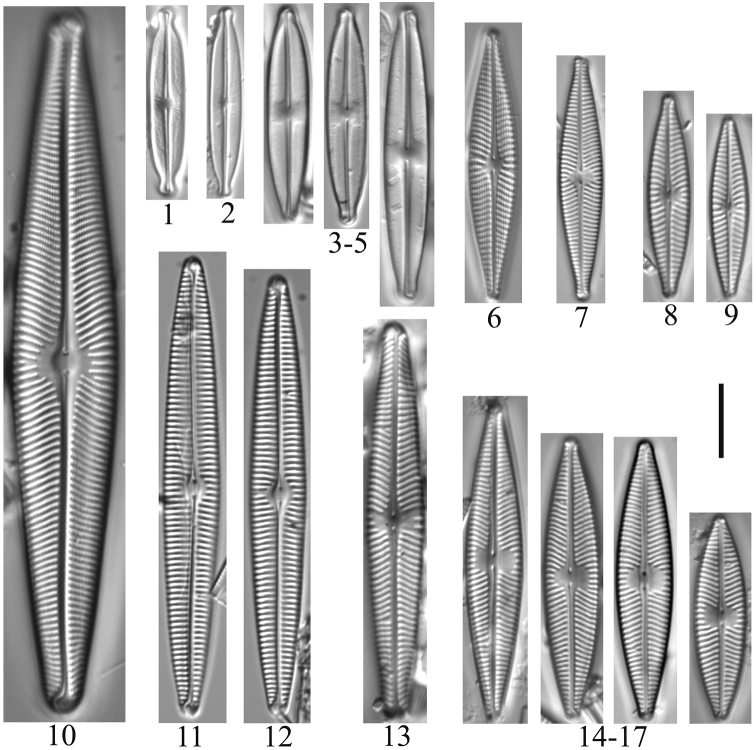
Baillie-Back. **1** *Kobayasiella okadae* (6857) **2** *Kobayasiella micropunctata* (6859) **3–5** *Kobayasiella jaagii* [*K. jaagii* sensu lato PH] (6857) **6** *Navicula subconcentrica* [cf. *N. cryptocephala* sensu lato PH] (6858) **7** *Navicula cryptocephala* [cf. *N. cryptocephala* PH] (6856) **8, 9** *Navicula notha* (6857) **10** *Navicula vulpina* (6857) **11, 12** Navicula tripunctata var. arctica (6857) **13** *Navicula venerablis* (6862) **14–17** *Navicula exilis* (6856, 6857). Scale bar: 10 µm.

**Plate 98.**
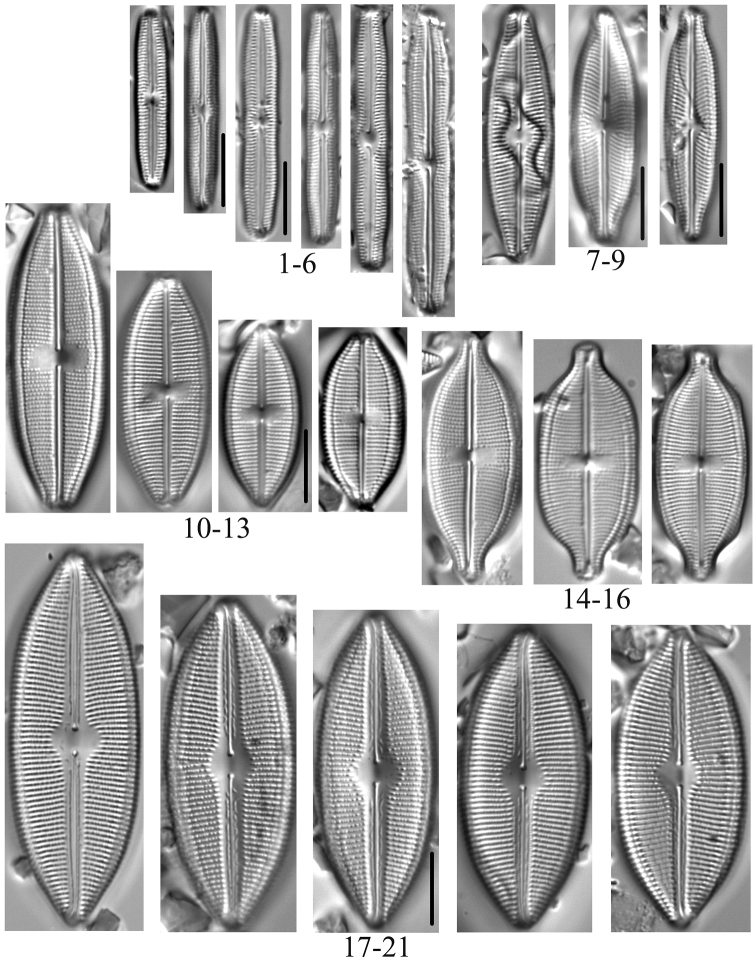
Baillie-Back. **1–6** *Neidiopsis vekhovii* (6856, 6858, 6859, 6862, 6865) **7–9** *Neidiopsis wulffii* (6862) **10–13** *Neidium ladogensis* (6862) **14–16** *Neidium alaskaense* (6862) **17–21** *Neidium holstii* (6858, 6862). Scale bars: 10 µm.

**Plate 99.**
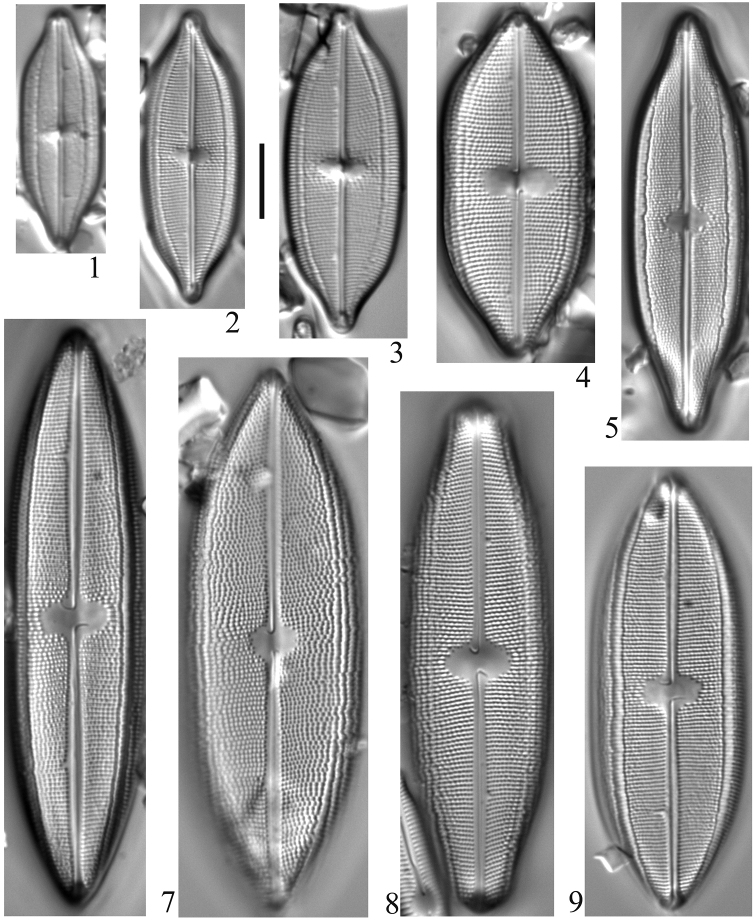
Baillie-Back. **1** *Neidium* sp. (6862) **2, 3** *N. apiculatum* (6862) **4** *N. fossum* [*N.* sp. PH] (6862) **5** N. affine var. humerus (6862) **6** *N. fossum* (6858) **7** *N. amphigomphus* (6863) **8** *N. ampliatum* [*N. ampliatum* sensu lato PH] (6856, 6864) **9** *N. fossum* (6863). Scale bar: 10 µm.

**Plate 100.**
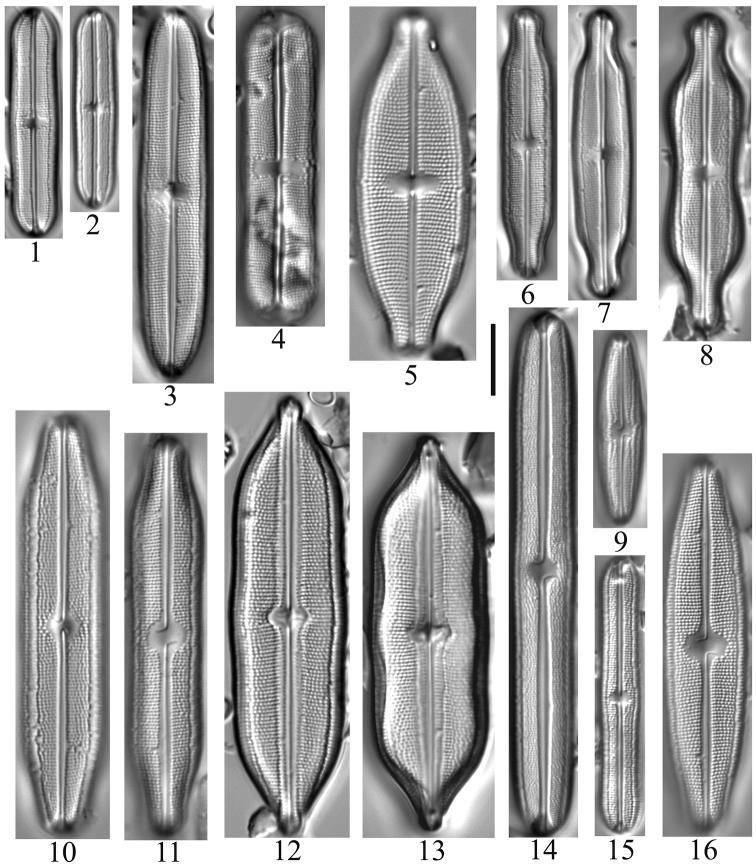
Baillie-Back. **1, 2** *Neidium* sp. (6858, 6862) **3** *N.* sp. [cf. N. bisulcatum var. baicalense PH] (6862) **4** *N.* sp. (6862) **5** *N. temperei* (6863) **6, 7** N. affine var. longiceps [Fig. **6** *N. longiceps*. Fig. **7** *N.* sp. PH] (6858, 6862) **8** *N. affine* (6858) **9** *N. bergii* (6862) **10, 11** *N.* sp. (6859, 6860, 6863) **12, 13** *N. hitchcockii* (6862, 6863) **14, 15** *N. bisulcatum* [Fig. **15** cf. *N. bisulcatum* PH] (6856, 6862, 6864, 6865, 6866) **16** *N. ampliatum* [*N. ampliatum* sensu lato PH] (6856, 6864). Scale bar: 10 µm.

**Plate 101.**
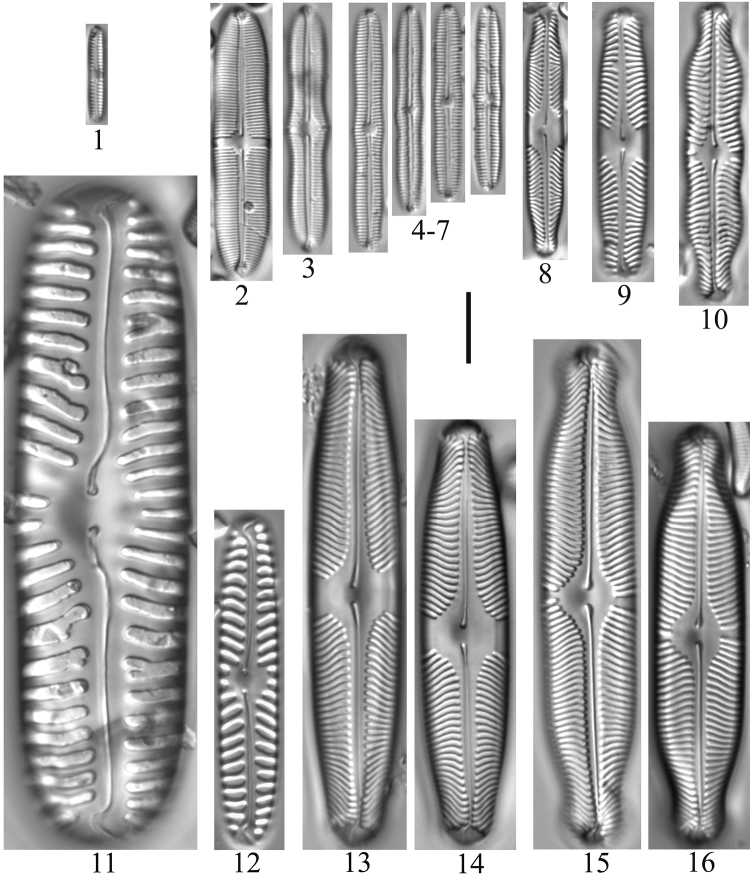
Baillie-Back. **1** *Chamaepinnularia bergeri* (6857) **2** *Caloneis* sp. (6862) **3** *Caloneis silicula* (6866) **4–7** *Caloneis fusus* (6866) **8** *Pinnularia marchica* (6862) **9** *Pinnularia krammeri* (6856) **10** *Pinnularia grunowii* (6863) **11** *Pinnularia lata* (6863) **12** *Pinnularia borealis* [cf. *P. rabenhorstii* PH] (6862) **13, 14** *Pinnularia* sp. [cf. *P. microstauron*] (6857) **15, 16** *Pinnularia biceps* (6857). Scale bar: 10 µm.

**Plate 102.**
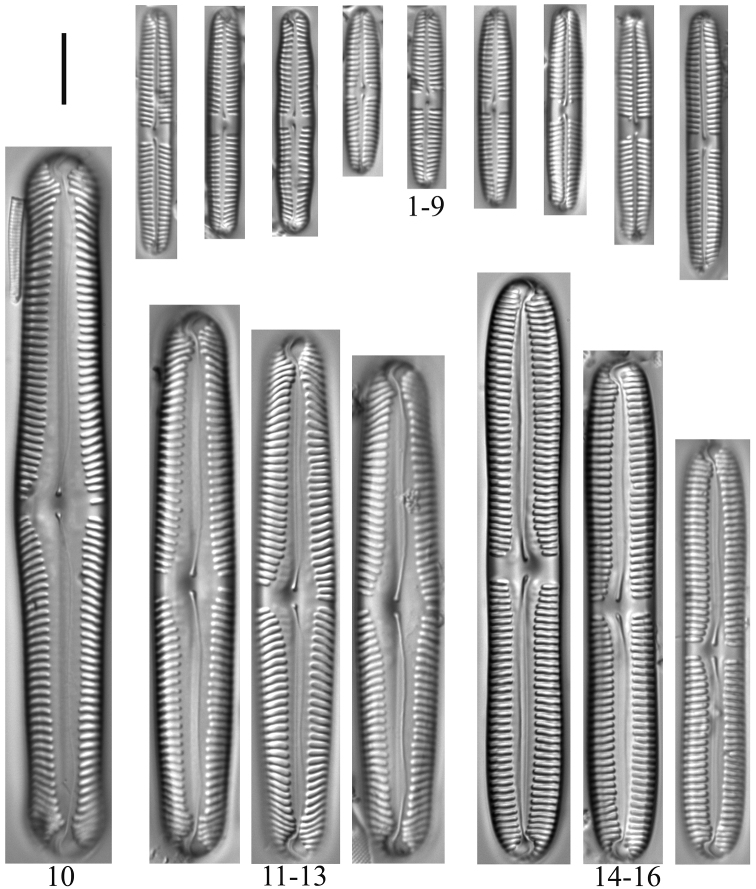
Baillie-Back. **1–9** *Pinnularia* sp. [cf. *P. sinistra*] [*P. sinistra* PH] (6856, 6857, 6864) **10** *P. macilenta* (6857, 6858) **11–13** *P. subgibba* (6856, 6857, 6859) **14–16** *P. crucifera* (6856, 6857, 6859). Scale bar: 10 µm.

**Plate 103.**
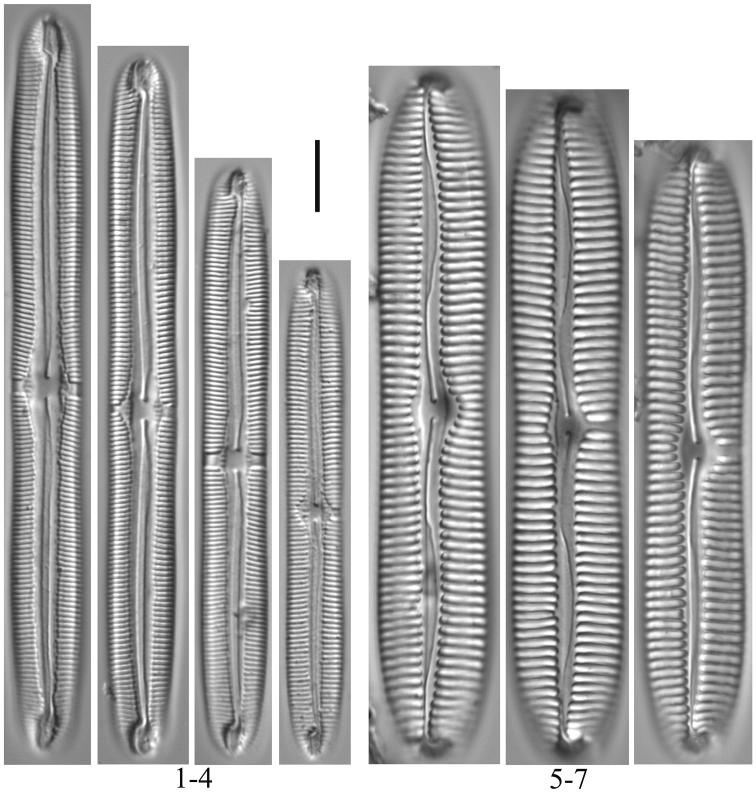
Baillie-Back. **1–4** *Pinnularia spitsbergensis* (6866) **5–7** *P. genkalii* (6866). Scale bar: 10 µm.

**Plate 104.**
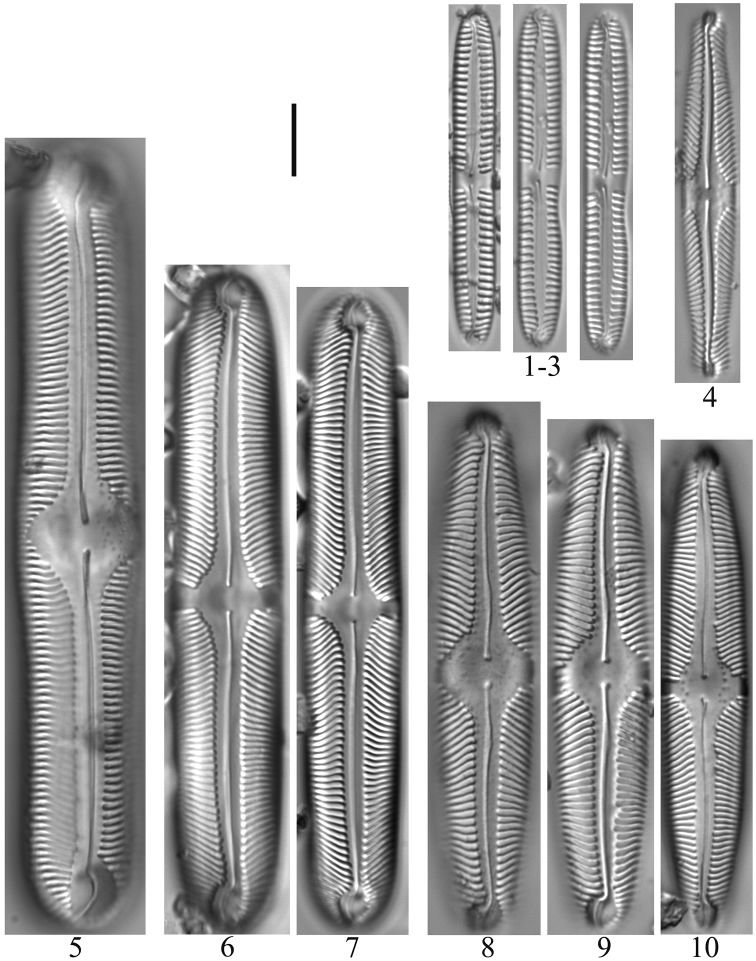
Baillie-Back. **1–3** *Pinnularia sinistra* (6859) [Figs 2 and 3 are the same specimen] **4** *P.* sp. [cf. *P. similiformis* PH] (6866) **5** *P. lailaensis* [cf. *P. lailaensis* PH] (6866) **6, 7** P. divergens var. sublinearis (6862) **8–10** *P.* sp. [cf. *P. decrescens*] (6866). Scale bar: 10 µm.

**Plate 105.**
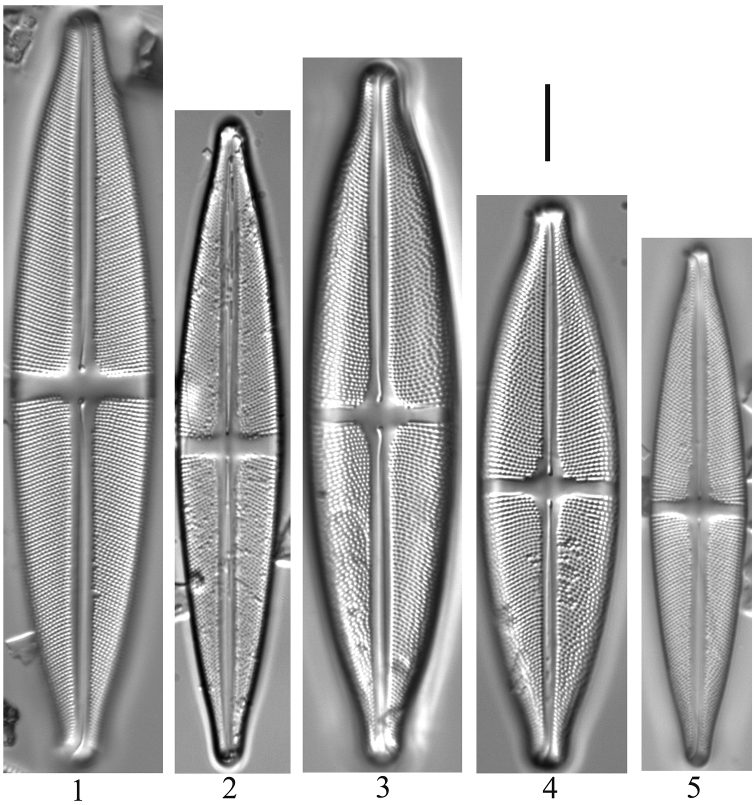
Baillie-Back. **1** *Stauroneis heinii* (6863) **2** *S. angustilancea* (6858) **3, 4** *S. kuelbsii* (6857, 6858) **5** *S. anceps* (6864). Scale bar: 10 µm.

**Plate 106.**
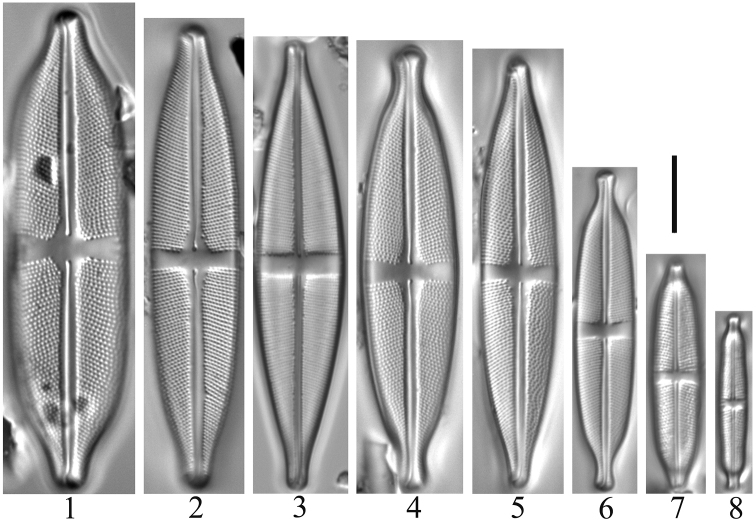
Baillie-Back. **1** *Stauroneis fluminea* (6863) **2** *S. gracilis* (6856) **3** *S. boyntoniae* (6862) **4** *S. amphicephala* (6856) **5** *S. anceps* [*S. anceps* sensu lato PH] (6856) **6** *S. reichardtii* (6856, 6864) **7** *S. livingstonii* (6862) **8** *S. prominula* [= *S. ignorata* = *S. biundulata* PH] (6862). Scale bar: 10 µm.

**Plate 107.**
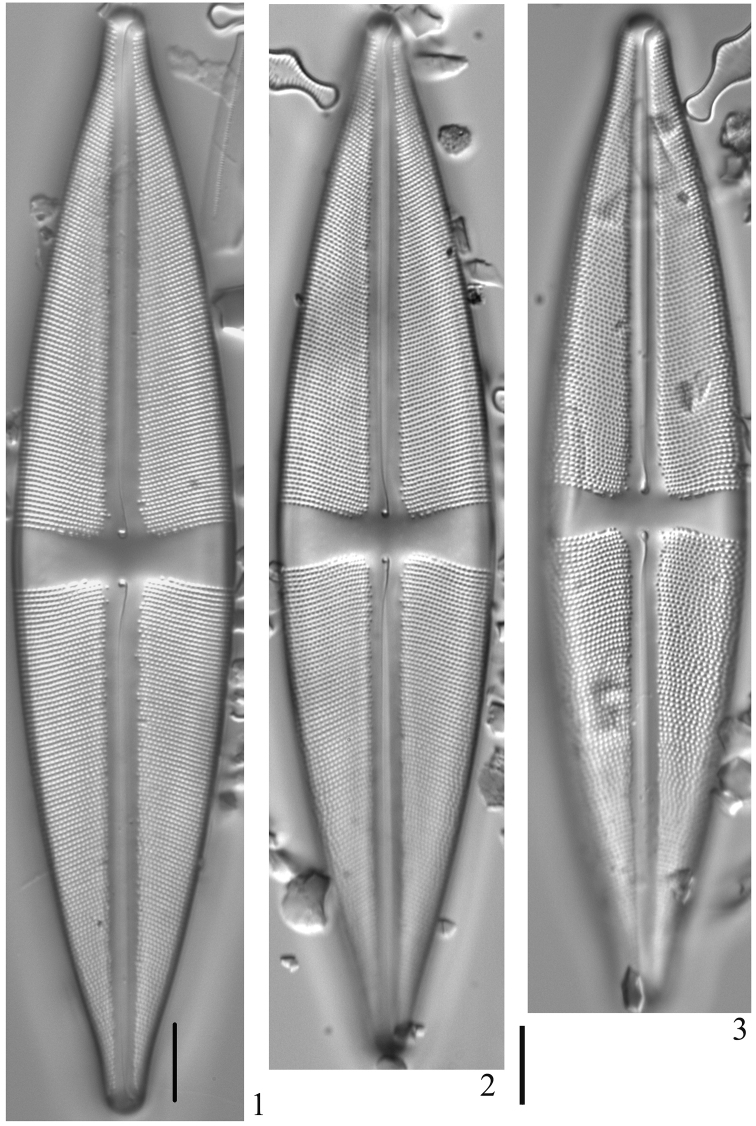
Baillie-Back. **1–3** *Stauroneis circumborealis* (6856, 6864). Scale bars: 10 µm.

**Plate 108.**
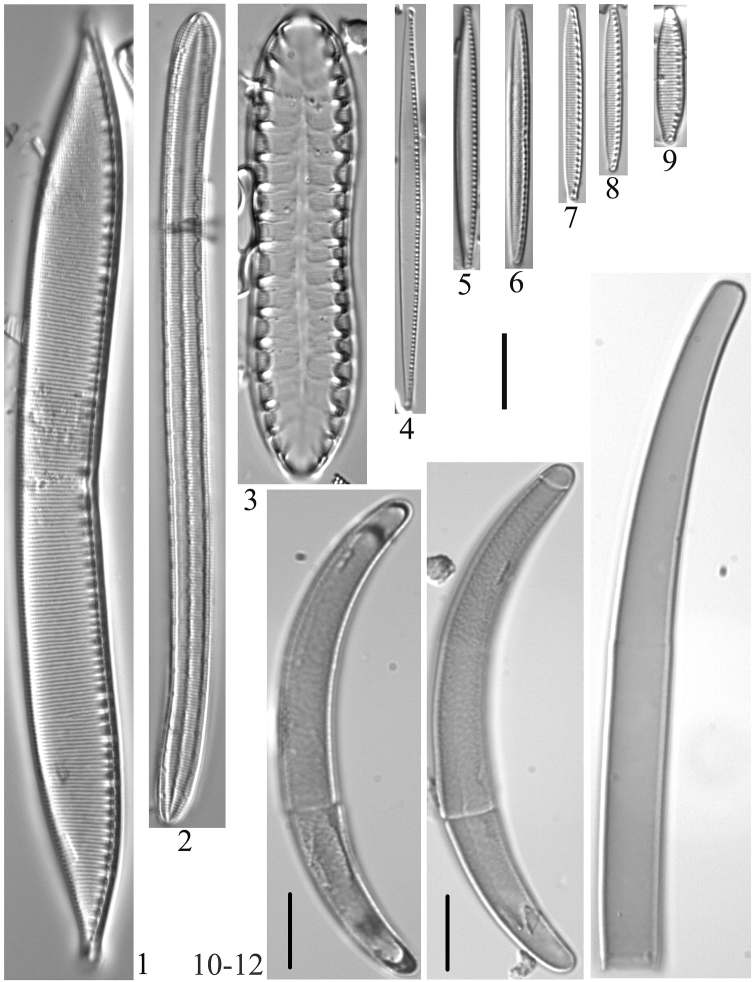
Baillie-Back. **1** *Hantzschia elongata* or *H. vivacior* (6856) **2** *Stenopterobia anceps* (6860) **3** *Surirella linearis* (6858) **4** *Nitzschia gracilis* (6862) **5** Nitzschia palea var. tenuirostris [N. palea var. tenuirostris sensu lato PH] (6857) **6** *Nitzschia acidoclinata* (6856) **7, 8** *Nitzschia perminuta* (6856, 6865) **9** *Nitzschia alpina* (6858) **10–12** UFOs (unidentified floating objects; desmids? *Closterium*?) (6860). Scale bar: 10 µm.

**Plate 109.**
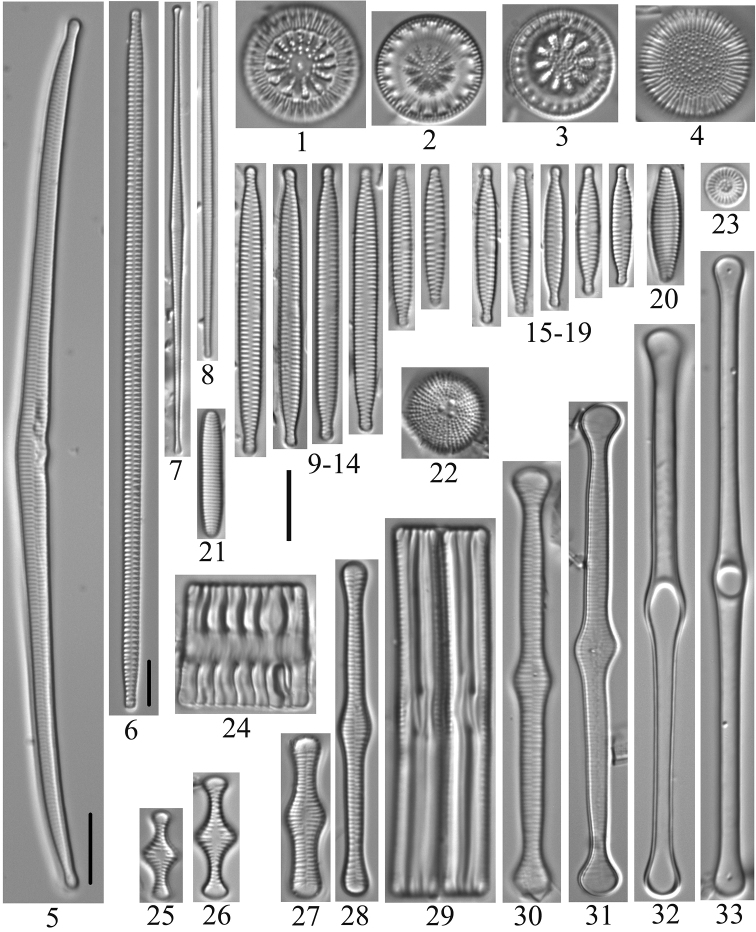
Hood. **1, 2** *Lindavia radiosa* (6898) **3** *Lindavia antiqua* (6898) **4** *Lindavia affinis* (6904) **5** *Hannaea superiorensis* (6906) **6** *Ulnaria* sp. (6907) **7** *Fragilaria crotonensis* (6908) **8** *Fragilaria sepes* (6898) **9–14** *Fragilaria* sp. (6900, 6904, 6909) **15–19** *Fragilaria* sp. [*F. vaucheriae* sensu Tuji PH] (6900) **20** *Stauroforma exiguiformis* (6905) **21** *Stauroforma* sp. (6912) **22** *Orthoseira roeseana* (6904) **23** *Discostella pseudostelligera* (6908) **24–26** *Tabellaria flocculosa* (6898, 6900) **27–33** *Tabellaria fenestrata* [Figs **27–29** *T. flocculosa* PH] (6898, 6906). Scale bars: 10 µm.

**Plate 110.**
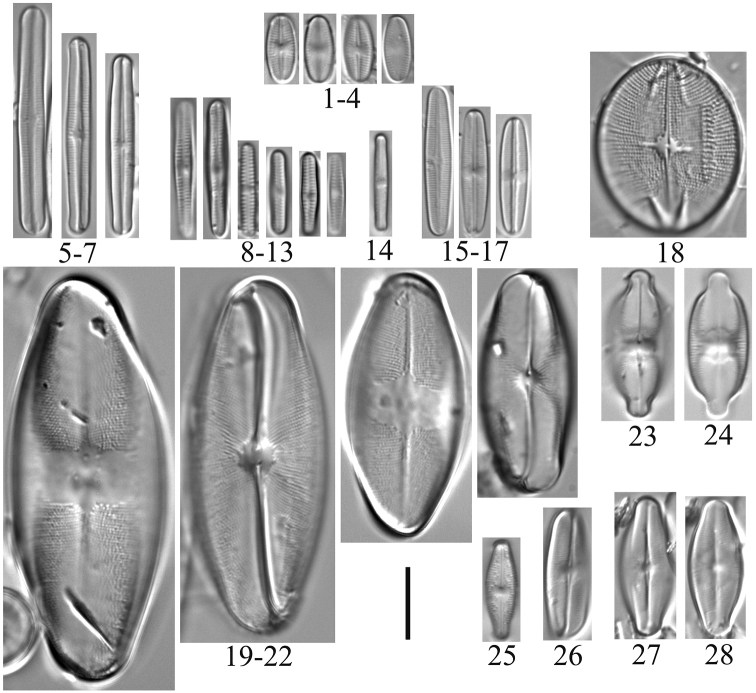
Hood. **1–4** *Psammothidium marginulatum* (6900) **5–7** *Achnanthidium duthiei* [*A*, sp. PH] (6904) **8–13** *Achnanthidium kriegeri* (6900) **14** *Achnanthidium minutissimum* (6912) **15–17** *Rossithidium petersenii* (6900) **18** *Cocconeis rugosa* (6898) **19–22** *Eucocconeis flexella* (6898, 6907) **23, 24** *Eucocconeis depressa* (6907) **25** *Nupela* (?) sp. (6899) **26** *Eucocconeis alpestris* (6909) **27, 28** *Eucocconeis laevis* (6909). Scale bar: 10 µm.

**Plate 111.**
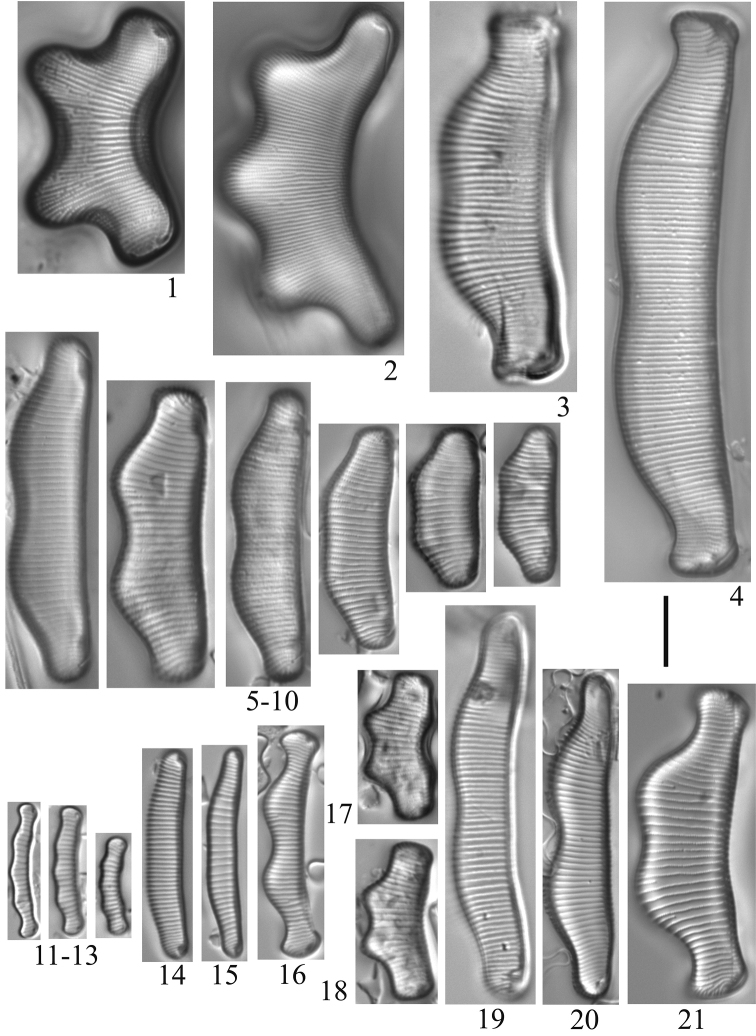
Hood. **1** *Eunotia pseudopapilio* (6898) **2** *E. semicircularis* (6899) **3** *E. bidens* (6898) **4** *E. superbidens* (6908) **5–10** *E. mihoi* [*E.* sp. PH] (6900) **11–13** *E. perminuta* (6900) **14** *E. circumborealis* [*E. rhomboidea* PH] (6900) **15** *E. implicata* (6900) **16** *E. herkiniensis* (6905) **17, 18** *E. suecica* (6900) **19** *E. dorofeyukae* (6898) **20** *E. diodon* (6905) **21** *E. sarek* (6901). Scale bar: 10 µm.

**Plate 112.**
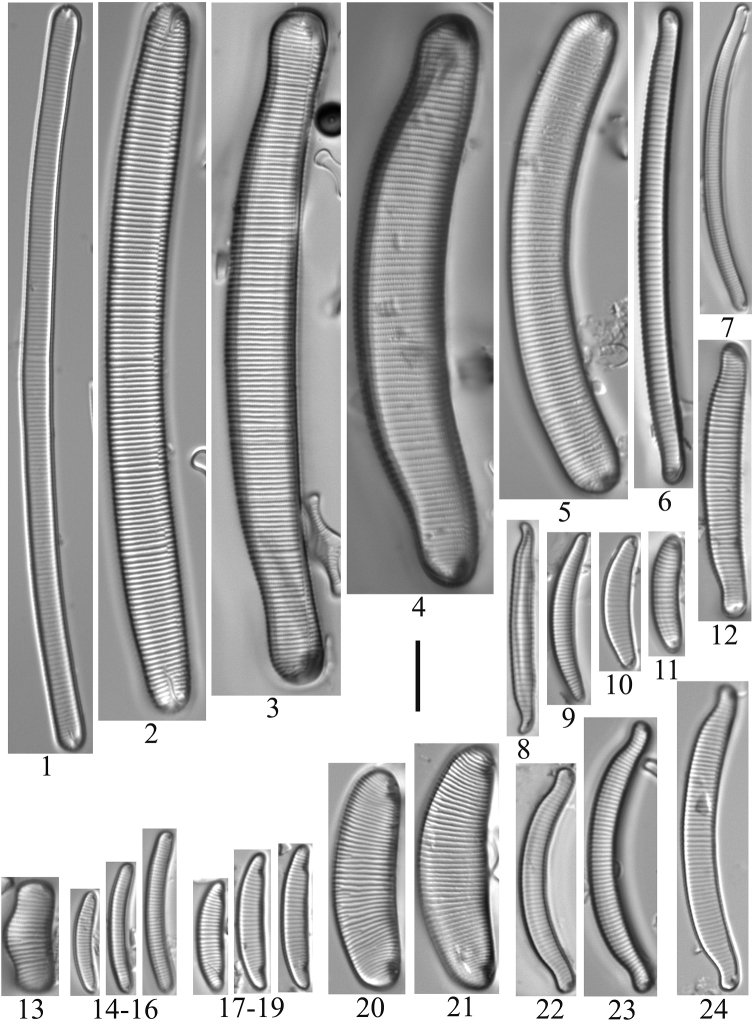
Hood. **1** *Eunotia julma* (6898) **2** *E. pseudopectinalis* (6898) **3** *E. maior* [or *E. metamonodon* PH] (6905) **4** *E. metamonodon* (6898) **5** *E. paralleladubia* (6899) **6** *E. pseudoflexuosa* (6898) **7** *E. mucophila* (6905) **8** *E. fallax* (6898) **9, 10** *E. boreotenuis* [Fig. **9** *E.* sp. PH] (6900, 6907) **11** *E. intermedia* (6900) **12** *E. glacialis* [*E. minor* PH] (6907) **13** *E. curtagrunowii* (6900) **14–16** *E. botuliformis* (6900) **17–19** *E. incisa* (6900) **20, 21** *E. faba* (6905) **22, 23** *E. nymanniana* (6898, 6905) **24** *E. exigua* [*E.* sp. PH] (6900). Scale bar: 10 µm.

**Plate 113.**
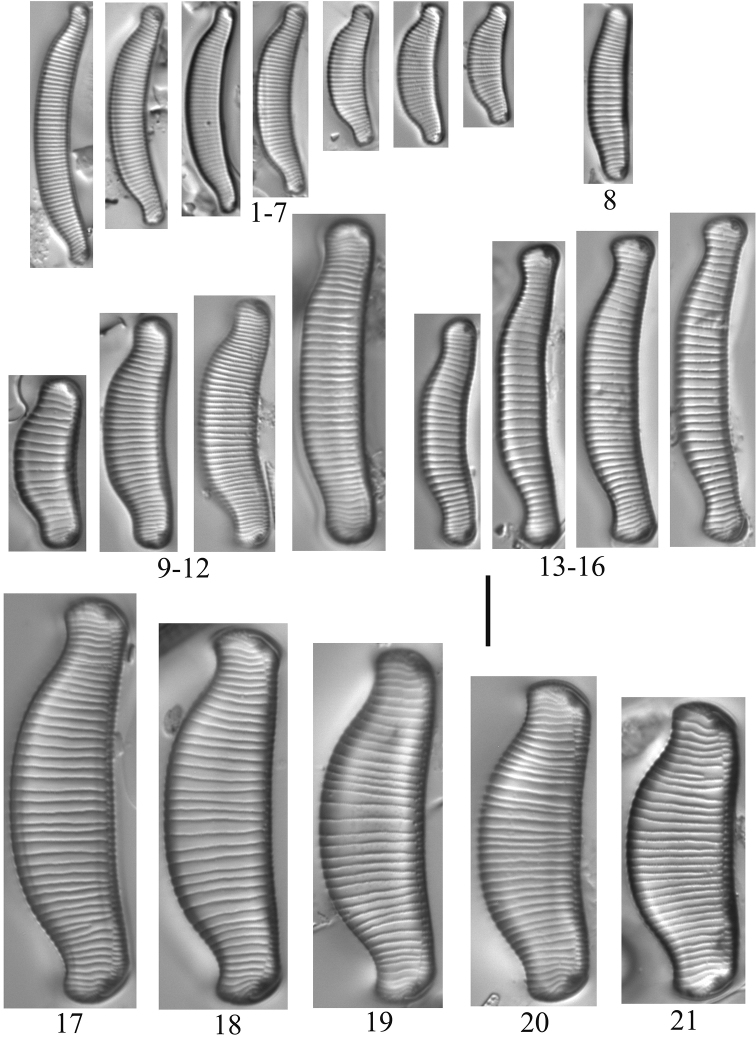
Hood. **1–7** *Eunotia ursamaioris* (6900) **8** *E. minor* [or *E. rhomboidea* PH] (6900) **9–12** *E. arcus* (6898, 6899, 6907) **13–16** *E. arcubus* (6898, 6907) **17–21** *E. praerupta* (6901, 6903). Scale bar: 10 µm.

**Plate 114.**
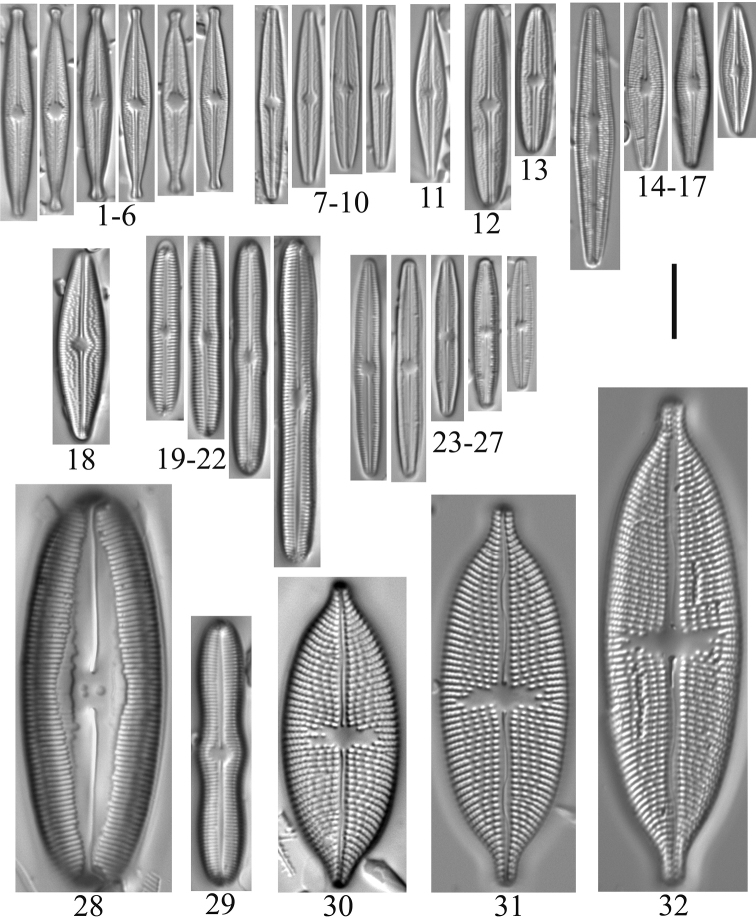
Hood. **1–6** *Brachysira* sp. (6898, 6904, 6908) **7–10** *Brachysira* sp. (6905) **11** *Brachysira microcephala* (6913) **12, 13** *Brachysira* sp. [cf. *Brachysira calcicola*] (6900) **14–17** *Brachysira styriaca* (6898, 6908) **18** *Brachysira arctoborealis* (6905) **19–22** *Caloneis fusus* (6898, 6904, 6906, 6912) **23–27** *Caloneis tenuis* [? PH] (6904, 6908) **28** *Caloneis obtusa* (6908) **29** *Caloneis silicula* (6912) **30–32** *Aneumastus tusculus* (6906, 6909). Scale bar: 10 µm.

**Plate 115.**
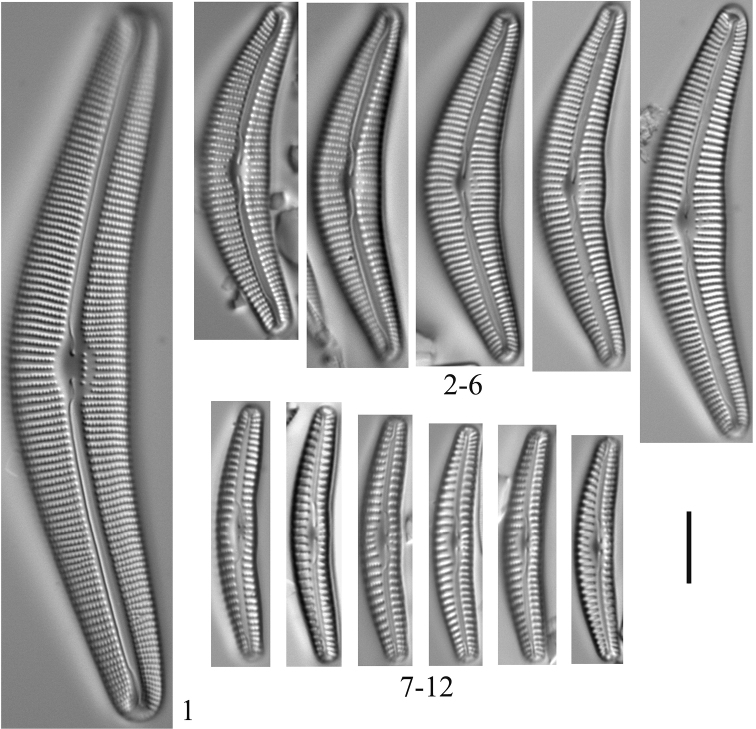
Hood. **1** *Cymbella arctica* (6904) **2–6** *C. krammeri* (6898, 6904, 6906, 6909) **7–12** *C. botellus* (6904, 6906). Scale bar: 10 µm.

**Plate 116.**
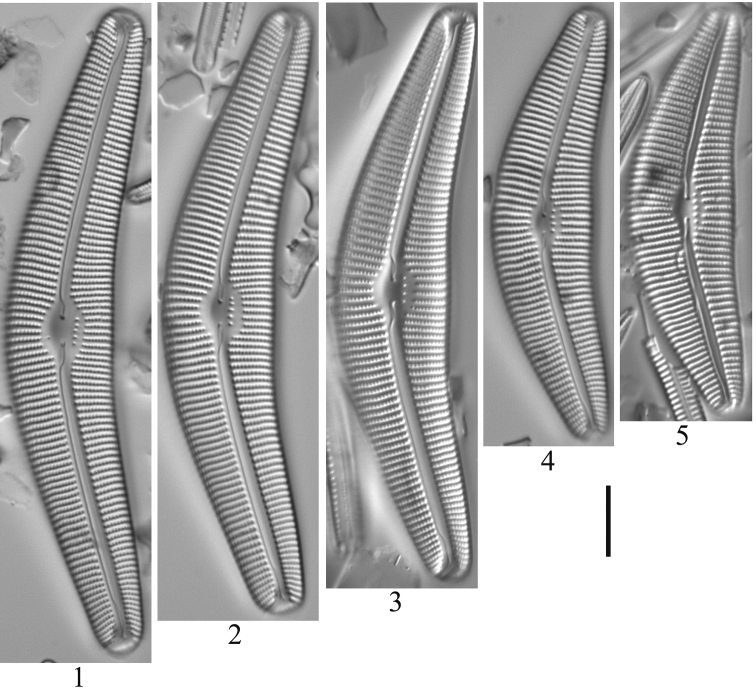
Hood. **1–5** *Cymbella arctica* (6904). Scale bar: 10 µm.

**Plate 117.**
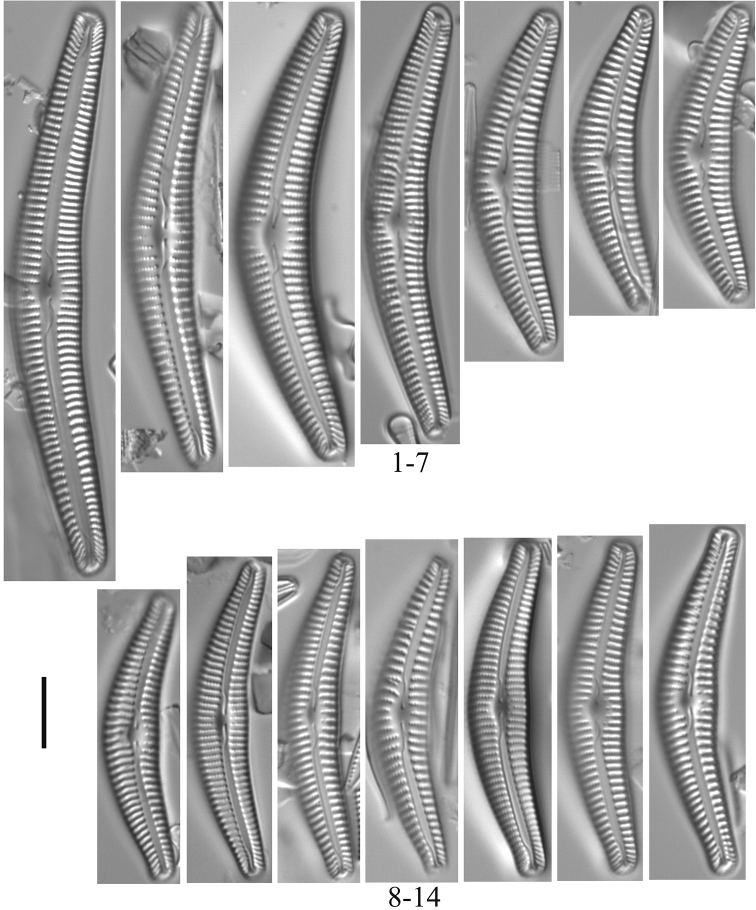
Hood. **1–14** *Cymbella cleve-eulerae* (6898, 6905, 6909). Scale bar: 10 µm.

**Plate 118.**
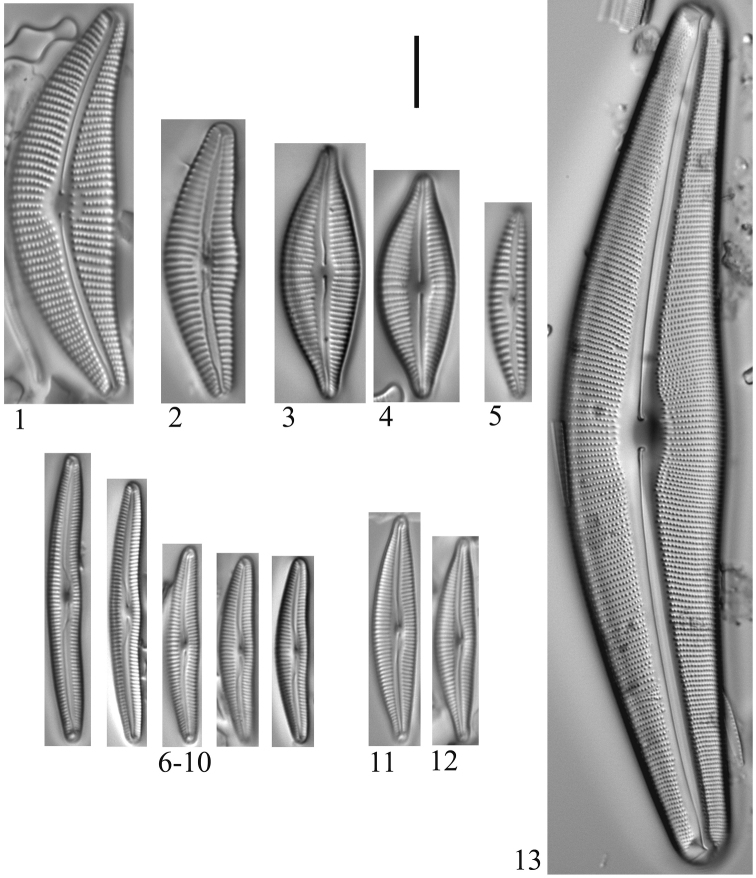
Hood. **1** *Cymbella proxima* (6904) **2** *Cymbella hantzschiana* (6898) **3, 4** *Cymbella designata* (6898, 6906) [= *Cymbopleura citriformis*] **5** *Cymbella* sp. (6904) **6–10** *Delicata canadensis* (6898, 6904) **11, 12** *Delicata delicatula* (6904) **13** *Cymbella aspera* (6912). Scale bar: 10 µm.

**Plate 119.**
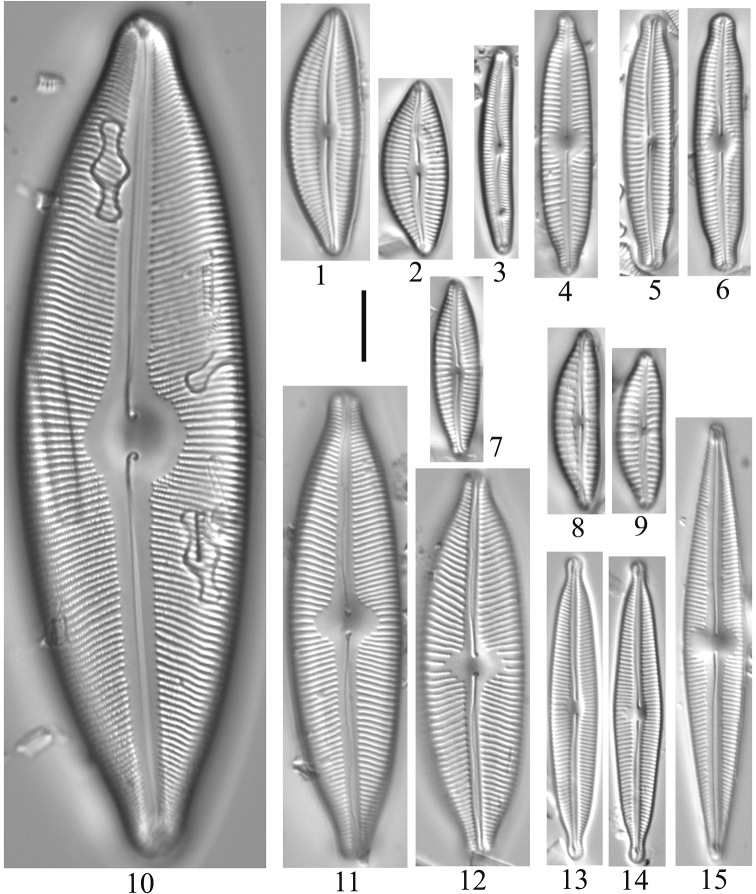
Hood. **1, 2** *Cymbopleura heilprinensis* (6907, 6909) **3** *C. tundraphila* (6904) **4** *C. hybrida* (6908) **5, 6** *C. fluminea* (6905, 6912) **7** *C. rupicola* (6909) **8, 9** *C.* sp. (6909) **10** *C. neoheteropleura* (6898) **11, 12** *C. tynnii* (6907, 6912) **13, 14** *C. angustata* (6900, 6904) **15** *C. stauroneiformis* (6898). Scale bar: 10 µm.

**Plate 120.**
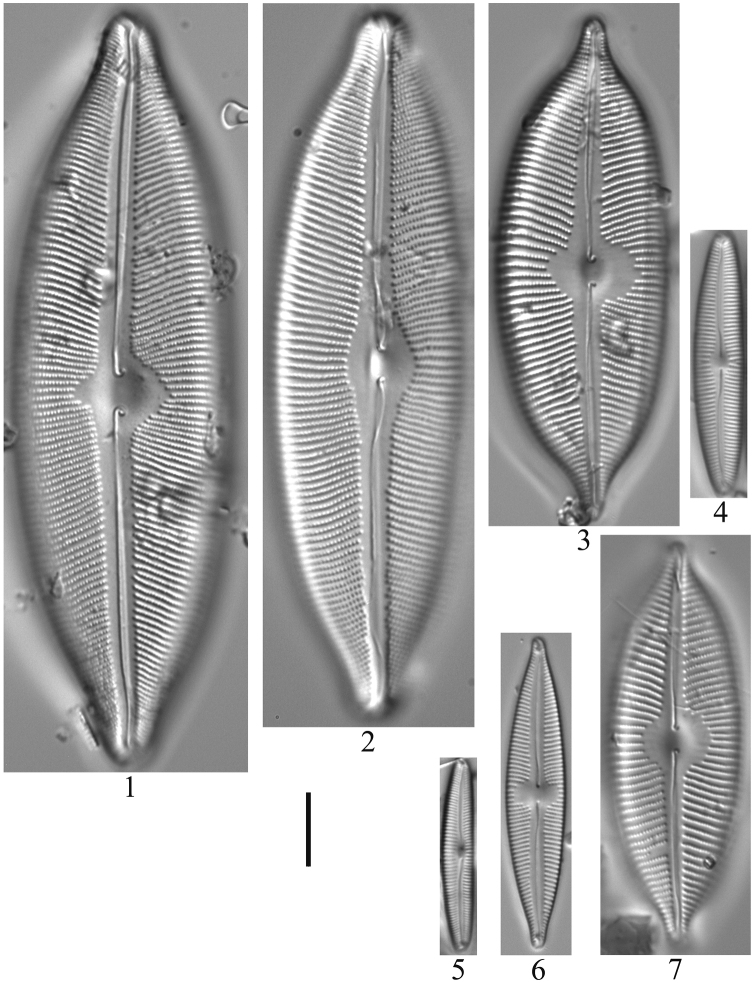
Hood. **1, 2** *Cymbopleura neoheteropleura* (6907) **3, 7** *C. apiculata* (6906, 6907) **4** C. incerta var. spitsbergensis [*C.* sp. PH] (6904) **5** *C. tundraphila* (6904) **6** *C. stauroneiformis* (6905). Scale bar: 10 µm.

**Plate 121.**
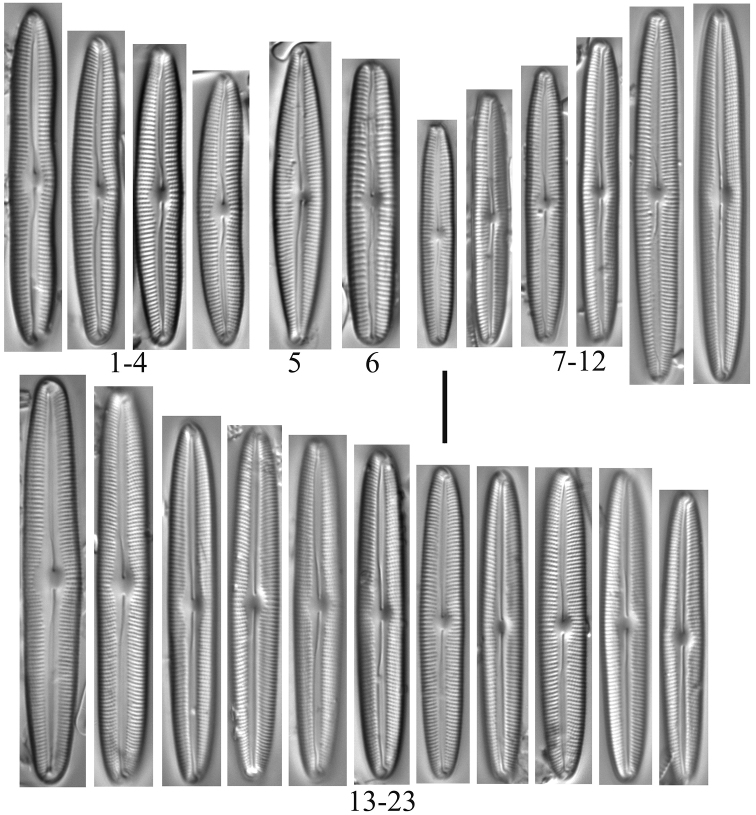
Hood. **1–4** Cymbopleura incerta var. spitsbergensis (6904, 6907, 6912) **5** *Cymbopleura incertiformis* (6905) **6** *Cymbopleura oblongata* (6907). **7–10** (**11, 12**?). Cymbopleura incertiformis var. linearis (6898, 6903, 6906, 6907, 6908) **13–23** *Encyonopsis* (*Cymbopleura*?) *grunowii* [= *Cymbella naviculacea*] (6899, 6904). Scale bar: 10 µm.

**Plate 122.**
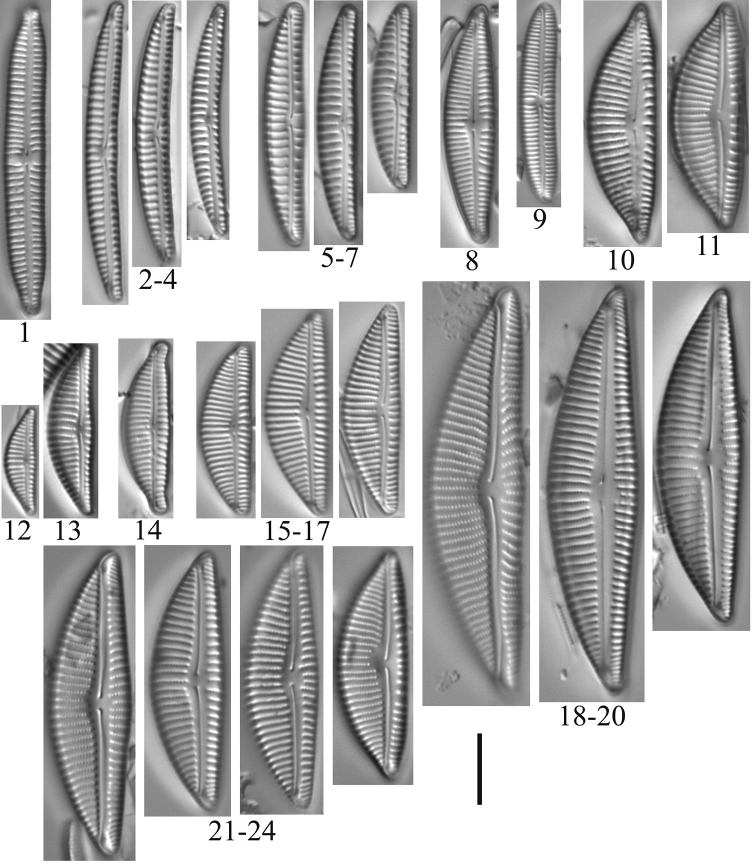
Hood. **1** *Encyonema sibericum* (6903, 6906) **2–4** *E. lunatum* (6898, 6907, 6912) **5–7** *E. paucistriatum* (6899, 6900, 6912) **8** *E. hebridicum* [*E. hebridicum* sensu lato PH] (6905) **9** *E. norvegicum* (6907, 6909) **10, 11** *E. elginense* (6907, 6908, 6909) **12, 13** *E. perminutum* (6898, 6902) **14** *E. ventricosum* **15–17** *E. silesiacum* (6902). (6898, 6904, 6913) **18–24** *E. hintzii* (6898, 6906, 6907, 6908, 6909, 6912). Scale bar: 10 µm.

**Plate 123.**
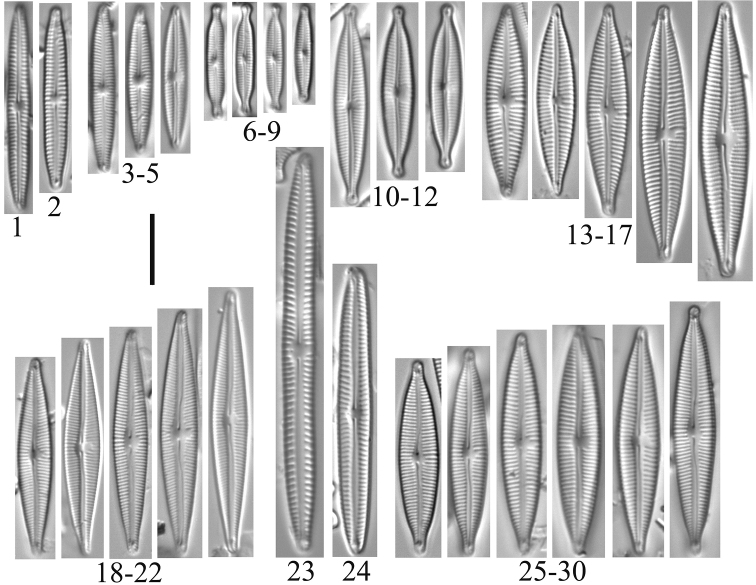
Hood. **1, 2** *Encyonopsis inuitorum* (6898, 6904) **3–5** *Encyonopsis falaisensis* (6904, 6909) **6–9** *Encyonopsis* sp. (6898, 6904) **10–12** *Encyonopsis descripta* (6904, 6909) **13–17** *Encyonopsis cesatiformis* (6899, 6904, 6905, 6907, 6912) **18–22** *Encyonopsis stafsholtii* (6904, 6905, 6907, 6908) **23** *Kurtkrammeria pseudoamphioxys* (6905) **24** *Kurtkrammeria neoamphioxys* (6905) **25–30** *Encyonopsis cesatii* (6904, 6905, 6907, 6912). Scale bar: 10 µm.

**Plate 124.**
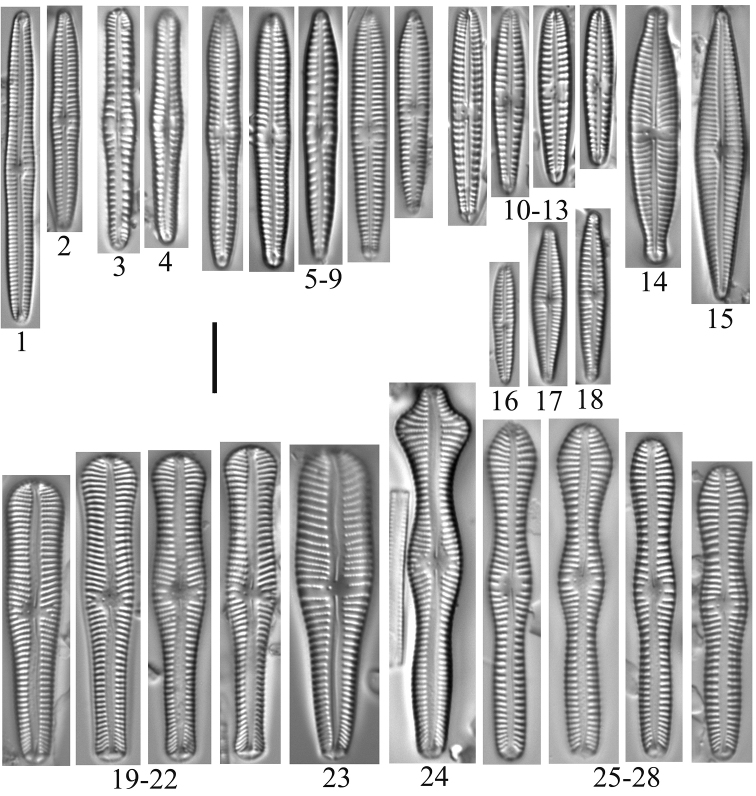
Hood. **1, 2** *Gomphonema astridae* (6906) **3, 4** *G.* sp. (6907) **5–9** *G. lagerheimii* sensu lato (6898, 6905, 6906, 6907, 6908, 6912) **10–13** *G. lateripunctatum* (6904) **14** *G. sarcophagus* [*G.* sp. PH] (6904) **15** *G. gracile* (6907) **16** *G.* sp. [cf. *G. pygmaeum*] (6912) **17** *G. exilissimum* (6913) **18** *G.* sp. [cf. *G. minusculum*] (6900) **19–22** *G.* sp. [cf. *G. capitatum*] (6904, 6906, 6913) **23** *G. laticollum* (6898) **24** *G. acuminatum* (6904) **25–28** *G.* sp. [cf. *G. interpositum*] [cf. *G. montanum* PH] (6907, 6908, 6909). Scale bar: 10 µm.

**Plate 125.**
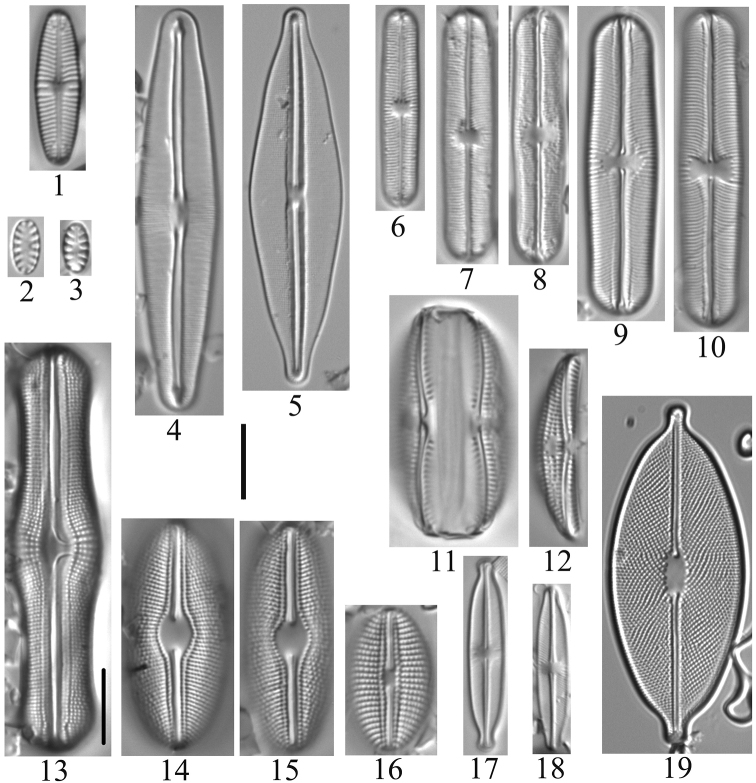
Hood. **1** *Geissleria moseri* (6898) **2, 3** *Hygropetra balfouriana* (6904) **4** *Frustulia vulgaris* (6913) **5** *Frustulia crassinervia* (6899, 6900, 6905) **6** *Sellaphora* sp. (6909) **7** *Sellaphora rectangularis* (6909) **8** *Sellaphora pupula* (6909) **9, 10** *Sellaphora parapupula* (6903, 6907, 6909) **11, 12** *Amphora copulata* (6908, 6912) **13** *Muelleria bachmannii* (6908) **14, 15** *Diploneis arctica* [D. ovalis ssp. arctica PH] (6907, 6908) **16** *Diploneis parma* (6908) **17** *Kobayasiella micropunctata* (6903, 6905) **18** *Kobayasiella jaagii* [*K. micropunctata* PH] (6907) **19** *Decussata placenta* (6898). Scale bars: 10 µm.

**Plate 126.**
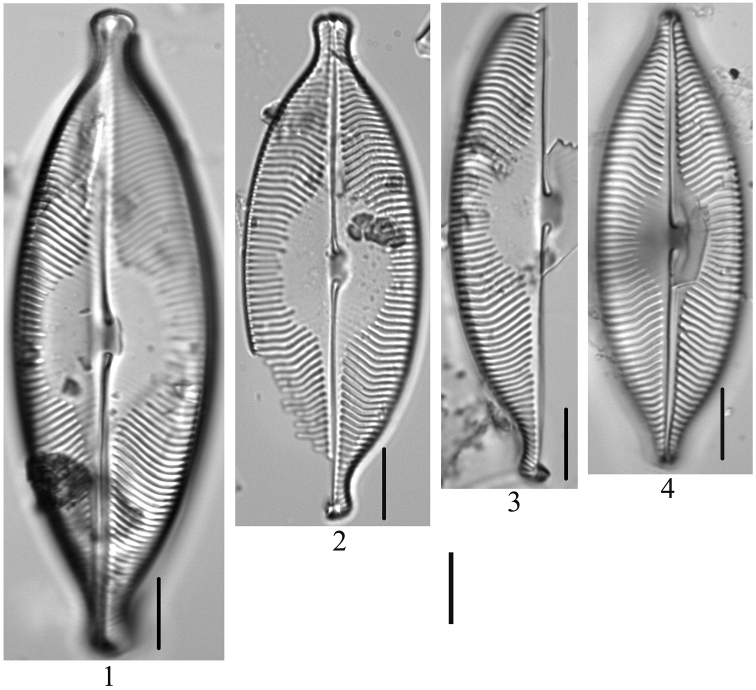
Hood. **1–4** *Pinnuavis* sp. [cf. Pinnuavis elegans f. crowbillensis PH] (6912). Scale bars: 10 µm.

**Plate 127.**
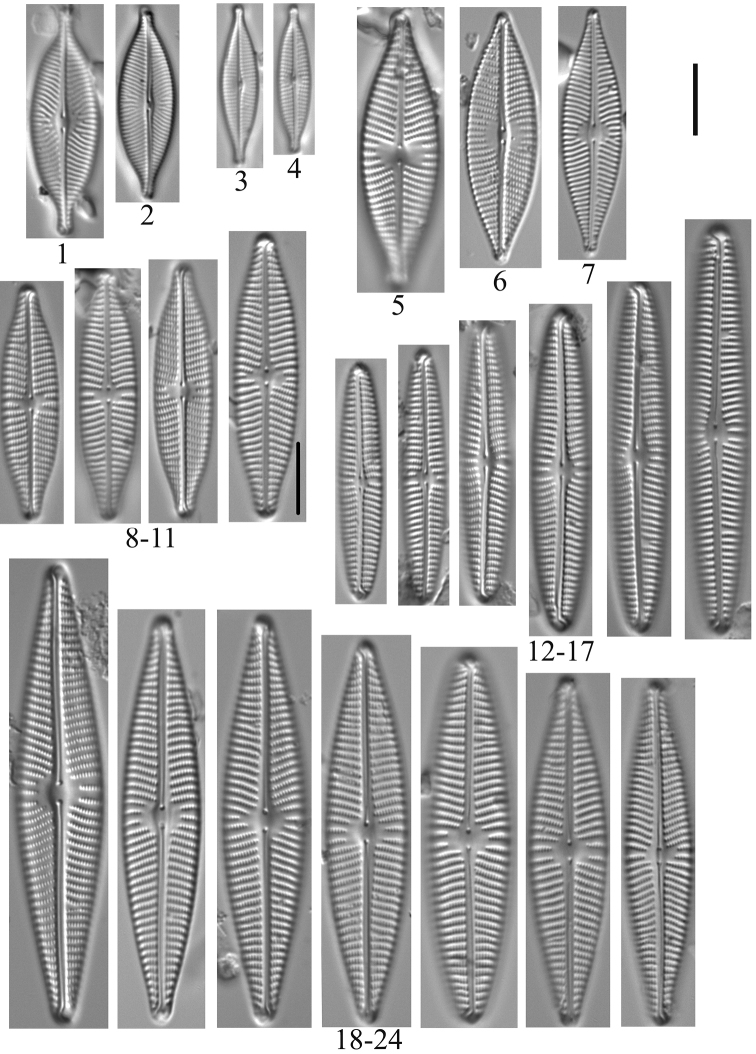
Hood. **1, 2** *Navicula salinarum* (6912, 6913) **3** *N. gregaria* (6913) **4** *N. vandamii* (6913) **5** N. hanseatica subsp. hanseatica (6911) **6** N. hanseatica subsp. circumarctica (6912) **7** *N. trivialis* (6913) **8–11** *N. slesvicensis* (6912, 6913) **12–17** *N. eidrigiana* (6906, 6912) **18–24** *N. vaneei* (6904, 6906, 6912). Scale bars: 10 µm.

**Plate 128.**
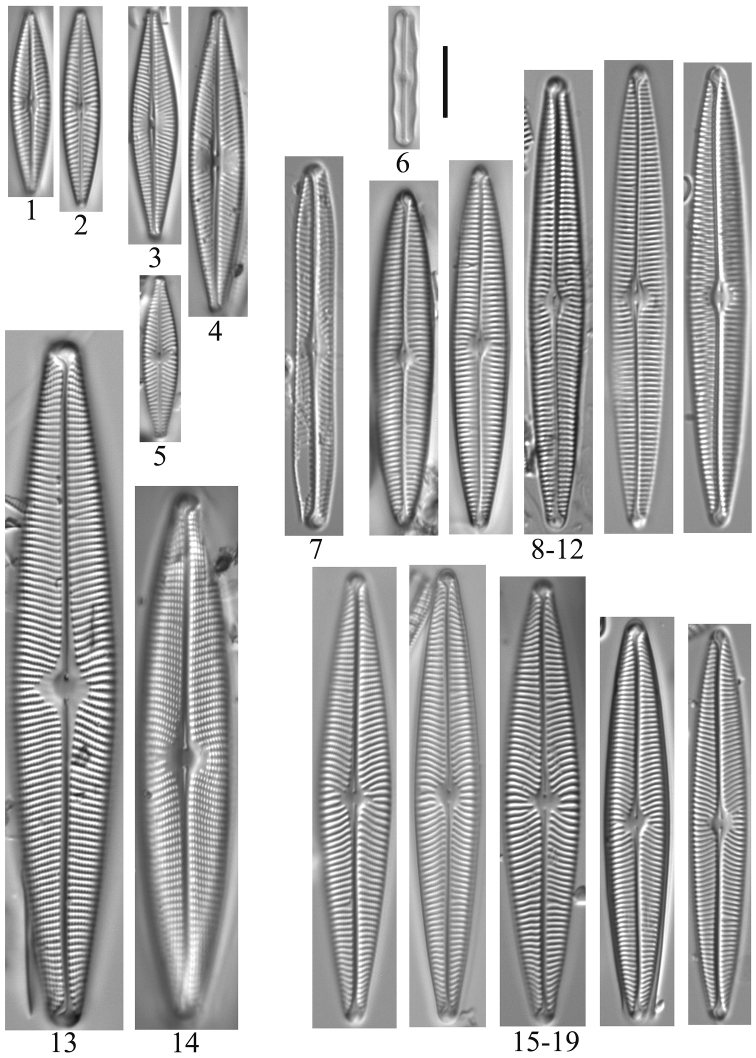
Hood. **1, 2** *Navicula notha* (6898, 6909) **3–5** *N. cryptocephala* (6898, 6906, 6912, 6913) **6** *N. tridentula* (6909) **7** *N. angusta* (6906) **8–12** N. tripunctata var. arctica (6898, 6906, 6907, 6912, 6908) **13, 14** *N. vulpina* (6898, 6906) **15–19** *N. radiosa* (6904, 6906, 6912, 6913). Scale bar: 10 µm.

**Plate 129.**
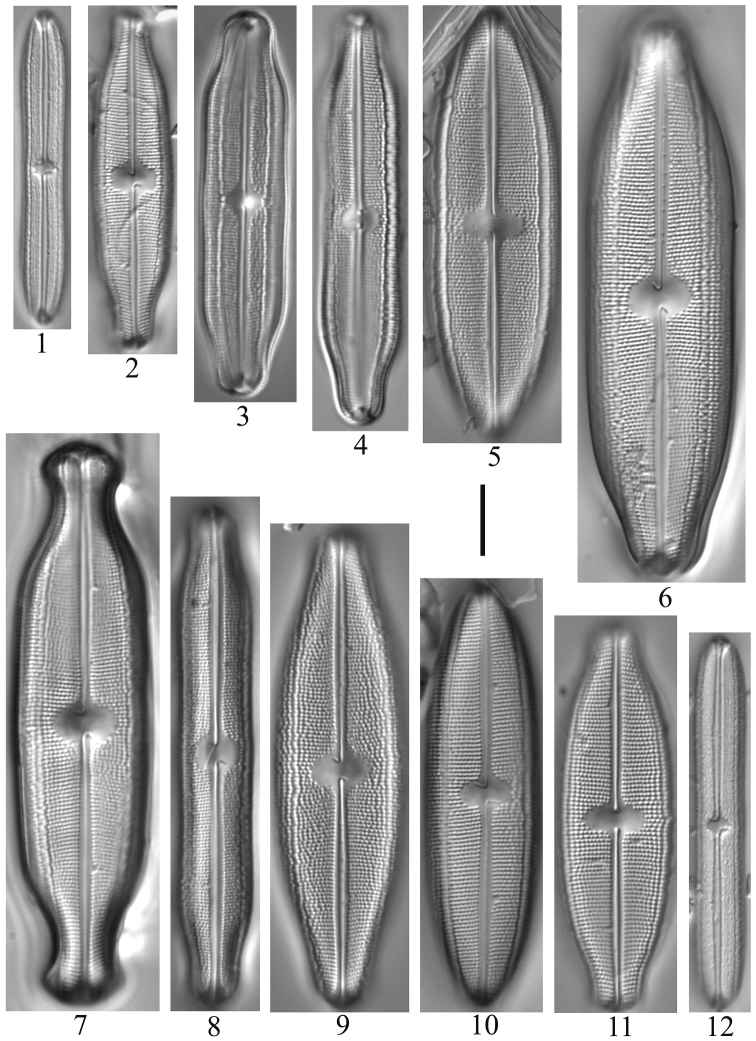
Hood. **1, 12** *Neidium bisulcatum* (6899, 6908, 6912) **2** *N. affine* [*N. potapovae* PH] (6908) **3, 4** *N.* sp. [cf. *N. septentrionale*] (6902, 6912) **5, 10** *N. fossum* (6905, 6908) **6, 9** *N. ampliatum* [*N.* sp. PH] (6899, 6906) **7** *N. productum* (6898) **8** *N.* sp. [cf. *N. septentrionale*] (6907) **11** *N. temperei* (6907, 6909). Scale bar: 10 µm.

**Plate 130.**
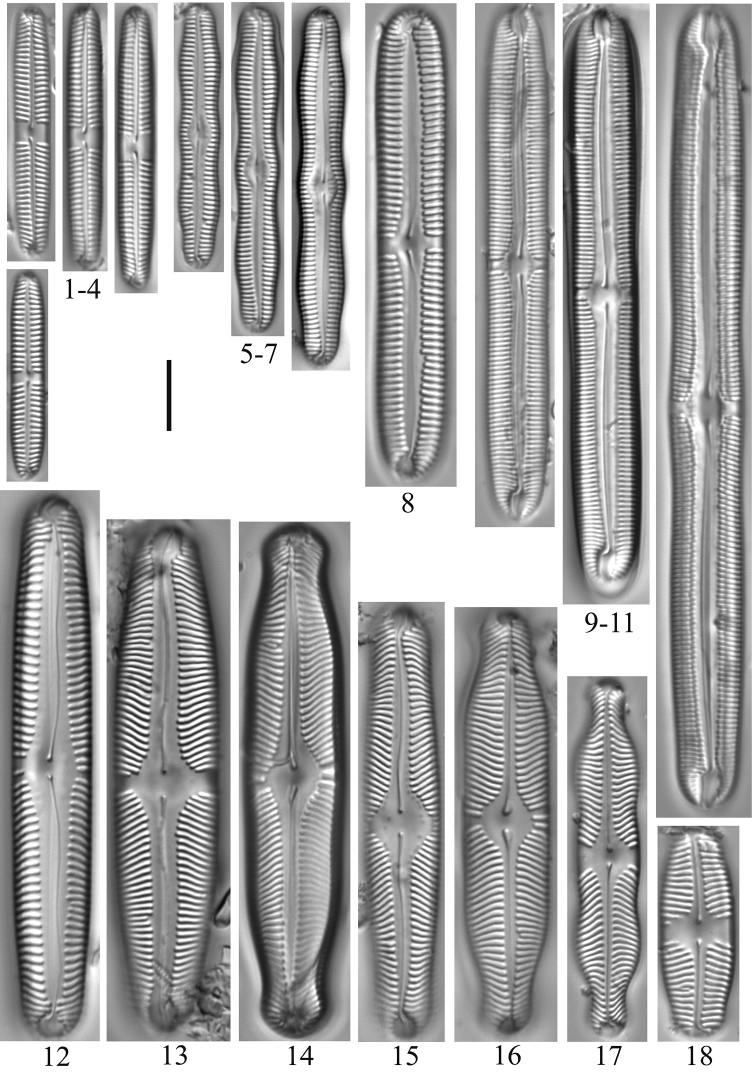
Hood. **1–4** *Pinnularia* sp. [cf. *P. sinistra*] (6898, 6899, 6901, 6903, 6906) **5–7** *P. subpulchra* (6899) **8** *P. crucifera* (6909) **9–11** *P. spitsbergensis* (6898, 6906, 6907, 6908) **12** *P.* sp. [cf. *P. lokana*] [cf. *P. gibba* sensu lato PH] (6906, 6907, 6912) **13** *P. divergens* (6899) **14** *P. biceps* (6898) **15** *P. decrescens* (6899, 6907) **16** *P. anglica* (6906, 6907) **17** *P. grunowii* (6906, 6907) **18** *P. birnirkiana* (6912). Scale bar: 10 µm.

**Plate 131.**
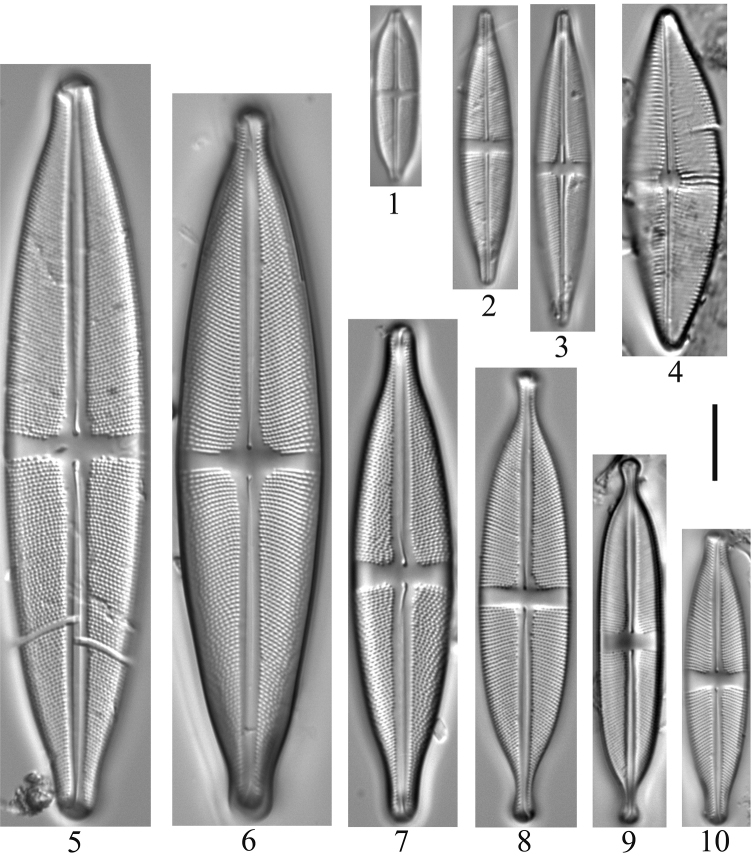
Hood. **1** *Stauroneis livingstonii* (6913) **2, 3** Stauroneis smithii var. incisa [*S.* sp. PH] (6912) **4** *Staurophora* sp. (6912) **5** *Stauroneis gracilis* (6906) **6** *Stauroneis kuelbsii* (6898, 6903, 6906) **7** *Stauroneis hyperborea* [PH] (6906, 6909) **8** *Stauroneis amphicephala* (6903, 6906) **9** *Stauroneis reichardtii* (6898) **10** *Stauroneis jarensis* (6903, 6904, 6906, 6907). Scale bar: 10 µm.

**Plate 132.**
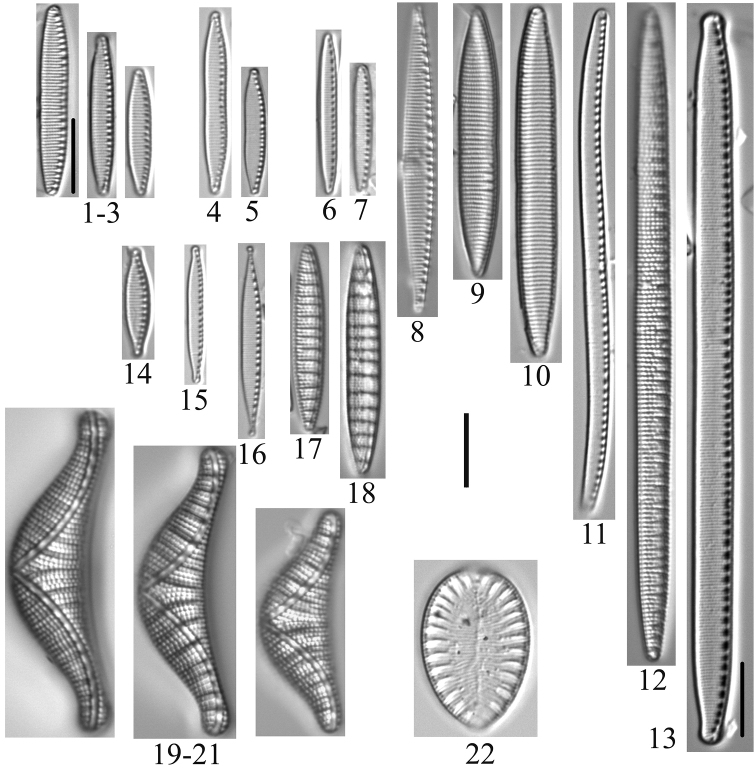
Hood. **1–3** *Nitzschia alpina* (6898, 6904) **4, 5** *Nitzschia frustulum* (6908, 6912) **6, 7** *Nitzschia acidoclinata* (6898, 6899) **8** *Nitzschia* sp. [cf. *N. fossilis*] (6911) **9, 10** *Nitzschia angustata* (6898, 6904, 6909) **11** *Nitzschia nana* (6912) **12** Denticula kuetzingii var. rumrichae (6906) **13** Nitzschia regula var. robusta (6904) **14** *Nitzschia* sp. [cf. *N. bacillum*] (6898) **15** *Nitzschia perminuta* (6912) **16** *Nitzschia suchlandtii* [*N. gracilis* sensu lato PH] (6900) **17** *Denticula kuetzingii* (6898, 6904) **18** *Denticula valida* [*D. kuetzingii* PH] (6909) **19–21** *Epithemia smithii* (6898, 6902, 6908) **22** *Surirella brebissonii* (6913). Scale bars: 10 µm.

## Appendix 2: Index to diatom images

**Table 7.** List of taxa and index to plates.

Taxa	Waterton	Haida Gwaii	Clearwater	Coppermine	Baillie/Back	Hood
*Achnanthidium* Kützing	3				77	
*Achnanthidium duthiei* (Sreenivasa) Edlund						110
*Achnanthidium kriegeri* (Krasske) Hamilton, Antoniades & Siver		24				110
*Achnanthidium minutissimum* (Kützing) Czarnecki		24				110
*Actinella punctata* Lewis		20				
*Adlafia minuscula* (Grunow) Lange-Bertalot			46			
*Amphora* Ehrenberg in Kützing	5					
*Amphora copulata* (Kützing) Schoeman & Archibald	5					125
*Amphora lange-bertalotii* Levkov & Metzeltin				64		
*Amphora pediculus* (Kützing) Grunow	5					
*Amphora thumensis* (Mayer) Cleve-Euler	5					
*Aneumastus rostratus* (Hustedt) Lange-Bertalot	4					
*Aneumastus tusculus* (Ehrenberg) Mann & Stickle	4			59		114
*Aulacoseira* Thwaites		18				
*Aulacoseira alpigena* (Grunow) Krammer	1		38			
*Aulacoseira ambigua* (Grunow) Simonsen			38			
*Aulacoseira crassipunctata* Krammer		18				
*Aulacoseira italica* (Ehrenberg) Simonsen	1		38			
*Aulacoseira nivalis* (W. Smith) English & Potapova	1					
*Aulacoseira subarctica* (O. Müller) Haworth			38		77	
*Boreozonacola olympica* (Sovereign) Lange-Bertalot et al.			46			
*Brachysira* Kützing		25		59	89	114
*Brachysira arctoborealis* Wolfe & Kling						114
*Brachysira brebissonii* Ross in Hartley	3	25				
*Brachysira calcicola* Lange-Bertalot						114
*Brachysira microcephala* (Grunow) Compére	3			59		114
*Brachysira ocalanensis* Shayler & Siver		25				
*Brachysira procera* Lange-Bertalot & Moser		25				
*Brachysira styriaca* (Grunow) Ross in Hartley						114
*Brachysira zellensis* (Grunow) Round & Mann				59		
*Caloneis* Cleve	12, 13			70	101	
*Caloneis bacillum* (Grunow) Cleve		33				
*Caloneis falcifera* Lange-Bertalot, Genkal & Vekhov				70		
*Caloneis fasciata* (Lagerstedt) Cleve				70		
*Caloneis fusus* Hamilton & Antoniades in Antoniades et al.				70	101	114
*Caloneis obtusa* (W. Smith) Cleve				70		114
*Caloneis schumanniana* (Grunow) Cleve	13					
*Caloneis silicula* (Ehrenberg) Cleve					101	114
*Caloneis tenuis* (Gregory) Krammer	12, 13	33		70		114
*Caloneis undulata* Skvortzow & Meyer	13					
*Cavinula davisiae* Bahls	3					
*Cavinula jaernefeltii* (Hustedt) Mann & Stickle					89	
*Cavinula pseudoscutiformis* (Hustedt) Mann & Stickle		24	46	59		
*Cavicula scutiformis* (Grunow) Mann & Stickle					89	
*Chamaepinnularia bergeri* (Krasske) Lange-Bertalot					101	
*Cocconeis* Ehrenberg		24				
*Cocconeis placentula* Ehrenberg		24				
*Cocconeis pseudothumensis* Reichardt	3					
*Cocconeis rugosa* Sovereign						110
*Coscinodiscus* Ehrenberg		18				
*Craticula* Grunow					89	
*Craticula buderi* (Hustedt) Lange-Bertalot			46			
*Craticula cuspidata* (Kützing) Mann			46			
*Craticula johnstoniae* Bahls	4					
*Craticula sardiniana* Bahls	4					
*Cyclotella distinguenda* Hustedt	1					
*Cymatopleura solea* (Brébisson) W. Smith				76		
*Cymbella* Agardh			43	60		118
*Cymbella alpestris* Krammer	5					
*Cymbella americana* A. Schmidt	7		44			
*Cymbella arctica* (Lagerstedt) A. Schmidt						115, 116
*Cymbella aspera* (Ehrenberg) H. Peragallo			43			118
*Cymbella botellus* (Lagerstedt) A. Schmidt				60		115
*Cymbella cleve-eulerae* Krammer				60, 61	92	117
*Cymbella cosleyi* Bahls	5					
*Cymbella designata* Krammer				63	92	118
*Cymbella excisiformis* Krammer	5					
*Cymbella hantzschiana* Krammer	5		43			118
*Cymbella krammeri* Bahls				61	92	115
*Cymbella naviculacea* Grunow			43			121
*Cymbella neocistula* Krammer	5	28		61	92	
Cymbella neocistula var. islandica Krammer	5					
*Cymbella neoleptoceros* Krammer	5					
*Cymbella proxima* Reimer in Patrick & Reimer	5	28			92	118
*Cymbella stigmaphora* Østrup	5					
*Cymbella subturgidula* Krammer				60		
*Cymbopleura* Krammer		27			94	119
*Cymbopleura amphicephala* (Naegeli) Krammer	7			62		
*Cymbopleura anglica* (Lagerstedt) Krammer	7				94	
*Cymbopleura angustata* (W. Smith) Krammer	7			62	94	119
*Cymbopleura apiculata* Krammer	7		44	63	94	120
*Cymbopleura austriaca* (Grunow) Krammer				63		
*Cymbopleura citriformis* Krammer				63	92	118
*Cymbopleura crassipunctata* Krammer			44			
*Cymbopleura fluminea* (Patrick & Freese) Lange-Bertalot & Krammer		27	44		94	119
*Cymbopleura geofriedii* Reichardt in Krammer				62		
*Cymbopleura heilprinensis* Foged	7			63	92	119
*Cymbopleura hybrida* (Grunow) Krammer	7			62		119
*Cymbopleura incerta* (Grunow) Krammer	7			62		
Cymbopleura incerta var. spitsbergensis Krammer				62	94	120, 121
*Cymbopleura incertiformis* Krammer						121
Cymbopleura incertiformis var. linearis (Fontell) Krammer				62	94	121
*Cymbopleura lapponica* (Grunow) Krammer	7					
*Cymbopleura lata* (Grunow) Krammer	7			63		
*Cymbopleura naviculiformis* (Auerswald) Krammer			44			
*Cymbopleura neoheteropleura* Krammer					93, 94	119, 120
*Cymbopleura oblongata* Krammer	7			62		121
*Cymbopleura rainierensis* (Sovereign) Bahls	7		44			
*Cymbopleura rupicola* (Grunow) Krammer	7			62		119
*Cymbopleura similiformis* Krammer	7					
*Cymbopleura stauroneiformis* (Lagerstedt) Krammer			44	62	94	119, 120
*Cymbopleura subaequalis* (Grunow) Krammer	7					
*Cymbopleura subcuspidata* (Krammer) Krammer	7	27	44			
*Cymbopleura tundraphila* Bahls				62		119, 120
*Cymbopleura tynnii* (Krammer) Krammer				63	94	119
*Decussata placenta* (Ehrenberg) Lange-Bertalot & Metzeltin		26				125
*Delicata alpestris* (Krammer) Bahls		28				
*Delicata canadensis* Bahls				64	91	118
*Delicata delicatula* (Kützing) Krammer	5					118
*Denticula* Kützing				75, 76		
*Denticula kuetzingii* Grunow	17	37		76		132
Denticula kuetzingii var. rumrichae Krammer						132
*Denticula tenuis* Kützing				76		
*Denticula valida* (Pedicino) Grunow				76		132
*Diadesmis perpusilla* (Grunow) Mann	3					
*Diatoma moniliformis* Kützing	2					
*Diatoma tenuis* Agardh		19		56		
*Diatomella balfouriana* Greville	3					
*Didymosphenia geminata* (Lyngbye) M. Schmidt	8					
*Diploneis arctica* (Lange-Bertalot) Lange-Bertalot & Fuhrmann						125
*Diploneis elliptica* (Kützing) Cleve		24				
*Diploneis finnica* (Ehrenberg) Cleve		24				
*Diploneis krammeri* Lange-Bertalot & Reichardt				59		
*Diploneis oblongella* (Naegeli in Kützing) Cleve-Euler	10					
*Diploneis oculata* (Brébisson) Cleve	10					
*Diploneis parma* Cleve	10					125
*Diploneis pseudovalis* Hustedt	10					
*Discostella pseudostelligera* (Hustedt) Houk & Klee						109
*Encyonema* Kützing	6	30				
*Encyonema elginense* (Krammer) Mann						122
*Encyonema fogedii* Krammer	6	29	45			
*Encyonema hamsherae* Winter & Bahls	6					
*Encyonema hebridicum* (Gregory) Grunow in Cleve & Möller	6	30	45	64		122
*Encyonema hintzii* Krammer	6			64	90	122
*Encyonema latum* Krammer		29				
*Encyonema lunatum* (W. Smith) Van Heurck					90	122
*Encyonema minutiforme* Krammer		29				
*Encyonema minutum* (Hilse in Rabenhorst) Mann	6		45			
*Encyonema neogracile* Krammer		30	45		90	
*Encyonema norvegicum* (Grunow) Mayer	6			64		122
*Encyonema paucistriatum* (Cleve-Euler) Mann				64	90	122
*Encyonema pergracile* Krammer		30				
*Encyonema perminutum* Krammer						122
*Encyonema perpusillum* (Cleve-Euler) Mann		30				
*Encyonema procerum* Krammer	6					
*Encyonema sibericum* Krammer					90	122
*Encyonema silesiacum* (Bleisch) Mann		29		64		122
*Encyonema temperei* Krammer	6					
*Encyonema ventricosum* (Agardh) Grunow in A. Schmidt et al.	6			64	90	122
*Encyonema vulgare* Krammer		29		64	90	
*Encyonema willeyorum* Bahls					90	
*Encyonopsis* Krammer	6			65	91	123
*Encyonopsis alpina* Krammer & Lange-Bertalot	6					
*Encyonopsis angusta* Krammer & Lange-Bertalot				65		
*Encyonopsis cesatiformis* Krammer				65	91	123
*Encyonopsis cesatii* (Rabenhorst) Krammer	12	30			91	123
*Encyonopsis czarneckii* Bahls	12				91	
*Encyonopsis descripta* (Hustedt) Krammer				65		123
*Encyonopsis falaisensis* (Grunow) Krammer						123
*Encyonopsis grunowii* Krammer			43			121
*Encyonopsis inuitorum* Bahls				65	91	123
*Encyonopsis lacuscaerulei* Bahls				65		
*Encyonopsis montana* Bahls	6					
*Encyonopsis neerlandica* Van de Vijver et al.				65		
*Encyonopsis stafsholtii* Bahls		30		65	91	123
*Encyonopsis subminuta* Krammer & Reichardt	6	30				
*Entomoneis paludosa* (W. Smith) Reimer		37	53			
*Epithemia* Brébisson	17					
*Epithemia adnata* (Kützing) Brébisson	17					
*Epithemia argus* (Ehrenberg) Kützing	17		53			
*Epithemia smithii* Carruthers	17	36		76		132
Epithemia turgida var. granulata (Ehrenberg) Brun	17					
*Eucocconeis alpestris* (Brun) Lange-Bertalot	3					110
*Eucocconeis depressa* (Cleve) Lange-Bertalot					77	110
*Eucocconeis flexella* (Kützing) Meister		24		56	77	110
*Eucocconeis laevis* (Østrup) Lange-Bertalot				56		110
*Eunotia* Ehrenberg		21, 22, 23	39, 40, 41	57	78, 83, 85	
*Eunotia altimontana* Lange-Bertalot, Pavlov & Levkov				57		
*Eunotia ambivalens* Lange-Bertalot & Tagliaventi			40, 41		87	
*Eunotia arcofallax* Lange-Bertalot		23				
*Eunotia arcubus* Nörpel-Schempp & Lange-Bertalot				58	78	113
*Eunotia arculus* Lange-Bertalot & Nörpel-Schempp		23				
*Eunotia arcus* (Ehrenberg) W. Smith	2			57	78	113
*Eunotia bidens* Ehrenberg			39			111
*Eunotia bidentula* W. Smith		20				
*Eunotia bilunaris* (Ehrenberg) Schaarschmidt		22			88	
*Eunotia boreoalpina* Lange-Bertalot & Nörpel-Schempp					87	
*Eunotia boreotenuis* Nörpel-Schempp & Lange-Bertalot		23			88	112
*Eunotia botuliformis* Wild, Nörpel-Schempp & Lange-Bertalot	2	23			88	112
*Eunotia braendlei* Lange-Bertalot & Werum					83	
*Eunotia circumborealis* Lange-Bertalot & Nörpel-Schempp			39		85	111
*Eunotia curtagrunowii* Nörpel-Schempp & Lange-Bertalot					78, 82	112
*Eunotia denticulata* (Brébisson) Rabenhorst					83	
*Eunotia diadema* Ehrenberg		21			84	
*Eunotia diodon* Ehrenberg					85	111
*Eunotia dorofeyukae* Lange-Bertalot & Kulikovskiy						111
*Eunotia elegans* Østrup					83	
*Eunotia eurycephala* (Grunow) Nörpel-Schempp & Lange-Bertalot			40		87	
*Eunotia excelsa* (Krasske) Nörpel				58		
*Eunotia exigua* (Brébisson) Rabenhorst						112
*Eunotia faba* Ehrenberg			40			112
*Eunotia fallax* A. Cleve			42			112
*Eunotia flexuosa* (Brébisson in Kützing) Kützing		22	40			
*Eunotia glacialis* Meister						112
*Eunotia groenlandica* Nörpel-Schempp & Lange-Bertalot			42			
*Eunotia herkiniensis* Grunow						111
*Eunotia implicata* Nörpel-Schempp, Alles & Lange-Bertalot						111
*Eunotia incisa* Gregory		23	40		87	112
*Eunotia intermedia* (Krasske) Nörpel-Schempp & Lange-Bertalot						112
*Eunotia islandica* Østrup		20		57	85	
*Eunotia juettnerae* Lange-Bertalot	2				87	
*Eunotia julma* Lange-Bertalot		22	41			112
*Eunotia lapponica* Grunow					83	
*Eunotia latitaenia* Kobayasi, Ando & Nagumo					87	
*Eunotia lewisii* Siver & Hamilton		23				
*Eunotia maior* (W. Smith) Rabenhorst						112
*Eunotia mayamae* Lange-Bertalot, Bak & Witkowski					78, 82	
*Eunotia mertensiae* Lange-Bertalot		22				
*Eunotia metamonodon* Lange-Bertalot		21			81	112
*Eunotia mihoi* Lange-Bertalot, Pavlov & Levkov						111
*Eunotia minor* (Kützing) Grunow		23	39		83	113
*Eunotia monnieri* Lange-Bertalot & Tagliaventi			41			
*Eunotia mucophila* (Lange-Bertalot et al.) Lange-Bertalot			41	57		112
*Eunotia naegelii* Migula		22			88	
*Eunotia neoborealis* Lange-Bertalot		21			83	
*Eunotia neocompacta* Mayama					83	
*Eunotia nymanniana* Grunow			41			112
*Eunotia paludosa* Grunow		23			88	
*Eunotia paralleladubia* Lange-Bertalot & Mayama					80	112
*Eunotia paratridentula* Lange-Bertalot & Kulikovskiy			39			
*Eunotia pectinalis* (Kützing) Rabenhorst					79	
*Eunotia perminuta* (Grunow) Patrick					85	111
*Eunotia praerupta* Ehrenberg			42		78	113
*Eunotia pseudoflexuosa* Hustedt					87	112
*Eunotia pseudogroenlandica* Lange-Bertalot & Tagliaventi					88	
*Eunotia pseudopapilio* Lange-Bertalot & Nörpel-Schempp					86	111
*Eunotia pseudopectinalis* Hustedt					79	112
*Eunotia rhomboidea* Hustedt	2				88	
*Eunotia sarek* Berg					86	111
*Eunotia scandiorussica* Kulikovskiy et al.					88	
*Eunotia semicircularis* (Ehrenberg) Lange-Bertalot & Metzeltin					84	111
*Eunotia septentrionalis* Østrup					83	
*Eunotia serra* Ehrenberg			39			
*Eunotia silesioscandica* Lange-Bertalot & Sienkiewicz					83	
*Eunotia soleirolii* (Kützing) Rabenhorst					78	
*Eunotia subarcuatoides* Alles, Nörpel & Lange-Bertalot		23			88	
*Eunotia suecica* A. Cleve					85	111
*Eunotia superbidens* Lange-Bertalot		21			85	111
*Eunotia superpaludosa* Lange-Bertalot		23	42			
*Eunotia tetraodon* Ehrenberg		21				
*Eunotia triodon* Ehrenberg			39			
*Eunotia ursamaioris* Lange-Bertalot & Nörpel-Schempp			42	57	83	113
*Eunotia valida* Hustedt					88	
*Fallacia* Stickle & Mann					89	
*Fragilaria* Lyngbye	2		38	56		109
*Fragilaria capucina* Desmazières		18		56		
Fragilaria capucina var. rumpens (Kützing) Lange-Bertalot		18				
*Fragilaria crotonensis* Kitton	2					109
*Fragilaria nanana* Lange-Bertalot	2					
*Fragilaria sepes* Ehrenberg						109
*Fragilaria tenera* (W. Smith) Lange-Bertalot	2					
*Fragilaria vaucheriae* (Kützing) Petersen		18		56		
*Fragilariforma constricta* (Ehrenberg) Williams & Round					77	
*Fragilariforma nitzschioides* (Grunow) Lange-Bertalot			38			
*Fragilariforma polygonata* (Cleve-Euler) Kingston et al.		18				
*Frustulia amosseana* Lange-Bertalot in Rumrich et al.	4					
*Frustulia crassinervia* (Brébisson) Lange-Bertalot & Krammer		26			89	125
*Frustulia quadrisinuata* Lange-Bertalot		26				
*Frustulia saxonica* Rabenhorst	4	26	47			
*Frustulia vulgaris* (Thwaites) De Toni						125
*Geissleria* Lange-Bertalot & Metzeltin	3				89	
*Geissleria moseri* Metzeltin, Witkowski & Lange-Bertalot					89	125
*Geissleria paludosa* (Hustedt) Lange-Bertalot & Metzeltin	3					
*Geissleria schoenfeldii* (Hustedt) Lange-Bertalot & Metzeltin					89	
*Geissleria similis* (Krasske) Lange-Bertalot & Metzeltin	3					
*Geissleria tectissima* (Lange-Bertalot) Lange-Bertalot					89	
*Gomphoneis geitleri* Kociolek & Stoermer	9					
*Gomphonema* Ehrenberg	8, 9	31	45	67	95	124
*Gomphonema acidoclinatum* Lange-Bertalot & Reichardt	8					
*Gomphonema acuminatum* Ehrenberg						124
*Gomphonema affine* Kützing	8					
*Gomphonema anglicum* Ehrenberg	9					
*Gomphonema angusticephalum* Reichardt & Lange-Bertalot	9					
*Gomphonema astridae* Reichardt & Lange-Bertalot					95	
*Gomphonema auritum* Braun	8		45			
*Gomphonema brebissonii* Kützing	9		45	67	95	
*Gomphonema caperatum* Ponader & Potapova				66		
*Gomphonema capitatum* Ehrenberg	9			67	95	124
*Gomphonema citera* Hohn & Hellerman		31				
*Gomphonema clavatum* Ehrenberg		31				
*Gomphonema coronatumaceum* Bahls				67	95	
*Gomphonema distans* (Cleve-Euler) Lange-Bertalot & Reichardt			45	66		
*Gomphonema duplipunctatum* Lange-Bertalot & Reichardt		31	45			
*Gomphonema exilissimum* (Grunow) Lange-Bertalot & Reichardt	8	31				124
*Gomphonema gracile* Ehrenberg		31			95	124
*Gomphonema hebridense* Gregory	8					
*Gomphonema insigniforme* Reichardt & Lange-Bertalot			45			
*Gomphonema interpositum* Reichardt				67		124
*Gomphonema kobayasii* Kociolek & Kingston		31				
*Gomphonema lagerheimii* A. Cleve				66	95	124
*Gomphonema lateripunctatum* Reichardt & Lange-Bertalot				66, 71	95	124
*Gomphonema laticollum* Reichardt	9	31				124
*Gomphonema longilineare* Reichardt	8					
*Gomphonema louisiananum* Kalinsky		31				
*Gomphonema micropus* Kützing		31	45	66		
*Gomphonema minusculum* Krasske		31				124
*Gomphonema minutum* (Agardh) Agardh	8					
*Gomphonema multipunctatum* Bahls	8					
*Gomphonema nathorstii* Foged				66		
*Gomphonema pala* Reichardt	9					
*Gomphonema parvulum* Kützing			45			
*Gomphonema procerum* Reichardt & Lange-Bertalot		31				
*Gomphonema pumilum* (Grunow) Reichardt & Lange-Bertalot		31				
*Gomphonema pygmaeum* Kociolek & Stoermer	8					124
*Gomphonema sarcophagus* Gregory	8					124
*Gomphonema subclavatum* Grunow	8		45			
*Gomphonema subtile* Ehrenberg	9			67		
Gomphonema subtile var. sagitta (Schumann) Cleve				67		
*Gomphonema truncatum* Ehrenberg	9					
*Gomphosinica geitleri* (Kociolek & Stoermer) Kociolek et al.	9					
*Halamphora borealis* (Kützing) Levkov			47			
*Halamphora coraensis* (Foged) Levkov	5		43	64		
*Hannaea arcus* (Ehrenberg) Patrick		19				
*Hannaea superiorensis* Bixby, Edlund & Stoermer						109
*Hantzschia* Grunow			55			
*Hantzschia abundans* Lange-Bertalot	16					
*Hantzschia amphioxys* (Ehrenberg) Grunow			55			
*Hantzschia elongata* (Hantzsch) Grunow	16		55	75	108	
*Hantzschia hyperborea* (Grunow) Lange-Bertalot				75		
*Hantzschia vivacior* Lange-Bertalot			55	75		
*Hippodonta hungarica* (Grunow) Lange-Bertalot et al.				59		
*Hygroptera balfouiana* (Grunow) Krammer & Lange-Bertalot				59		125
Karayevia clevei var. bottnica (Cleve) Bukhtiyarova	3					
*Kobayasiella jaagii* (Meister) Lange-Bertalot				59	97	125
*Kobayasiella micropunctata* (Germain) Lange-Bertalot				59	97	125
*Kobayasiella okadae* (Skvortzow) Lange-Bertalot					97	
*Kobayasiella parasubtilissima* (Kobayasi & Nagumo) Lange-Bertalot		24				
*Kurtkrammeria aequalis* (W. Smith) Bahls	6					
*Kurtkrammeria lacusglacialis* Bahls		30				
*Kurtkrammeria neoamphioxys* (Krammer) Bahls					91	123
*Kurtkrammeria pseudoamphioxys* Bahls					91	123
*Kurtkrammeria treinishii* Bahls		30				
*Lacustriella* Lange-Bertalot, Kulikovskiy & Metzeltin					96	
*Lacustriella lacustris* (Gregory) Lange-Bertalot & Kulikovskiy					96	
*Lindavia affinis* (Grunow) Nakov et al.	1					109
*Lindavia antiqua* (W. Smith) Nakov et al.	1			56		109
*Lindavia intermedia* (Manguin) Nakov et al.				56	77	
*Lindavia michiganiana* (Skvortzow) Nakov et al.				56		
*Lindavia praetermissa* (Lund) Nakov et al.	1		38			
*Lindavia radiosa* (Grunow) De Toni & Forti				56		109
*Luticola* Mann		24				
*Luticola mutica* (Kützing) Mann	3	24	46			
*Mastogloia elliptica* (Agardh) Cleve			47			
*Mastogloia grevillei* W. Smith	3					
*Melosira nummuloides* (Dillwyn) Agardh		18				
*Meridion circulare* (Greville) Agardh			38			
*Meridion lineare* Williams	2					
*Muelleria bachmannii* (Hustedt) Spaulding & Stoermer						125
*Navicula* Bory	11					
*Navicula amphibola* Cleve	11			59		
*Navicula angusta* Grunow		32				128
*Navicula antonii* Lange-Bertalot	11					
*Navicula arctotenelloides* Lange-Bertalot & Metzeltin	11					
*Navicula aurora* Sovereign	12		48			
*Navicula caroliniae* Bahls	11					
*Navicula cryptocephala* Kützing			49		97	128
*Navicula cryptotenella* Lange-Bertalot	11	32				
*Navicula eidrigiana* Carter		32				127
*Navicula exilis* Kützing		32			97	
*Navicula gregaria* Donkin		32				127
Navicula hanseatica subsp. circumarctica Lange-Bertalot						127
Navicula hanseatica subsp. hanseatica Lange-Bertalot & Stachura						127
*Navicula kefvingensis* Ehrenberg			48			
*Navicula leptostriata* Jørgensen	11	32				
*Navicula lenzii* Hustedt	11					
*Navicula libonensis* Schoeman	11		48			
*Navicula lundii* Reichardt	11					
*Navicula notha* Wallace	11			68	97	128
*Navicula oblonga* (Kützing) Kützing			48			
*Navicula peregrina* (Ehrenberg) Kützing			48			
*Navicula radiosa* Kützing		32	49	68		128
*Navicula reichardtiana* Lange-Bertalot	11					
*Navicula reinhardtii* (Grunow) Grunow	11			68		
*Navicula rhynchocephala* Kützing			49			
*Navicula salinarum* Grunow						127
*Navicula schweigeri* Bahls	11					
*Navicula seibigiana* Lange-Bertalot	11					
*Navicula sieminskiae* Lange-Bertalot & Witkowski				68		
*Navicula slesvicensis* Grunow						127
*Navicula subconcentrica* Lange-Bertalot					97	
*Navicula subhamulata* Grunow in Van Heurck	11					
*Navicula tridentula* Krasske						128
*Navicula trilatera* Bahls	11		49			
*Navicula tripunctata* (O. F. Müller) Bory	11					
Navicula tripunctata var. arctica Patrick & Freese				68	97	128
*Navicula trivialis* Lange-Bertalot	11					127
*Navicula upsaliensis* (Grunow) M. Peragallo	11					
*Navicula vandamii* Schoeman & Archibald						127
*Navicula vaneei* Lange-Bertalot						127
*Navicula venerablis* Hohn & Hellerman					97	
*Navicula veneta* Kützing	11					
*Navicula viridulacalcis* Lange-Bertalot	11					
*Navicula vulpina* Kützing	12			68	97	128
*Navicula weberi* Bahls	11, 12					
*Navicula wildii* Lange-Bertalot	11					
*Navicymbula pusilla* (Grunow) Krammer			47			
*Neidiomorpha binodiformis* (Krammer) Cantonati et al.	10					
*Neidiopsis vekhovii* Lange-Bertalot & Genkal					98	
*Neidiopsis wulffii* (Petersen) Lange-Bertalot					98	
*Neidium* Pfitzer	10	27	46		99, 100	129
*Neidium affine* (Ehrenberg) Pfitzer	10	27			100	129
Neidium affine var. humerus Reimer					99	
Neidium affine var. longiceps (Gregory) Cleve	10				100	
Neidium affine var. undulatum (Grunow) Cleve				69		
*Neidium alaskaense* Foged					98	
*Neidium amphigomphus* (Ehrenberg) Pfitzer		27	46		99	
*Neidium ampliatum* (Ehrenberg) Krammer			46		99, 100	129
*Neidium apiculatum* Reimer	10				99	
*Neidium bergii* (Cleve-Euler) Krammer					100	
*Neidium bisulcatum* (Lagerstedt) Cleve	10		46		100	129
*Neidium dubium* (Ehrenberg) Cleve	10					
*Neidium fogedii* Bahls	10					
*Neidium fossum* Lefebvre & Hamilton		27		69	99	129
*Neidium hitchcockii* (Ehrenberg) Cleve					100	
*Neidium holstii* (Cleve) Krammer					98	
*Neidium ladogense* (Cleve) Foged					98	
*Neidium productum* (W. Smith) Cleve				69		129
*Neidium septentrionale* Cleve-Euler						129
*Neidium temperei* Reimer				69	100	129
*Nitzschia* Hassall	16	37		75		132
*Nitzschia acidoclinata* Lange-Bertalot	16				108	132
*Nitzschia alpina* Hustedt	16				108	132
*Nitzschia amphibia* Grunow	16		54	75		
*Nitzschia angustata* (W. Smith) Grunow		37		75		132
*Nitzschia bacillum* Hustedt	16					132
*Nitzschia commutata* Grunow			54			
*Nitzschia diversa* Hustedt	16					
Nitzschia fonticola var. pelagica Hustedt	16					
*Nitzschia fossilis* Grunow				75		132
*Nitzschia frauenfeldii* (Grunow) Grunow	16					
*Nitzschia frustulum* (Kützing) Grunow				75		132
*Nitzschia gessneri* Hustedt	16					
*Nitzschia gracilis* Hantzsch	16	37			108	
*Nitzschia inconspicua* Grunow	16					
*Nitzschia kittlii* Grunow			54			
*Nitzschia lacuum* Lange-Bertalot	16					
*Nitzschia lanceolata* W. Smith				75		
*Nitzschia liebetruthii* Rabenhorst		37				
*Nitzschia linearis* (Agardh) W. Smith		37	54			
*Nitzschia microcephala* Grunow		37				
*Nitzschia nana* Grunow						132
*Nitzschia palea* (Kützing) W. Smith	16		54			
Nitzschia palea var. tenuirostris Grunow					108	
*Nitzschia perminuta* (Grunow) Peragallo	16		54	75	108	132
*Nitzschia perspicua* Cholnoky			54			
*Nitzschia pseudofonticola* Hustedt		37				
*Nitzschia pura* Hustedt	16					
*Nitzschia pusilla* Grunow		37				
*Nitzschia radicula* Hustedt	16		54	75		
*Nitzschia recta* Hantzsch		37				
Nitzschia regula var. robusta Hustedt	16			75		132
*Nitzschia sinuata* (Thwaites in W. Smith) Grunow	16			75		
*Nitzschia suchlandtii* Hustedt						132
*Nitzschia vermicularis* (Kützing) Hantzsch	16					
*Nupela* Vyverman & Compére						110
*Nupela tenuicephala* (Hustedt) Lange-Bertalot		24				
*Odontidium mesodon* (Ehrenberg) Kützing		19				
*Orthoseira roeseana* (Rabenhorst) Pfitzer	1					109
*Peronia fibula* (Brébisson in Kützing) Ross		20			83	
*Pinnuavis* Bourrelly						126
*Pinnuavis elegans* (W. Smith) Okuno						126
*Pinnularia* Ehrenberg			70	70, 71	101, 102, 104	130
*Pinnularia acrosphaeria* W. Smith			51			
*Pinnularia anglica* Krammer	13					130
*Pinnularia biceps* Gregory	13				101	130
*Pinnularia birnirkiana* Patrick & Freese						130
*Pinnularia borealis* Ehrenberg	13				101	
Pinnularia borealis var. scalaris (Ehrenberg) Rabenhorst	13					
*Pinnularia brebissonii* (Kützing) Rabenhorst	13		49			
*Pinnularia crucifera* Cleve-Euler	13				102	130
*Pinnularia decrescens* (Grunow) Krammer		33		70		130
*Pinnularia divergens* W. Smith						130
Pinnularia divergens var. sublinearis Cleve					104	
*Pinnularia genkalii* Krammer & Lange-Bertalot			50	71	103	
*Pinnularia gibbiformis* Krammer			51			
Pinnularia graciloides var. triundulata (Fontell) Krammer			52			
*Pinnularia grunowii* Krammer				70	101	130
*Pinnularia ignobilis* (Krasske) Cleve-Euler			49			
*Pinnularia isostauron* (Ehrenberg) Cleve	13					
*Pinnularia ivaloensis* Krammer			52			
*Pinnularia krammeri* Metzeltin					101	
*Pinnularia lailaensis* Foged					104	
*Pinnularia lata* (Brébisson) Rabenhorst		33			101	
*Pinnularia lenticula* Cleve-Euler			49			
*Pinnularia lokana* Krammer				71		130
*Pinnularia lunata* Krammer & Lange-Bertalot	13					
*Pinnularia macilenta* Ehrenberg					102	
*Pinnularia marchica* Schönfelder					101	
*Pinnularia mesogongyla* Ehrenberg		34				
*Pinnularia microstauron* (Ehrenberg) Cleve	13	33			101	
Pinnularia microstauron var. angusta Krammer		33				
*Pinnularia neomajor* Krammer		35	50			
*Pinnularia nodosa* (Ehrenberg) W. Smith			52			
*Pinnularia obscura* Krasske	13		51			
*Pinnularia pseudogibba* Krammer				70		
*Pinnularia pseudosuchlandtii* Bahls	13			70		
*Pinnularia rabenhorstii* Hilse	13					
*Pinnularia rupestris* Hantzsch in Rabenhorst		35		71		
*Pinnularia septentrionalis* Krammer	13					
*Pinnularia sinistra* Krammer					102, 104	130
*Pinnularia spitsbergensis* Cleve			52	71	103	130
*Pinnularia stomatophora* (Grunow) Cleve		35	52			
*Pinnularia subcapitata* Gregory		33				
Pinnularia subcapitata var. elongata Krammer			51			
*Pinnularia subgibba* Krammer					102	
*Pinnularia subpulchra* Krammer			52			130
*Pinnularia sudetica* Hilse	13					
*Pinnularia transversa* (A. Schmidt) Mayer		34				
*Pinnularia turbulenta* (Cleve-Euler) Krammer	13					
*Pinnularia viridiformis* Krammer		34	49	71		
*Pinnularia viridis* (Nitzsch) Ehrenberg	13					
*Placoneis* Mereschkowsky				59		
*Placoneis abiskoensis* (Hustedt) Lange-Bertalot & Metzeltin	10					
*Placoneis amphibola* (Cleve) Cox	11			59		
*Placoneis elginensis* (Gregory) Cox			46			
*Placoneis explanata* (Hustedt) Mayama			46			
*Planothidium* Round & Bukhtiyarova			39			
*Planothidium apiculatum* (Patrick) Lange-Bertalot			39			
*Planothidium delicatulum* (Kützing) Round & Bukhtiyarova		24				
*Planothidium frequentissimum* (Lange-Bertalot) Lange-Bertalot					77	
*Platessa conspicua* (Mayer) Lange-Bertalot	3					
*Psammothidium curtissimum* (Carter) Aboal	3					
*Psammothidium daonense* (Lange-Bertalot) Lange-Bertalot	3					
*Psammothidium marginulatum* (Grunow) Bukhtiyarova & Round					77	110
*Psammothidium nivale* Potapova & Enache		24				
Pseudostaurosira brevistriata var. inflata (Pantocsek) Hartley	2					
*Reimeria* Kociolek & Stoermer	3					
*Rhopalodia gibba* (Ehrenberg) O. Müller	17	37	53	76		
*Rhopalodia operculata* (Agardh) Håkansson	17					
*Rossithidium petersenii* (Hustedt) Aboal					77	110
*Rossithidium pusillum* (Grunow) Round & Bukhtiyarova	3	24				
*Sellaphora* Mereschkowsky			46	59	89	125
*Sellaphora alastos* (Hohn & Hellerman) Lange-Bertalot & Metzeltin				59		
*Sellaphora laevissima* (Kützing) Mann	4					
*Sellaphora parapupula* Lange-Bertalot	4				89	125
*Sellaphora pupula* (Kützing) Mereschkowsky	4					125
*Sellaphora rectangularis* (Gregory) Lange-Bertalot & Metzeltin			46			125
*Semiorbis rotundus* Reid & Williams		20				
*Stauroforma* Flower, Jones & Round						109
*Stauroforma exiguiformis* (Lange-Bertalot) Flower, Jones & Round						109
*Stauroneis* Ehrenberg	14, 15					
*Stauroneis acidoclinata* Lange-Bertalot & Werum			47			
*Stauroneis acuta* W. Smith	15					
*Stauroneis akamina* Bahls	15					
*Stauroneis amphicephala* Kützing	15		47		106	131
*Stauroneis anceps* Ehrenberg					105, 106	
*Stauroneis angustilancea* Lange-Bertalot & Metzeltin					105	
*Stauroneis boyntoniae* Bahls					106	
*Stauroneis circumborealis* Lange-Bertalot & Krammer	14				107	
*Stauroneis conspicua* Metzeltin & Lange-Bertalot	15					
*Stauroneis fluminea* Patrick & Freese					106	
*Stauroneis gracilis* Ehrenberg	14		47	72	106	131
*Stauroneis heinii* Lange-Bertalot & Krammer	14	32			105	
*Stauroneis hyperborea* Lange-Bertalot & Krammer				73		131
*Stauroneis jarensis* Lange-Bertalot, Cavacini, Tagliaventi & Alfinito	15					131
*Stauroneis kootenai* Bahls	14					
*Stauroneis kriegeri* Patrick	14					
*Stauroneis kuelbsii* Lange-Bertalot				72	105	131
*Stauroneis lauenburgiana* Hustedt	14					
*Stauroneis livingstonii* Reimer					106	131
*Stauroneis neohyalina* (M. Peragallo & Brun) Lange-Bertalot & Krammer			47			
*Stauroneis pax* Bahls	15					
*Stauroneis phoenicenteron* (Nitzsch) Ehrenberg	14					
*Stauroneis pikuni* Bahls	15					
*Stauroneis prominula* (Grunow) Hustedt					106	
*Stauroneis reichardtii* Lange-Bertalot, Cavacini, Tagliaventi & Alfinito	15			72	106	131
*Stauroneis separanda* Lange-Bertalot & Werum	14					
*Stauroneis siberica* (Grunow) Lange-Bertalot & Krammer	15					
*Stauroneis silvahassiaca* Lange-Bertalot & Werum	15					
*Stauroneis smithii* Grunow	14					
Stauroneis smithii var. incisa Pantocsek						131
*Stauroneis superhyperborea* Van de Vijver, Beyens & Lange-Bertalot				74		
*Stauroneis vandevijveri* Bahls	15					
*Staurophora* Mereschkowsky						131
*Staurosira construens* Ehrenberg	2					
Staurosira construens var. venter (Ehrenberg) Hamilton	2					
*Staurosira oldenburgioides* (Lange-Bertalot) Kulikovskiy et al.	2					
*Staurosirella* Williams & Round	2			56		
*Staurosirella lapponica* (Grunow) Williams & Round	2					
*Staurosirella leptostauron* (Ehrenberg) Williams & Round	2					
*Staurosirella pinnata* (Ehrenberg) Williams & Round	2			56		
*Stenopterobia anceps* (Lewis) Brébisson in Van Heurck					108	
*Stenopterobia curvula* (W. Smith) Krammer			54			
*Stephanodiscus alpinus* Hustedt	1					
*Surirella brebissonii* Krammer & Lange-Bertalot						132
*Surirella linearis* W. Smith					108	
*Synedra* Ehrenberg	2					
Synedra acus var. delicatissima (W. Smith) Rabenhorst	2					
*Synedra famelica* Kützing			38			
*Tabellaria fenestrata* (Lyngbye) Kützing				56	77	109
*Tabellaria flocculosa* (Roth) Kützing	2	18	38	56	77	109
*Tabularia fasciculata* (Agardh) Williams & Round		19				
*Thalassiosira* Cleve		18				
*Ulnaria* (Kützing) Compére		19		56		109
*Ulnaria ulna* (Nitzsch) Compére	2					
Unknown genus		24			108	
